# Development of a 1,2,4-Triazole-Based Lead Tankyrase Inhibitor: Part II

**DOI:** 10.1021/acs.jmedchem.1c01264

**Published:** 2021-12-08

**Authors:** Ruben G. G. Leenders, Shoshy Alam Brinch, Sven T. Sowa, Enya Amundsen-Isaksen, Albert Galera-Prat, Sudarshan Murthy, Sjoerd Aertssen, Johannes N. Smits, Piotr Nieczypor, Eddy Damen, Anita Wegert, Marc Nazaré, Lari Lehtiö, Jo Waaler, Stefan Krauss

**Affiliations:** † Symeres, Kerkenbos 1013, 6546 BB Nijmegen, The Netherlands; ‡ Hybrid Technology Hub - Centre of Excellence, Institute of Basic Medical Sciences, University of Oslo, 0317 Oslo, Norway; § Department of Immunology and Transfusion Medicine, Oslo University Hospital, 0424 Oslo, Norway; ∥ Faculty of Biochemistry and Molecular Medicine, Biocenter Oulu, University of Oulu, 90014 Oulu, Finland; ⊥ Medicinal Chemistry, Leibniz-Forschungsinstitut für Molekulare Pharmakologie (FMP), Campus Berlin Buch, Robert-Roessle-Str. 10, 13125 Berlin, Germany

## Abstract

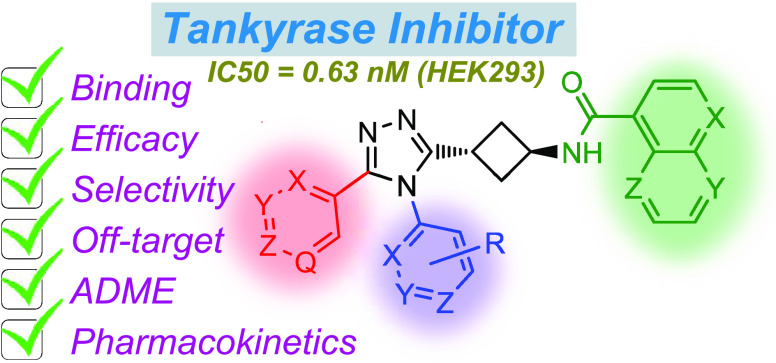

Tankyrase 1 and 2 (TNKS1/2) catalyze post-translational modification by poly-ADP-ribosylation of a plethora of target proteins. In this function, TNKS1/2 also impact the WNT/β-catenin and Hippo signaling pathways that are involved in numerous human disease conditions including cancer. Targeting TNKS1/2 with small-molecule inhibitors shows promising potential to modulate the involved pathways, thereby potentiating disease intervention. Based on our 1,2,4-triazole-based lead compound **1** (OM-1700), further structure–activity relationship analyses of East-, South- and West-single-point alterations and hybrids identified compound **24** (OM-153). Compound **24** showed picomolar IC_50_ inhibition in a cellular (HEK293) WNT/β-catenin signaling reporter assay, no off-target liabilities, overall favorable absorption, distribution, metabolism, and excretion (ADME) properties, and an improved pharmacokinetic profile in mice. Moreover, treatment with compound **24** induced dose-dependent biomarker engagement and reduced cell growth in the colon cancer cell line COLO 320DM.

## Introduction

Tankyrase 1 and tankyrase 2 (TNKS1/2) are members of the poly(ADP-ribose) polymerase (PARP) family of enzymes that regulate the turnover of specific target proteins through covalently linking the cellular redox metabolite NAD^+^ to target proteins in a process called poly-ADP-ribosylation (PARylation). The PAR chain produced in this post-translational modification is subsequently recognized by the E3 ubiquitin ligase ring finger protein 146 (RNF146) leading to poly-ubiquitination of the PARylated target proteins followed by proteasomal degradation.^1−4^ Independent of their catalytic activity, TNKS1/2 also provides scaffolding functions that are important in the formation of protein complexes.^5−8^ TNKS1/2 PARylate a plethora of target proteins including peroxisome proliferator-activated receptor-gamma coactivator 1 α (PGC-1α), telomeric repeat binding factor 1 (TRF1), phosphatase and tensin homologue (PTEN), AMP-activated protein kinase (AMPK), SRY-box transcription factor 9 (SOX9), and SH3 domain binding protein 2 (SH3BP2).^3,9−15^ In particular, TNKS1/2 regulate the turnover of AXIN1, AXIN2 (AXIN1/2) and of angiomotin (AMOT) proteins at the crossroad of the elementary wingless-type mammary tumor virus integration site (WNT)/β-catenin and Hippo signaling pathways, respectively.^3,14,16,17^ Hence, controlling the catalytic activity of tankyrase by pharmacological intervention provides an attractive tool for reducing WNT/β-catenin and Hippo signaling.^18,19^

Multiple potent TNKS1/2 inhibiting small molecules, including bicyclic lactams, compounds based on heteroalicyclic amide scaffolds, tricyclic fused ring systems and 1,2,4-triazole scaffolds, have been identified. These compounds block the catalytic domain of TNKS1/2, either by binding with high selectivity to the adenosine binding pocket (which differs from other members of the PARP family) or by binding to the more conserved nicotinamide pocket. The latter binding mode results in a lower selectivity across the PARP family. Other compounds target both pockets in the catalytic domain.^16,20−37^ Inhibitors based on the 1,2,4-triazole scaffold such as JW74,^38^ G007-LK,^21^ OD336,^28^ and OM-1700 (**1**)^39^ target the adenosine binding pocket of the TNKS1/2 catalytic domain with high selectivity and are therefore able to display selectivity over other members of the PARP family.

Despite the significant progress in developing TNKS1/2 inhibitors, there is currently no viable TNKS1/2 specific inhibitor in clinical practice for any disease indication. Concerns that have hampered clinical trials of TNKS1/2 inhibitors include earlier reports indicating intestinal toxicity^40,41^ and bone loss in mouse models,^13^ although other studies show a beneficial effect of tankyrase inhibition on fracture healing.^42^ Nevertheless, mice that have been treated for an extended time with a moderate dose of a tankyrase inhibitor have not shown visible adverse effects.^43^ Ongoing studies suggest that it is possible to overcome a potential biotarget toxicity and recently the tankyrase inhibitors E7449 (a dual inhibitor of PARP1/2 and TNKS1/2) and STP1002 have entered clinical trials with dose escalation studies in patients with advanced solid tumors.^44,45^ This clearly illustrates the potential of TNKS1/2 inhibitors and justifies the development of drugs directed toward TNKS1/2 inhibition with high potency and an optimized pharmacokinetic (PK) profile.

Here, we further optimize compounds based on the 1,2,4-triazole series as landmark compounds^21,28,38,39^ and we present compounds reaching picomolar IC_50_ values in a cellular WNT/β-catenin signaling reporter assay, while also showing optimized absorption, distribution, metabolism, excretion (ADME), and pharmacokinetic (PK) properties.

## Results and Discussion

### Compound Design Strategy

**Figure 1:**
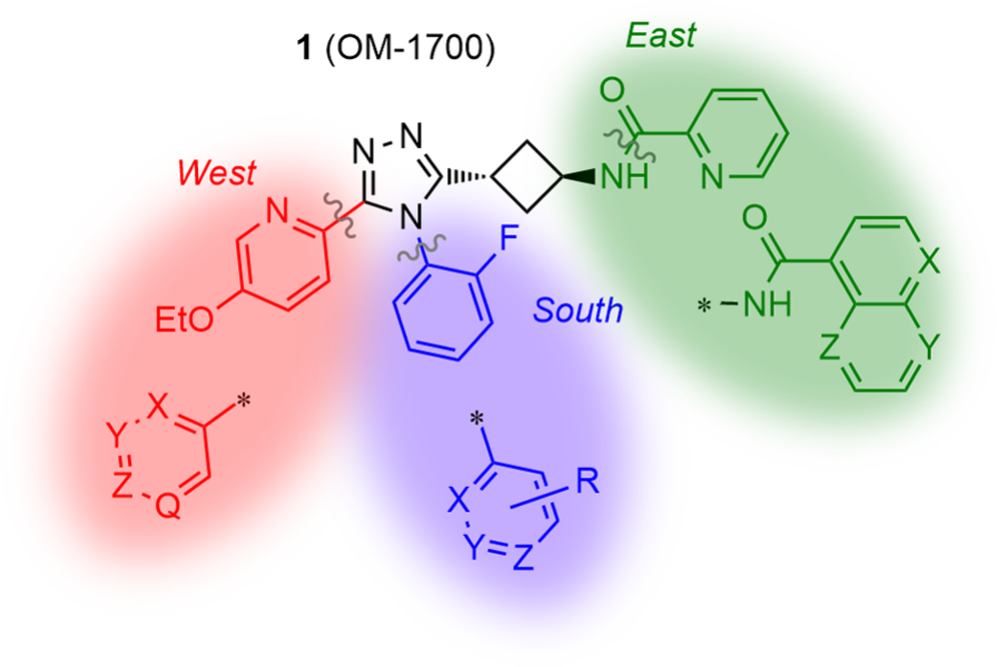
Lead compound **1** (OM-1700) and main modifications.

Starting from the previously described lead compound **1** (Figure 1)^39^ and having developed synthetic methodologies for preparing compound iterations in a modular fashion (see Supporting Experimentals for the general scheme of synthesis), we aimed at further improving the potency of tankyrase inhibition and optimizing ADME and pharmacokinetic properties of **1**. Optimization was motivated by the highly potent compounds discovered during the development of **1** (e.g., compounds **16**, **21**, and **27** as numbered in our preceding paper,^39^ see Chart S-1). In addition to this, the pharmacokinetic properties of **1**, primarily its half-life and exposure, necessitated further improvements.

In the process of making next-generation derivatives based on these previous compounds,^39^ we revisited South-, East-, and West-single-point mutations and hybrids thereof. From these structures, we selected six compounds based on their cellular activity and on the diversity of their molecular architecture. The properties of these six shortlisted compounds were evaluated in mouse peroral pharmacokinetic studies and compared with that of compound **1**. The best compound of this set was selected and further evaluated with respect to early ADME properties, off-target effects, binding affinity in the catalytic pocket of the TNKS2 protein, as well as inhibition of WNT/β-catenin signaling and proliferation in the colon cancer cell line COLO 320DM. The results from these experiments culminated in the identification of a new 1,2,4-triazole based lead tankyrase inhibitor.

### Structure–Activity Relationship (SAR) Investigation and Biological Evaluation

To further explore the SAR of the West- and South*-*regions of **1**, single-point modifications departing from **1** were synthesized (Table 1). From these compounds, we concluded that a gain in potency in our *in vitro* TNKS2 assay and cell-based WNT/β-catenin signaling assay cannot be attained with compounds possessing the East 2-pyridyl group while changing South/West groups, and we therefore focused on compounds with annulated aromatic heterocycles such as naphthyridines and quinoxalines^46^ (Table 2). Such variations enabled picomolar potencies as these moieties are able to form a significantly more efficient π–π-stacking interaction with His1048 and an additional hydrophobic interaction with Phe1035 as evidenced by the obtained co-crystal structures.^39^

**Table 1:**
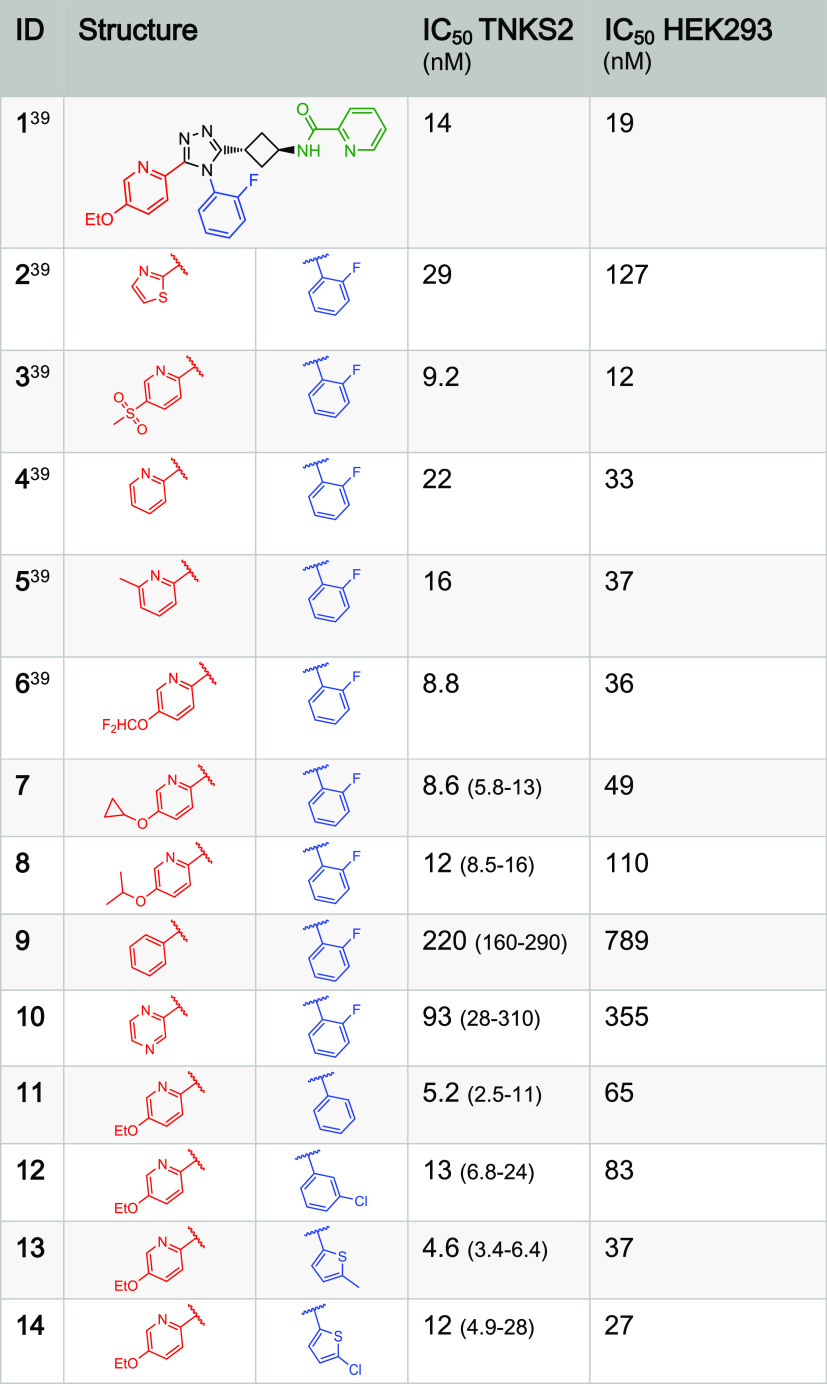
West Variations of **1** (OM-1700)^a^

a The IC_50_ values of the compounds of this work were determined with both the TNKS2 biochemical assay (quadruplicates used for each concentration tested, 95% confidence intervals are given in parentheses) and the cellular (HEK293) WNT/β-catenin signaling reporter assay (triplicates used for each concentration tested, T/F-tests were performed for the IC_50_ curve fitting; all *p* > 0.95).

**Table 2:**
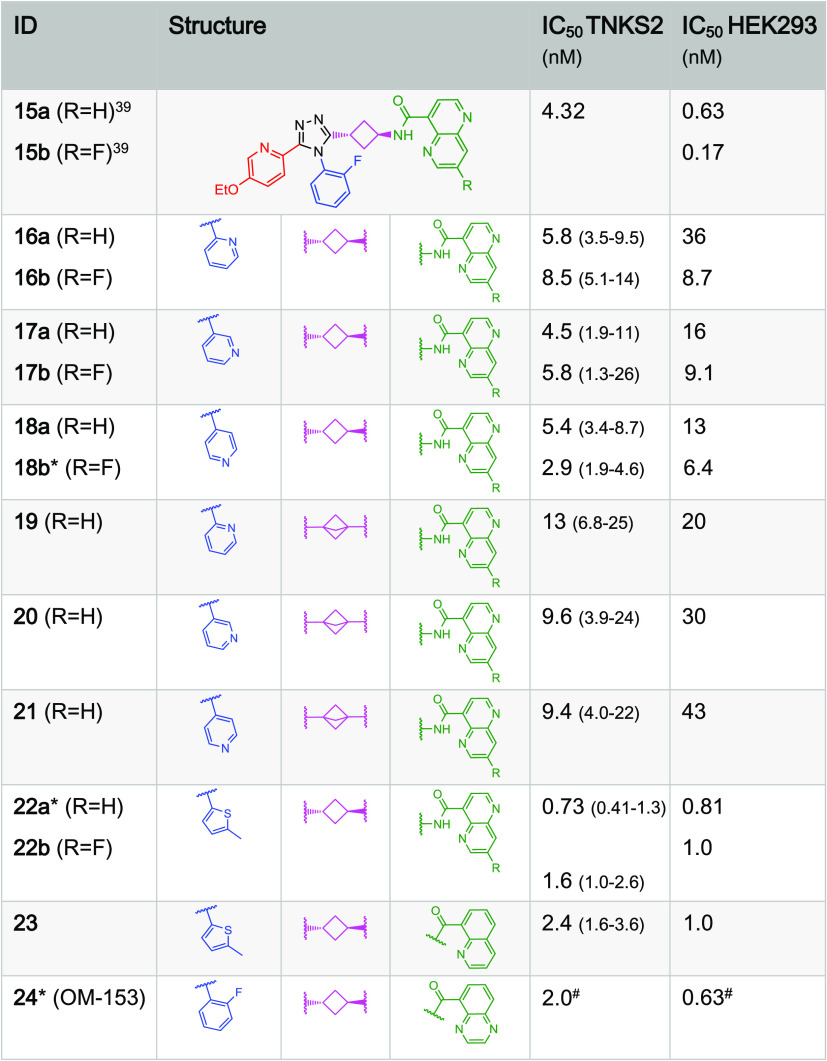
East Naphthyridines and Quinoxalines^a^

a The IC_50_ values of the compounds of this work were determined with both the TNKS2 biochemical assay (quadruplicates used for each concentration tested, 95% confidence intervals are given in parentheses) and the cellular (HEK293) WNT/β-catenin signaling reporter assay (triplicates used for each concentration tested, T/F-tests were performed for the IC_50_ curve fitting; all *p* > 0.95). * indicates a shortlisted compound. ^#^ averages of multiple independent measurements; standard error of the means (SEMs) are shown in Table 5.

First, we explored a series based on the East-side 1,5-naphthyridine, either with or without a 3-fluoro substituent (Table 2). Since compound **15a** was impaired by a high metabolic instability in mouse microsomes, we aimed at making the structure metabolically more stable by increasing the polarity with the introduction of a South-pyridyl-substitution and compounds **16**–**18** (Table 2) were prepared. In general, more polar compounds are expected to show greater metabolic stability. Compounds with such a South*-*pyridyl, however, proved to be less potent in the cellular WNT/β-catenin signaling reporter assay compared to **15a** and only compound **18b** with an IC_50_ value of 6 nM was selected for further characterization (Table 2). To improve potency of compounds **16**–**18** as earlier observed, a bicyclo[1.1.1]pentane core as an alternative to the cyclobutane influenced activity favorably (see, e.g., compound **19** as numbered in our preceding paper^39^). Unfortunately, here, compounds built on this bicyclo[1.1.1]pentane core lacked picomolar cellular activity when combined with a South*-*pyridyl moiety (compounds **19**–**21**, Table 2) and these South-pyridyl structures were abandoned. Furthermore, a South-methyl thiophene group was introduced as a phenyl bio-isostere^47^ (compounds **22** and **23**, Table 2), resulting in potent inhibitors of which compound **22a** was selected for further evaluation.

**Figure 2:**
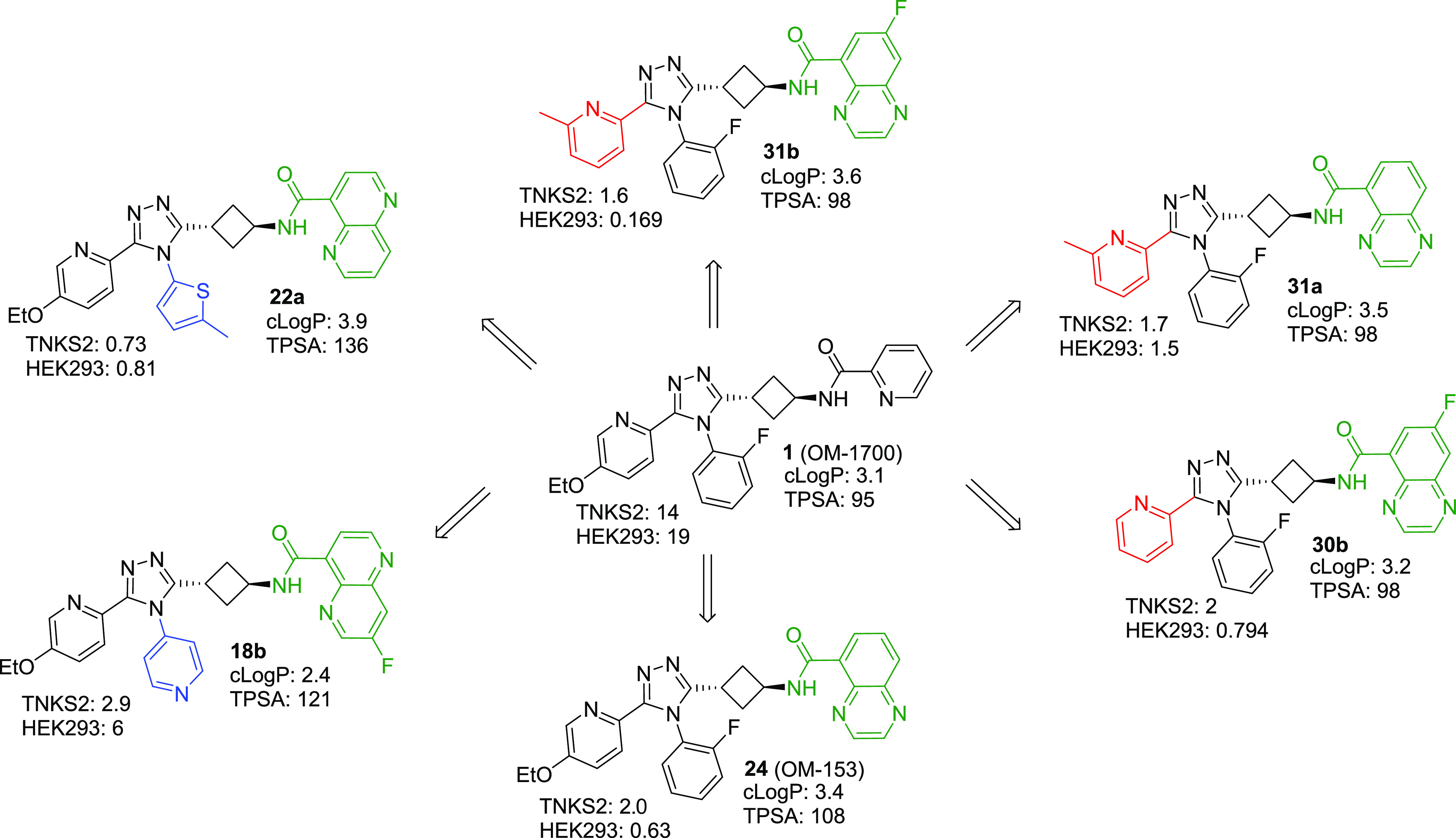
Short list of six compounds and **1** including their respective biochemical TNKS2 and cellular (HEK293) WNT/β-catenin signaling reporter assays IC_50_ values in nM. clog *P* and tPSA (in Å^2^) as calculated by DataWarrior v5.5.0. Moieties in color were different from **1**.

Finally, the introduction of an East quinoxaline moiety instead of the naphthyridine group (compound **24**) again led to picomolar cellular activity (IC_50_ = 0.63 nM), and we prepared further variations based on a quinoxaline core to interrogate this part of the pharmacophore. Variations of lead **24** by four fluorine- and methyl-substituted quinoxalines showed similar inhibitory activity (compounds **25**–**28**, Table 3) but by varying the West-moiety, highly efficacious inhibitors could be obtained with the quinoxaline East-group. Three of these compounds were selected for further profiling (compounds **30b**, **31a**, and **31b**). This resulted in a short list of six inhibitors with relatively high structural diversity (Figure 2).

**Table 3:**
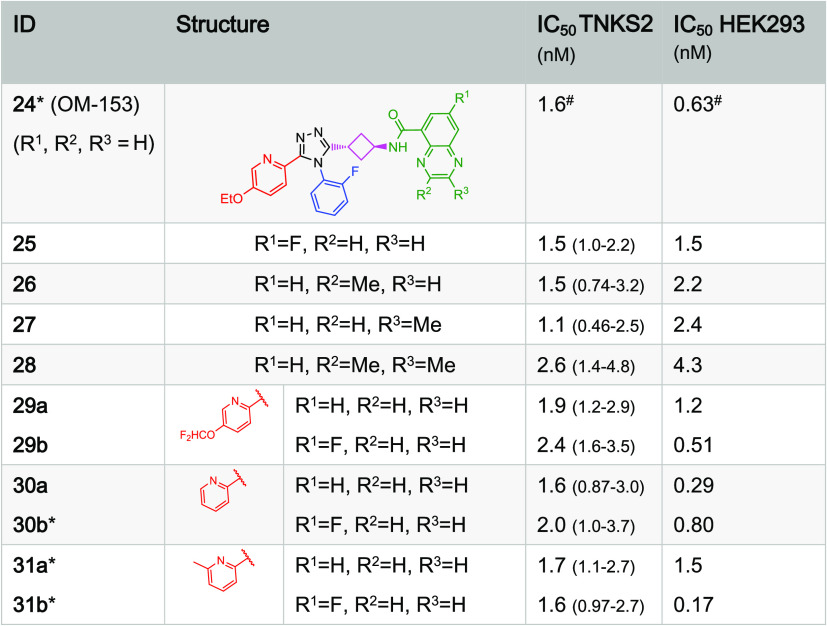
East Quinoxaline Variations^a^

a The IC_50_ values of the compounds of this work were determined with both the TNKS2 biochemical assay (quadruplicates used for each concentration tested, 95% confidence intervals are given in parentheses) and the cellular (HEK293) WNT/β-catenin signaling reporter assay (triplicates used for each concentration tested, T/F-tests were performed for the IC_50_ curve fitting; all *p* > 0.95). * indicates shortlisted compound. ^#^ averages of multiple independent measurements; SEMs are shown in Table 5.

Next, the set of six shortlisted compounds (Figure 2) was subjected to mouse peroral pharmacokinetic (PK) analyses and compared with **1** (Table 4). Here, compounds **22a** and **18b** displayed a decreased exposure (area under the curve, AUC) while compound **31a** showed an AUC similar to **1**. In contrast, compound **30b** showed a high AUC, however, with a small volume of distribution (0.33 L/kg), and consequently, none of these compounds were selected for further evaluation.

**Table 4:** Mouse PO PK 5 mg/kg and Kinetic Solubility Data of **1** and the Six Shortlisted Compounds

compound	*t*_1/2_ (h)	*t*_max_ (h)	*C*_max_ (ng/mL)	AUC 0 → *t* (ng/mL·h)	AUC 0 → ∞ (ng/mL·h)	MRT 0 → ∞ (h)	*V*_d_ (L/kg)	CL (L/h/kg)	solubility (μM)
**1** (OM-1700)^39^	0.67	0.25	3203	2384	2388	0.69	2.03	2.09	>80
**18b**	1.00	0.25	285	319	322	1.56	22.4	15.6	>80
**22a**	1.17	0.25	779	543	547	1.39	15.5	9.14	50
**24** (OM-153)	1.50	0.5	1967	4945	5038	2.39	2.15	0.99	31
**30b**	0.69	0.5	6512	15 083	15 105	1.93	0.33	0.33	>80
**31a**	0.76	0.25	2770	3401	3404	1.24	1.61	1.47	>80
**31b**	0.59	0.25	5796	5313	5316	1.47	0.80	0.94	13

Further, compounds **31b** and **24** showed improved pharmacokinetic properties compared to **1** (Table 4). The AUCs for **31b** and **24** were similar and about twice as high compared to the AUC for **1**. The peak value (*C*_max_) of exposure of **24** was approximately 3 times lower compared to the peak value for compound **31b**. In addition, **24** showed a more favorable volume of distribution and solubility compared to these data for compound **31b**. Compared to **1**, compound **24** possesses an AUC about twice as high and a peak value of about 60% of that of **1** resulting in a higher exposure with a lower peak level. In addition to this, efficacy in the cellular WNT/β-catenin signaling reporter assay was approximately 30 times higher while the clearance of **24** was about half of that of **1**.

In summary, based on the overall improved mouse PK profile and the enhanced potency in the cellular WNT/β-catenin signaling reporter assay, compound **24** was designated as the best derivative.

Subsequently, compound **24** was further characterized and compared to benchmark structure **1** with respect to early ADME properties and off-target effects. Tested ADME parameters such as kinetic solubility, Caco-2 permeability and efflux, microsomal stabilities, mouse plasma stability, and mouse plasma protein binding were found to be in line with **1**, while solubility was somewhat lower although in an acceptable range (Table 5).

**Table 5:** Profiling of Compound **24**

parameter	**1** (OM-1700)	**24** (OM-153)
efficacy		
TNKS1 (IC_50_, nM (pIC_50_ ± SEM))^a^	127 (6.90 ± 0.05)	13 (7.90 ± 0.054)
TNKS2 (IC_50_, nM (pIC_50_ ± SEM))^a^	14 (7.85 ± 0.04)	2.0 (8.71 ± 0.069)
HEK293 reporter assay (IC_50_, nM (pIC_50_ ± SEM))	19 (7.75 ± 0.067)	0.63 (9.22 ± 0.037)
COLO 320DM/RKO cells (GI_50_, nM)	650/>10 000	10/>10 000
ADME		
kinetic solubility PBS pH = 7 (μM)	>80	31
Caco-2 A–B: *P*_app_ (10^–6^ cm/s)	39.5	40.5
Caco-2 efflux ratio	0.61	0.64
microsomal stability human/mouse/dog CL_int_ (μL/min/mg protein)	<5/27/nd	18/22/3.8
mouse plasma stability *t*_1/2_ (min)	>120	>120
mouse PPB (%)	93.92	98.58
off-target		
PARPs^b^ PARP1/2/3/4/10/12/14/15 (IC_50_, μM)	>10	>10
hERG inhibition (IC_50_, μM)	>25	>25
Ames test	nongenotoxic	nongenotoxic
CYP3A4 inhibition (IC_50_, μM)	>25	>25
CYP induction (human PXR)	nd	nonactivator^b^^c^
Cerep Safety panel 44 targets@10 μM (inhibition)	clean, (A2A, 53%)	clean, (all <50%)
mouse pharmacokinetics		
PO PK mouse *t*_1/2_ (h)	0.67	1.5
PO PK mouse *C*_max_ (ng/mL)	3202	1967
PO PK mouse CL (L/h/kg)	2.09	0.99
PO PK mouse *V*_d_ (L/kg)	2.03	2.15
PO PK mouse AUC 0 → *t* (ng/mL)	2384	4945
calculated properties^d^		
MW (g/mol)	458.5	509.6
clog *P*	3.1	3.4
tPSA	95	108

a See Figure 1, Supporting Information.

b See Table 1, Supporting Information.

c Highest concentration, 100 μM.

d Calculated by DataWarrior v5.5.0.

**Figure 3:**
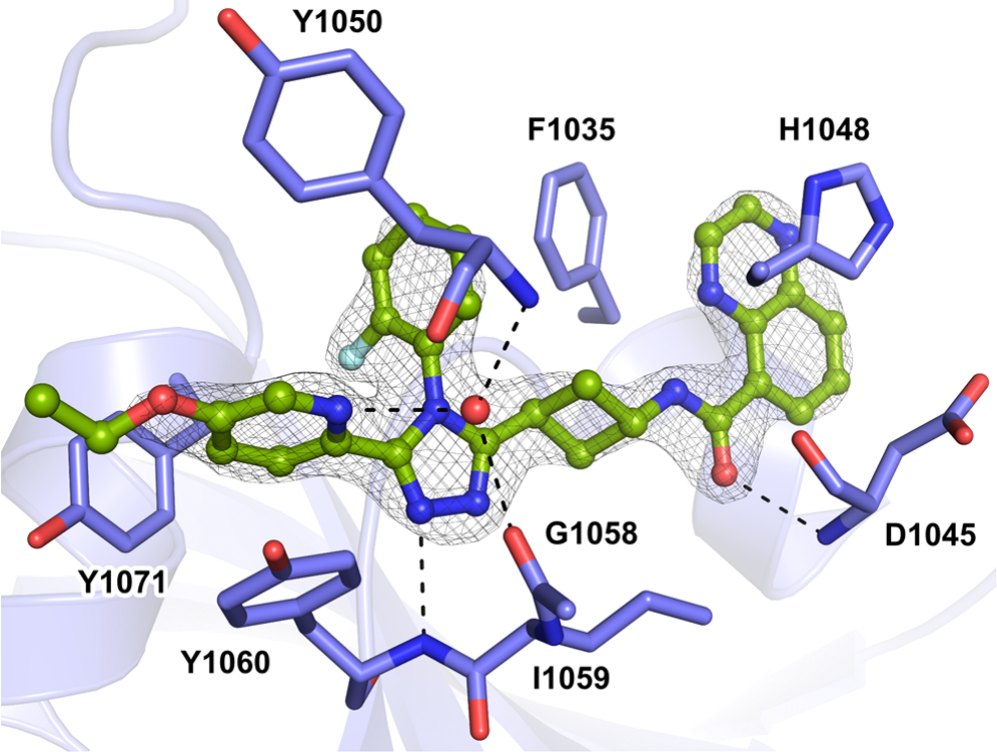
Co-crystal structure of TNKS2 with **24** (PDB 7O6X). The protein is shown in blue, and **24** in green. The dashed lines in black represent hydrogen bonds, and the red spheres represent water molecules. The σ_A_ weighted 2*F*_o_ – *F*_c_ electron density maps around the ligands are contoured at 1.8σ.

Next, we solved a co-crystal structure of TNKS2-**24** demonstrating that **24** binds to the NAD^+^ cleft of the catalytic domain in a similar manner to the previously reported analogue **1**(39) (Figure 3). The observed electron density is clear for the compound except for an apparently mobile ethoxy group extending toward the nicotinamide site. The previously identified hydrogen bonds of the scaffold^39^ were once more observed between **24** and the Tyr1060 and Asp1045 backbones, as well as with a water molecule. The quinoxaline moiety is forming π–π stacking interactions with both His1048 and Phe1035 providing improved binding affinity compared with that of **1**.

**Figure 4:**
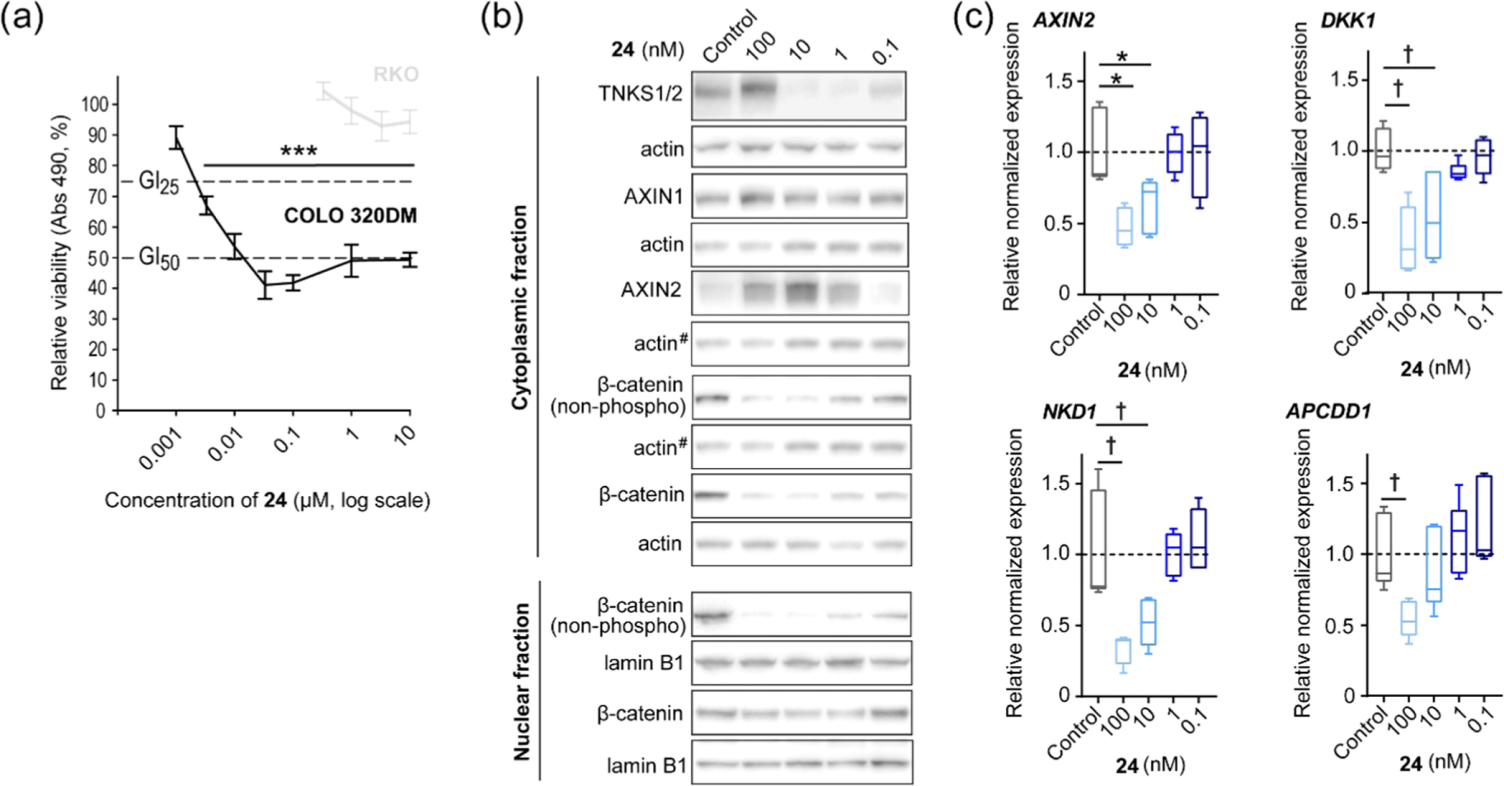
Compound **24** decreased cell growth and inhibited WNT/β-catenin signaling activity in COLO 320DM cells. (a) 3-(4,5-Dimethylthiazol-2-yl)-5-(3-carboxymethoxyphenyl)-2-(4-sulfophenyl)-2*H*-tetrazolium (MTS) colorimetric cell growth assay for various doses of **24** in APC^mutated^ COLO 320DM (black) and APC^wild-type^ RKO (gray) cells. After 5 days, the antiproliferative effect of compound treatment was measured at 490 nm. Mean value ± standard deviation (SD) for one representative experiment of more than three repeated assays, each with six replicates, are shown. Dotted lines depict 50% (GI_50_-value) and 25% (GI_25_-value) growth inhibition levels and control = 100% (0.1% dimethyl sulfoxide (DMSO)). (b) Representative immunoblots of cytoplasmic TNKS1/2, AXIN1, AXIN2, and cytoplasmic and nuclear transcriptionally active β*-*catenin (non-phospho) and β-catenin. Actin and lamin B1 show equal protein loading, while ^#^ indicates that the same actin immunoblot is used as loading control for both AXIN2 and β-catenin. For (b) and (c), control = 0.001% DMSO. (c) Real-time RT-qPCR analyses of WNT/β*-*catenin signaling target genes (*AXIN2*, *DKK1*, *NKD1*, and *APCDD1*). Boxplots show median, first and third quartiles, and maximum and minimum whiskers for combined data from three independent experiments with three replicates each. Dotted lines depict the control mean value = 1. For (a) and (c), analysis of variance (ANOVA) tests (Holm–Sidak method, versus control) are indicated by *** (*p* < 0.001) and * (*p* < 0.05), while ANOVA on ranks tests (Dunn’s method, versus control) are indicated by ^†^ (*p* < 0.05).

Finally, to complete the analysis of compound **24**, COLO 320DM cells, likely of neuroendocrinal origin,^48^ were treated with various doses of **24** to evaluate the efficacy in reducing canonical WNT/β*-*catenin signaling and the potential as an antiproliferative agent in this cancer cell line. Previously, we have shown that tankyrase inhibition can block WNT/β*-*catenin signaling and attenuate proliferation and viability in cancer cell lines *in vitro* and *in vivo*, including COLO 320DM that is commonly used for testing tankyrase inhibitors.^28,39,49,50^ Treatment of COLO 320DM cells with **24** decreased viability with a GI_50_ value of 10.1 nM and a GI_25_ value of 2.5 nM (for **1**, these values were 650 and 94 nM, respectively^39^), while APC^wild-type^ RKO cells as a control colon cancer line were only modestly affected by treatment with compound **24** (Figure 4a). As previously observed upon tankyrase inhibition,^49,51^ treatment with **24** dose-dependently either increased or decreased the TNKS1/2 protein levels (Figure 4b). Compound **24** also stabilized AXIN1 and AXIN2 proteins and reduced the level of transcriptionally active β*-*catenin (nonphosphorylated) in both cytoplasmic and nuclear fractions (Figure 4b). In addition, real-time qRT-PCR analyses revealed reduced levels of transcripts of the WNT/β*-*catenin signaling target genes *AXIN2, DKK1, NKD1*, and *APCDD1* in a dose-dependent manner (Figure 4c). Collectively, these results showed that **24** both potently and specifically can inhibit WNT/β*-*catenin signaling activity and block proliferation in COLO 320DM cells.

## Conclusions

In this further development of our structure-guided lead optimization program of 1,2,4-triazole-based tankyrase inhibitors, we have extensively improved previous lead compound **1** to lead candidate compound **24**.^52^ We showed that compound **24** possesses improved binding affinity in the catalytic pocket of the TNKS2 protein with concurrently modest selectivity over TNKS1, picomolar IC_50_ activity in the cellular WNT/β-catenin signaling reporter assay, a clean off-target safety profile, good ADME properties, an optimized mouse PK profile, and potent inhibition of WNT/β-catenin signaling and proliferation in COLO 320DM. These results justify testing compound **24** in a pharmacodynamics setting as well as in toxicity models (publication pending).

## Experimental Section

### General Methods

NMR spectra were recorded on a 400 MHz spectrometer with tetramethylsilane as internal standards. Coupling constants are given in hertz. Peaks are reported as singlet (s), doublet (d), triplet (t), quartet (q), quintet (p), sextet (h), septet (hept), multiplet (m), or a combination thereof; br stands for broad.

Liquid chromatography/mass spectroscopy (LC/MS) chromatograms mass spectra were recorded using electrospray ionization (ESI) in positive or negative ionization mode on Agilent 1260 Bin: pump, G1312B, degasser; autosampler; ColCom; DAD G1315C; MSD G6130B ESI; eluent A, acetonitrile; eluent B, 10 mM ammonium bicarbonate in water (base mode) or 0.1% formic acid in water (acid mode). High-resolution mass spectra (HRMS) were recorded with an LC-MS Q Exactive Focus high-resolution mass spectrometer (Thermo Scientific). Calibration was done with the Pierce calibration solutions containing 1-butylamine, caffeine, MRFA, and Ultramark 1621 (positive mode) and the Pierce calibration solution containing sodium dodecyl sulfate, sodium taurocholate, and Ultramark 1621 (negative mode). Analysis: 1 μL of a 10 μg/mL sample in MeCN/DMSO 99:1 is injected and data are acquired under full MS mode (resolution 70 000 FWHM at 200 Da) over the mass range *m*/*z* of 150–2000. Standard ESI conditions compatible with the flow rate are applied: spray voltage, 3.5 kV; auxiliary gas heater temperature, 463 °C; capillary temperature, 280 °C; sheath gas, 58; auxiliary gas, 16; sweep gas, 3; S-lens radio frequency (RF) level, 50. Mass scan range is 150–2000 *m*/*z*. Mass resolution is set at 70 000 (<3 ppm mass accuracy). Data are evaluated using Xcalibur Qual Browser version 4.2.47 (Thermo Fisher).

All test compounds were >95% pure by LC/MS and ^1^H NMR analyses. All spectra as well as preparation of the intermediates and general procedures are given in the Supporting Experimentals.

#### *N*-(*trans-*3-(5-(5-Cyclopropoxypyridin-2-yl)-4-(2-fluorophenyl)-4*H-*1,2,4-triazol-3-yl)cyclobutyl)picolinamide (**7**)

The title compound was prepared according to general procedure F as a white solid (24.3 mg, 75%). LC/MS (ESI) *m*/*z* for C_26_H_23_N_6_O_2_F 470 (calculated) 471 ([M + H]^+^, found). ^1^H NMR (400 MHz, CDCl_3_) δ 8.56–8.49 (m, 1H), 8.26–8.13 (m, 3H), 7.99 (s, 1H), 7.83 (td, *J* = 7.7, 1.7 Hz, 1H), 7.50–7.37 (m, 3H), 7.24–7.14 (m, 3H), 4.82–4.71 (m, 1H), 3.74 (tt, *J* = 6.1, 3.1 Hz, 1H), 3.46 (tt, *J* = 10.3, 5.3 Hz, 1H), 3.11–2.96 (m, 2H), 2.52–2.35 (m, 2H), 0.84–0.71 (m, 4H). HRMS *m*/*z* [M + H]^+^: 471.19393 (calculated), 471.1929 (found), Δ = −2.28 ppm.

#### *N*-(*trans-*3-(4-(2-Fluorophenyl)-5-(5-isopropoxypyridin-2-yl)-4*H-*1,2,4-triazol-3-yl)cyclobutyl)picolinamide (**8**)

The title compound was prepared according to general procedure F as a white solid (18.3 mg, 77%). LC/MS (ESI) *m*/*z* for C_26_H_25_N_6_O_2_F 472 (calculated) 473 ([M + H]^+^, found). ^1^H NMR (400 MHz, CDCl_3_) δ 8.57–8.48 (m, 1H), 8.21 (d, *J* = 6.9 Hz, 1H), 8.18–8.08 (m, 2H), 7.91–7.79 (m, 2H), 7.48–7.38 (m, 2H), 7.25–7.14 (m, 4H), 4.76 (h, *J* = 7.2 Hz, 1H), 4.54 (h, *J* = 6.0 Hz, 1H), 3.47 (tt, *J* = 10.1, 5.8 Hz, 1H), 3.11–2.97 (m, 2H), 2.52–2.36 (m, 2H), 1.32 (d, *J* = 6.0 Hz, 6H). HRMS *m*/*z* [M + H]^+^: 473.20958 (calculated), 473.2089 (found), Δ = −1.42 ppm.

#### *N***-(***trans-*3-(4-(2-Fluorophenyl)-5-phenyl-4*H-*1,2,4-triazol-3-yl)cyclobutyl)picolinamide (**9**)

The title compound was prepared according to general procedure F as a white solid (21 mg, 84%). LC/MS (ESI) *m*/*z* for C_24_H_20_N_5_OF 413 (calculated) 414 ([M + H]^+^, found). ^1^H NMR (400 MHz, CDCl_3_) δ 8.56–8.50 (m, 1H), 8.23 (d, *J* = 6.8 Hz, 1H), 8.17 (dt, *J* = 7.9, 1.1 Hz, 1H), 7.84 (td, *J* = 7.7, 1.7 Hz, 1H), 7.53–7.46 (m, 1H), 7.46–7.39 (m, 3H), 7.39–7.32 (m, 1H), 7.32–7.27 (m, 2H), 7.26–7.21 (m, 2H), 7.21–7.13 (m, 1H), 4.83–4.70 (m, 1H), 3.49 (tt, *J* = 10.2, 5.6 Hz, 1H), 3.11–2.95 (m, 2H), 2.52–2.40 (m, 2H). HRMS *m*/*z* [M + H]^+^: 414.17246 (calculated), 414.1714 (found), Δ = −2.46 ppm.

#### *N*-(*trans-*3-(4-(-2-Fluorophenyl)-5-(pyrazin-2-yl)-4*H-*1,2,4-triazol-3-yl)cyclobutyl)picolinamide (**10**)

The title compound was prepared according to general procedure F as a white solid (7.9 mg, 21%). LC/MS (ESI) *m*/*z* for C_22_H_18_FN_7_O 415 (calculated) 416 ([M + H]^+^, found). ^1^H NMR (400 MHz, CDCl_3_) δ 9.52 (d, *J* = 1.8 Hz, 1H), 8.53 (d, *J* = 4.4 Hz, 1H), 8.49 (d, *J* = 2.5 Hz, 1H), 8.22–8.14 (m, 2H), 7.84 (td, *J* = 7.7, 1.7 Hz, 1H), 7.54–7.39 (m, 2H), 7.22 (td, *J* = 8.1, 7.5, 1.9 Hz, 3H), 4.83–4.74 (m, 1H), 3.52–3.46 (m, 1H), 3.07–3.02 (m, 1H), 2.56–2.41 (m, 2H), 1.55 (s, 2H). HRMS *m*/*z* [M + H]^+^: 416.16296 (calculated), 416.1620 (found), Δ = −2.40 ppm.

#### *N*-(*trans-*3-(5-(5-Ethoxypyridin-2-yl)-4-phenyl-4*H-*1,2,4-triazol-3-yl)cyclobutyl)picolinamide (**11**)

The title compound was prepared according to general procedure F as a white solid (30 mg, 52%). LC/MS (ESI) *m*/*z* for C_25_H_24_N_6_O_2_ 440 (calculated) 441 ([M + H]^+^, found). ^1^H NMR (400 MHz, CDCl_3_) δ 8.53 (dq, *J* = 4.8, 1.1 Hz, 1H), 8.21 (d, *J* = 7.0 Hz, 1H), 8.16 (dt, *J* = 7.8, 1.2 Hz, 1H), 8.01 (d, *J* = 8.8 Hz, 1H), 7.94 (d, *J* = 2.9 Hz, 1H), 7.84 (td, *J* = 7.7, 1.7 Hz, 1H), 7.46–7.38 (m, 4H), 7.21 (dd, *J* = 8.7, 2.9 Hz, 1H), 7.17–7.12 (m, 2H), 4.76 (h, *J* = 6.5 Hz, 1H), 4.04 (q, *J* = 7.0 Hz, 2H), 3.50 (dddd, *J* = 10.4, 8.9, 6.2, 5.0 Hz, 1H), 3.09–2.98 (m, 2H), 2.47–2.37 (m, 2H), 1.40 (t, *J* = 7.0 Hz, 3H). HRMS *m*/*z* [M + H]^+^: 441.20335 (calculated), 441.2025 (found), Δ = −1.86 ppm.

#### *N*-(*trans-*3-(4-(3-Chlorophenyl)-5-(5-ethoxypyridin-2-yl)-4*H-*1,2,4-triazol-3-yl)cyclobutyl)picolinamide (**12**)

The title compound was prepared according to general procedure F as a white solid (14.3 mg, 74%). LC/MS (ESI) *m*/*z* for C_25_H_23_N_6_O_2_Cl 474/476 (calculated) 475/477 ([M + H]^+^, found). ^1^H NMR (400 MHz, CDCl_3_) δ 8.53 (dt, *J* = 4.7, 1.3 Hz, 1H), 8.22 (d, *J* = 7.0 Hz, 1H), 8.17 (dt, *J* = 7.7, 1.0 Hz, 1H), 8.10 (d, *J* = 8.7 Hz, 1H), 7.93 (s, 1H), 7.84 (td, *J* = 7.7, 1.7 Hz, 1H), 7.46–7.40 (m, 2H), 7.36 (t, *J* = 8.0 Hz, 1H), 7.23 (dd, *J* = 8.7, 2.7 Hz, 1H), 7.18 (t, *J* = 2.0 Hz, 1H), 7.07 (dt, *J* = 8.0, 1.4 Hz, 1H), 4.78 (h, *J* = 7.0 Hz, 1H), 4.05 (q, *J* = 7.0 Hz, 2H), 3.53–3.42 (m, 1H), 3.09–2.97 (m, 2H), 2.51–2.39 (m, 2H), 1.41 (t, *J* = 7.0 Hz, 3H). HRMS *m*/*z* [M + H]^+^: 475.16438 (calculated), 475.1636 (found), Δ = −1.73 ppm.

#### *N*-(*trans-*3-(5-(5-Ethoxypyridin-2-yl)-4-(5-methylthiophen-2-yl)-4*H-*1,2,4-triazol-3-yl)cyclobutyl)picolinamide (**13**)

The title compound was prepared according to general procedure F as a white solid (19 mg, 85%). LC/MS (ESI) *m*/*z* for C_24_H_24_N_6_O_2_S 460 (calculated) 461 ([M + H]^+^, found). ^1^H NMR (400 MHz, CDCl_3_) δ 8.57–8.52 (m, 1H), 8.25 (d, *J* = 7.1 Hz, 1H), 8.18 (dt, *J* = 7.8, 1.1 Hz, 1H), 8.12 (d, *J* = 2.9 Hz, 1H), 7.95 (d, *J* = 8.7 Hz, 1H), 7.85 (td, *J* = 7.7, 1.7 Hz, 1H), 7.43 (ddd, *J* = 7.7, 4.8, 1.3 Hz, 1H), 7.22 (dd, *J* = 8.7, 3.0 Hz, 1H), 6.70 (d, *J* = 3.6 Hz, 1H), 6.62 (dd, *J* = 3.6, 1.3 Hz, 1H), 4.78 (q, *J* = 7.1 Hz, 1H), 4.08 (q, *J* = 6.9 Hz, 2H), 3.65–3.56 (m, 1H), 3.11–3.01 (m, 2H), 2.56–2.49 (m, 2H), 2.47 (d, *J* = 1.1 Hz, 3H), 1.43 (t, *J* = 7.0 Hz, 3H). HRMS *m*/*z* [M + H]^+^: 461.17542 (calculated), 461.1746 (found), Δ = −1.85 ppm.

#### *N*-(*trans-*3-(4-(5-Chlorothiophen-2-yl)-5-(5-ethoxypyridin-2-yl)-4*H-*1,2,4-triazol-3-yl)cyclobutyl)picolinamide (**14**)

The title compound was prepared according to general procedure F as a white solid (14.9 mg, 103%). LC/MS (ESI) *m*/*z* for C_23_H_21_N_6_O_2_SCl 480/482 (calculated) 481/483 ([M + H]^+^, found). ^1^H NMR (400 MHz, CDCl_3_) δ 8.55 (dt, *J* = 4.7, 1.4 Hz, 1H), 8.26 (d, *J* = 6.9 Hz, 1H), 8.18 (dt, *J* = 7.8, 1.1 Hz, 1H), 8.09 (d, *J* = 2.9 Hz, 1H), 8.06 (d, *J* = 8.8 Hz, 1H), 7.85 (td, *J* = 7.7, 1.7 Hz, 1H), 7.43 (ddd, *J* = 7.8, 4.7, 1.3 Hz, 1H), 7.25–7.20 (m, 1H), 6.82 (d, *J* = 4.0 Hz, 1H), 6.72 (d, *J* = 4.0 Hz, 1H), 4.79 (q, *J* = 7.2 Hz, 1H), 4.09 (q, *J* = 6.9 Hz, 2H), 3.63–3.56 (m, 1H), 3.11–3.01 (m, 2H), 2.59–2.48 (m, 2H), 1.43 (t, *J* = 7.0 Hz, 3H). HRMS *m*/*z* [M + H]^+^: 481.12080 (calculated), 481.1201 (found), Δ = −1.14 ppm.

#### *N*-(*trans-*3-(5-(5-Ethoxypyridin-2-yl)-4-(pyridin-2-yl)-4*H-*1,2,4-triazol-3-yl)cyclobutyl)-1,5-naphthyridine-4-carboxamide (**16a**)

The title compound was prepared according to general procedure F as an off-white solid (13.7 mg, 55%). LC/MS (ESI) *m*/*z* for C_27_H_24_N_8_O_2_ 492 (calculated) 493 ([M + H]^+^, found). ^1^H NMR (400 MHz, CDCl_3_) δ 11.32 (d, *J* = 6.0 Hz, 1H), 9.14 (d, *J* = 4.4 Hz, 1H), 8.99 (dd, *J* = 4.3, 1.8 Hz, 1H), 8.59–8.52 (m, 3H), 8.17 (d, *J* = 8.8 Hz, 1H), 7.87 (d, *J* = 2.9 Hz, 1H), 7.79 (td, *J* = 7.7, 1.9 Hz, 1H), 7.75 (dd, *J* = 8.5, 4.2 Hz, 1H), 7.37 (ddd, *J* = 7.5, 4.9, 1.0 Hz, 1H), 7.26–7.22 (m, 1H), 7.21–7.16 (m, 1H), 4.86–4.73 (m, 1H), 4.05 (q, *J* = 6.9 Hz, 2H), 3.80–3.69 (m, 1H), 3.11–3.01 (m, 2H), 2.57–2.46 (m, 2H), 1.41 (t, *J* = 7.0 Hz, 3H). HRMS *m*/*z* [M + H]^+^: 493.20950 (calculated), 493.2085 (found), Δ = −1.99 ppm.

#### *N*-(*trans-*3-(5-(5-Ethoxypyridin-2-yl)-4-(pyridin-2-yl)-4*H-*1,2,4-triazol-3-yl)cyclobutyl)-7-fluoro-1,5-naphthyridine-4-carboxamide (**16b**)

The title compound was prepared according to general procedure F as a white solid (17.9 mg, 69%). LC/MS (ESI) *m*/*z* for C_27_H_23_N_8_O_2_F 510 (calculated) 511 ([M + H]^+^, found). ^1^H NMR (400 MHz, CDCl_3_) δ 10.81 (d, *J* = 6.0 Hz, 1H), 9.15 (d, *J* = 4.5 Hz, 1H), 8.92 (d, *J* = 2.9 Hz, 1H), 8.56 (dd, *J* = 5.1, 1.9 Hz, 1H), 8.52 (d, *J* = 4.4 Hz, 1H), 8.22–8.18 (m, 1H), 8.17 (d, *J* = 9.0 Hz, 1H), 7.87 (d, *J* = 2.8 Hz, 1H), 7.79 (td, *J* = 7.7, 1.9 Hz, 1H), 7.37 (dd, *J* = 7.5, 4.8 Hz, 1H), 7.26–7.21 (m, 1H), 7.18 (d, *J* = 8.0 Hz, 1H), 4.80 (h, *J* = 6.9 Hz, 1H), 4.05 (q, *J* = 7.0 Hz, 2H), 3.72 (tt, *J* = 10.2, 5.3 Hz, 1H), 3.06 (ddd, *J* = 13.2, 8.0, 5.3 Hz, 2H), 2.50 (ddd, *J* = 12.6, 9.5, 6.2 Hz, 2H), 1.41 (t, *J* = 7.0 Hz, 3H). HRMS *m*/*z* [M + H]^+^: 511.20008 (calculated), 511.1991 (found), Δ = −1.86 ppm.

#### *N*-(*trans-*3-(5-(5-Ethoxypyridin-2-yl)-4-(pyridin-3-yl)-4*H-*1,2,4-triazol-3-yl)cyclobutyl)-1,5-naphthyridine-4-carboxamide (**17a**)

The title compound was prepared according to general procedure F as a white solid (12.6 mg, 50%). LC/MS (ESI) *m*/*z* for C_27_H_24_N_8_O_2_ 492 (calculated) 493 ([M + H]^+^, found). ^1^H NMR (400 MHz, CDCl_3_) δ 11.31 (d, *J* = 6.0 Hz, 1H), 9.14 (d, *J* = 4.5 Hz, 1H), 8.98 (dd, *J* = 4.3, 1.8 Hz, 1H), 8.68 (dd, *J* = 4.8, 1.5 Hz, 1H), 8.59–8.52 (m, 2H), 8.48 (d, *J* = 2.5 Hz, 1H), 8.17 (d, *J* = 8.8 Hz, 1H), 7.87 (d, *J* = 2.9 Hz, 1H), 7.74 (dd, *J* = 8.6, 4.2 Hz, 1H), 7.58 (ddd, *J* = 8.1, 2.5, 1.5 Hz, 1H), 7.41 (dd, *J* = 8.0, 4.7 Hz, 1H), 7.24 (dd, *J* = 8.7, 2.9 Hz, 1H), 4.86 (dtd, *J* = 8.7, 6.9, 5.2 Hz, 1H), 4.05 (q, *J* = 7.0 Hz, 2H), 3.58–3.47 (m, 1H), 3.14–3.03 (m, 2H), 2.62–2.51 (m, 2H), 1.41 (t, *J* = 7.0 Hz, 3H). HRMS *m*/*z* [M + H]^+^: 493.20950 (calculated), 493.2084 (found), Δ = −2.32 ppm.

#### *N*-(*trans-*3-(5-(5-Ethoxypyridin-2-yl)-4-(pyridin-3-yl)-4*H-*1,2,4-triazol-3-yl)cyclobutyl)-7-fluoro-1,5-naphthyridine-4-carboxamide (**17b**)

The title compound was prepared according to general procedure F as a white solid (22 mg, 85%). LC/MS (ESI) *m*/*z* for C_27_H_23_N_8_O_2_F 510 (calculated) 511 ([M + H]^+^, found). ^1^H NMR (400 MHz, CDCl_3_) δ 10.83 (d, *J* = 6.0 Hz, 1H), 9.15 (d, *J* = 4.4 Hz, 1H), 8.91 (d, *J* = 2.8 Hz, 1H), 8.69 (dd, *J* = 4.8, 1.5 Hz, 1H), 8.52 (d, *J* = 4.4 Hz, 1H), 8.48 (d, *J* = 2.5 Hz, 1H), 8.23–8.18 (m, 1H), 8.17 (d, *J* = 8.5 Hz, 1H), 7.87 (d, *J* = 2.8 Hz, 1H), 7.57 (dt, *J* = 8.1, 2.0 Hz, 1H), 7.41 (dd, *J* = 8.2, 4.8 Hz, 1H), 7.26–7.21 (m, 1H), 4.92–4.81 (m, 1H), 4.05 (q, *J* = 7.0 Hz, 2H), 3.56–3.45 (m, 1H), 3.14–3.03 (m, 2H), 2.60–2.50 (m, 2H), 1.41 (t, *J* = 7.0 Hz, 3H). HRMS *m*/*z* [M + H]^+^: 511.20008 (calculated), 511.1991 (found), Δ = −1.95 ppm.

#### *N*-(*trans-*3-(5-(5-Ethoxypyridin-2-yl)-4-(pyridin-4-yl)-4*H-*1,2,4-triazol-3-yl)cyclobutyl)-1,5-naphthyridine-4-carboxamide (**18a**)

The title compound was prepared according to general procedure F as a white solid (12.7 mg, 51%). LC/MS (ESI) *m*/*z* for C_27_H_24_N_8_O_2_ 492 (calculated) 493 ([M + H]^+^, found). ^1^H NMR (400 MHz, CDCl_3_) δ 11.31 (d, *J* = 6.0 Hz, 1H), 9.15 (d, *J* = 4.4 Hz, 1H), 8.97 (dd, *J* = 4.3, 1.8 Hz, 1H), 8.75–8.69 (m, 2H), 8.59–8.52 (m, 2H), 8.17 (d, *J* = 8.8 Hz, 1H), 7.88 (d, *J* = 2.8 Hz, 1H), 7.74 (dd, *J* = 8.5, 4.2 Hz, 1H), 7.26–7.22 (m, 1H), 7.17–7.11 (m, 2H), 4.92–4.81 (m, 1H), 4.06 (q, *J* = 7.0 Hz, 2H), 3.58–3.47 (m, 1H), 3.14–3.04 (m, 2H), 2.63–2.52 (m, 2H), 1.42 (t, *J* = 7.0 Hz, 3H). HRMS *m*/*z* [M + H]^+^: 493.20950 (calculated), 493.2084 (found), Δ = −2.16 ppm.

#### *N*-(*trans-*3-(5-(5-Ethoxypyridin-2-yl)-4-(pyridin-4-yl)-4*H-*1,2,4-triazol-3-yl)cyclobutyl)-7-fluoro-1,5-naphthyridine-4-carboxamide (**18b**)

The title compound was prepared according to general procedure F as a white solid (19.2 mg, 73%). LC/MS (ESI) *m*/*z* for C_27_H_23_N_8_O_2_F 510 (calculated) 511 ([M + H]^+^, found). ^1^H NMR (400 MHz, CDCl_3_) δ 10.83 (d, *J* = 6.0 Hz, 1H), 9.16 (d, *J* = 4.5 Hz, 1H), 8.91 (d, *J* = 2.9 Hz, 1H), 8.76–8.69 (m, 2H), 8.52 (d, *J* = 4.5 Hz, 1H), 8.20 (dd, *J* = 8.7, 2.9 Hz, 1H), 8.17 (d, *J* = 8.6 Hz, 1H), 7.91–7.85 (m, 1H), 7.26–7.22 (m, 1H), 7.17–7.10 (m, 2H), 4.87 (h, *J* = 7.1 Hz, 1H), 4.06 (q, *J* = 6.9 Hz, 2H), 3.56–3.45 (m, 1H), 3.14–3.03 (m, 2H), 2.62–2.51 (m, 2H), 1.42 (t, *J* = 7.0 Hz, 3H). HRMS *m*/*z* [M + H]^+^: 511.20008 (calculated), 511.1992 (found), Δ = −1.69 ppm.

#### *N*-(3-(5-(5-Ethoxypyridin-2-yl)-4-(pyridin-2-yl)-4*H-*1,2,4-triazol-3-yl)bicyclo[1.1.1]pentan-1-yl)-1,5-naphthyridine-4-carboxamide (**19**)

The title compound was prepared according to general procedure F as a white solid (12.1 mg, 47%). LC/MS (ESI) *m*/*z* for C_28_H_24_N_8_O_2_ 504 (calculated) 505 ([M + H]^+^, found). ^1^H NMR (400 MHz, CDCl_3_) δ 11.46 (s, 1H), 9.14 (d, *J* = 4.5 Hz, 1H), 9.00 (dd, *J* = 4.3, 1.7 Hz, 1H), 8.63 (dd, *J* = 5.1, 1.8 Hz, 1H), 8.55 (dd, *J* = 8.6, 1.7 Hz, 1H), 8.50 (d, *J* = 4.4 Hz, 1H), 8.15 (d, *J* = 8.8 Hz, 1H), 7.87 (td, *J* = 7.7, 1.9 Hz, 1H), 7.84 (d, *J* = 3.0 Hz, 1H), 7.75 (dd, *J* = 8.6, 4.3 Hz, 1H), 7.46 (ddd, *J* = 7.6, 4.8, 1.0 Hz, 1H), 7.34 (d, *J* = 7.9 Hz, 1H), 7.21 (dd, *J* = 8.7, 2.9 Hz, 1H), 4.03 (q, *J* = 7.0 Hz, 2H), 2.48 (s, 6H), 1.40 (t, *J* = 7.0 Hz, 3H). HRMS *m*/*z* [M + H]^+^: 505.20950 (calculated), 505.2086 (found), Δ = −1.87 ppm.

#### *N*-(3-(5-(5-Ethoxypyridin-2-yl)-4-(pyridin-3-yl)-4*H-*1,2,4-triazol-3-yl)bicyclo[1.1.1]pentan-1-yl)-1,5-naphthyridine-4-carboxamide (**20**)

The title compound was prepared according to general procedure F as a white solid (20.2 mg, 78%). LC/MS (ESI) *m*/*z* for C_28_H_24_N_8_O_2_ 504 (calculated) 505 ([M + H]^+^, found). ^1^H NMR (400 MHz, CDCl_3_) δ 11.45 (s, 1H), 9.14 (d, *J* = 4.5 Hz, 1H), 8.99 (dd, *J* = 4.1, 1.8 Hz, 1H), 8.74 (dd, *J* = 4.8, 1.5 Hz, 1H), 8.58–8.52 (m, 2H), 8.49 (d, *J* = 4.4 Hz, 1H), 8.14 (d, *J* = 8.8 Hz, 1H), 7.87 (d, *J* = 2.9 Hz, 1H), 7.74 (dd, *J* = 8.6, 4.3 Hz, 1H), 7.69 (ddd, *J* = 8.1, 2.5, 1.5 Hz, 1H), 7.46 (dd, *J* = 8.0, 4.7 Hz, 1H), 7.22 (dd, *J* = 8.8, 2.9 Hz, 1H), 4.04 (q, *J* = 6.9 Hz, 2H), 2.49 (s, 6H), 1.40 (t, *J* = 7.0 Hz, 3H). HRMS *m*/*z* [M + H]^+^: 505.20950 (calculated), 505.2085 (found), Δ = −2.01 ppm.

#### *N*-(3-(5-(5-Ethoxypyridin-2-yl)-4-(pyridin-4-yl)-4*H-*1,2,4-triazol-3-yl)bicyclo[1.1.1]pentan-1-yl)-1,5-naphthyridine-4-carboxamide (**21**)

The title compound was prepared according to general procedure F as a white solid (10.2 mg, 62%). LC/MS (ESI) *m*/*z* for C_28_H_24_N_8_O_2_ 504 (calculated) 505 ([M + H]^+^, found). ^1^H NMR (400 MHz, CDCl_3_) δ 11.45 (s, 1H), 9.14 (d, *J* = 4.5 Hz, 1H), 8.99 (dd, *J* = 4.2, 1.8 Hz, 1H), 8.82–8.75 (m, 2H), 8.55 (dd, *J* = 8.6, 1.8 Hz, 1H), 8.50 (d, *J* = 4.4 Hz, 1H), 8.14 (d, *J* = 8.8 Hz, 1H), 7.86 (d, *J* = 2.8 Hz, 1H), 7.74 (dd, *J* = 8.5, 4.2 Hz, 1H), 7.27 (d, *J* = 1.7 Hz, 2H), 7.23 (dd, *J* = 8.7, 2.9 Hz, 1H), 4.04 (q, *J* = 6.9 Hz, 2H), 2.51 (s, 6H), 1.41 (t, *J* = 7.0 Hz, 3H). HRMS *m*/*z* [M + H]^+^: 505.20950 (calculated), 505.2085 (found), Δ = −2.02 ppm.

#### *N*-(*trans-*3-(5-(5-Ethoxypyridin-2-yl)-4-(5-methylthiophen-2-yl)-4*H-*1,2,4-triazol-3-yl)cyclobutyl)-1,5-naphthyridine-4-carboxamide (**22a**)

The title compound was prepared according to general procedure F as a white solid (8.4 mg, 33%). LC/MS (ESI) *m*/*z* for C_27_H_25_N_7_O_2_S 511 (calculated) 512 ([M + H]^+^, found). ^1^H NMR (400 MHz, CDCl_3_) δ 11.31 (d, *J* = 6.0 Hz, 1H), 9.15 (d, *J* = 4.4 Hz, 1H), 9.00 (dd, *J* = 4.3, 1.8 Hz, 1H), 8.60–8.53 (m, 2H), 8.12 (d, *J* = 2.8 Hz, 1H), 7.97 (d, *J* = 8.7 Hz, 1H), 7.75 (dd, *J* = 8.5, 4.2 Hz, 1H), 7.23–7.20 (m, 1H), 6.72 (d, *J* = 3.7 Hz, 1H), 6.61 (dd, *J* = 3.7, 1.3 Hz, 1H), 4.86 (q, *J* = 7.0 Hz, 1H), 4.08 (q, *J* = 7.0 Hz, 2H), 3.72–3.62 (m, 1H), 3.16–3.05 (m, 2H), 2.69–2.58 (m, 2H), 2.47 (d, *J* = 1.1 Hz, 3H), 1.43 (t, *J* = 7.0 Hz, 3H). HRMS *m*/*z* [M + H]^+^: 512.18632 (calculated), 512.1854 (found), Δ = −1.73 ppm.

#### *N*-(*trans-*3-(5-(5-Ethoxypyridin-2-yl)-4-(5-methylthiophen-2-yl)-4*H-*1,2,4-triazol-3-yl)cyclobutyl)-7-fluoro-1,5-naphthyridine-4-carboxamide (**22b**)

The title compound was prepared according to general procedure F as a white solid (13 mg, 43%). LC/MS (ESI) *m*/*z* for C_27_H_24_N_7_O_2_SF 529 (calculated) 530 ([M + H]^+^, found). ^1^H NMR (400 MHz, CDCl_3_) δ 10.84 (d, *J* = 6.0 Hz, 1H), 9.16 (d, *J* = 4.4 Hz, 1H), 8.93 (d, *J* = 2.9 Hz, 1H), 8.54 (d, *J* = 4.4 Hz, 1H), 8.20 (dd, *J* = 8.7, 2.9 Hz, 1H), 8.12 (d, *J* = 2.9 Hz, 1H), 7.97 (d, *J* = 8.8 Hz, 1H), 7.23–7.19 (m, 1H), 6.72 (d, *J* = 3.7 Hz, 1H), 6.65–6.59 (m, 1H), 4.94–4.82 (m, 1H), 4.08 (q, *J* = 7.1 Hz, 2H), 3.72–3.60 (m, 1H), 3.15–3.06 (m, 2H), 2.66–2.57 (m, 2H), 2.47 (d, *J* = 1.1 Hz, 3H), 1.43 (t, *J* = 7.0 Hz, 3H). HRMS *m*/*z* [M + H]^+^: 530.17690 (calculated), 530.1761 (found), Δ = −1.59 ppm.

#### *N*-(*trans-*3-(5-(5-Ethoxypyridin-2-yl)-4-(5-methylthiophen-2-yl)-4*H-*1,2,4-triazol-3-yl)cyclobutyl)quinoline-8-carboxamide (**23**)

The title compound was prepared according to general procedure F as a white solid (12 mg, 41%). LC/MS (ESI) *m*/*z* for C_28_H_26_N_6_O_2_S 510 (calculated) 511 ([M + H]^+^, found). ^1^H NMR (400 MHz, CDCl_3_) δ 11.57 (d, *J* = 5.9 Hz, 1H), 8.94 (dd, *J* = 4.2, 1.9 Hz, 1H), 8.85 (dd, *J* = 7.4, 1.6 Hz, 1H), 8.29 (dd, *J* = 8.3, 1.8 Hz, 1H), 8.12 (d, *J* = 2.8 Hz, 1H), 7.96 (dd, *J* = 8.3, 1.6 Hz, 2H), 7.68 (t, *J* = 7.7 Hz, 1H), 7.53–7.47 (m, 1H), 7.22–7.19 (m, 1H), 6.72 (d, *J* = 3.7 Hz, 1H), 6.63–6.58 (m, 1H), 4.83 (q, *J* = 7.0 Hz, 1H), 4.08 (q, *J* = 7.0 Hz, 2H), 3.77–3.60 (m, 1H), 3.12–3.06 (m, 2H), 2.67–2.60 (m, 2H), 2.46 (d, *J* = 1.2 Hz, 3H), 1.43 (t, *J* = 6.9 Hz, 3H). HRMS *m*/*z* [M + H]^+^: 511.19107 (calculated), 511.1902 (found), Δ = −1.63 ppm.

#### *N*-(*trans-*3-(5-(5-Ethoxypyridin-2-yl)-4-(2-fluorophenyl)-4*H-*1,2,4-triazol-3-yl)cyclobutyl)quinoxaline-5-carboxamide (**24**, OM-153)

The title compound was prepared according to general procedure F as a white solid (20.4 mg, 79%). LC/MS (ESI) *m*/*z* for C_28_H_24_N_7_O_2_F 509 (calculated) 510 ([M + H]^+^, found). ^1^H NMR (400 MHz, CDCl_3_) δ 10.68 (d, *J* = 5.9 Hz, 1H), 8.97 (d, *J* = 1.9 Hz, 1H), 8.89–8.83 (m, 2H), 8.26 (dd, *J* = 8.3, 1.6 Hz, 1H), 8.16 (d, *J* = 8.7 Hz, 1H), 7.94–7.86 (m, 2H), 7.47–7.39 (m, 1H), 7.25–7.13 (m, 4H), 4.83 (dqd, *J* = 8.5, 6.8, 5.3 Hz, 1H), 4.04 (q, *J* = 7.0 Hz, 2H), 3.53 (dp, *J* = 10.1, 5.5 Hz, 1H), 3.08 (dtd, *J* = 12.0, 5.8, 3.9 Hz, 2H), 2.53 (ddtt, *J* = 19.4, 9.6, 6.2, 3.0 Hz, 2H), 1.40 (t, *J* = 7.0 Hz, 3H). HRMS *m*/*z* [M + H]^+^: 510.20483 (calculated), 510.2040 (found), Δ = −1.66 ppm.

#### *N*-(*trans-*3-(5-(5-Ethoxypyridin-2-yl)-4-(2-fluorophenyl)-4*H-*1,2,4-triazol-3-yl)cyclobutyl)-7-fluoroquinoxaline-5-carboxamide (**25**)

The title compound was prepared according to general procedure F as a white solid (18.2 mg, 68%). LC/MS (ESI) *m*/*z* for C_28_H_23_N_7_O_2_F 527 (calculated) 528 ([M + H]^+^, found). ^1^H NMR (400 MHz, CDCl_3_) δ 10.62 (d, *J* = 5.8 Hz, 1H), 8.96 (d, *J* = 1.8 Hz, 1H), 8.82 (d, *J* = 1.9 Hz, 1H), 8.64 (dd, *J* = 9.6, 3.1 Hz, 1H), 8.24–8.11 (m, 1H), 7.87 (dd, *J* = 7.9, 3.1 Hz, 2H), 7.48–7.39 (m, 1H), 7.25–7.14 (m, 4H), 4.83 (h, *J* = 6.8 Hz, 1H), 4.04 (q, *J* = 6.9 Hz, 2H), 3.57–3.46 (m, 1H), 3.13–3.01 (m, 2H), 2.60–2.45 (m, 2H), 1.40 (t, *J* = 6.9 Hz, 3H). HRMS *m*/*z* [M + H]^+^: 528.19541 (calculated), 528.1946 (found), Δ = −1.59 ppm.

#### *N*-(*trans-*3-(5-(5-Ethoxypyridin-2-yl)-4-(2-fluorophenyl)-4*H-*1,2,4-triazol-3-yl)cyclobutyl)-3-methylquinoxaline-5-carboxamide (**26**)

The title compound was prepared according to general procedure F as a slightly pink solid (93.1 mg, 65%). LC/MS (ESI) *m*/*z* for C_29_H_26_N_7_O_2_F 523 (calculated) 524 ([M + H]^+^, found). ^1^H NMR (400 MHz, DMSO) δ 10.42 (d, *J* = 7.1 Hz, 1H), 8.98 (s, 1H), 8.41 (dd, *J* = 7.3, 1.5 Hz, 1H), 8.21 (dd, *J* = 8.3, 1.5 Hz, 1H), 8.06 (d, *J* = 8.7 Hz, 1H), 7.94 (d, *J* = 3.0 Hz, 1H), 7.87 (dd, *J* = 8.3, 7.3 Hz, 1H), 7.61–7.53 (m, 2H), 7.50 (dd, *J* = 8.8, 3.0 Hz, 1H), 7.47–7.39 (m, 1H), 7.36–7.29 (m, 1H), 4.75 (q, *J* = 7.3 Hz, 1H), 4.10 (q, *J* = 7.0 Hz, 2H), 3.40 (dt, *J* = 9.4, 4.6 Hz, 1H), 2.92–2.82 (m, 1H), 2.75 (s, 3H), 2.72–2.66 (m, 1H), 2.45–2.29 (m, 2H), 1.31 (t, *J* = 6.9 Hz, 3H). HRMS *m*/*z* [M + H]^+^: 524.22048 (calculated), 524.2197 (found), Δ = −1.56 ppm.

#### *N*-(*trans-*3-(5-(5-Ethoxypyridin-2-yl)-4-(2-fluorophenyl)-4*H-*1,2,4-triazol-3-yl)cyclobutyl)-2-methylquinoxaline-5-carboxamide (**27**)

The title compound was prepared according to general procedure F as an off-white solid (20.1 mg, 71%). LC/MS (ESI) *m*/*z* for C_29_H_26_N_7_O_2_F 523 (calculated) 524 ([M + H]^+^, found). ^1^H NMR (400 MHz, DMSO) δ 10.42 (d, *J* = 7.1 Hz, 1H), 8.98 (s, 1H), 8.41 (dd, *J* = 7.3, 1.6 Hz, 1H), 8.21 (dd, *J* = 8.4, 1.5 Hz, 1H), 8.06 (d, *J* = 8.8 Hz, 1H), 7.93 (d, *J* = 2.8 Hz, 1H), 7.91–7.84 (m, 1H), 7.63–7.52 (m, 2H), 7.50 (dd, *J* = 8.8, 3.0 Hz, 1H), 7.47–7.39 (m, 1H), 7.36–7.29 (m, 1H), 4.75 (q, *J* = 7.2 Hz, 1H), 4.10 (q, *J* = 7.0 Hz, 2H), 3.44–3.36 (m, 1H), 2.87 (q, *J* = 3.8 Hz, 1H), 2.75 (s, 3H), 2.68 (s, 1H), 2.45–2.28 (m, 2H), 1.31 (t, *J* = 6.9 Hz, 3H). HRMS *m*/*z* [M + H]^+^: 524.22048 (calculated), 524.2198 (found), Δ = −1.36 ppm.

#### *N*-(*trans-*3-(5-(5-Ethoxypyridin-2-yl)-4-(2-fluorophenyl)-4*H-*1,2,4-triazol-3-yl)cyclobutyl)-2,3-dimethylquinoxaline-5-carboxamide (**28**)

The title compound was prepared according to general procedure F as an off-white solid (29.7 mg, 43%). LC/MS (ESI) *m*/*z* for C_30_H_28_N_7_O_2_F 537 (calculated) 538 ([M + H]^+^, found). ^1^H NMR (400 MHz, DMSO) δ 10.59 (d, *J* = 7.2 Hz, 1H), 8.36 (dd, *J* = 7.4, 1.6 Hz, 1H), 8.18–8.01 (m, 2H), 7.94 (d, *J* = 3.0 Hz, 1H), 7.82 (t, *J* = 7.8 Hz, 1H), 7.56 (td, *J* = 8.2, 3.1 Hz, 2H), 7.50 (dd, *J* = 8.8, 2.9 Hz, 1H), 7.43 (t, *J* = 9.2 Hz, 1H), 7.33 (t, *J* = 7.7 Hz, 1H), 4.75 (q, *J* = 7.2 Hz, 1H), 4.10 (q, *J* = 7.0 Hz, 2H), 2.86 (d, *J* = 9.7 Hz, 1H), 2.72 (d, *J* = 3.6 Hz, 7H), 2.45–2.28 (m, 2H), 1.31 (t, *J* = 7.0 Hz, 3H). HRMS *m*/*z* [M + H]^+^: 538.23613 (calculated), 538.2349 (found), Δ = −2.27 ppm.

#### *N*-(*trans-*3-(5-(5-(Difluoromethoxy)pyridin-2-yl)-4-(2-fluorophenyl)-4*H-*1,2,4-triazol-3-yl)cyclobutyl)quinoxaline-5-carboxamide (**29a**)

The title compound was prepared according to general procedure F as a white solid (23 mg, 86%). LC/MS (ESI) *m*/*z* for C_27_H_20_N_7_O_2_F_3_ 531 (calculated) 532 ([M + H]^+^, found). ^1^H NMR (400 MHz, CDCl_3_) δ 10.67 (d, *J* = 5.9 Hz, 1H), 8.97 (d, *J* = 1.8 Hz, 1H), 8.89–8.83 (m, 2H), 8.33 (d, *J* = 8.8 Hz, 1H), 8.26 (dd, *J* = 8.4, 1.6 Hz, 1H), 8.07 (d, *J* = 2.8 Hz, 1H), 7.90 (dd, *J* = 8.4, 7.4 Hz, 1H), 7.55 (dd, *J* = 8.8, 2.8 Hz, 1H), 7.51–7.42 (m, 1H), 7.26–7.16 (m, 3H), 6.52 (t, *J* = 72.5 Hz, 1H), 4.90–4.79 (m, 1H), 3.57–3.47 (m, 1H), 3.14–3.02 (m, 2H), 2.63–2.47 (m, 2H). HRMS *m*/*z* [M + H]^+^: 532.17033 (calculated), 532.1692 (found), Δ = −2.12 ppm.

#### *N*-(*trans*-3-(5-(5-(2,2-Difluoroethoxy)pyridin-2-yl)-4-((*S*)-2-fluorophenyl)-4*H*-1,2,4-triazol-3-yl)cyclobutyl)-7-fluoroquinoxaline-5-carboxamide (**29b**)

The title compound was prepared according to general procedure F as a white solid (19.8 mg, 71%). LC/MS (ESI) *m*/*z* for C_27_H_19_N_7_O_2_F_4_ 549 (calculated) 550 ([M + H]^+^, found). ^1^H NMR (400 MHz, CDCl_3_) δ 10.63 (d, *J* = 5.9 Hz, 1H), 8.96 (d, *J* = 2.0 Hz, 1H), 8.82 (d, *J* = 1.9 Hz, 1H), 8.64 (dd, *J* = 9.5, 3.1 Hz, 1H), 8.33 (d, *J* = 8.7 Hz, 1H), 8.07 (d, *J* = 2.6 Hz, 1H), 7.87 (dd, *J* = 7.8, 3.1 Hz, 1H), 7.55 (dd, *J* = 8.8, 2.8 Hz, 1H), 7.51–7.42 (m, 1H), 7.25–7.16 (m, 3H), 6.52 (t, *J* = 72.5 Hz, 1H), 4.91–4.79 (m, 1H), 3.56–3.45 (m, 1H), 3.14–3.01 (m, 2H), 2.62–2.47 (m, 2H). HRMS *m*/*z* [M + H]^+^: 550.16091 (calculated), 550.1597 (found), Δ = −2.25 ppm.

#### *N*-(*trans-*3-(4-(2-Fluorophenyl)-5-(pyridin-2-yl)-4*H-*1,2,4-triazol-3-yl)cyclobutyl)quinoxaline-5-carboxamide (**30a**)

The title compound was prepared according to general procedure F as a white solid (19.3 mg, 83%). LC/MS (ESI) *m*/*z* for C_26_H_20_N_7_OG 465 (calculated) 466 ([M + H]^+^, found). ^1^H NMR (400 MHz, CDCl_3_) δ 10.68 (d, *J* = 5.8 Hz, 1H), 8.97 (d, *J* = 1.8 Hz, 1H), 8.89–8.83 (m, 2H), 8.26 (dd, *J* = 8.3, 1.5 Hz, 2H), 8.24–8.18 (m, 1H), 7.94–7.87 (m, 1H), 7.76 (td, *J* = 7.8, 1.7 Hz, 1H), 7.49–7.40 (m, 1H), 7.25–7.14 (m, 4H), 4.90–4.78 (m, 1H), 3.59–3.49 (m, 1H), 3.15–3.03 (m, 2H), 2.62–2.47 (m, 2H). HRMS *m*/*z* [M + H]^+^: 466.17861 (calculated), 466.1775 (found), Δ = −2.34 ppm.

#### *N*-(*trans*-3-(5-(5-(6-Methylpyridin-2-yl)-4-(*S*)-2-fluorophenyl)-4*H*-1,2,4-triazol-3-yl)cyclobutyl)-7-fluoroquinoxaline-5-carboxamide (**30b**)

The title compound was prepared according to general procedure F as a white solid (16 mg, 65%). LC/MS (ESI) *m*/*z* for C_26_H_19_N_7_OF_2_ 483 (calculated) 484 ([M + H]^+^, found). ^1^H NMR (400 MHz, CDCl_3_) δ 10.63 (d, *J* = 5.8 Hz, 1H), 8.96 (d, *J* = 1.8 Hz, 1H), 8.83 (d, *J* = 1.8 Hz, 1H), 8.64 (dd, *J* = 9.6, 3.1 Hz, 1H), 8.26 (dt, *J* = 8.1, 1.1 Hz, 1H), 8.22 (dd, *J* = 4.8, 1.6 Hz, 1H), 7.87 (dd, *J* = 7.8, 3.1 Hz, 1H), 7.76 (td, *J* = 7.8, 1.8 Hz, 1H), 7.49–7.40 (m, 1H), 7.25–7.14 (m, 4H), 4.84 (ht, *J* = 7.1, 1.5 Hz, 1H), 3.53 (tt, *J* = 9.3, 5.3 Hz, 1H), 3.14–3.02 (sym. m, 2H), 2.55 (dddd, *J* = 16.5, 13.1, 9.8, 6.5 Hz, 2H). HRMS *m*/*z* [M + H]^+^: 484.16919 (calculated), 484.1684 (found), Δ = −1.65 ppm.

#### *N*-(*trans-*3-(4-(2-Fluorophenyl)-5-(6-methylpyridin-2-yl)-4*H-*1,2,4-triazol-3-yl)cyclobutyl)quinoxaline-5-carboxamide (**31a**)

The title compound was prepared according to general procedure F as a white solid (20.7 mg, 86%). LC/MS (ESI) *m*/*z* for C_27_H_22_N_7_OF 479 (calculated) 480 ([M + H]^+^, found). ^1^H NMR (400 MHz, CDCl_3_) δ 10.68 (d, *J* = 5.7 Hz, 1H), 8.96 (d, *J* = 1.9 Hz, 1H), 8.90–8.82 (m, 2H), 8.26 (dd, *J* = 8.4, 1.6 Hz, 1H), 8.07 (d, *J* = 7.8 Hz, 1H), 7.90 (dd, *J* = 8.4, 7.4 Hz, 1H), 7.63 (t, *J* = 7.8 Hz, 1H), 7.49–7.41 (m, 1H), 7.26–7.14 (m, 3H), 7.03 (d, *J* = 7.7 Hz, 1H), 4.89–4.78 (m, 1H), 3.61–3.50 (m, 1H), 3.15–3.05 (m, 2H), 2.62–2.48 (m, 2H), 2.07 (s, 3H). HRMS *m*/*z* [M + H]^+^: 480.19426 (calculated), 480.1934 (found), Δ = −1.75 ppm.

#### *N*-(*trans-*3-(4-(2-Fluorophenyl)-5-(6-methylpyridin-2-yl)-4*H-*1,2,4-triazol-3-yl)cyclobutyl)-7-fluoroquinoxaline-5-carboxamide (**31b**)

The title compound was prepared according to general procedure F as a white solid (18.2 mg, 72%). LC/MS (ESI) *m*/*z* for C_27_H_21_N_7_OF_2_ 497 (calculated) 498 ([M + H]^+^, found). ^1^H NMR (400 MHz, CDCl_3_) δ 10.63 (d, *J* = 5.9 Hz, 1H), 8.96 (d, *J* = 1.9 Hz, 1H), 8.83 (d, *J* = 1.8 Hz, 1H), 8.64 (dd, *J* = 9.5, 3.1 Hz, 1H), 8.06 (d, *J* = 7.8 Hz, 1H), 7.87 (dd, *J* = 7.8, 3.1 Hz, 1H), 7.63 (t, *J* = 7.8 Hz, 1H), 7.49–7.41 (m, 1H), 7.24–7.15 (m, 3H), 7.03 (d, *J* = 7.7 Hz, 1H), 4.91–4.78 (m, 1H), 3.60–3.49 (m, 1H), 3.15–3.03 (m, 2H), 2.62–2.48 (m, 2H), 2.07 (s, 3H). HRMS *m*/*z* [M + H]^+^: 498.18484 (calculated), 498.1838 (found), Δ = −2.11 ppm.

### Biochemical Assay

Recombinantly expressed human tankyrase active constructs for TNKS1 (residues 1030–1317) and TNKS2 (residues 873–1162) and other PARP enzymes used for biochemical assays were produced as previously described.^39^ The enzymatic assay measures unreacted NAD^+^, which is chemically reacted into a fluorescent compound.^53^ The fluorescence intensity was measured with excitation/emission wavelengths of 372 and 444 nm, respectively, using Tecan Infinity M1000 Pro. New compounds were prepared in half-log dilution series, and the reactions were carried out in quadruplicates with protein and compound controls to exclude the effect of compound autofluorescence. All reactions were performed at ambient temperature. TNKS1 (20 nM) or TNKS2 (5 nM) was incubated for 20 h in assay buffer (50 mM Bis-Tris propane (BTP), pH 7.0, 0.5 mM Tris(2-carboxyethyl)phosphine (TCEP), 0.01% Triton X-100) with compound and 10 μM or 500 nM NAD^+^, respectively.

The assay conditions for the other PARP enzymes were used as previously described.^39^ Reference compound OD336 behaved in this assay as previously reported.^28^

For single IC_50_ curves, the 95% confidence interval (asymptotic) was calculated in GraphPad Prism 8.

### WNT/β-Catenin Signaling Reporter Assay

The luciferase based WNT/β-catenin signaling pathway reporter assay in human HEK293 cells was performed in triplicates as previously described^21^ also taking OD336 as a reference compound, which behaved in this assay as previously described.^28^

### ADME

Kinetic solubility, microsomal stability, and plasma stability were determined following our internal protocol (Symeres). The Caco-2 and PPB determinations were performed by Cyprotex.

### Off-Target

A safety panel (Cerep) of 44 selected targets (*n* = 2) including hERG Inhibition using 10 μM **24** was performed by Eurofins. Ames and CYP3A4 inhibition assays were performed by Cyprotex, and the PXR assay (CYP induction) was performed at Admescope. The PARP assays are described below.

### Mouse Pharmacokinetic Analysis

The pharmacokinetic analyses in mice were performed according to the standard protocols of Medicilon as previously described^21^ following approval by local animal experiment authorities (Shanghai, China) and in compliance with FELASA guidelines and recommendations. Three IRC mice were used per treatment group: 5 mg/kg **24** in 5% DMSO, 50% PEG400 (both Sigma-Aldrich), and 45% saline as vehicle. Samples were collected after 5, 15, and 30 min and 1, 2, 4, 8, and 24 h.

### Crystallography

The co-crystallization of human TNKS2 catalytic domain (residues 952–1161) in complex with **24** was done in the presence of chymotrypsin (1:100) and based on crystallization efforts previously described.^39^ Protein (5.3 mg/mL) was mixed with 0.4 mM compound from a 10 mM stock solution in DMSO. The droplets for crystallization were set up using the sitting-drop vapor diffusion method by mixing 200 nL of protein with 100 nL of precipitant solution (0.1 M Bicine, pH 8.5–9.0, 7.5–25% PEG6000). All steps were performed at room temperature. Rod-shaped crystals appeared within 24 h and were cryoprotected using the precipitant solution containing 25% PEG6000 and 20% glycerol. Data were collected at Diamond Light Source on beamline I04. Diffraction data were processed using the XDS package.^54^ The substructure was solved using molecular replacement with PHASER^55^ using the structure of TNKS2 (PDB code: 5NOB) as starting model. Model building and refinement was done using Coot^56^ and Refmac5,^57^ respectively. Preparation of the crystal structure images was done with The PyMOL Molecular Graphics System (PyMOL, version 1.8.4.0).

### Proliferation Assay

Antiproliferative assays using colon cancer cell lines COLO 320DM and RKO were performed as previously described.^39^

### Western Blot Analysis

Western blot analysis of nuclear and cytoplasmic lysates from compound-treated COLO 320DM cells was performed as previously described.^39,50^

### RNA Isolation and Real-Time qRT-PCR

RNA isolation and real-time qRT-PCR were performed as previously described.^39,50^

## Supporting Information Available

Molecular formula strings and data (CSV)
General and specific synthetic procedures and spectra for all compounds and inhibition data and crystallography; atomic coordinates and structure factors have been deposited to the Protein Data Bank under accession number 7O6X, and raw diffraction images are available at IDA (https://doi.org/10.23729/4d448eb0-c6ef-4b7d-a557-3758f5f68d47). The authors will release the atomic coordinates upon article publication (PDF)

## Abbreviations Used

ADME: absorption, distribution, metabolism, and excretion
ADP: adenosine 5′-diphosphate
AKT: serine/threonine kinase
AMOT: angiomotin
AMP: adenosine monophosphate
AMPK: AMP-activated protein kinase
APC: adenomatous polyposis coli
APCDD1: APC downregulated 1
AUC: area under the curve
AXIN: axis inhibition protein
CL: clearance
CMC: chemistry, manufacturing, and control
DKK1: dickkopf WNT signaling pathway inhibitor 1
DMSO: dimethyl sulfoxide
HEK293: human embryonic kidney 293
hERG: human ether-à-go-go-related gene
LC/MS: liquid chromatography/mass spectroscopy
MTS: 3-(4,5-dimethylthiazol-2-yl)-5-(3-carboxymethoxyphenyl)-2-(4-sulfophenyl)-2*H*-tetrazolium
NKD1: NKD inhibitor of WNT signaling pathway 1
NAD: nicotinamide adenine dinucleotide
NMR: nuclear magnetic resonance
PARP: poly-ADP-ribose polymerase
PARsylate: poly(ADP-ribose)sylate
PGC-1α: peroxisome proliferator-activated receptor-γ coactivator 1 α
PK: pharmacokinetics
PO: per oral
PPB: plasma protein binding
PTEN: phosphatase and tensin homolog
RT-qPCR: reverse transcription quantitative polymerase chain reaction
RNF146: ring finger protein 146
SD: standard deviation
SEM: standard error of the mean
SH3BP2: sarcoma homology 3 (SH3) domain binding protein 2
SOX9: sex-determining region Y (SRY)-box transcription factor 9
*t*_1/2_: half-life
TNKS: telomeric repeat factor (TRF1)-interacting ankyrin-related ADP-ribose polymerases, tankyrase
TNKS1/2: tankyrase 1 and tankyrase 2
tPSA: total polar surface area
TRF: telomeric repeat factor
*V*_d_: volume of distribution
WNT: wingless-type mammary tumor virus integration site
