# Supplementary material for: Development of a 1,2,4-Triazole-Based Lead Tankyrase Inhibitor: Part II
Source: J Med Chem. 2021 Dec 8;64(24):17936–49. doi: 10.1021/acs.jmedchem.1c01264 (PMC8713164; doi:10.1021/acs.jmedchem.1c01264)
Supplement: Supplementary file 2 — jm1c01264_si_002.pdf [file jm1c01264_si_002.pdf]

Supplementary Information

Development of a 1,2,4-Triazole Based Lead Tankyrase Inhibitor – Part-II

Ruben G. G. Leenders,[§,#] Shoshy Alam Brinch,[†,‡,#] Sven T. Sowa,[ǀ] Enya Amundsen-Isaksen,[†,‡] Albert Galera-Prat,[ǀ] Sudarshan Murthy,[ǀ] Sjoerd Aertssen,[§] Johannes N. Smits,[§] Piotr Nieczypor,[§] Eddy Damen,[§] Anita Wegert,[§] Marc Nazaré,[ǀǀ] Lari Lehtiö,[ǀ] Jo Waaler,[†,‡,#] and Stefan Krauss[†,‡,#,*]

[§] Symeres, Kerkenbos 1013, 6546 BB Nijmegen, the Netherlands

[†] Hybrid Technology Hub - Centre of Excellence, Institute of Basic Medical Sciences, University of Oslo, 0317 Oslo, Norway

[‡] Department of Immunology and Transfusion Medicine, Oslo University Hospital, 0424 Oslo, Norway

[ǀ] Faculty of Biochemistry and Molecular Medicine, Biocenter Oulu, University of Oulu, 90014 Oulu, Finland

[ǀǀ] Medicinal Chemistry, Leibniz-Forschungsinstitut für Molekulare Pharmakologie (FMP), Campus Berlin Buch, Robert-Roessle-Str. 10, 13125, Berlin, Germany

* Corresponding author; phone: +47 97610063; Email: stefan.krauss@medisin.uio.no.

# R.G.G.L. and S.A.B. contributed equally to this article while J.W. and S.K. contributed equally as co-senior authors of this article.

Contents

Page S2	Synthetic Organic Procedures
Page S17	Spectra of final products
Page S81	Figure S-1: Dose response curves of compound **24** (OM-153) for TNKS1 and -2
Page S82	Table S-1: Inhibition of PARPs
Page S82	Chart S-1: Selected compounds from our previous paper[1]
Page S83	Table S-2: Data collection and refinement statistics for the cocrystal structure of TNKS2 in complex with compound **24** (OM-153)

Synthetic Organic Procedures

General Methods

All starting materials and dry solvents were commercially obtained. Reactions were performed under an inert atmosphere of nitrogen when necessary. Microwave reactions were carried out in sealed vials. Column chromatography was carried out on silica gel cartridges (40 µm irregular), and TLC analysis was performed on silica gel 60 F254 plates.

NMR. NMR spectra were recorded in chloroform-d, unless otherwise stated, on a 400 MHz spectrometer with tetramethylsilane as internal standards. Coupling constants are given in Hz.

LC/MS. LC/MS chromatograms mass spectra were recorded using electrospray ionization in positive or negative ionization mode on Agilent 1260 Bin: pump, G1312B, degasser; autosampler; ColCom; DAD G1315C; MSD G6130B ESI; eluent A, acetonitrile; eluent B, 10 mM ammonium bicarbonate in water (base mode) or 0.1% formic acid in water (acid mode).

HRMS. HRMS spectra were recorded with a LC-MS Q Exactive Focus high resolution mass spectrometer (Thermo Scientific). Calibration: With the Pierce calibration solutions containing 1-butylamine, caffeine, MRFA and Ultramark 1621 (positive mode) and the Pierce calibration solution containing sodium dodecyl sulfate, sodium taurocholate, and Ultramark 1621 (negative mode). Analysis: 1 µl of a 10 µg/ml sample in MeCN/DMSO 99/1 is injected and data is acquired under full MS mode (resolution 70000 FWHM at 200 Da) over the mass range m/z of 150 – 2000. Standard ESI conditions compatible with the flow rate are applied: spray voltage 3.5 kV, auxiliary gas heater temperature 463°C, capillary temperature 280°C, sheath gas 58, auxiliary gas 16, sweep gas 3, S-lens RF level 50. Mass scan range is 150 – 2000 m/z. Mass resolution is set at 70000 (< 3 ppm mass accuracy). Data are evaluated using Xcalibur Qual Browser version 4.2.47 (Thermo Fisher).

GC/MS. Agilent 6890N, injection S/SL; injector 7683. MS: 5973 MS, EI-positive; carrier gas He.

Analytical SFC. Waters UPC2, Bin pump ACQ-ccBSM; autosampler, column manager; PDA ACQ-PDA; QDA and isocratic pump ACQ-ISM. Phenomenex Amylose-1 (100 mm × 4.6 mm, 5 µm); column temp 35 °C; flow 2.5 mL/min; BPR 170 bar; eluent A, CO_2; eluent B, MeOH + 20 mM ammonia. Linear gradient: t = 0 min 5% B, t = 5 min 50% B; t = 6 min 50% B. Detection: PDA (210–320 nm).

Preparative SFC. Waters Prep 100 SFC UV/MS directed system; Waters 2998 photodiode array (PDA) detector; Waters Acquity QDa MS detector; Waters 2767 sample manager. Columns: Phenomenex Lux Amylose-1 (250 mm × 21 mm, 5 µm), Phenomenex Lux Cellulose-1 (250 mm × 21.2 mm, 5 µm), Phenomenex Lux Cellulose- 2 (250 mm × 21.2 mm, 5 µm), Diacel Chiralpak IC for SFC (250 mm × 20 mm, 5 µm); column temp 35 °C; flow 70 mL/min; ABPR 120 bar; eluent A, CO_2; eluent B, 20 mM ammonia in methanol. Linear gradient: t = 0 min 10% B, t = 5 min 50% B; t = 7.5 min 50% B. Detection: PDA (210–400 nm). Fraction collection is based on PDA TIC.

MPLC. Base: Preparative base XSelect. Instrument type: Reveleris prep MPLC; column, Waters XSelect CSH C18 (145 mm × 25 mm, 10 µm); flow 40 mL/min; column temp, room temperature; eluent A, 10 mM ammonium bicarbonate in water (pH = 9.0); eluent B, 99% acetonitrile + 1% 10 mM ammonium bicarbonate in water. Gradient: t = 0 min 5% B, t = 1 min 5% B, t = 2 min 30% B, t = 17 min 70% B, t = 18 min 100% B, t = 23 min 100% B. Detection UV: 220, 254, 340 nm. Acid: Preparative acid Luna. Instrument type: Reveleris prep MPLC; column, Phenomenex Luna C18(3) (150 mm × 25 mm, 10 µm); flow 40 mL/min; column temp, room temperature; eluent A, 0.1% (v/v) formic acid in water; eluent B, 0.1% (v/v) formic acid in acetonitrile. Gradient: t = 0 min 5% B, t = 1 min 5% B, t = 2 min 30% B, t = 17 min 70% B, t = 18 min 100% B, t = 23 min 100% B. Detection UV: 220, 254, 340 nm, ELSD.

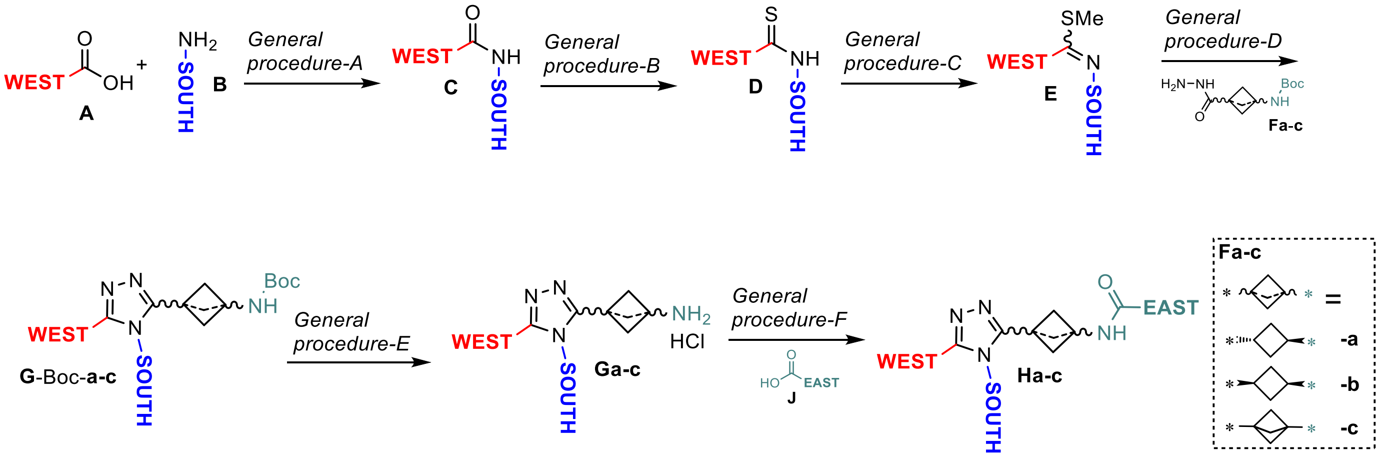

Scheme S-1. General scheme of synthesis.

General synthetic procedures

General Procedure A: Amide synthesis

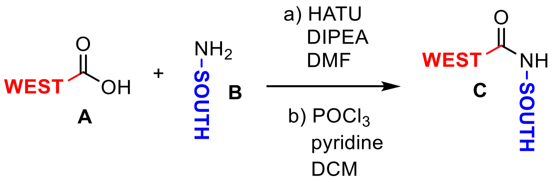

Scheme S-2. Amide synthesis.

Method a): To a solution of an appropriate acid **A** (1.0 equiv.) and DIPEA (1.2 equiv.) in dried DMF (0.2–0.5 M) was added HATU (1.1 equiv.) under an inert atmosphere. The reaction was stirred for 1 hour before the suitable amine **B** (1.1 equiv.) was added. The stirring was continued for 2 to 24 hours and then evaporated to dryness. The residue was either first extracted (treated with a diluted aqueous sodium bicarbonate and DCM) or directly purified by flash column chromatography on silica gel (gradient of ethyl acetate in heptane, usually 10% to 100%) to afford the target amides **C**.

Method b): An equimolar mixture of the starting acid and amine were dissolved in a 5:1 mixture of DCM and pyridine (reaction molarity 0.2-0.5 M), the solution was cooled in an ice-bath and treated by a dropwise addition of 1.0-1.1 equiv. of phosphorous oxychloride. The cooling bath was removed and the mixture was stirred at ambient temperature for 1 to 18 hours. After an acidic extractive work-up, drying and chromatography, the desired amides were obtained.

General Procedure B: Thioamide synthesis

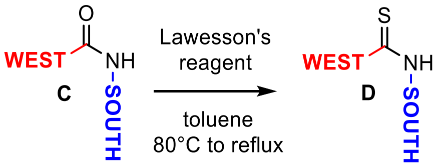

Scheme S-3. Thioamide synthesis.

The amide **C** (1.0 equiv.) was suspended under a nitrogen atmosphere in anhydrous toluene (0.10 to 0.25 M). Lawesson's reagent (1 equiv.) was added and the mixture was heated at temperature between 80 °C and reflux during 2 to 24 hours. After the reaction mixture was concentrated, the residue was extracted with DCM from aqueous phase or directly purified by flash column chromatography on silica gel (usually a gradient of ethyl acetate in heptane was used, sometimes a gradient of DCM in heptane) to afford a batch of desired thioamide **D**.

General Procedure C: Methylation of thioamide

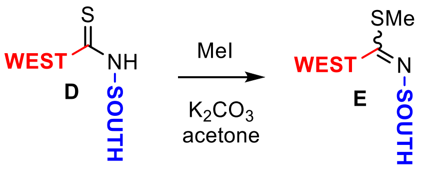

Scheme S-4. Methylation of thioamide.

To a solution of thioamide **D** (1.0 equiv.) and iodomethane (1.1-1.3 equiv.) in acetone (0.10 to 0.30 M) was added potassium carbonate (1.3–1.5 equiv.). The suspension was stirred at room temperature until the reaction completion (from 2 hours up to overnight). After solvent evaporation, the reaction mixture was either extracted from aqueous solution with DCM affording the crude product **E** (as a mixture of E/Z isomers) that could be used without any purification in the next step. For better results in the next step, the crude product **E** might be flashed over silica gel column eluted with a gradient of ethyl acetate (5% to 30%) in heptane.

General Procedure D: Triazole cyclisation

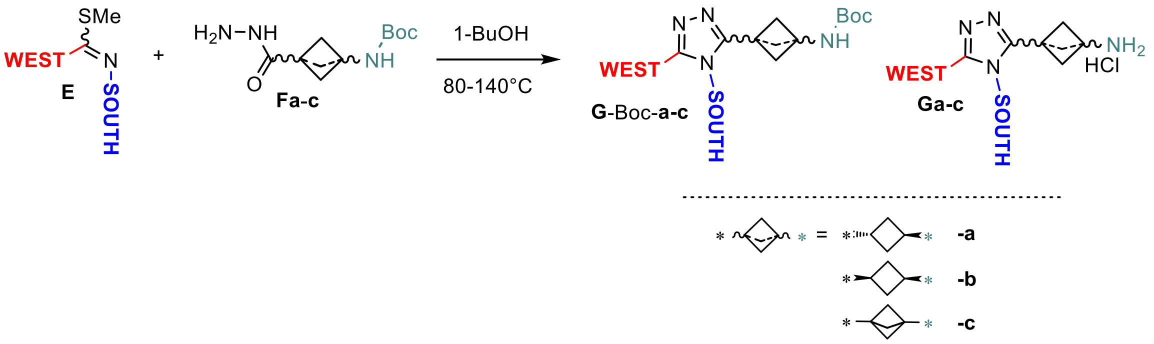

Scheme S-5. Triazole cyclisation.

A suspension of carbimidothioate **E** (1.0-1.1 equiv.) and an appropriate hydrazide **Fa-c** [1] (1.0-1.1 equiv.) in 1-butanol (0.10 to 0.30 M) was placed into a microwave vial and closed with a cap. The mixture was irradiated (or heated in an oil bath) at a temperature ranging from 80 to 140 °C until the completion of the reaction (typically 5 to 20 hours). After evaporating to dryness, the residue was purified by flash column chromatography on silica gel (gradient of ethyl

acetate in heptane as eluent) to afford 1,2,4-triazole derivatives Boc protected **G**-Boc-**a** to **G**-Boc-**c** or **Ga** to **Gc**, depending on the reaction temperature.

General Procedure E: Boc removal

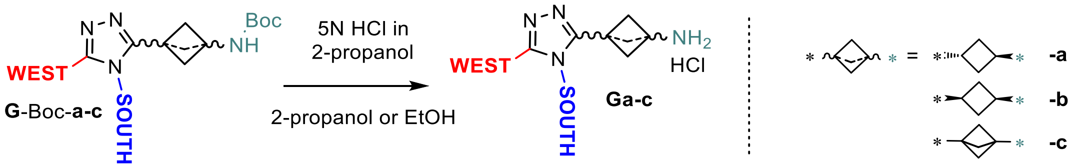

Scheme S-6. Boc removal.

To a solution or suspension of the Boc-protected 1,2,4-triazole derivate **G**-Boc (1.0 equiv.) in absolute ethanol or 2-propanol (0.05 to 0.25 M) was added hydrogen chloride as a 5 N solution in 2-propanol (10-40 equiv.). The reaction was stirred at ambient or slightly elevated (50-60 °C) temperature during 2 to 18 hours. After reaction completion (if needed, extra portions of HCl solution were added) the solvents were removed *in vacuo*, sometimes stripping the residue with acetonitrile. The crude salt **G**, or a dihydrochloride depending on the actual South and West moieties. This was used as such in the final step.

General Procedure F: Amide coupling towards the final product

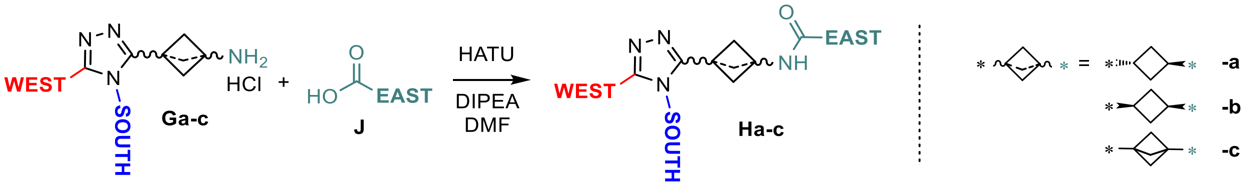

Scheme S-7. Amide coupling towards the final product.

To a suspension or solution of the appropriate acid **J** (1.1 equiv.) and HATU (1.2 equiv.) in anhydrous acetonitrile or DMF (0.02 to 0.10 M) was added DIPEA (4.0 equiv.) and the mixture was stirred from 30 to 60 minutes, preferably under an inert atmosphere before amine **G** (1.0 equiv.) was added. The coupling was complete mostly within 1-3 hours, when the mixture was concentrated to dryness. The residue was submitted to purification by preparative SFC or by flash silica gel chromatography (0% via 3-5% to 10% gradient of methanol in DCM) followed by basic mode reversed-phase column (PoraPak Rxn RP, gradient acetonitrile in 10 mM aqueous ammonium bicarbonate). Final compounds **H** were obtained mostly as a white powder after lyophilization from acetonitrile / water.

Preparation of intermediates

Final products **1** – **6**, **15a** and **15b** and intermediate for compounds **24** – **28** have been described earlier by us.[1]

Preparation of *trans*-3-(5-(5-cyclopropoxypyridin-2-yl)-4-(2-fluorophenyl)-4H-1,2,4-triazol-3-yl)cyclobutan-1-amine dihydrochloride, intermediate for final product 7.

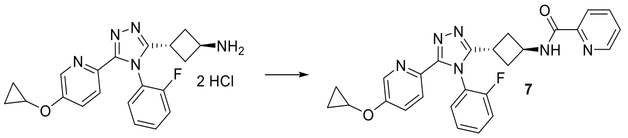

Step-1: In a microwave tube methyl 5-hydroxypicolinate (0.631 g, 4.0 mmol, 1.0 equiv.), potassium iodide (0.066 g, 0.400 mmol, 0.1 equiv.) and cesium carbonate (2.085 g, 6.40 mmol, 1.6 equiv.) were suspended in *N,N*-dimethylformamide (dry) (20 ml, 0.2 M). To the suspension was added bromo cyclopropane (0.481 ml, 6.00 mmol, 1.5 equiv.), the vial was capped and the reaction mixture was stirred in an oil bath at 120 °C overnight. An extra portion of bromo cyclopropane (0.481 ml, 6.00 mmol, 1.5 equiv.) was added and the reaction mixture was irradiated in a microwave oven at 200 °C for 18 hours. Reaction mixture was concentrated under reduced pressure. The residue was resuspended in aqueous sodium carbonate and extracted three times with CH_2Cl_2. Combined organic layers were dried over sodium sulfate, filtered and evaporated to dryness. The impure product was purified by flash column chromatography eluted with a gradient of methanol (0 to 5% then to 10%) in DCM. Desired fractions were combined and concentrated under reduced pressure to give methyl 5-cyclopropoxypicolinate as a yellow solid (50 mg, 5%). LC/MS (ESI) m/z for $C_{10}H_{11}NO_3$ 194 ($[M + H]^+$, calculated) 194 ($[M + H]^+$, found).

Step-2: 2-fluoroaniline (0.030 ml, 0.308 mmol, 1.5 equiv.) was dissolved in toluene (dry) (2.0 ml) under a nitrogen atmosphere and the solution was treated with a dropwise addition of trimethylaluminum (2 M in toluene, 0.154 ml, 0.308 mmol, 1.5 equiv.). After 15 minutes, this mixture was added to a solution of methyl 5-cyclopropoxypicolinate (0.050 g, 0.205 mmol, 1.0 equiv.) in toluene (dry) (5.0 ml) under a nitrogen atmosphere. The reaction mixture was stirred for 3 hours at 100 °C, followed by 3 days at room temperature. A new portion of the reagent was prepared from 2-fluoroaniline (9.89 µl, 0.103 mmol, 0.5 equiv.) and trimethylaluminum (2 M in toluene, 0.051 ml, 0.103 mmol, 0.5 equiv.) in toluene (dry) (1.0 ml) and after 30 minutes it was added to the main reaction mixture, which was heated again to 100 °C for 2 hours. The reaction mixture was quenched with 1N aqueous HCl. Some water was added and extracted with DCM. Combined organic layers were dried over sodium sulfate, filtered and evaporated to dryness. The impure product was purified by flash column chromatography on silica gel (gradient of ethyl acetate in heptane, 0% to 35%, then to 100%) to obtain 5-cyclopropoxy-*N*-(2-fluorophenyl)picolinamide as a colorless glass (36 mg, 64%). LC/MS (ESI) m/z for $C_{15}H_{13}FN_2O_2$ 273 ($[M + H]^+$, calculated) 273 ($[M + H]^+$, found).

Step-3: 5-cyclopropoxy-*N*-(2-fluorophenyl)pyridine-2-carbothioamide was prepared according to the General Procedure **B** as a yellow crystalline solid (34.6 mg, 92%). LC/MS (ESI) m/z for $C_{15}H_{13}FN_2OS$ 289 ($[M + H]^+$, calculated) 289 ($[M + H]^+$, found).

Step-4: methyl 5-cyclopropoxy-*N*-(2-fluorophenyl)pyridine-2-carbimidothioate was prepared according to the General Procedure **C** as a pale yellow semisolid (29 mg, 80%). LC/MS (ESI) m/z for $C_{16}H_{15}FN_2OS$ 303 ($[M + H]^+$, calculated) 303 ($[M + H]^+$, found).

Step-5: *tert*-butyl (*trans*-3-(5-(5-cyclopropoxypyridin-2-yl)-4-(2-fluorophenyl)-4H-1,2,4-triazol-3-yl)cyclobutyl)carbamate was prepared according to the General Procedure **D** as a pale ochre semisolid (34 mg, 70%). LC/MS (ESI) m/z for $C_{25}H_{28}FN_5O_3$ 466 ($[M + H]^+$, calculated) 466 ($[M + H]^+$, found).

Step-6: The title compound was prepared following to the General Procedure **E** and obtained as a pale yellow glass (36 mg, 100%). LC/MS (ESI) m/z for $C_{20}H_{20}FN_5O$ 366 ([M + H]+, calculated) 366 ([M + H]$^+$, found).

Preparation of *trans*-3-(4-(2-fluorophenyl)-5-(5-isopropoxypyridin-2-yl)-4H-1,2,4-triazol-3-yl)cyclobutan-1-amine dihydrochloride, intermediate for final product 8.

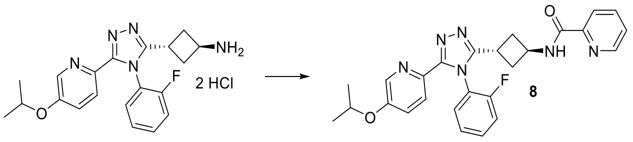

Step-1: In a microwave tube methyl 5-hydroxypicolinate (0.613 g, 4.0 mmol, 1.0 equiv.) and potassium carbonate (1.382 g, 10.00 mmol, 2.5 equiv.) were suspended in acetone (20 ml). The suspension was treated with 2-bromopropane (0.488 ml, 5.20 mmol, 1.3 equiv.), the vial was capped and the reaction mixture was stirred while heating in an oil bath at 80 °C for 42 hours. The mixture was then evaporated to dryness and the residue was resuspended in ethyl acetate. After trituration for 30 minutes, the suspension was filtered through celite rinsing with ethyl acetate. The filtrate was concentrated to dryness to obtain methyl 5-isopropoxypicolinate as a pale green liquid (814 mg, 100%). LC/MS (ESI) m/z for $C_{10}H_{13}NO_3$ 196 ([M + H]+, calculated) 196 ([M + H]$^+$, found).

Step-2: 2-fluoroaniline (0.267 g, 2.400 mmol, 1.2 equiv.) was dissolved in toluene (dry) (9.6 ml) under a nitrogen atmosphere and solution was treated with dropwise addition of trimethylaluminum, (2 M in toluene, 1.200 ml, 2.400 mmol, 1.2 equiv.). After 15 minutes, methyl 5-isopropoxypicolinate (0.407 g, 2.00 mmol, 1.0 equiv.) was added and the mixture was heated to 100 °C for 2 hours. The mixture was cooled down and quenched with 6 ml of 1N aqueous HCl. Water was added and the suspension was stirred overnight at ambient temperature. A clear, two phase system was obtained. It was further diluted with DCM and water and the aqueous layer was extracted with DCM. The combined extracts were dried over sodium sulfate, filtered and evaporated to dryness to obtain *N*-(2-fluorophenyl)-5-isopropoxypicolinamide as an orange oil (564 mg, 98%). LC/MS (ESI) m/z for $C_{15}H_{15}FN_2O_2$ 275 ([M + H]+, calculated) 275 ([M + H]$^+$, found).

Step-3: *N*-(2-fluorophenyl)-5-isopropoxypyridine-2-carbothioamide was prepared according to the General Procedure **B** as a yellow solid (460 mg, 80%). LC/MS (ESI) m/z for $C_{15}H_{15}FN_2OS$ 291 ([M + H]+, calculated) 291 ([M + H]$^+$, found).

Step-4: methyl *N*-(2-fluorophenyl)-5-isopropoxypyridine-2-carbimidothioate was prepared according to the General Procedure **C** as a yellow oil (461 mg, 96%). LC/MS (ESI) m/z for $C_{16}H_{17}FN_2OS$ 305 ([M + H]+, calculated) 305 ([M + H]$^+$, found).

Step-5: *tert*-butyl (*trans*-3-(4-(2-fluorophenyl)-5-(5-isopropoxypyridin-2-yl)-4H-1,2,4-triazol-3-yl)cyclobutyl)carbamate was prepared according to the General Procedure **D** as a brown semisolid (345 mg, 65%). LC/MS (ESI) m/z for $C_{25}H_{30}FN_5O_3$ 468 ([M + H]$^+$, calculated) 468 ([M + H]$^+$, found).

Step-6: The title compound was prepared following to the General Procedure **E** and obtained as a brown glass (300 mg, 100%). LC/MS (ESI) m/z for $C_{20}H_{22}FN_5O$ 368 ([M + H]$^+$, calculated) 368 ([M + H]$^+$, found).

Preparation of *trans*-3-(4-(2-fluorophenyl)-5-phenyl-4H-1,2,4-triazol-3-yl)cyclobutan-1-amine dihydrochloride, intermediate for final product 9.

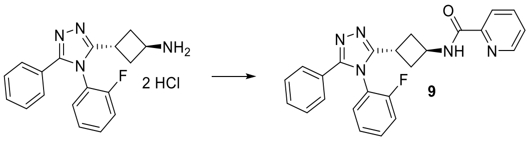

Step-1: 2-fluoroaniline (1.157 mL, 12.0 mmol, 1.2 equiv.) was dissolved in toluene (dry) (24 ml) under a nitrogen atmosphere, then the solution was treated with a dropwise addition of trimethylaluminum (2 M in toluene, 6.00 ml,

12.00 mmol, 1.2 equiv.). After stirring for 1 hour methyl benzoate (1.251 mL, 10.0 mmol, 1.0 equiv.) was added and the mixture was stirred for 2 hours at 90 °C and then overnight at room temperature. Then it was quenched with 1N aqueous HCl, the mixture was further diluted with DCM and water and the aqueous layer was extracted with DCM. The orange extracts were dried over sodium sulfate, filtered and evaporated thoroughly to dryness to obtain *N*-(2-fluorophenyl)benzamide as a beige solid (2.18 g, 100%). LC/MS (ESI) m/z for $C_{13}H_{10}FNO$ 216 ($[M + H]^+$, calculated) 216 ($[M + H]^+$, found).

Step-2: *N*-(2-fluorophenyl)benzothioamide was prepared according to the General Procedure **B** as a yellow oil (2.28 g, 98%). LC/MS (ESI) m/z for $C_{13}H_{10}FNS$ 232 ($[M + H]^+$, calculated) 232 ($[M + H]^+$, found).

Step-3: methyl *N*-(2-fluorophenyl)benzimidothioate was prepared according to the General Procedure **C** as a yellow oil (2.23 g, 92%). LC/MS (ESI) m/z for $C_{14}H_{12}FNS$ 246 ($[M + H]^+$, calculated) 246 ($[M + H]^+$, found).

Step-4: *tert*-butyl (*trans*-3-(4-(2-fluorophenyl)-5-phenyl-4H-1,2,4-triazol-3-yl)cyclobutyl)carbamate was prepared according to the General Procedure **D** as a white foam (739 mg, 43%). LC/MS (ESI) m/z for $C_{23}H_{25}FN_4O_2$ 409 ($[M + H]^+$, calculated) 409 ($[M + H]^+$, found).

Step-5: The title compound was prepared following to the General Procedure **E** and obtained as a white solid (665 mg, 100%). LC/MS (ESI) m/z for $C_{18}H_{17}FN_4$ 309($[M + H]^+$, calculated) 309 ($[M + H]^+$, found).

Preparation of *trans*-3-(4-(2-fluorophenyl)-5-(pyrazin-2-yl)-4H-1,2,4-triazol-3-yl)cyclobutan-1-amine dihydrochloride, intermediate for final product 10.

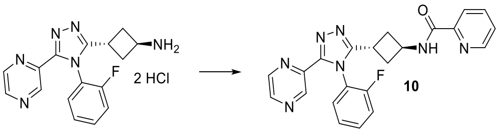

Step-1: *N*-(2-fluorophenyl)pyrazine-2-carboxamide was prepared following the General Procedure **A**, method a) as an off-white solid (812 mg, 88%). LC/MS (ESI) m/z for $C_{11}H_8FN_3O$ 218 ($[M + H]^+$, calculated) 218 ($[M + H]^+$, found).

Step-2: *N*-(2-fluorophenyl)pyrazine-2-carbothioamide was prepared according to the General Procedure **B** as a yellow solid (333 mg, 37%). LC/MS (ESI) m/z for $C_{11}H_8FN_3S$ 234 ($[M + H]^+$, calculated) 234 ($[M + H]^+$, found).

Step-3: methyl *N*-(2-fluorophenyl)pyrazine-2-carbimidothioate was prepared according to the General Procedure **C** as a yellow oil (333 mg, 92%). GC/MS (ESI) m/z for $C_{12}H_{10}FN_3S$ 247 ($[M + H]^+$, calculated) 247 ($[M]^+$, found).

Step-4: *tert*-butyl (*trans*-3-(4-(2-fluorophenyl)-5-(pyrazin-2-yl)-4H-1,2,4-triazol-3-yl)cyclobutyl)carbamate was prepared according to the General Procedure **D** as a brown oil (141 mg, 33%). LC/MS (ESI) m/z for $C_{21}H_{23}FN_6O_2$ 411 ($[M + H]^+$, calculated) 411 ($[M + H]^+$, found).

Step-5: The title compound was prepared following to the General Procedure **E** and obtained as a purple glass (nd. mg, nd.%). LC/MS (ESI) m/z for $C_{16}H_{15}FN_6$ 311 ($[M + H]^+$, calculated) 311 ($[M + H]^+$, found).

Preparation of *trans*-3-(5-(5-ethoxypyridin-2-yl)-4-phenyl-4H-1,2,4-triazol-3-yl)cyclobutan-1-amine trihydrochloride, intermediate for final product 11.

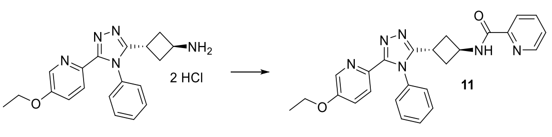

Step-1: 5-ethoxy-*N*-phenylpicolinamide was prepared following the General Procedure **A**, method a) as a white crystalline solid (560 mg, 77%). LC/MS (ESI) m/z for $C_{14}H_{14}N_2O_2$ 243 ($[M + H]^+$, calculated) 243 ($[M + H]^+$, found).

Step-2: 5-ethoxy-*N*-phenylpyridine-2-carbothioamide was prepared according to the General Procedure **B** as a yellow solid (455 mg, 76%). LC/MS (ESI) m/z for $C_{14}H_{14}N_2OS$ 259 ([M + H]$^+$, calculated) 259 ([M + H]$^+$, found).

Step-3: methyl 5-ethoxy-*N*-phenylpyridine-2-carbimidothioate was prepared according to the General Procedure **C** as a yellow solid (449 mg, 88%). LC/MS (ESI) m/z for $C_{15}H_{16}N_2OS$ 273 ([M + H]$^+$, calculated) 273 ([M + H]$^+$, found).

Step-4: *tert*-butyl (*trans*-3-(5-(5-ethoxypyridin-2-yl)-4-phenyl-4H-1,2,4-triazol-3-yl)cyclobutyl)carbamate was prepared according to the General Procedure **D** as a yellow foam (595 mg, 76%). LC/MS (ESI) m/z for $C_{24}H_{29}N_5O_3$ 436 ([M + H]$^+$, calculated) 436 ([M + H]$^+$, found).

Step-5: The compound was prepared following to the General Procedure **E** and obtained as a white solid (462 mg, 92%). LC/MS (ESI) m/z for $C_{19}H_{21}N_5O$ 336 ([M + H]$^+$, calculated) 336 ([M + H]$^+$, found).

Preparation of *trans*-3-(4-(3-chlorophenyl)-5-(5-ethoxypyridin-2-yl)-4H-1,2,4-triazol-3-yl)cyclobutan-1-amine dihydrochloride, intermediate for final product 12.

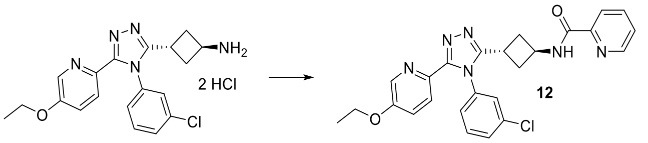

Step-1: *N*-(3-chlorophenyl)-5-ethoxypicolinamide was prepared following the General Procedure **A**, method a) as a white crystalline solid (106 mg, 82%). LC/MS (ESI) m/z for $C_{14}H_{13}ClN_2O_2$ 277/279 ([M + H]$^+$, calculated) 277/279 ([M + H]$^+$, found).

Step-2: *N*-(3-chlorophenyl)-5-ethoxypyridine-2-carbothioamide was prepared according to the General Procedure **B** as a yellow solid (95 mg, 85%). LC/MS (ESI) m/z for $C_{14}H_{13}ClN_2OS$ 293/295 ([M + H]$^+$, calculated) 293/295 ([M + H]$^+$, found).

Step-3: methyl *N*-(3-chlorophenyl)-5-ethoxypyridine-2-carbimidothioate was prepared according to the General Procedure **C** as a yellow solid (92 mg, 93%). LC/MS (ESI) m/z for $C_{15}H_{15}ClN_2OS$ 307/309 ([M + H]$^+$, calculated) 307/309 ([M + H]$^+$, found).

Step-4: *tert*-butyl (*trans*-3-(4-(3-chlorophenyl)-5-(5-ethoxypyridin-2-yl)-4H-1,2,4-triazol-3-yl)cyclobutyl)carbamate was prepared according to the General Procedure **D** as a white foam (130 mg, 90%). LC/MS (ESI) m/z for $C_{24}H_{28}ClN_5O_3$ 470/472 ([M + H]$^+$, calculated) 470/472 ([M + H]$^+$, found).

Step-5: The title compound was prepared following to the General Procedure **E** and obtained as a white solid (131 mg, 100%). LC/MS (ESI) m/z for $C_{19}H_{20}ClN_5O$ 370/372 ([M + H]$^+$, calculated) 370/372 ([M + H]$^+$, found).

Preparation of *trans*-3-(5-(5-ethoxypyridin-2-yl)-4-(5-methylthiophen-2-yl)-4H-1,2,4-triazol-3-yl)cyclobutan-1-amine trihydrochloride, intermediate for final products 13, 22a, 22b and 23.

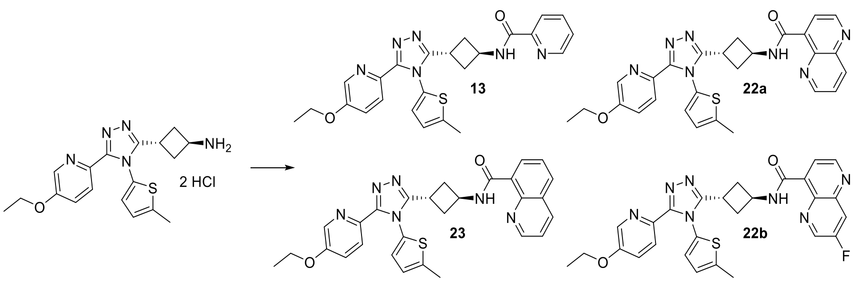

Step-1: 5-ethoxy-*N*-(5-methylthiophen-2-yl)picolinamide was prepared following the General Procedure **A**, method b) as a beige solid (273 mg, 77%). LC/MS (ESI) m/z for $C_{13}H_{14}N_2O_2S$ 263 ([M + H]$^+$, calculated) 263 ([M + H]$^+$, found).

Step-2: 5-ethoxy-*N*-(5-methylthiophen-2-yl)pyridine-2-carbothioamide was prepared according to the General Procedure **B** as a yellow solid (176 mg, 57%). LC/MS (ESI) m/z for $C_{13}H_{14}N_2OS_2$ 279 ([M + H]$^+$, calculated) 279 ([M + H]$^+$, found).

Step-3: methyl 5-ethoxy-*N*-(5-methylthiophen-2-yl)pyridine-2-carbimidothioate was prepared according to the General Procedure **C** as a yellow oil (191 mg, 99%). LC/MS (ESI) m/z for $C_{14}H_{16}N_2OS_2$ 293 ([M + H]$^+$, calculated) 293 ([M + H]$^+$, found).

Step-4: *tert*-butyl (*trans*-3-(5-(5-ethoxypyridin-2-yl)-4-(5-methylthiophen-2-yl)-4H-1,2,4-triazol-3-yl)cyclobutyl)carbamate was prepared according to the General Procedure **D** as a brown oil (188 mg, 52%). LC/MS (ESI) m/z for $C_{23}H_{29}N_5O_3S$ 456 ([M + H]$^+$, calculated) 456 ([M + H]$^+$, found).

Step-5: The title compound was prepared following to the General Procedure **E** and obtained as a brown solid (153 mg, 72%). LC/MS (ESI) m/z for $C_{18}H_{21}N_5OS$ 356 ([M + H]$^+$, calculated) 356 ([M + H]$^+$, found).

Preparation of *trans*-3-(4-(5-chlorothiophen-2-yl)-5-(5-ethoxypyridin-2-yl)-4H-1,2,4-triazol-3-yl)cyclobutan-1-amine dihydrochloride, intermediate for final product 14.

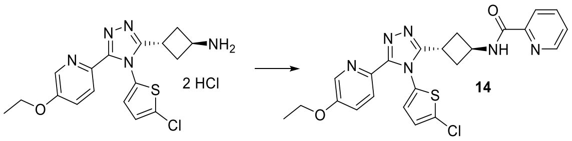

Step-1: 5-chlorothiophene-2-carboxylic acid (8.13 g, 50 mmol, 1.00 equiv.) was placed under a nitrogen atmosphere. About 10 equivalents of t-Butanol (47.5 ml) was added as solvent followed by triethylamine (7.32 ml, 52.5 mmol, 1.05 equiv.) and DPPA diphenylphosphoryl azide (11.38 ml, 52.5 mmol, 1.05 equiv.). The reaction mixture was stirred at 90 °C for 16 hours. The reaction mixture was concentrated under reduced pressure, and the residue was partitioned between water and MTBE. After phase separation, the organic layer was rinsed once with water and then with brine. The aqueous phases were extracted twice more with MTBE and the combined organic layer were dried over sodium sulfate, filtered and concentrated under reduced pressure to obtain *tert*-butyl (5-chlorothiophen-2-yl)carbamate as a grey solid (11.75 g, 98%). LC/MS (ESI) m/z for $C_9H_{12}ClNO_2S$ 232/234 ([M + H]$^+$, calculated) 232/234 ([M - H]$^+$, found).

Step-2: *tert*-butyl (5-chlorothiophen-2-yl)carbamate (4.82 g, 20.0 mmol, 1.0 equiv.) was dissolved in 1,4-dioxane (25 ml) followed by the addition of 4 M hydrochloric acid in 1,4-dioxane (25.00 ml, 100 mmol, 5.0 equiv.). The reaction mixture was stirred for 22 hours at room temperature. Followed by 4 hours at 60 °C. The suspension was cooled in an ice-bath and filtered through a P3 glass filter under a nitrogen flow, rinsing with MTBE and drying in a nitrogen flow to obtain 5-chlorothiophen-2-amine hydrochloride as brown solid (3.26 g, 89%). LC/MS (ESI) m/z for C_4H_4ClNS 132/134 ([M + H]$^+$, calculated) no mass found.

Step-3: *N*-(5-chlorothiophen-2-yl)-5-ethoxypicolinamide was prepared following the General Procedure **A**, method b) using 5-ethoxypicolinate as described in Step-1 in the synthesis for final products **16a** and **16b** as a brown solid (516 mg, 79%). LC/MS (ESI) m/z for $C_{12}H_{11}ClN_2O_2S$ 283/285 ([M + H]$^+$, calculated) 283/285 ([M + H]$^+$, found).

Step-4: *N*-(5-chlorothiophen-2-yl)-5-ethoxypyridine-2-carbothioamide was prepared according to the General Procedure **B** as a yellow solid (135 mg, 24%). LC/MS (ESI) m/z for $C_{12}H_{11}ClN_2OS_2$ 299/301 ([M + H]$^+$, calculated) 299/301 ([M + H]$^+$, found).

Step-5: methyl *N*-(5-chlorothiophen-2-yl)-5-ethoxypyridine-2-carbimidothioate was prepared according to the General Procedure **C** as a yellow oil (98 mg, 90%). LC/MS (ESI) m/z for $C_{13}H_{13}ClN_2OS_2$ 313/315 ([M + H]$^+$, calculated) 313/315 ([M + H]$^+$, found).

Step-6: *tert*-butyl (*trans*-3-(4-(5-chlorothiophen-2-yl)-5-(5-ethoxypyridin-2-yl)-4H-1,2,4-triazol-3-yl)cyclobutyl)carbamate was prepared according to the General Procedure **D** as a brown oil (97 mg, 48%). LC/MS (ESI) m/z for $C_{22}H_{26}ClN_5O_3S$ 476/478 ([M + H]$^+$, calculated) 476/478 ([M + H]$^+$, found).

Step-7: The title compound was prepared following to the General Procedure **E** and obtained as a brown glass (67 mg, 39%). LC/MS (ESI) m/z for $C_{17}H_{18}ClN_5OS$ 376/378 ([M + H]$^+$, calculated) 376/378 ([M + H]$^+$, found).

Preparation of *trans*-3-(5-(5-ethoxypyridin-2-yl)-4-(pyridin-2-yl)-4H-1,2,4-triazol-3-yl)cyclobutan-1-amine trihydrochloride, intermediate for final products 16a and 16b.

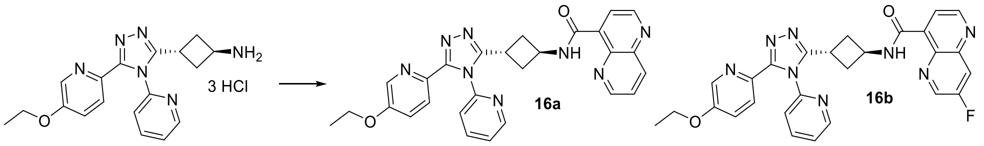

Step-1: methyl 5-hydroxypicolinate (6.13 g, 40.00 mmol, 1.0 equiv.) and potassium carbonate (13.82 g, 100 mmol, 2.5 equiv.) were suspended in acetone (160 ml). The suspension was treated with iodoethane (4.00 ml, 50.0 mmol, 1.25 equiv.). The reaction mixture was stirred for 17 hours at room temperature, followed by 24 hours at 50 °C. The reaction mixture was evaporated to dryness and the residue was triturated with DCM. The residue was filtered off on a celite pad rinsing with DCM and the yellow filtrate was concentrated to obtain methyl 5-ethoxypicolinate as a yellow solid (7.24 g, 99%). LC/MS (ESI) m/z for $C_9H_{11}NO_3$ 182 ([M + H]$^+$, calculated) 182 ([M + H]$^+$, found).

Step-2: pyridin-2-amine (0.339 g, 3.60 mmol, 1.2 equiv.) was dissolved in toluene (dry) (15 ml) under a nitrogen atmosphere, then the solution was treated with a dropwise addition of trimethylaluminum (2 M in toluene, 1.800 ml, 3.60 mmol, 1.2 equiv.). After stirring for 1 hour methyl 5-ethoxypicolinate (0.549 g, 3.0 mmol, 1.0 equiv.) was added and the mixture was stirred for 2 hours at 90 °C and then overnight at room temperature. Then it was quenched with 1N aqueous HCl, the mixture was further diluted with DCM and water and the aqueous layer was extracted with DCM. The orange extracts were dried over sodium sulfate, filtered and evaporated thoroughly to dryness to obtain 5-ethoxy-*N*-(pyridin-2-yl)picolinamide as an off-white solid (737 mg, 99%). LC/MS (ESI) m/z for $C_{13}H_{13}N_3O_2$ 244 ([M + H]$^+$, calculated) 244 ([M + H]$^+$, found).

Step-3: 5-ethoxy-*N*-(pyridin-2-yl)pyridine-2-carbothioamide was prepared according to the General Procedure **B** as a yellow solid (449 mg, 58%). LC/MS (ESI) m/z for $C_{13}H_{13}N_3OS$ 260 ([M + H]$^+$, calculated) 260 ([M + H]$^+$, found).

Step-4: methyl 5-ethoxy-*N*-(pyridin-2-yl)pyridine-2-carbimidothioate was prepared according to the General Procedure **C** as a yellow crystalline solid (175 mg, 31%). LC/MS (ESI) m/z for $C_{14}H_{15}N_3OS$ 274 ([M + H]$^+$, calculated) 274 ([M + H]$^+$, found).

Step-5: *tert*-butyl (*trans*-3-(5-(5-ethoxypyridin-2-yl)-4-(pyridin-2-yl)-4H-1,2,4-triazol-3-yl)cyclobutyl)carbamate was prepared according to the General Procedure **D** as a colorless glass (134 mg, 97%). LC/MS (ESI) m/z for $C_{23}H_{28}N_6O_3$ 437 ([M + H]$^+$, calculated) 437 ([M + H]$^+$, found).

Step-6: The title compound was prepared following to the General Procedure **E** and obtained as a white solid (125 mg, 95%). LC/MS (ESI) m/z for $C_{18}H_{20}N_6O$ 337 ([M + H]$^+$, calculated) 337 ([M + H]$^+$, found).

Preparation of *trans*-3-(5-(5-ethoxypyridin-2-yl)-4-(pyridin-3-yl)-4H-1,2,4-triazol-3-yl)cyclobutan-1-amine trihydrochloride, intermediate for final products 17a and 17b.

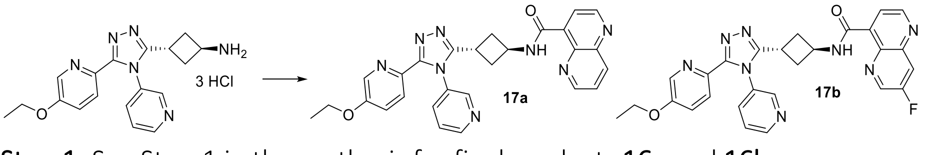

Step-1: See Step-1 in the synthesis for final products **16a** and **16b**

Step-2: pyridin-3-amine (0.339 g, 3.60 mmol, 1.2 equiv.) was dissolved in toluene (dry) (15 ml) under a nitrogen atmosphere, then the solution was treated with a dropwise addition of trimethylaluminum (2 M in toluene, 1.800 ml, 3.60 mmol, 1.2 equiv.). After stirring for 1 hour methyl 5-ethoxypicolinate (0.549 g, 3.0 mmol, 1.0 equiv.) was added and the mixture was stirred for 2 hours at 90 °C and then overnight at room temperature. Then the reaction mixture was quenched with 1N aqueous HCl, the mixture and further diluted with DCM and water and the aqueous layer was extracted with DCM once more. The orange extracts were dried over sodium sulfate, filtered and evaporated thoroughly to dryness to obtain 5-ethoxy-*N*-(pyridin-3-yl)picolinamide as an off-white solid (716 mg, 96%). LC/MS (ESI) m/z for $C_{13}H_{13}N_3O_2$ 244 ([M + H]$^+$, calculated) 244 ([M + H]$^+$, found).

Step-3: 5-ethoxy-*N*-(pyridin-3-yl)pyridine-2-carbothioamide was prepared according to the General Procedure **B** as a yellow solid (641 mg, 86%). LC/MS (ESI) m/z for $C_{13}H_{13}N_3OS$ 260 ([M + H]$^+$, calculated) 260 ([M + H]$^+$, found).

Step-4: methyl 5-ethoxy-*N*-(pyridin-3-yl)pyridine-2-carbimidothioate was prepared according to the General Procedure **C** as a yellow crystalline solid (377 mg, 50%). LC/MS (ESI) m/z for $C_{14}H_{15}N_3OS$ 274 ([M + H]$^+$, calculated) 274 ([M + H]$^+$, found).

Step-5: *tert*-butyl ((1r,3r)-3-(5-(5-ethoxypyridin-2-yl)-4-(pyridin-3-yl)-4H-1,2,4-triazol-3-yl)cyclobutyl)carbamate was prepared according to the General Procedure **D** as a whitish glass (277 mg, 92%). LC/MS (ESI) m/z for $C_{23}H_{28}N_6O_3$ 437 ([M + H]$^+$, calculated) 437 ([M + H]$^+$, found).

Step-6: The title compound was prepared following to the General Procedure **E** and obtained as a white solid (264 mg, 99%). LC/MS (ESI) m/z for $C_{18}H_{20}N_6O$ 337 ([M + H]$^+$, calculated) 337 ([M + H]$^+$, found).

Preparation of *trans*-3-(5-(5-ethoxypyridin-2-yl)-4-(pyridin-4-yl)-4H-1,2,4-triazol-3-yl)cyclobutan-1-amine trihydrochloride, intermediate for final products 18a and 18b.

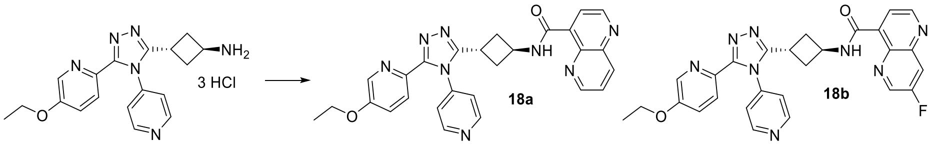

Step-1: See Step-1 in the synthesis for final products **16a** and **16b**

Step-2: pyridin-4-amine (0.339 g, 3.60 mmol, 1.2 equiv.) was dissolved in toluene (dry) (15 ml) under a nitrogen atmosphere, then the solution was treated with a dropwise addition of trimethylaluminum (2 M in toluene, 1.800 ml, 3.60 mmol, 1.2 equiv.). After stirring for 1 hour methyl 5-ethoxypicolinate (0.549 g, 3.0 mmol, 1.0 equiv.) was added and the mixture was stirred for 2 hours at 90 °C and then overnight at room temperature. Then the reaction mixture was quenched with 1N aqueous HCl and further diluted with DCM and water and the aqueous layer was extracted with DCM once more. The orange extracts were dried over sodium sulfate, filtered and evaporated thoroughly to dryness to obtain 5-ethoxy-*N*-(pyridin-4-yl)picolinamide as an orange solid (649 mg, 84%). LC/MS (ESI) m/z for $C_{13}H_{13}N_3O_2$ 244 ([M + H]$^+$, calculated) 244 ([M + H]$^+$, found).

Step-3: 5-ethoxy-*N*-(pyridin-4-yl)pyridine-2-carbothioamide was prepared according to the General Procedure **B** as a yellow solid (381 mg, 55%). LC/MS (ESI) m/z for $C_{13}H_{13}N_3OS$ 260 ([M + H]$^+$, calculated) 260 ([M + H]$^+$, found).

Step-4: ethyl 5-ethoxy-*N*-(pyridin-4-yl)pyridine-2-carbimidothioate was prepared according to the General Procedure **C** were iodoethane was used instead of iodomethane as a yellow oil (222 mg, 52%). LC/MS (ESI) m/z for $C_{15}H_{17}N_3OS$ 288 ([M + H]$^+$, calculated) 288 ([M + H]$^+$, found).

Step-5: *tert*-butyl (*trans*-3-(5-(5-ethoxypyridin-2-yl)-4-(pyridin-4-yl)-4H-1,2,4-triazol-3-yl)cyclobutyl)carbamate was prepared according to the General Procedure **D** as a colorless oil (269 mg, 83%). LC/MS (ESI) m/z for $C_{23}H_{28}N_6O_3$ 437 ([M + H]$^+$, calculated) 437 ([M + H]$^+$, found).

Step-6: The title compound was prepared following to the General Procedure **E** and obtained as a white solid (262 mg, 100%). LC/MS (ESI) m/z for $C_{18}H_{20}N_6O$ 337 ([M + H]$^+$, calculated) 337 ([M + H]$^+$, found).

Preparation of 3-(5-(5-ethoxypyridin-2-yl)-4-(pyridin-2-yl)-4H-1,2,4-triazol-3-yl)bicyclo[1.1.1]pentan-1-amine trihydrochloride, intermediate for final product 19.

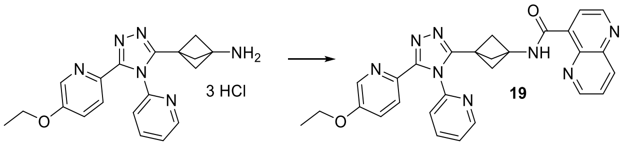

Step-1: See Step-1 in the synthesis for final products **16a** and **16b**

Step-2: pyridin-2-amine (0.339 g, 3.60 mmol, 1.2 equiv.) was dissolved in toluene (dry) (15 ml) under a nitrogen atmosphere, then the solution was treated with a dropwise addition of trimethylaluminum (2 M in toluene, 1.800 ml, 3.60 mmol, 1.2 equiv.). After stirring for 1 hour methyl 5-ethoxypicolinate (0.549 g, 3.0 mmol, 1.0 equiv.) was added and the mixture was stirred for 2 hours at 90 °C and then overnight at room temperature. Then the reaction mixture was quenched with 1N aqueous HCl and further diluted with DCM and water and the aqueous layer was extracted with DCM once more. The orange extracts were dried over sodium sulfate, filtered and evaporated thoroughly to dryness to obtain 5-ethoxy-*N*-(pyridin-2-yl)picolinamide as an off-white solid (737 mg, 99%). LC/MS (ESI) m/z for $C_{13}H_{13}N_3O_2$ 244 ([M + H]$^+$, calculated) 244 ([M + H]$^+$, found).

Step-3: 5-ethoxy-*N*-(pyridin-2-yl)pyridine-2-carbothioamide was prepared according to the General Procedure **B** as a yellow solid (449 mg, 58%). LC/MS (ESI) m/z for $C_{13}H_{13}N_3OS$ 260 ([M + H]$^+$, calculated) 260 ([M + H]$^+$, found).

Step-4: methyl 5-ethoxy-*N*-(pyridin-2-yl)pyridine-2-carbimidothioate was prepared according to the General Procedure **C** as a yellow crystalline solid (175 mg, 31%). LC/MS (ESI) m/z for $C_{14}H_{15}N_3OS$ 274([M + H]$^+$, calculated) 274 ([M + H]$^+$, found).

Step-5: *tert*-butyl (3-(5-(5-ethoxypyridin-2-yl)-4-(pyridin-2-yl)-4H-1,2,4-triazol-3-yl)bicyclo[1.1.1]pentan-1-yl)carbamate was prepared according to the General Procedure **D** as a colorless glass (116 mg, 83%). LC/MS (ESI) m/z for $C_{24}H_{28}N_6O_3$ 449 ([M + H]$^+$, calculated) 449 ([M + H]$^+$, found).

Step-6: The title compound was prepared following to the General Procedure **E** and obtained as a white solid (108 mg, 97%). LC/MS (ESI) m/z for $C_{19}H_{20}N_6O$ 349 ([M + H]$^+$, calculated) 349 ([M + H]$^+$, found).

Preparation of 3-(5-(5-ethoxypyridin-2-yl)-4-(pyridin-3-yl)-4H-1,2,4-triazol-3-yl)bicyclo[1.1.1]pentan-1-amine trihydrochloride, intermediate for final product 20.

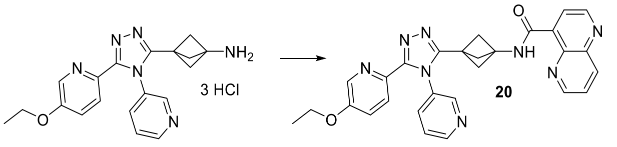

Step-1: See Step-1 in the synthesis for final products **16a** and **16b**

Step-2: pyridin-3-amine (0.339 g, 3.60 mmol, 1.2 equiv.) was dissolved in toluene (dry) (15 ml) under a nitrogen atmosphere, then the solution was treated with a dropwise addition of trimethylaluminum (2 M in toluene, 1.800 ml, 3.60 mmol, 1.2 equiv.). After stirring for 1 hour methyl 5-ethoxypicolinate (0.549 g, 3.0 mmol, 1.0 equiv.) was added and the mixture was stirred for 2 hours at 90 °C and then overnight at room temperature. Then the reaction mixture was quenched with 1N aqueous HCl and further diluted with DCM and water, the aqueous layer was extracted with DCM once more. The orange extracts were dried over sodium sulfate, filtered and evaporated thoroughly to dryness to obtain 5-ethoxy-*N*-(pyridin-3-yl)picolinamide as an off-white solid (716 mg, 96%). LC/MS (ESI) m/z for $C_{13}H_{13}N_3O_2$ 244 ($[M + H]^+$, calculated) 244 ($[M + H]^+$, found).

Step-3: 5-ethoxy-*N*-(pyridin-3-yl)pyridine-2-carbothioamide was prepared according to the General Procedure **B** as a yellow solid (641 mg, 86%). LC/MS (ESI) m/z for $C_{13}H_{13}N_3OS$ 260 ($[M + H]^+$, calculated) 260 ($[M + H]^+$, found).

Step-4: methyl 5-ethoxy-*N*-(pyridin-3-yl)pyridine-2-carbimidothioate was prepared according to the General Procedure **C** as a yellow crystalline solid (377 mg, 50%). LC/MS (ESI) m/z for $C_{14}H_{15}N_3OS$ 274 ($[M + H]^+$, calculated) 274 ($[M + H]^+$, found).

Step-5: *tert*-butyl (3-(5-(5-ethoxypyridin-3-yl)-4-(pyridin-2-yl)-4H-1,2,4-triazol-3-yl)bicyclo[1.1.1]pentan-1-yl)carbamate was prepared according to the General Procedure **D** as a yellow semisolid (263 mg, 77%). LC/MS (ESI) m/z for $C_{24}H_{28}N_6O_3$ 449 ($[M + H]^+$, calculated) 449 ($[M + H]^+$, found).

Step-6: The title compound was prepared following to the General Procedure **E** and obtained as a white solid (233 mg, 100%). LC/MS (ESI) m/z for $C_{19}H_{20}N_6O$ 349 ($[M + H]^+$, calculated) 349 ($[M + H]^+$, found).

Preparation of 3-(5-(5-ethoxypyridin-2-yl)-4-(pyridin-4-yl)-4H-1,2,4-triazol-3-yl)bicyclo[1.1.1]pentan-1-amine trihydrochloride, intermediate for final product 21.

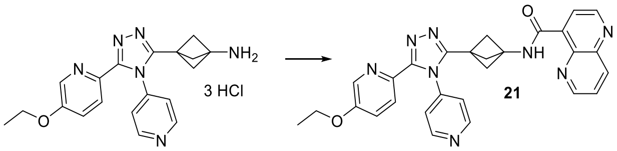

Step-1: See Step-1 in the synthesis for final products **16a** and **16b**.

Step-2: pyridin-4-amine (0.339 g, 3.60 mmol, 1.2 equiv.) was dissolved in toluene (dry) (15 ml) under a nitrogen atmosphere, then the solution was treated with a dropwise addition of trimethylaluminum (2 M in toluene, 1.800 ml, 3.60 mmol, 1.2 equiv.). After stirring for 1 hour methyl 5-ethoxypicolinate (0.549 g, 3.0 mmol, 1.0 equiv.) was added and the mixture was stirred for 2 hours at 90 °C and then overnight at room temperature. Then the reaction mixture was quenched with 1N aqueous HCl and further diluted with DCM and water, the aqueous layer was extracted with DCM once more. The orange extracts were dried over sodium sulfate, filtered and evaporated thoroughly to dryness to obtain 5-ethoxy-*N*-(pyridin-4-yl)picolinamide as an orange solid (649 mg, 84%). LC/MS (ESI) m/z for $C_{13}H_{13}N_3O_2$ 244 ($[M + H]^+$, calculated) 244 ($[M + H]^+$, found).

Step-3: 5-ethoxy-*N*-(pyridin-4-yl)pyridine-2-carbothioamide was prepared according to the General Procedure **B** as a yellow solid (381 mg, 55%). LC/MS (ESI) m/z for $C_{13}H_{13}N_3OS$ 260 ($[M + H]^+$, calculated) 260 ($[M + H]^+$, found).

Step-4: ethyl 5-ethoxy-*N*-(pyridin-4-yl)pyridine-2-carbimidothioate was prepared according to the General Procedure **C** were iodoethane was used instead of iodomethane as a yellow oil (222 mg, 52%). LC/MS (ESI) m/z for $C_{15}H_{17}N_3OS$ 288 ([M + H]$^+$, calculated) 288 ([M + H]$^+$, found).

Step-5: *tert*-butyl (3-(5-(5-ethoxypyridin-4-yl)-4-(pyridin-2-yl)-4H-1,2,4-triazol-3-yl)bicyclo[1.1.1]pentan-1-yl)carbamate was prepared according to the General Procedure **D** as an off-white solid (17 mg, 98%). LC/MS (ESI) m/z for $C_{24}H_{28}N_6O_3$ 449 ([M + H]$^+$, calculated) 449 ([M + H]$^+$, found).

Step-6: The title compound was prepared following to the General Procedure **E** and obtained as a white solid (17 mg, 99%). LC/MS (ESI) m/z for $C_{19}H_{20}N_6O$ 349 ([M + H]$^+$, calculated) 349 ([M + H]$^+$, found).

Preparation of *trans*-3-(5-(5-(difluoromethoxy)pyridin-2-yl)-4-(2-fluorophenyl)-4H-1,2,4-triazol-3-yl)cyclobutan-1-amine dihydrochloride, intermediate for final products 29a and 29b.

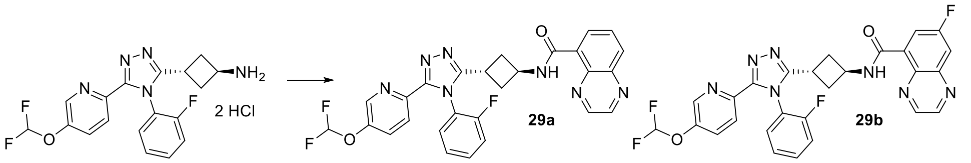

Step-1: potassium carbonate (0.829 g, 6.00 mmol, 1.5 equiv.) was suspended in *N,N*-dimethylformamide (4.0 ml) and then heated up to 100 °C. To the hot suspension a solution of methyl 5-hydroxypicolinate (0.631 g, 4.0 mmol, 1.0 equiv.) and sodium 2-chloro-2,2-difluoroacetate (1.220 g, 8.00 mmol, 2.0 equiv.) in *N,N*-dimethylformamide (8.0 ml) was added dropwise over a period of 20 minutes. After the addition the reaction mixture was allowed to cool to room temperature. Water was added and the product was extracted DCM, the organic extracts were rinsed once with water, dried over sodium sulfate, filtered and evaporated to dryness at not too high vacuum. The crude was flashed on a 24 gram silica gel cartridge eluted with a gradient of ethyl acetate (10% via 50% to 100%) in heptane to obtain methyl 5-(difluoromethoxy)picolinate as a colorless oil (676 mg, 83%). LC/MS (ESI) m/z for $C_8H_7F_2NO_3$ 204 ([M + H]$^+$, calculated) 204 ([M + H]$^+$, found).

Step-2: 2-fluoroaniline (0.267 g, 2.400 mmol, 1.2 equiv.) was dissolved in toluene (dry) (10 ml) under a nitrogen atmosphere and solution was treated with dropwise addition of trimethylaluminum, (2 M in toluene, 1.200 ml, 2.400 mmol, 1.2 equiv.). After 15 minutes, methyl 5-(difluoromethoxy)picolinate (0.406 g, 2.00 mmol, 1.0 equiv.) was added and the mixture was heated to 100 °C for 1 hour. The mixture was cooled down and quenched with 1N aqueous HCl. Water was added aqueous layer was extracted with DCM. The combined extracts were dried over sodium sulfate, filtered and evaporated to dryness to obtain a solid. This was stripped with acetonitrile to obtain 5-(difluoromethoxy)-*N*-(2-fluorophenyl)picolinamide as a beige solid (563 mg, 99%). LC/MS (ESI) m/z for $C_{13}H_9F_3N_2O_2$ 283 ([M + H]$^+$, calculated) 283 ([M + H]$^+$, found).

Step-3: 5-(difluoromethoxy)-*N*-(2-fluorophenyl)pyridine-2-carbothioamide was prepared according to the General Procedure **B** as a yellow solid (551 mg, 93%). LC/MS (ESI) m/z for $C_{13}H_9F_3N_2OS$ 299 ([M + H]$^+$, calculated) 299 ([M + H]$^+$, found).

Step-4: methyl 5-(difluoromethoxy)-*N*-(2-fluorophenyl)pyridine-2-carbimidothioate was prepared according to the General Procedure **C** as a yellow oil (569 mg, 100%). LC/MS (ESI) m/z for $C_{14}H_{11}F_3N_2OS$ 313 ([M + H]$^+$, calculated) 313 ([M + H]$^+$, found).

Step-5: *tert*-butyl (*trans*-3-(5-(5-(difluoromethoxy)pyridin-2-yl)-4-(2-fluorophenyl)-4H-1,2,4-triazol-3-yl)cyclobutyl)carbamate was prepared according to the General Procedure **D** as an off-white foam (448 mg, 91%). LC/MS (ESI) m/z for $C_{23}H_{24}F_3N_5O_3$ 476 ([M + H]$^+$, calculated) 476 ([M + H]$^+$, found).

Step-6: The title compound was prepared following to the General Procedure **E** and obtained as a white solid (447 mg, 100%). LC/MS (ESI) m/z for $C_{18}H_{16}F_3N_5O$ 376 ([M + H]$^+$, calculated) 376 ([M + H]$^+$, found).

Preparation of *trans*-3-(4-(2-fluorophenyl)-5-(pyridin-2-yl)-4H-1,2,4-triazol-3-yl)cyclobutan-1-amine dihydrochloride, intermediate for final products 30a and 30b.

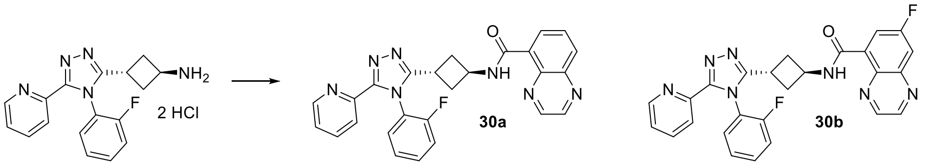

Step-1: *N*-(2-fluorophenyl)picolinamide was prepared following the General Procedure **A**, method a) as an off-white solid (825 mg, 76%). LC/MS (ESI) m/z for $C_{12}H_9FN_2O$ 217 ([M + H]$^+$, calculated) 217 ([M + H]$^+$, found).

Step-2: *N*-(2-fluorophenyl)pyridine-2-carbothioamide was prepared according to the General Procedure **B** as a yellow solid (792 mg, 89%). LC/MS (ESI) m/z for $C_{12}H_9FN_2S$ 233 ([M + H]$^+$, calculated) 233 ([M + H]$^+$, found).

Step-3: methyl *N*-(2-fluorophenyl)pyridine-2-carbimidothioate was prepared according to the General Procedure **C** as a yellow oil (809 mg, 97%). LC/MS (ESI) m/z for $C_{13}H_{11}FN_2S$ 247 ([M + H]$^+$, calculated) 247 ([M + H]$^+$, found).

Step-4: *tert*-butyl (*trans*-3-(4-(2-fluorophenyl)-5-(pyridin-2-yl)-4H-1,2,4-triazol-3-yl)cyclobutyl)carbamate was prepared according to the General Procedure **D** as a white solid (653 mg, 77%). LC/MS (ESI) m/z for $C_{22}H_{24}FN_5O_2$ 410 ([M + H]$^+$, calculated) 410 ([M + H]$^+$, found).

Step-5: The title compound was prepared following to the General Procedure **E** and obtained as a white solid (661 mg, 100%). LC/MS (ESI) m/z for $C_{17}H_{16}FN_5$ 310 ([M + H]$^+$, calculated) 310 ([M + H]$^+$, found).

Preparation of *trans*-3-(4-(2-fluorophenyl)-5-(6-methylpyridin-2-yl)-4H-1,2,4-triazol-3-yl)cyclobutan-1-amine dihydrochloride, intermediate for final products 31a and 31b.

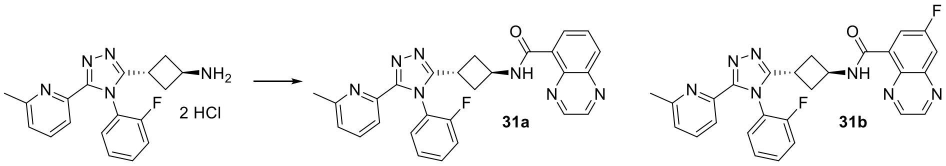

Step-1: *N*-(2-fluorophenyl)-6-methylpicolinamide was prepared following the General Procedure **A**, method a) as an off-white solid (519 mg, 89%). LC/MS (ESI) m/z for $C_{13}H_{11}FN_2O$ 231 ([M + H]$^+$, calculated) 231 ([M + H]$^+$, found).

Step-2: *N*-(2-fluorophenyl)-6-methylpyridine-2-carbothioamide was prepared according to the General Procedure **B** as a yellow solid (475 mg, 86%). LC/MS (ESI) m/z for $C_{13}H_{11}FN_2S$ 247 ([M + H]$^+$, calculated) 247 ([M + H]$^+$, found).

Step-3: methyl *N*-(2-fluorophenyl)-6-methylpyridine-2-carbimidothioate was prepared according to the General Procedure **C** as a yellow oil (492 mg, 99%). LC/MS (ESI) m/z for $C_{14}H_{13}FN_2S$ 260 ([M + H]$^+$, calculated) 260 ([M + H]$^+$, found).

Step-4: *tert*-butyl (*trans*-3-(4-(2-fluorophenyl)-5-(6-methylpyridin-2-yl)-4H-1,2,4-triazol-3-yl)cyclobutyl)carbamate was prepared according to the General Procedure **D** as a white solid (405 mg, 93%). LC/MS (ESI) m/z for $C_{23}H_{26}FN_5O_2$ 424 ($[M + H]^+$, calculated) 424 ($[M + H]^+$, found).

Step-5: The title compound was prepared following to the General Procedure **E** and obtained as a white solid (396 mg, 100%). LC/MS (ESI) m/z for $C_{18}H_{18}FN_5$ 324 ($[M + H]^+$, calculated) 324 ($[M + H]^+$, found).

NMR and LCMS spectra of final products

For spectra of final products **1** – **6**, **15a** and **15b** see the Supplementary Experimentals published earlier.[1]

Compound 7

title	**PINI22-65-1**
Method	AN_ACID.M
Date acquired	08-Jun-18, 17:11:18
FileName	Analysis\LCMS6_0608_106.D
Column	XSelect CSH C18 (50x2.1mm, 3.5μ) valve:3
Flow	0.8 ml/min; Column temp: 35°C
Eluent A	0.1% formic acid in acetonitrile
Eluent B	0.1% formic acid in water
Gradient	t=0 min 5% A, t=3.5 min 98% A, t=6 min 98%A
Posttime	2 min
Detection	DAD(210, 220 and 220-320nm)
Detection	PDA(210-320nm)
Detection	MSD (ESI pos/neg) mass range: 100 - 1000
Detection	ELSD gas temp: 40°C, flow 1.5 ml/min, gain 1

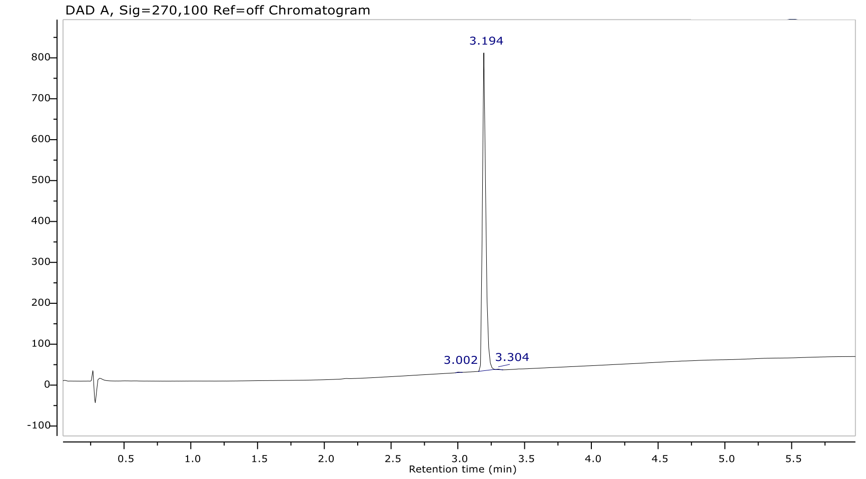

Integrals spectrum Chromatogram DAD A, Sig=270,100 Ref=off

rt (min)	height	area	area (%)
3.00	1.198	0.02797	0.12
3.19	777.0	22.84	99.75
3.30	1.097	0.02833	0.12

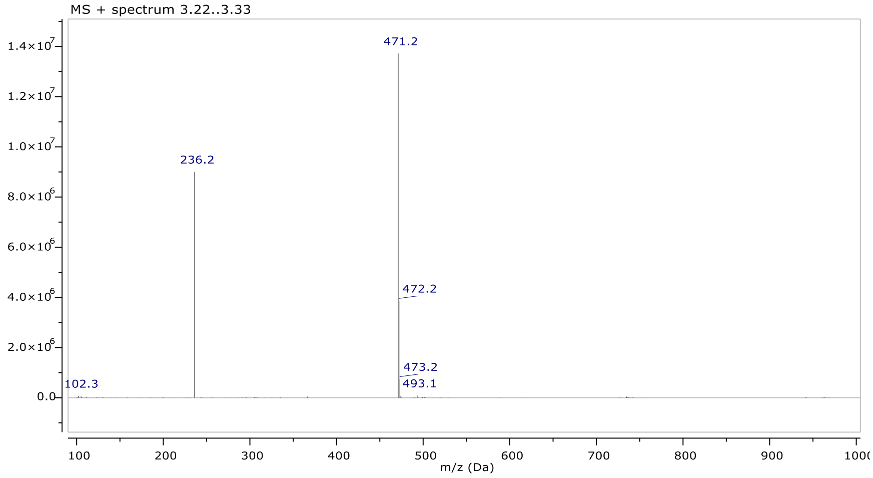

Title	**PINI22-65-1**
Data File Name	0611_50
Origin	Bruker BioSpin GmbH
Method	
Pulse Sequence	zg30
Relaxation Delay	1 s
Solvent	CDCl3
Acquisition Date	2018-06-11T08:48:54
Temperature	~ 298 K
Number of Scans	32
Frequency	400.232471584084 MHz
Nucleus	1H

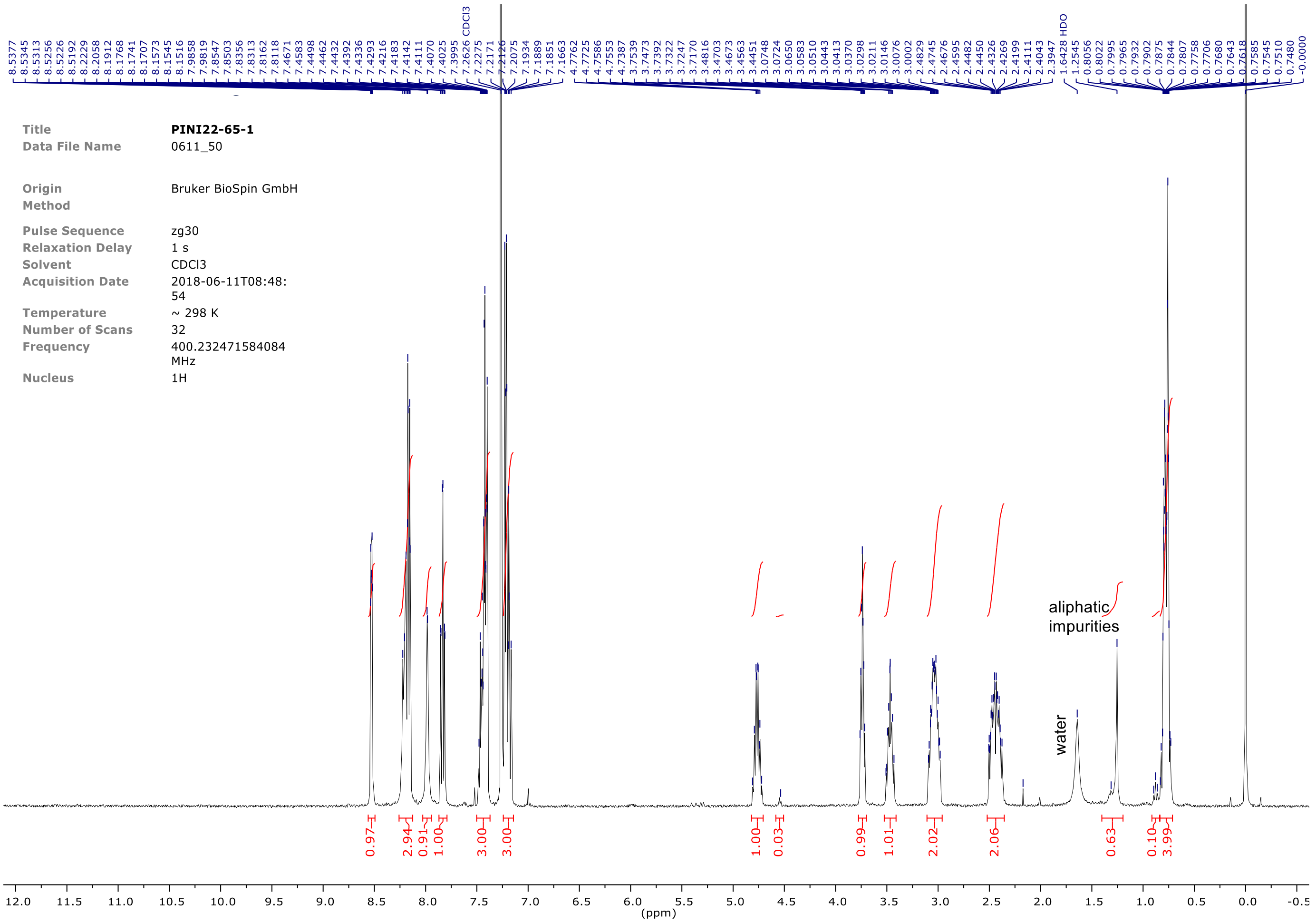

HRMS (m/z): C26H23N6O2F, [M+H]+ Calc: 471.19393; found: 471.1929, Δppm -2.28

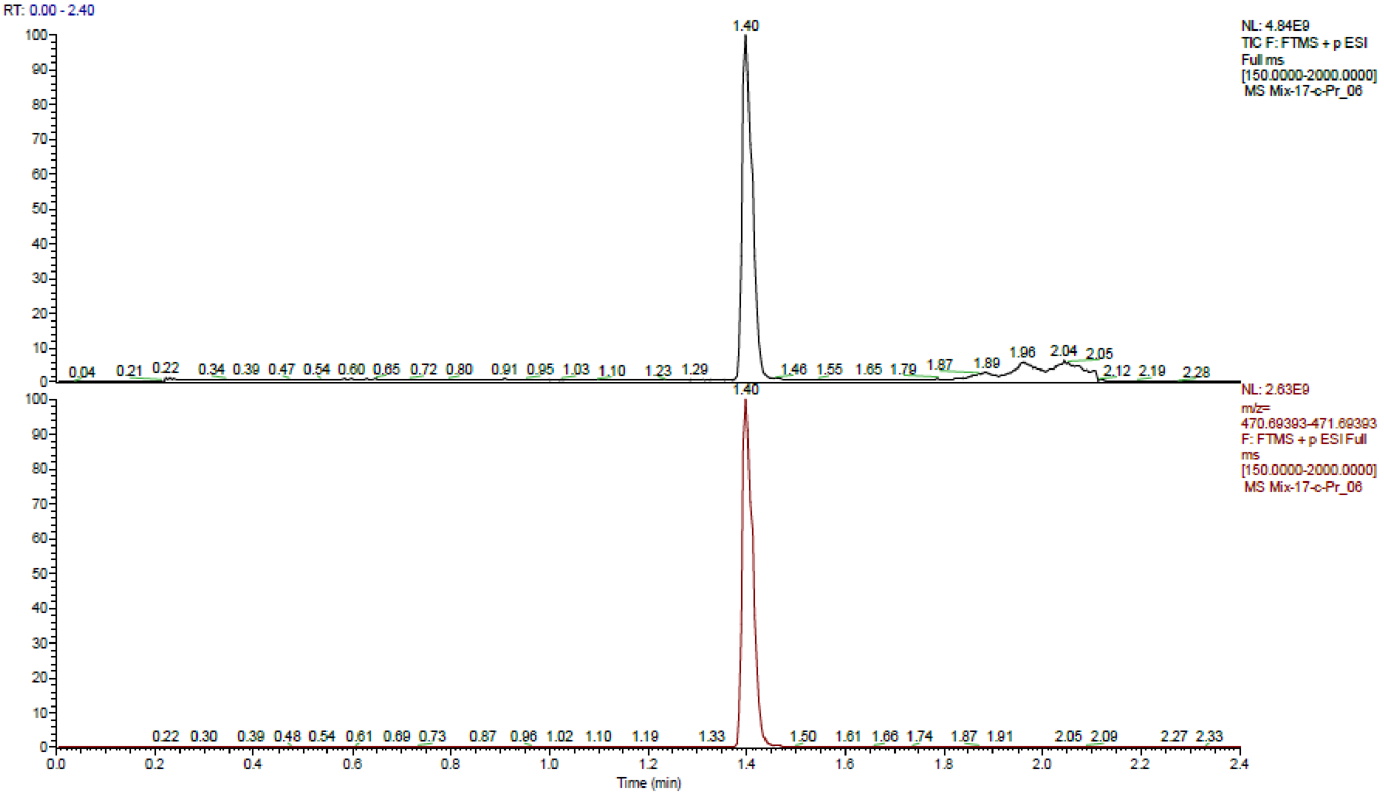

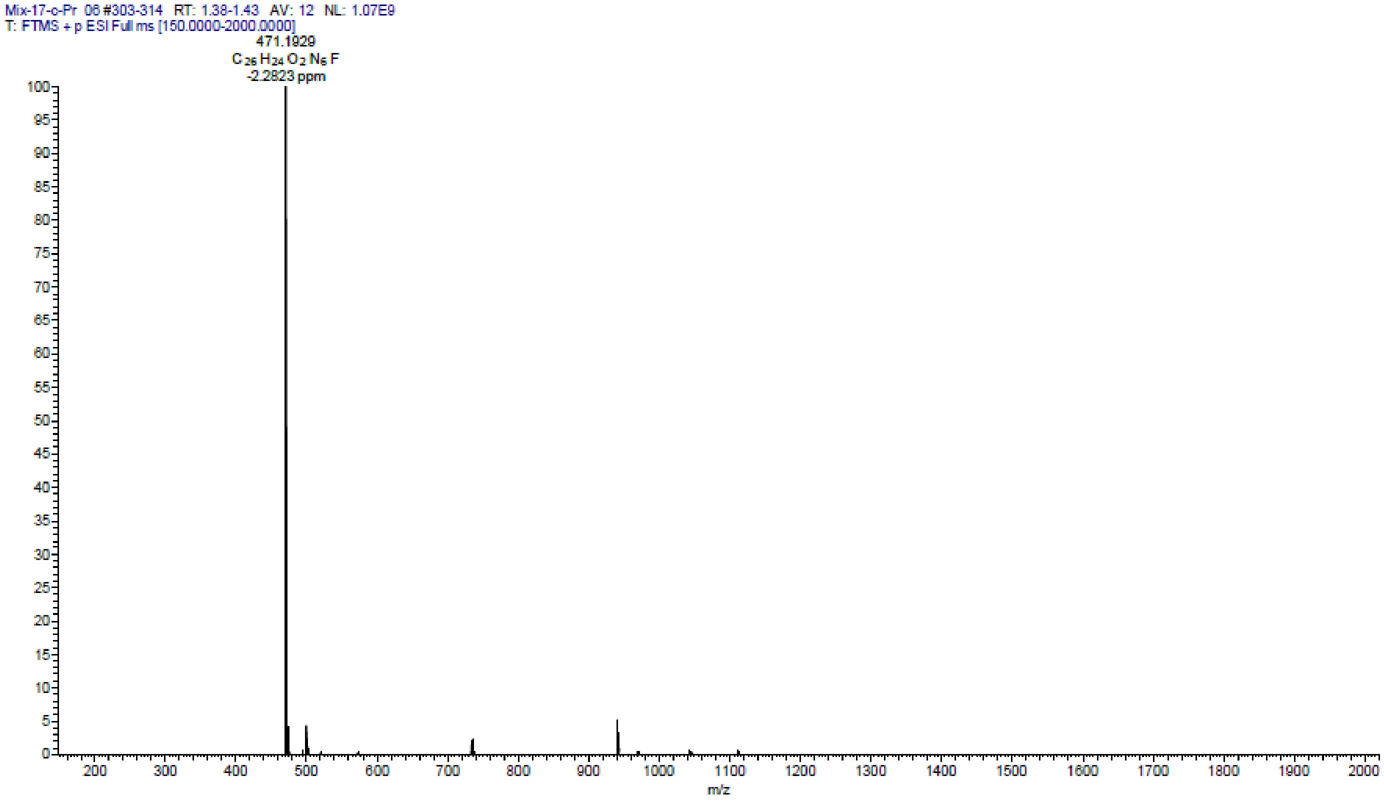

Compound 8

**title**	**PINI22-04-1**
**Method**	AN_ACID.M
**Date acquired**	19-Mar-18, 16:57:25
**FileName**	Analysis\LCMS6_0319_079.D
**Column**	XSelect CSH C18 (50x2.1mm, 3.5µ) valve:3
**Flow**	0.8 ml/min; Column temp: 35°C
**Eluent A**	0.1% Formic acid in acetonitrile
**Eluent B**	0.1% Formic acid in water
**Gradient**	t=0 min 5% A, t=3.5 min 98% A, t=6 min 98% A
**Posttime**	2 min
**Detection**	DAD (210, 220, 220-320nm)
**Detection**	PDA (210-320nm)
**Detection**	MSD (ESI pos/neg) mass range: 100-1000
**Detection**	ELSD gas temp: 40°C, flow 1.5 ml/min gain 1

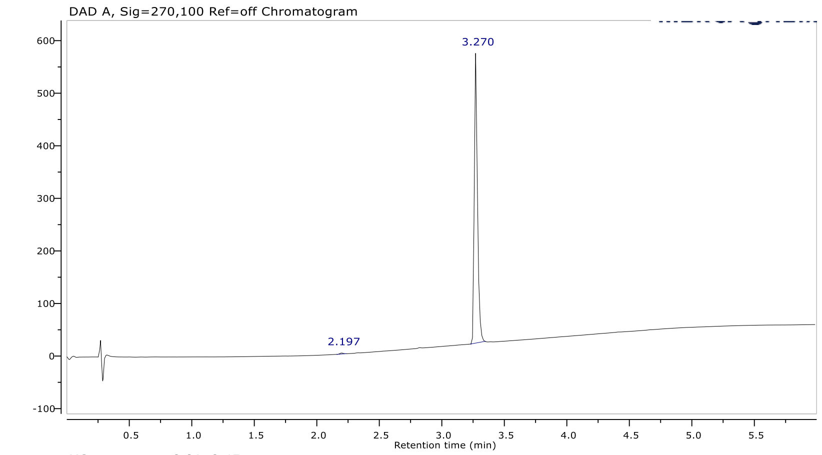

Integrals spectrum Chromatogram DAD A, Sig=270,100 Ref=off

rt (min)	height	area	area (%)
2.20	1.988	0.05947	0.36
3.27	551.4	16.35	99.64

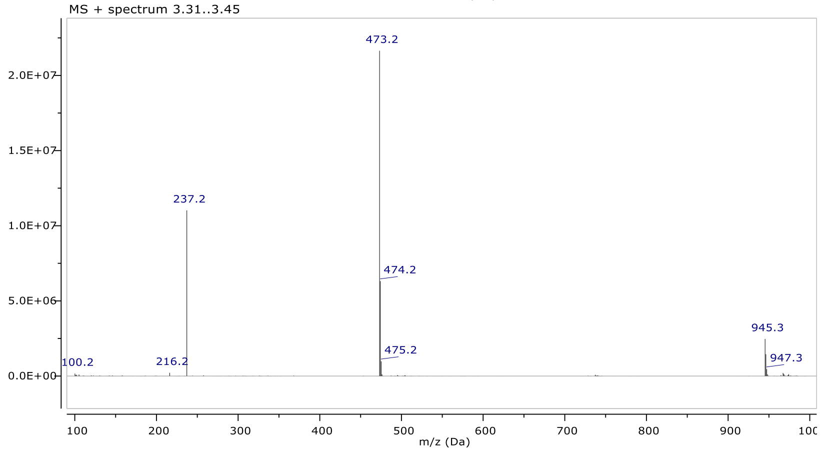

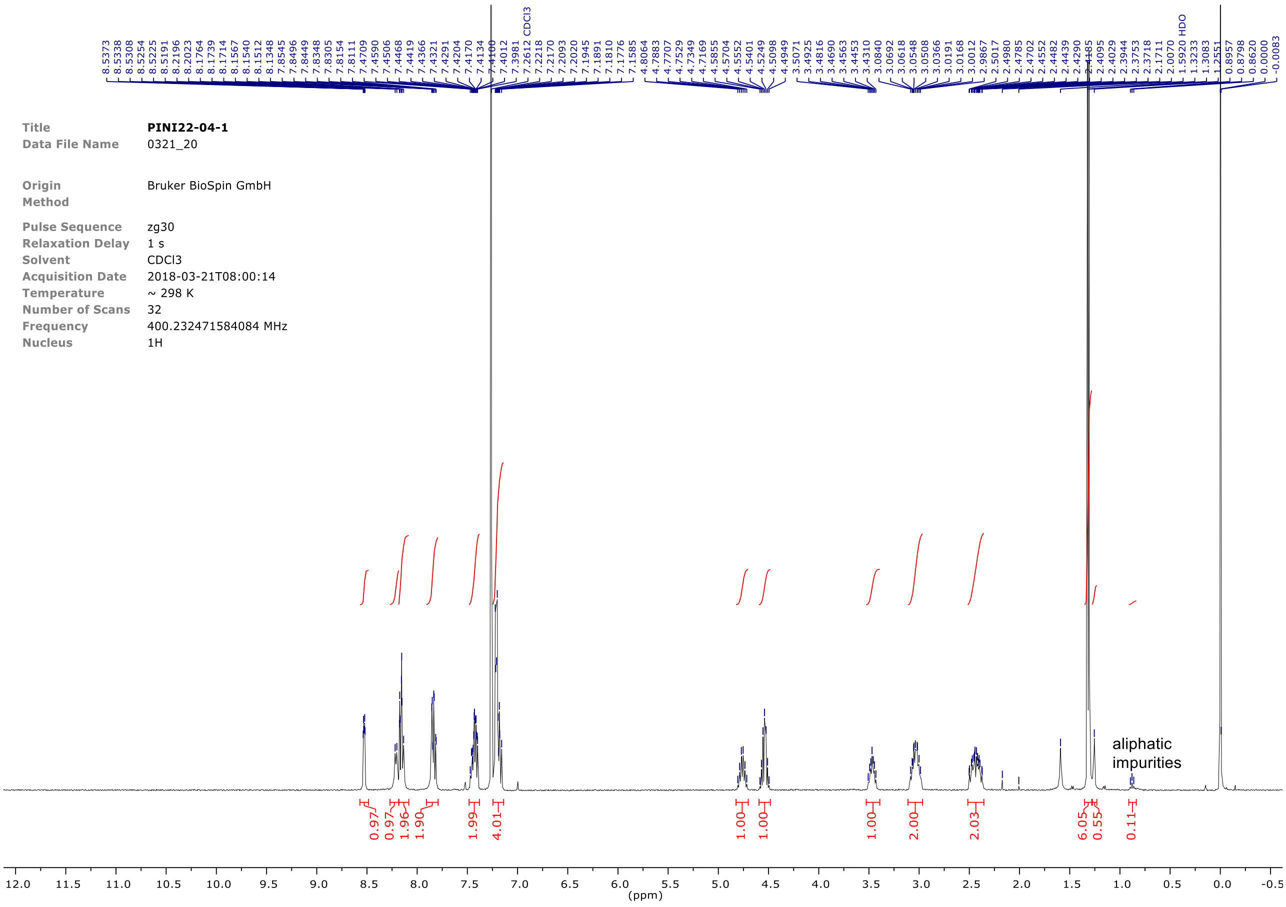

**Title**	**PINI22-04-1**
**Data File Name**	0321_20
**Origin**	Bruker BioSpin GmbH
**Method**	
**Pulse Sequence**	zg30
**Relaxation Delay**	1 s
**Solvent**	CDCl3
**Acquisition Date**	2018-03-21T08:00:14
**Temperature**	~ 298 K
**Number of Scans**	32
**Frequency**	400.232471584084 MHz
**Nucleus**	1H

HRMS (m/z): C26H25N6O2F, [M+H]+ Calc: 473.20958; found: 473.2089, Δppm -1.42

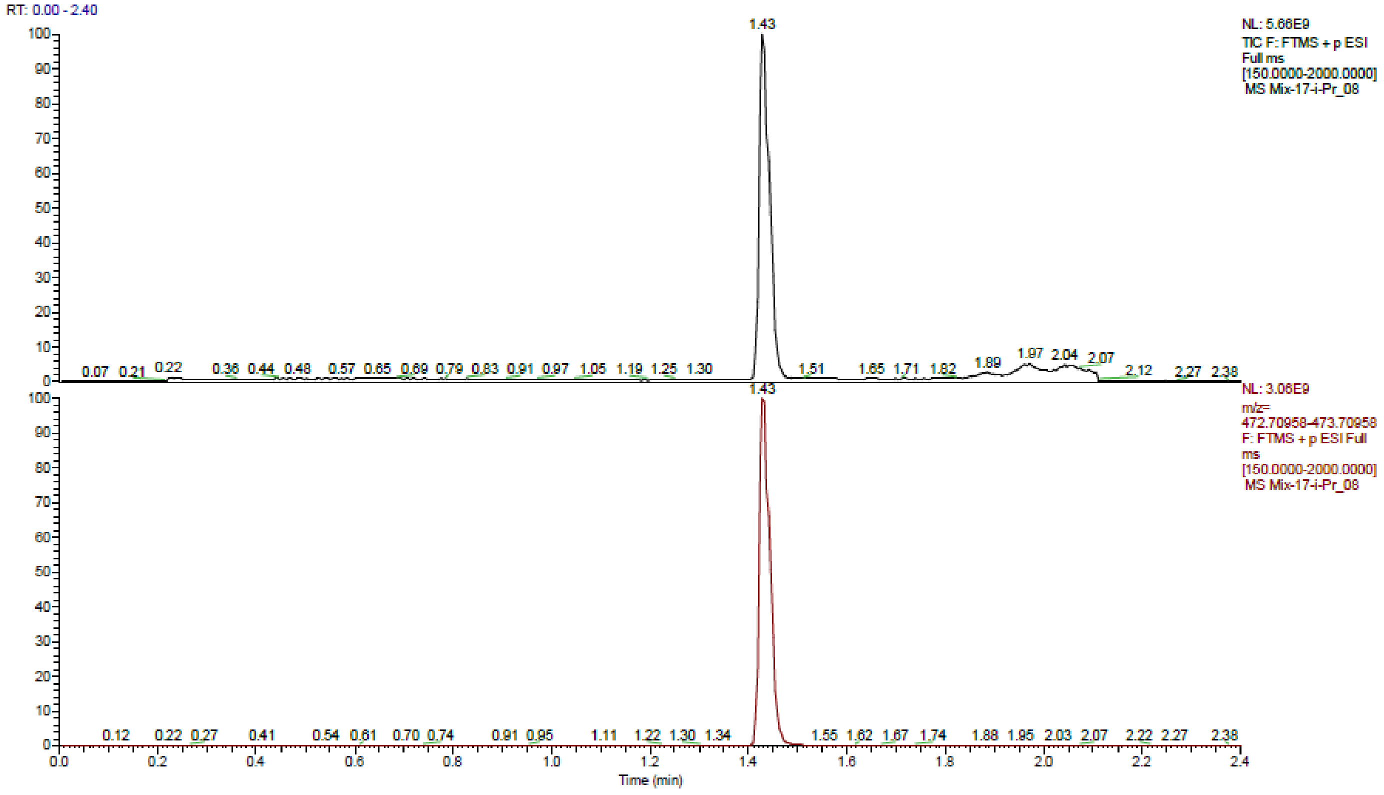

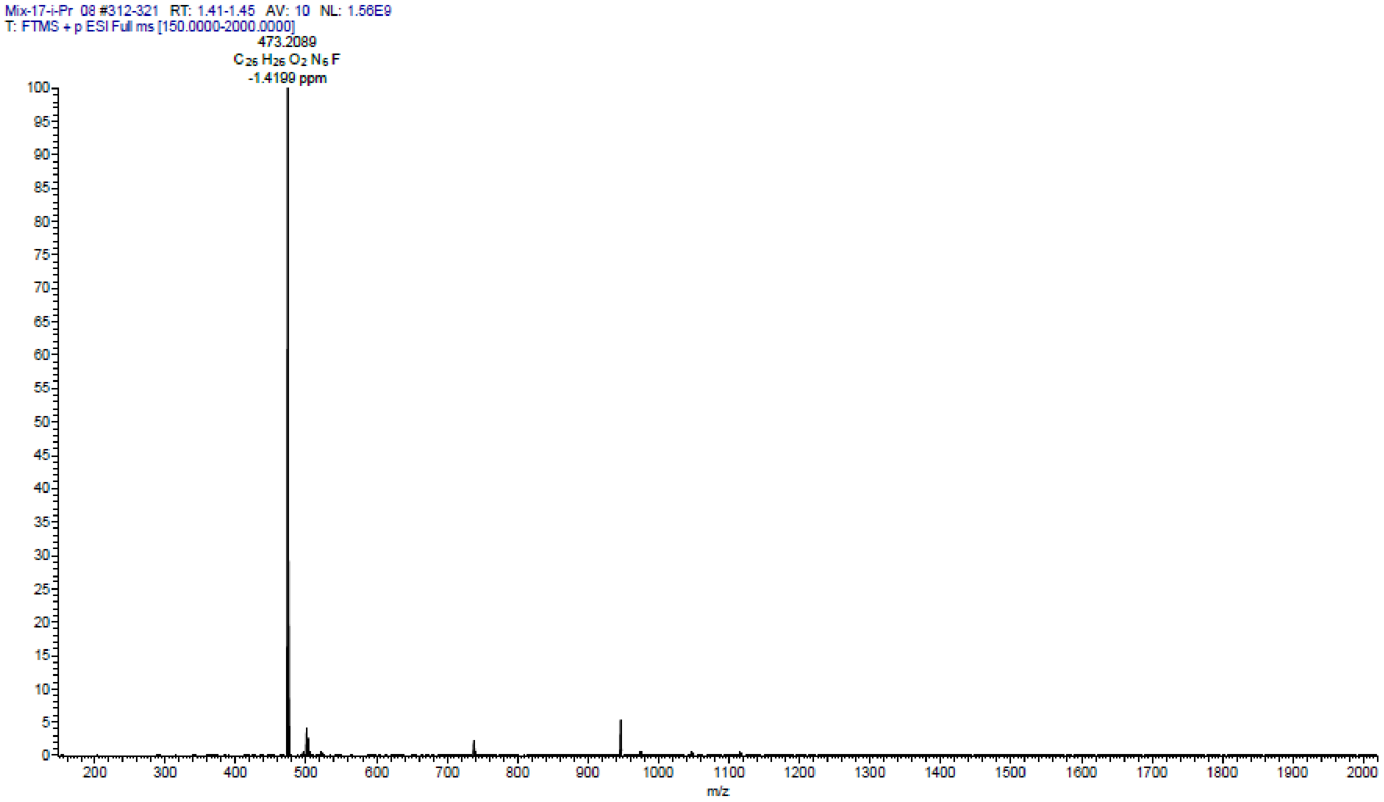

Compound 9

**title**	**PINI23-12-1**
**Method**	AN_ACID.M
**Date acquired**	29-Aug-18, 17:37:04
**FileName**	Analysis\LCMS6_0829_100.D
**Column**	XSelect CSH C18 (50x2.1mm, 3.5μ) valve:3
**Flow**	0.8 ml/min; Column temp: 35°C
**Eluent A**	0.1% formic acid in acetonitrile
**Eluent B**	0.1% formic acid in water
**Gradient**	t=0 min 5% A, t=3.5 min 98% A, t=6 min 98%A
**Posttime**	2 min
**Detection**	DAD(210, 220 and 220-320nm)
**Detection**	PDA(210-320nm)
**Detection**	MSD (ESI pos/neg) mass range: 100 - 1000
**Detection**	ELSD gas temp: 40°C, flow 1.5 ml/min, gain 1

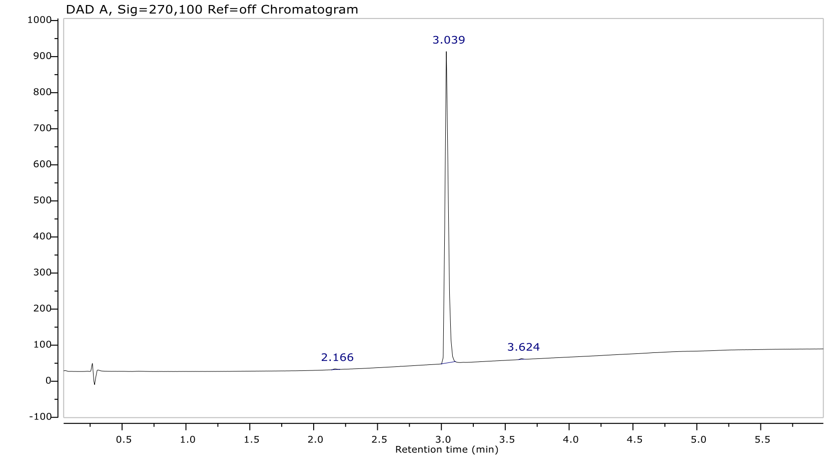

Integrals spectrum Chromatogram DAD A, Sig=270,100 Ref=off

rt (min)	height	area	area (%)
2.17	2.131	0.06417	0.25
3.04	863.9	26.04	99.52
3.62	2.476	0.06064	0.23

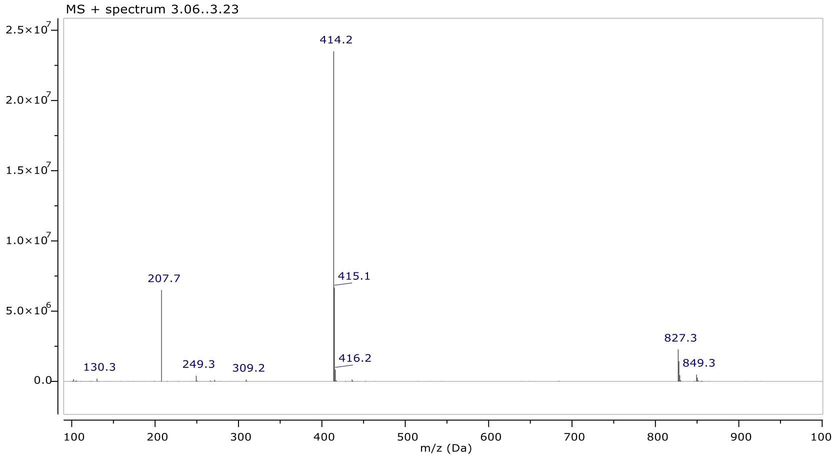

**Title**	**PINI23-12-1**
**Data File Name**	0829_330
**Origin**	Bruker BioSpin GmbH
**Method**	
**Pulse Sequence**	zg30
**Relaxation Delay**	1 s
**Solvent**	CDCl3
**Acquisition Date**	2018-08-29T12:48:01
**Temperature**	~ 295.3 K
**Number of Scans**	32
**Frequency**	400.232471584084 MHz
**Nucleus**	1H

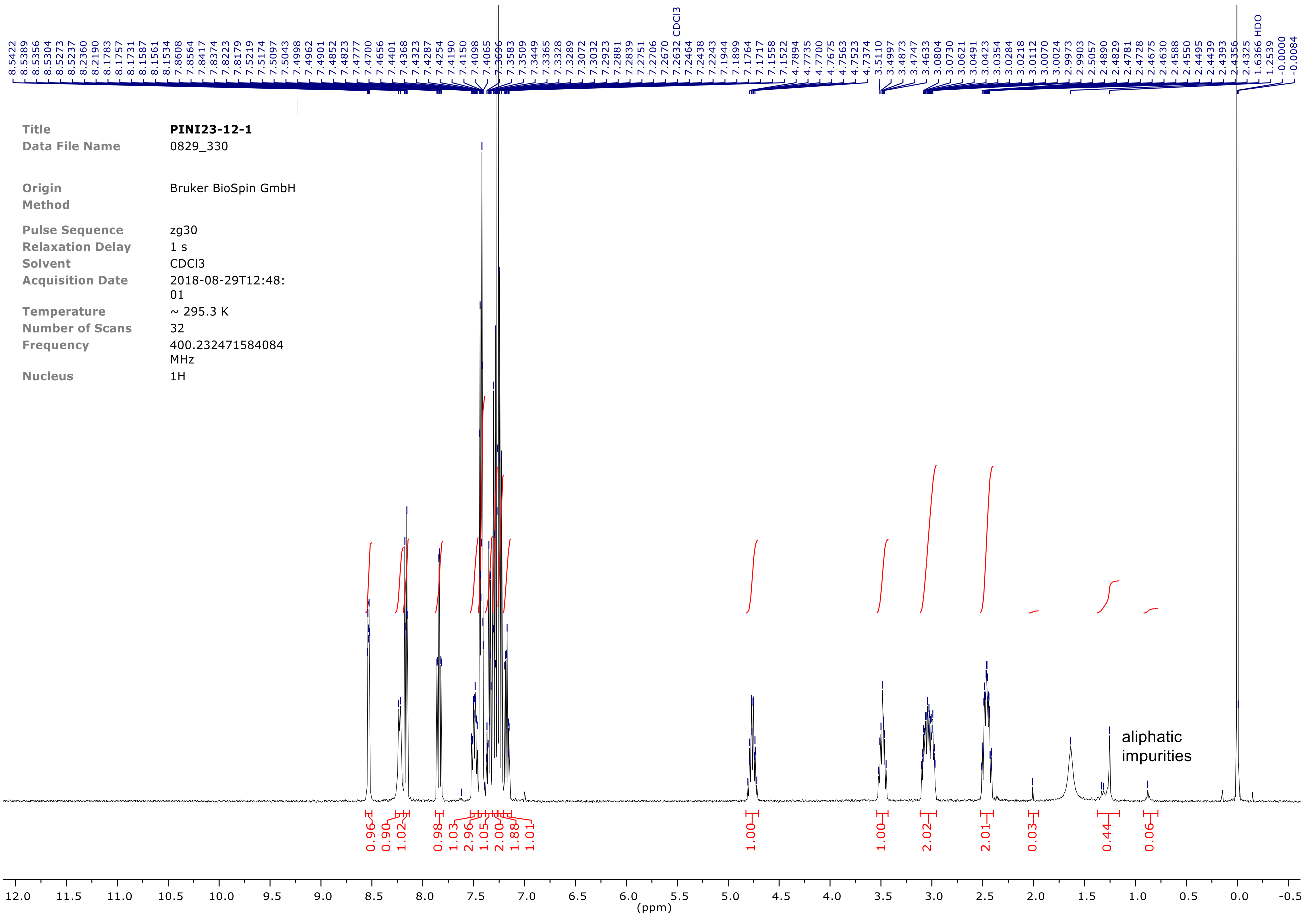

HRMS (m/z): C24H20N5OF, [M+H]+ Calc 414.17246; found: 414.1714, Δppm -2.46

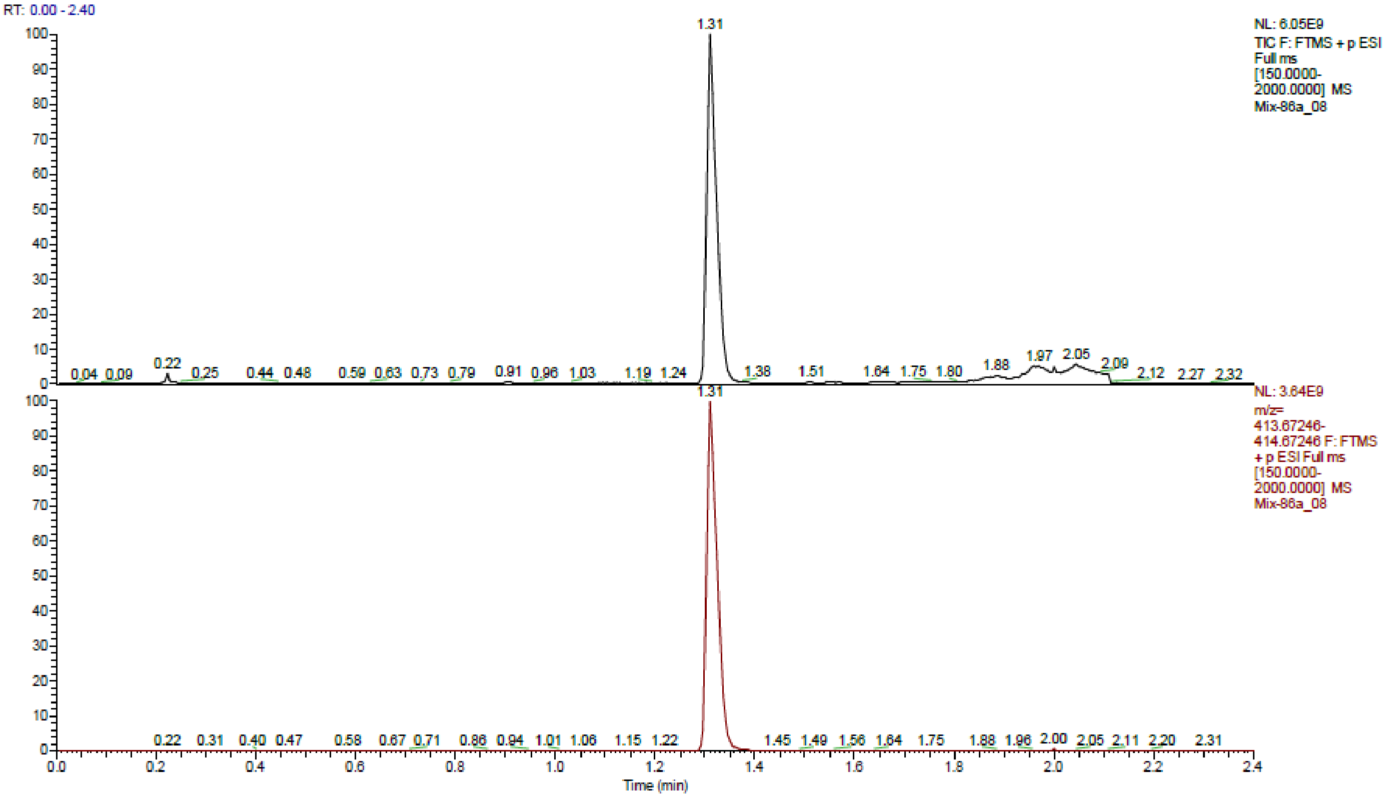

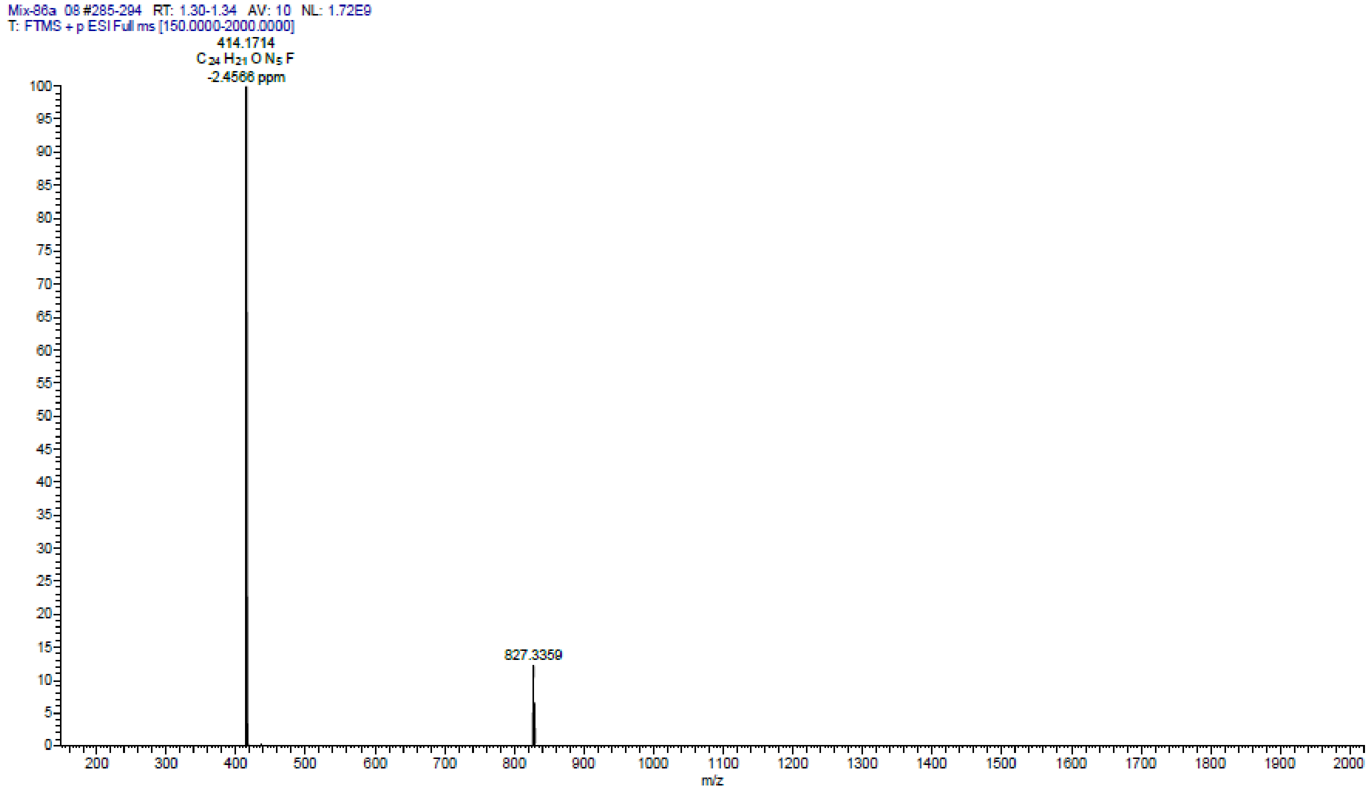

Compound 10

**title**	**sjae 54 204-2**
**Method**	AN_BASE.M
**Date acquired**	27-Nov-17, 17:49:51
**FileName**	Analysis\LCMS5_1127_039.D
**Acq. method**	AN_BASE valve: 2
**Column**	Waters XSelect CSH C18 (50x2.1mm, 3.5µ)
**Flow**	0.8 ml/min; Column temp: 25°C
**Eluent A**	95% acetonitrile + 5% 10 mM ammoniumbicarbonate in water
**Eluent B**	10mM ammoniumbicarbonate in water
**Lin. Gradient**	t=0 min 5% A, t=3.5 min 98% A, t=6 min 98% A
**Posttime**	2 min
**Detection**	DAD (210, 220 and 220-320 nm)
**Detection**	PDA (210-320 nm)
**Detection**	MSD (ESI pos/neg) mass range: 100-800

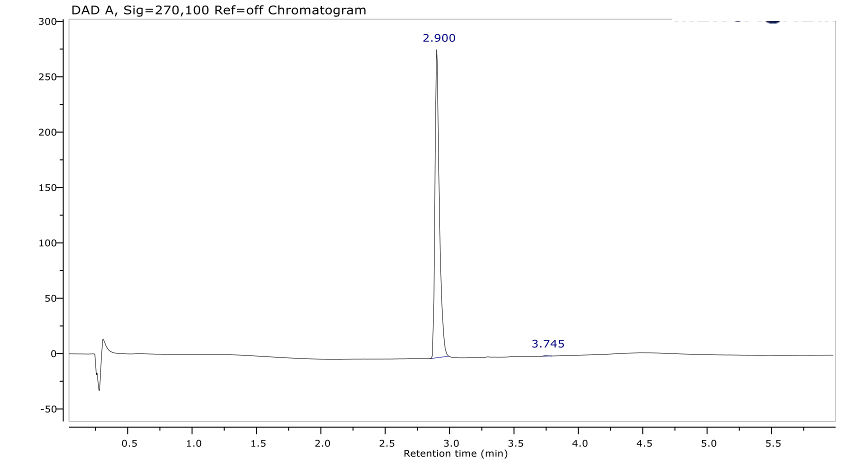

Integrals spectrum **Chromatogram DAD A, Sig=270,100 Ref=off**

rt (min)	height	area	area (%)
2.90	278.2	10.97	99.81
3.75	0.6238	0.02094	0.19

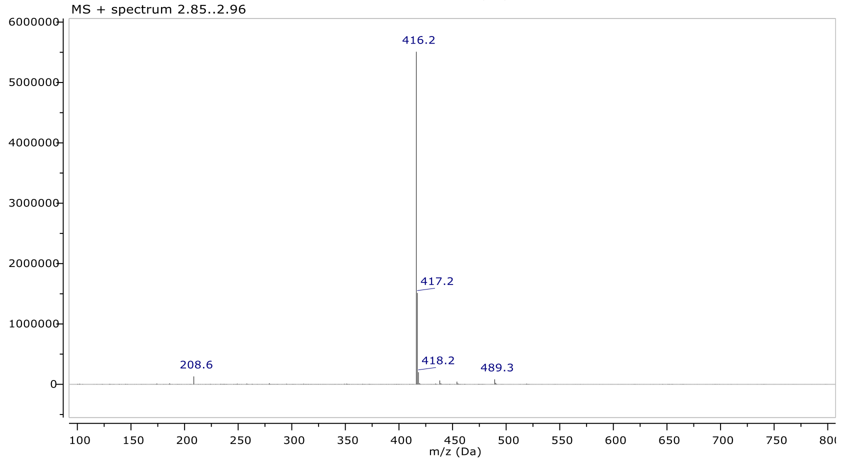

**Title**	**sjae 54 204-2**
**Data File Name**	1129_80
**Origin**	Bruker BioSpin GmbH
**Method**	
**Pulse Sequence**	zg30
**Relaxation Delay**	1 s
**Solvent**	CDCl3
**Acquisition Date**	2017-11-29T11:21:26
**Temperature**	~ 298 K
**Number of Scans**	64
**Frequency**	400.232471584084 MHz
**Nucleus**	1H

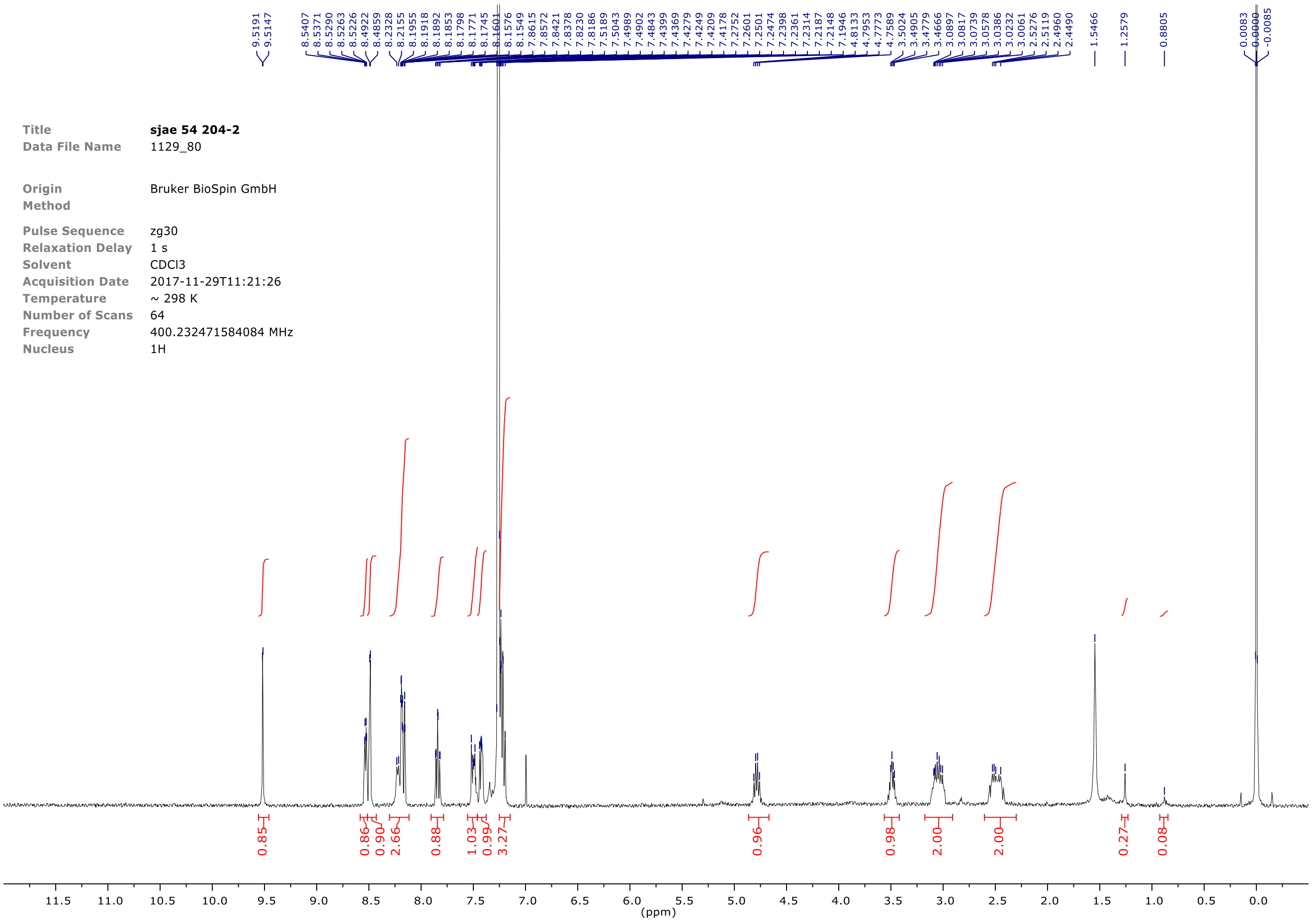

HRMS (m/z): C22H18N7OF, [M+H]+ Calc: 416.16296; found: 416.1620, Δppm -2.40

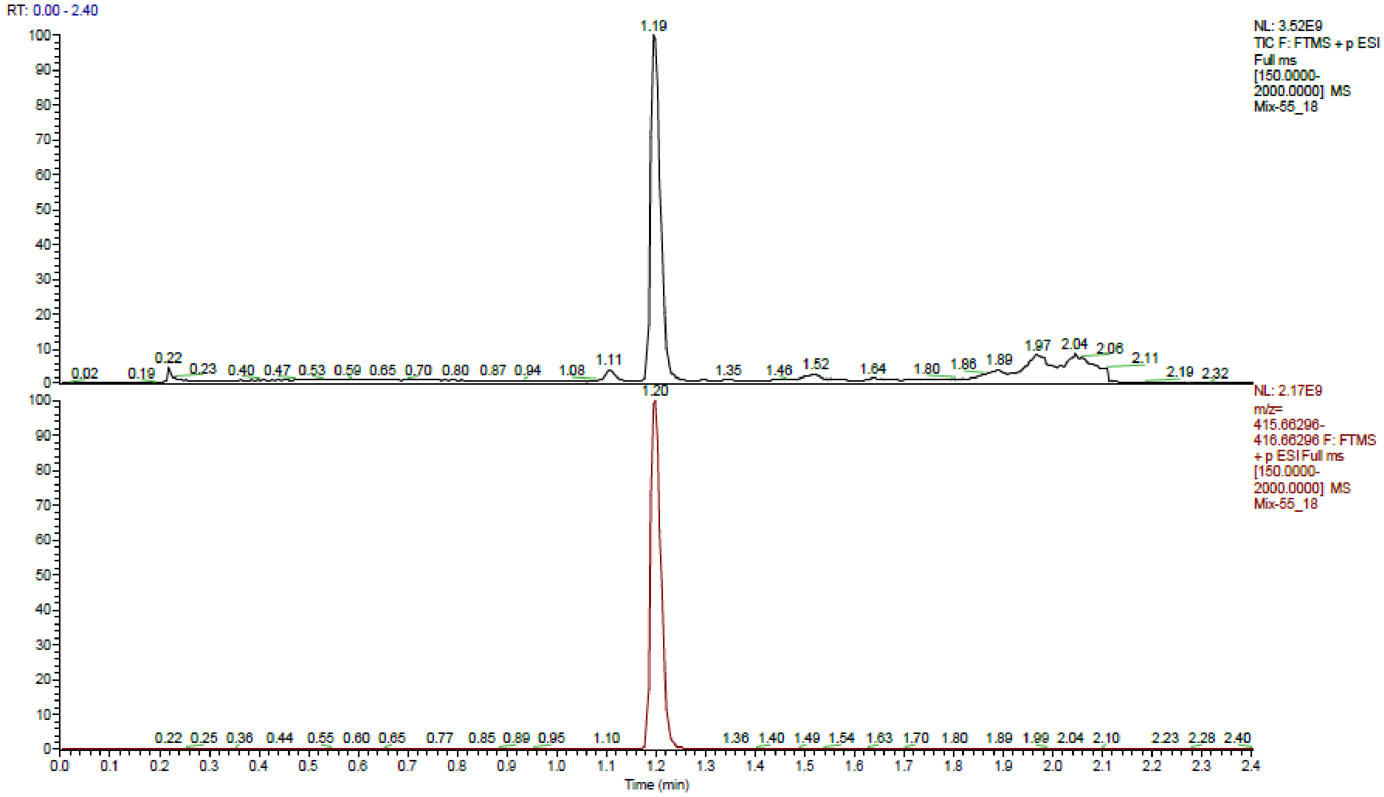

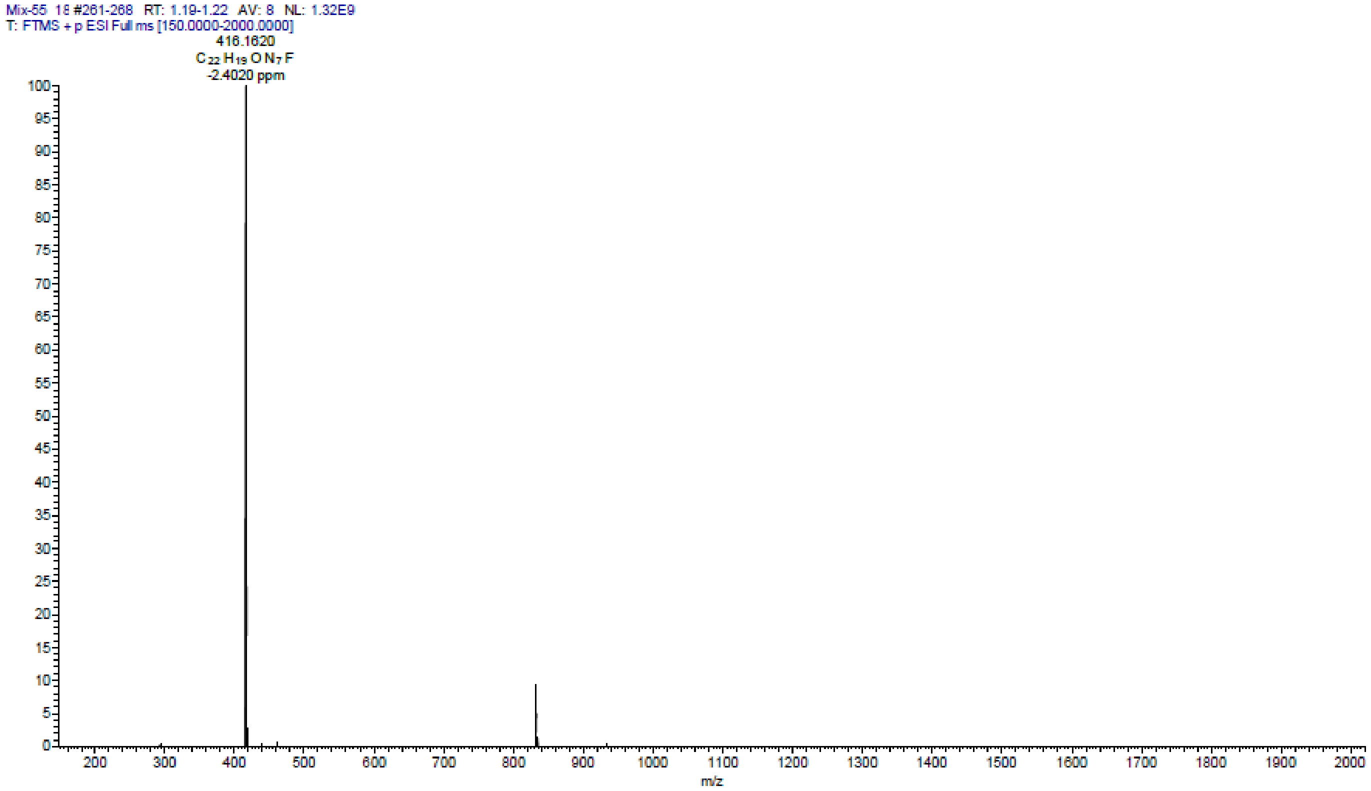

Compound 11

**title**	**sjae 54 172-3**
**Method**	AN_BASE.M
**Date acquired**	03-Nov-17, 16:28:52
**FileName**	Analysis\LCMS5_1103_053.D
**Acq. method**	AN_BASE valve: 2
**Column**	Waters XSelect CSH C18 (50x2.1mm, 3.5μ)
**Flow**	0.8 ml/min; Column temp: 25°C
**Eluent A**	95% acetonitrile + 5% 10 mM ammoniumbicarbonate in water
**Eluent B**	10mM ammoniumbicarbonate in water
**Lin. Gradient**	t=0 min 5% A, t=3.5 min 98% A, t=6 min 98% A
**Posttime**	2 min
**Detection**	DAD (220-320 nm)
**Detection**	PDA (200-400 nm)
**Detection**	MSD (ESI pos/neg) mass range: 100-800

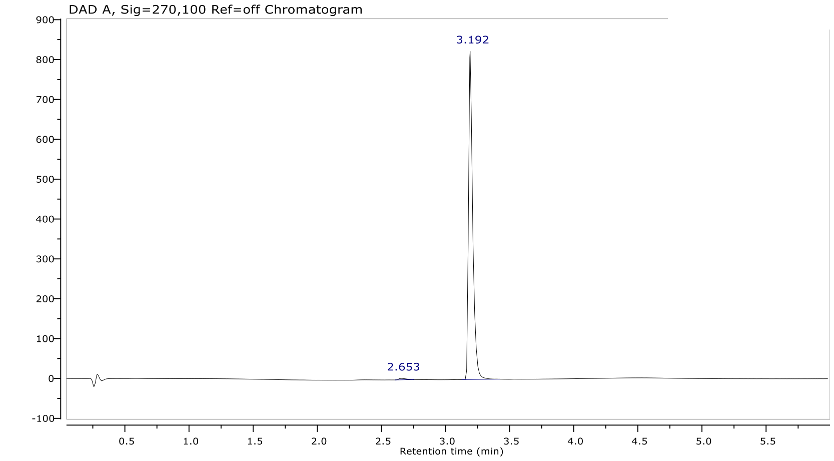

Integrals spectrum Chromatogram DAD A, Sig=270,100 Ref=off

rt (min)	height	area	area (%)
2.65	3.445	0.2334	0.74
3.19	823.3	31.22	99.26

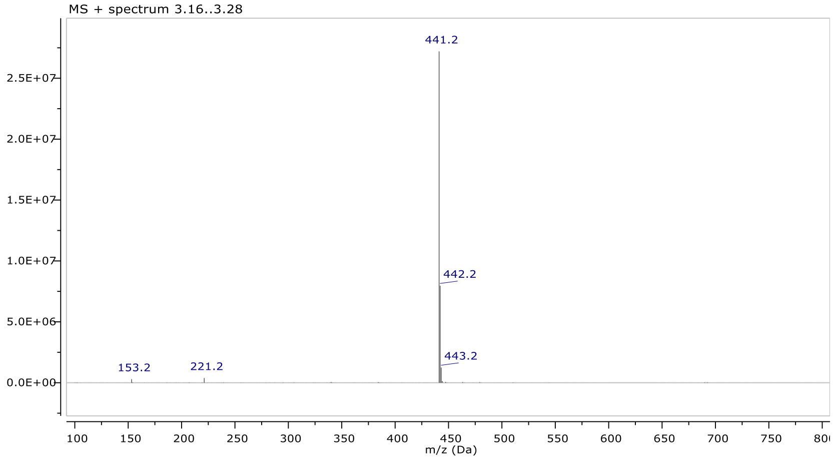

**Title**	**sjae 54 172-3**
**Data File Name**	1102_440
**Origin**	Bruker BioSpin GmbH
**Method**	
**Pulse Sequence**	zg30
**Relaxation Delay**	1 s
**Solvent**	CDCl3
**Acquisition Date**	2017-11-02T17:00:40
**Temperature**	~ 293.2 K
**Number of Scans**	64
**Frequency**	400.232471584084 MHz
**Nucleus**	1H

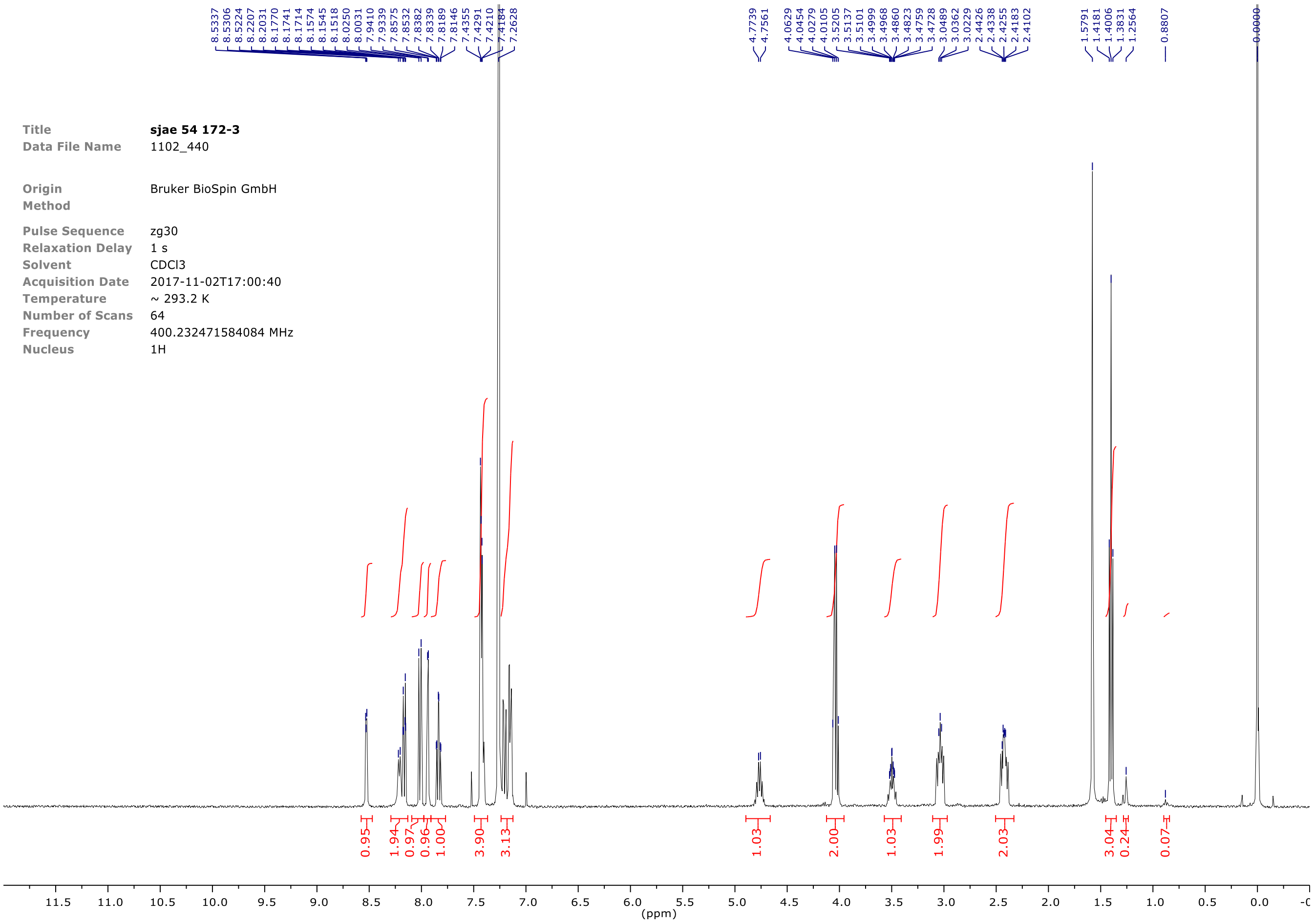

HRMS (m/z): $C_{25}H_{24}N_6O_2$, [M+H]+ Calc: 441.20335; found: 441.2025, Δppm -1.86

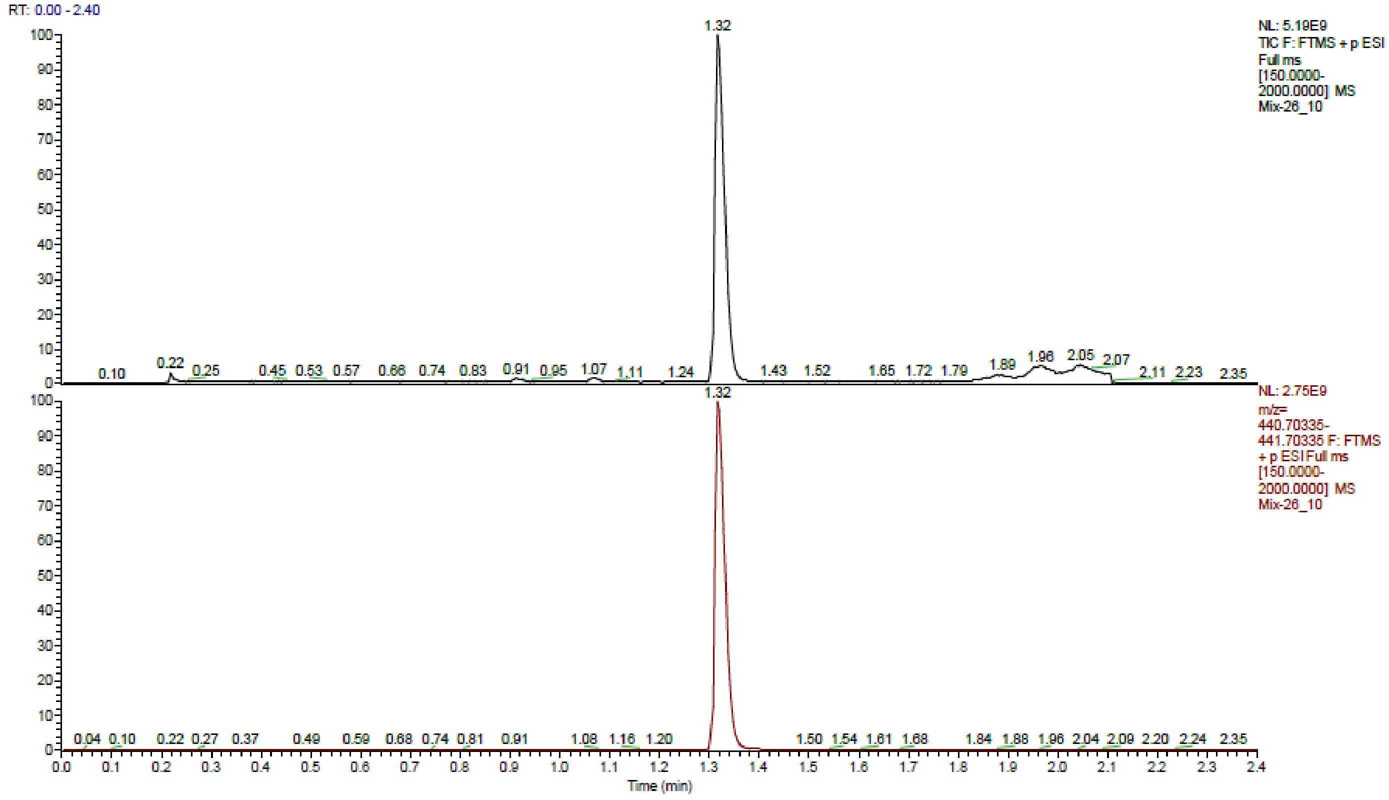

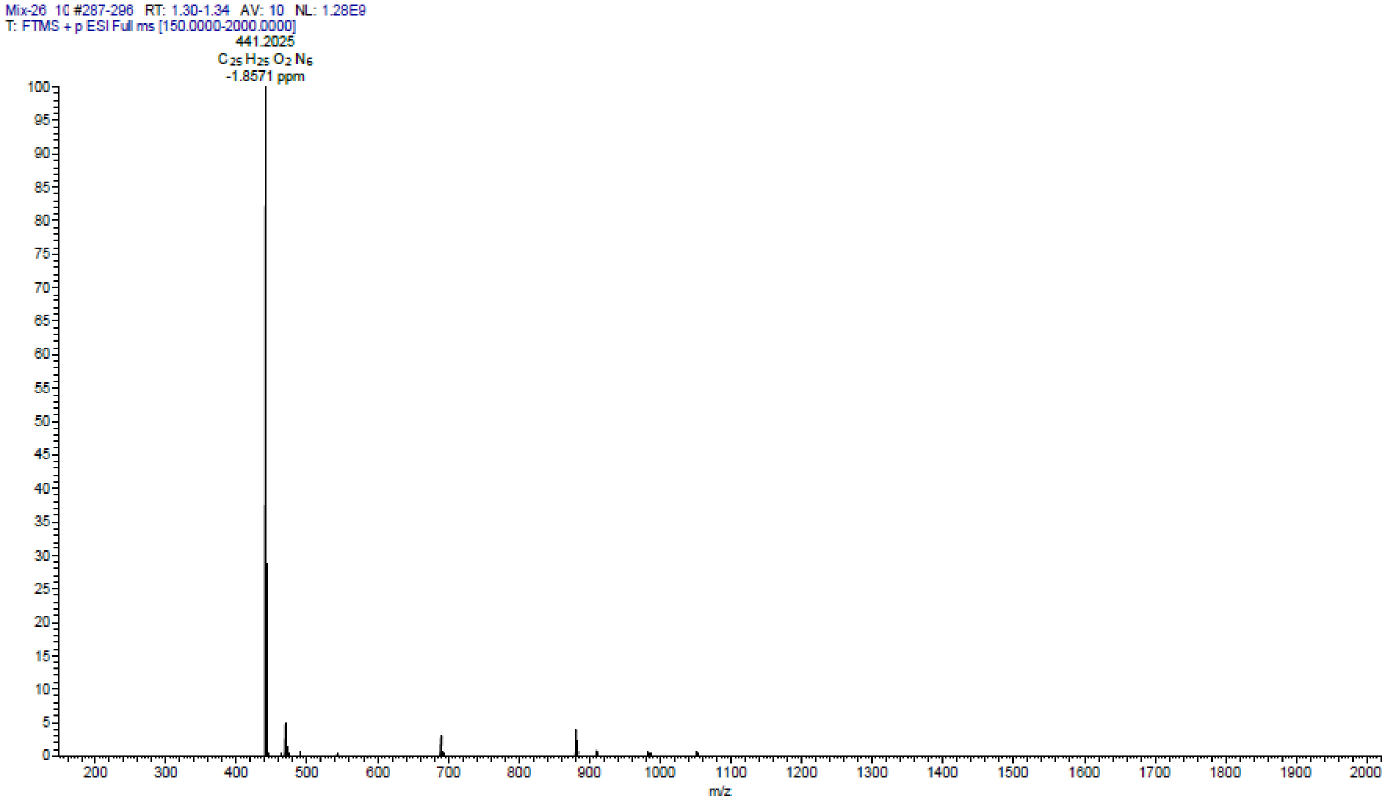

Compound 12

title	**PINI21-74-1**
Method	AN_ACID.M
Date acquired	07-Feb-18, 19:06:26
FileName	Analysis\LCMS6_0207_129.D
Column	XSelect CSH C18 (50x2.1mm, 3.5µ) valve:3
Flow	0.8 ml/min; Column temp: 35°C
Eluent A	0.1% Formic acid in acetonitrile
Eluent B	0.1% Formic acid in water
Gradient	t=0 min 5% A, t=3.5 min 98% A, t=6 min 98% A
Posttime	2 min
Detection	DAD (210, 220, 220-320nm)
Detection	PDA (210-320nm)
Detection	MSD (ESI pos/neg) mass range: 100-800
Detection	CAD Temp. 40°C, iontrap 20.2 V, chargervoltage 2.64 kV

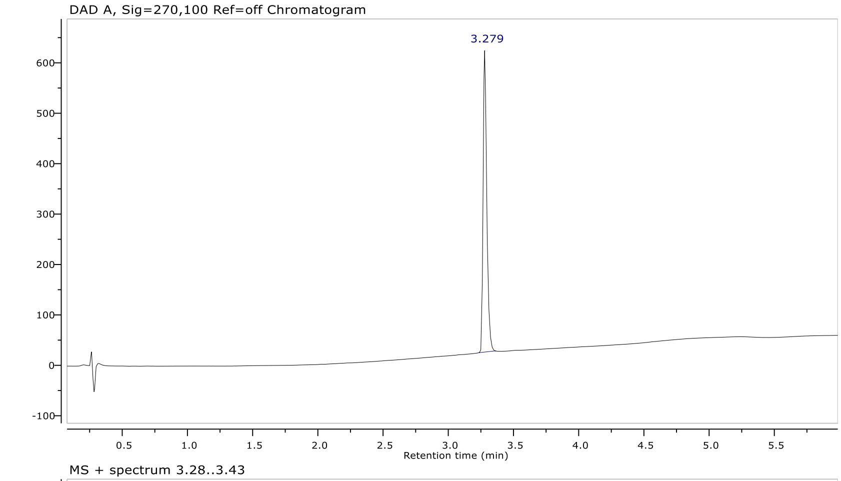

Integrals spectrum Chromatogram DAD A, Sig=270,100 Ref=off

rt (min)	height	area	area (%)
3.28	598.3	19.37	100.00

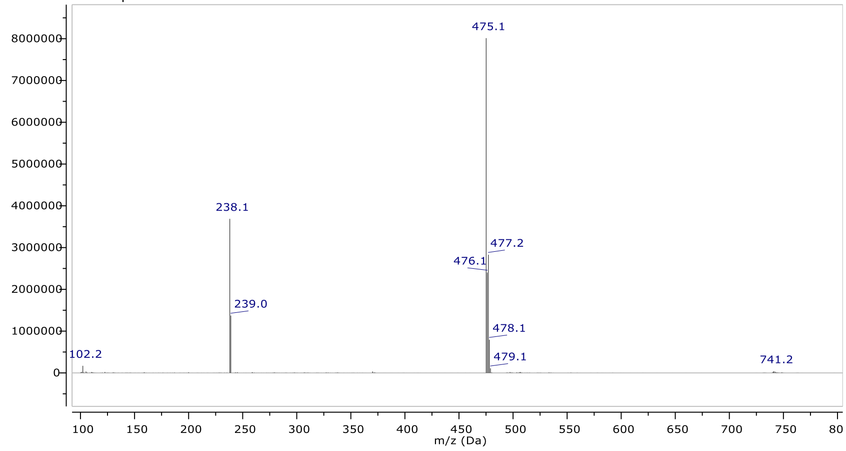

Title	**PINI21-74-1**
Data File Name	0208_230
Origin	Bruker BioSpin GmbH
Method	
Pulse Sequence	zg30
Relaxation Delay	1 s
Solvent	CDCl3
Acquisition Date	2018-02-08T11:32:37
Temperature	~ 298 K
Number of Scans	64
Frequency	400.232471584084 MHz
Nucleus	1H

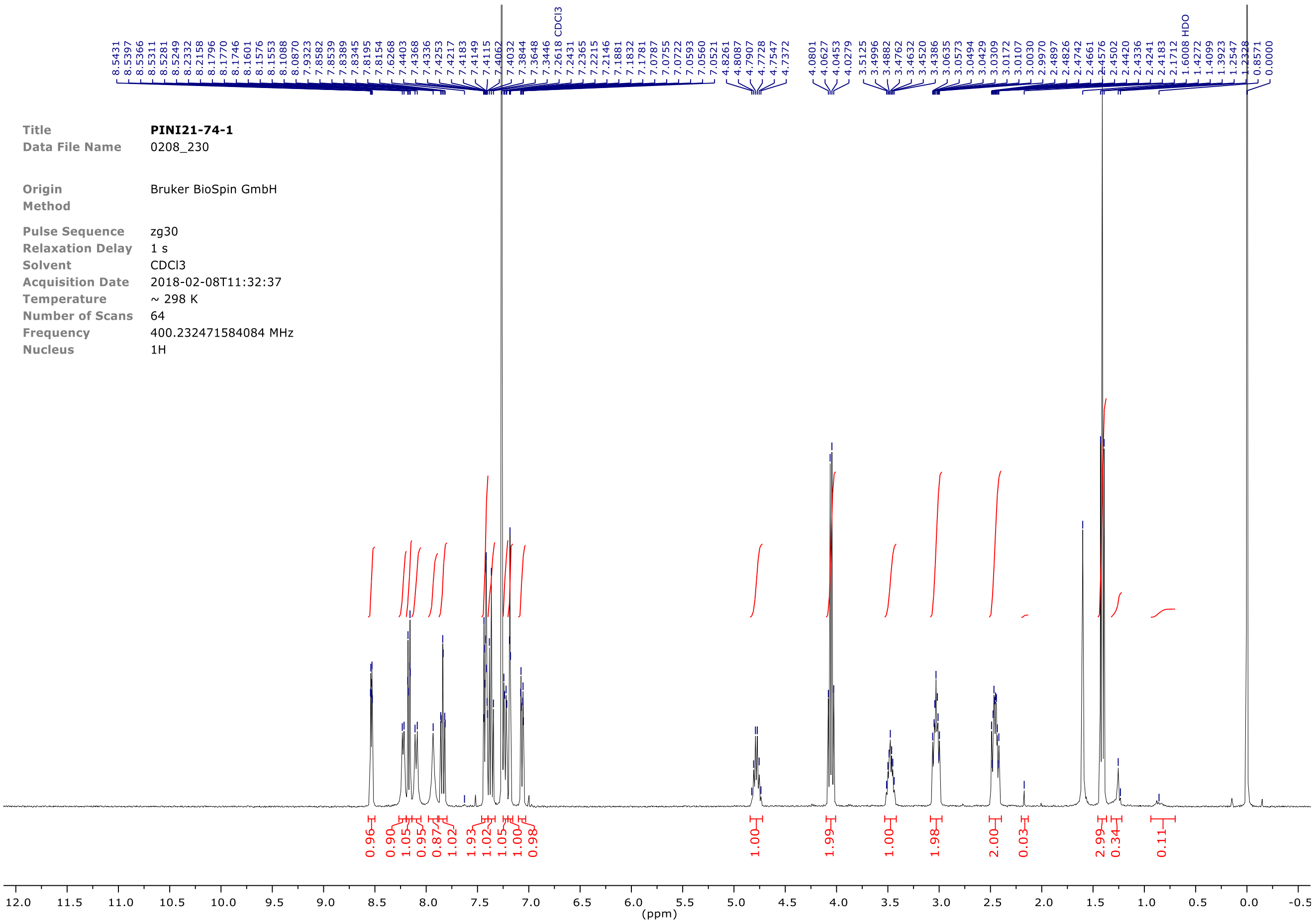

HRMS (m/z): C25H23N6O2Cl , [M+H]+ Calc: 475.16438; found: 475.1636, Δppm -1.73

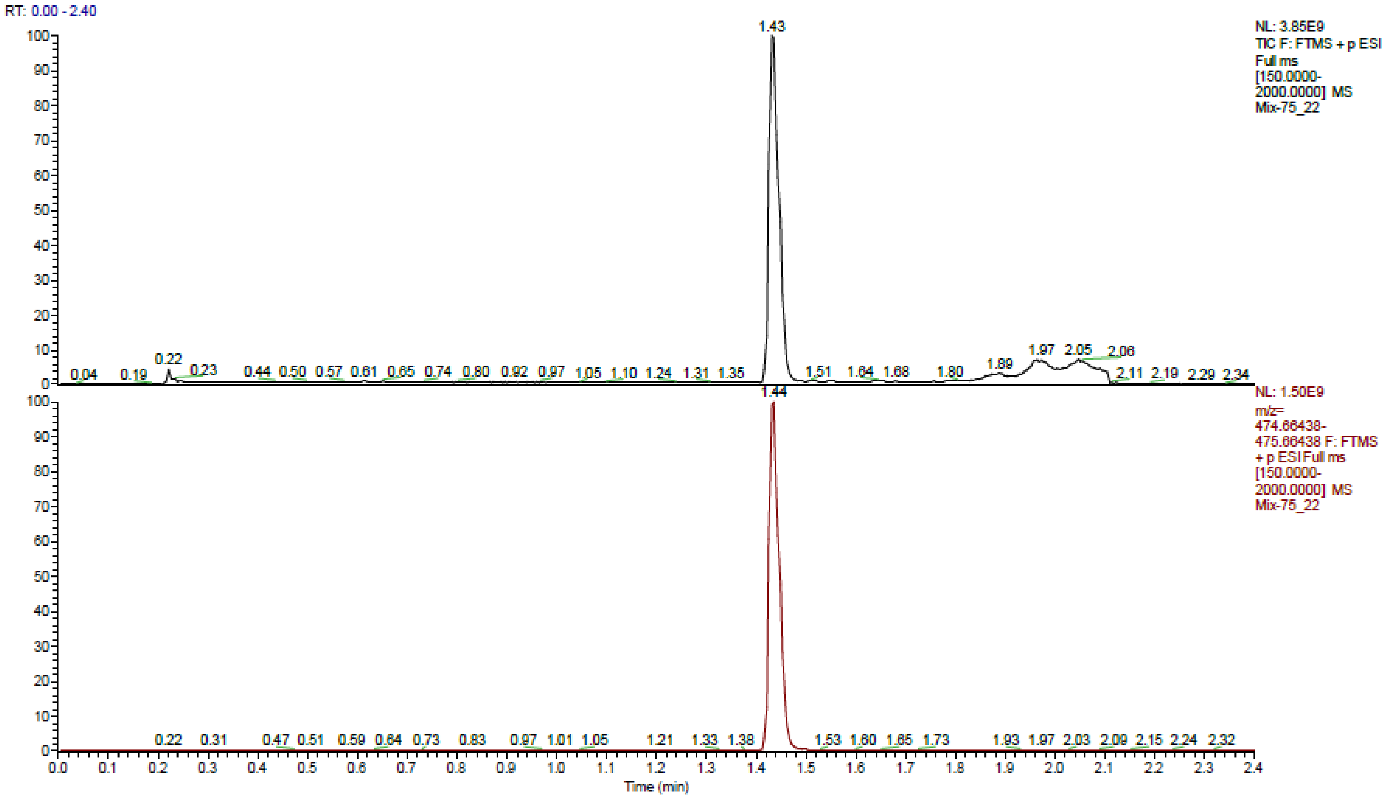

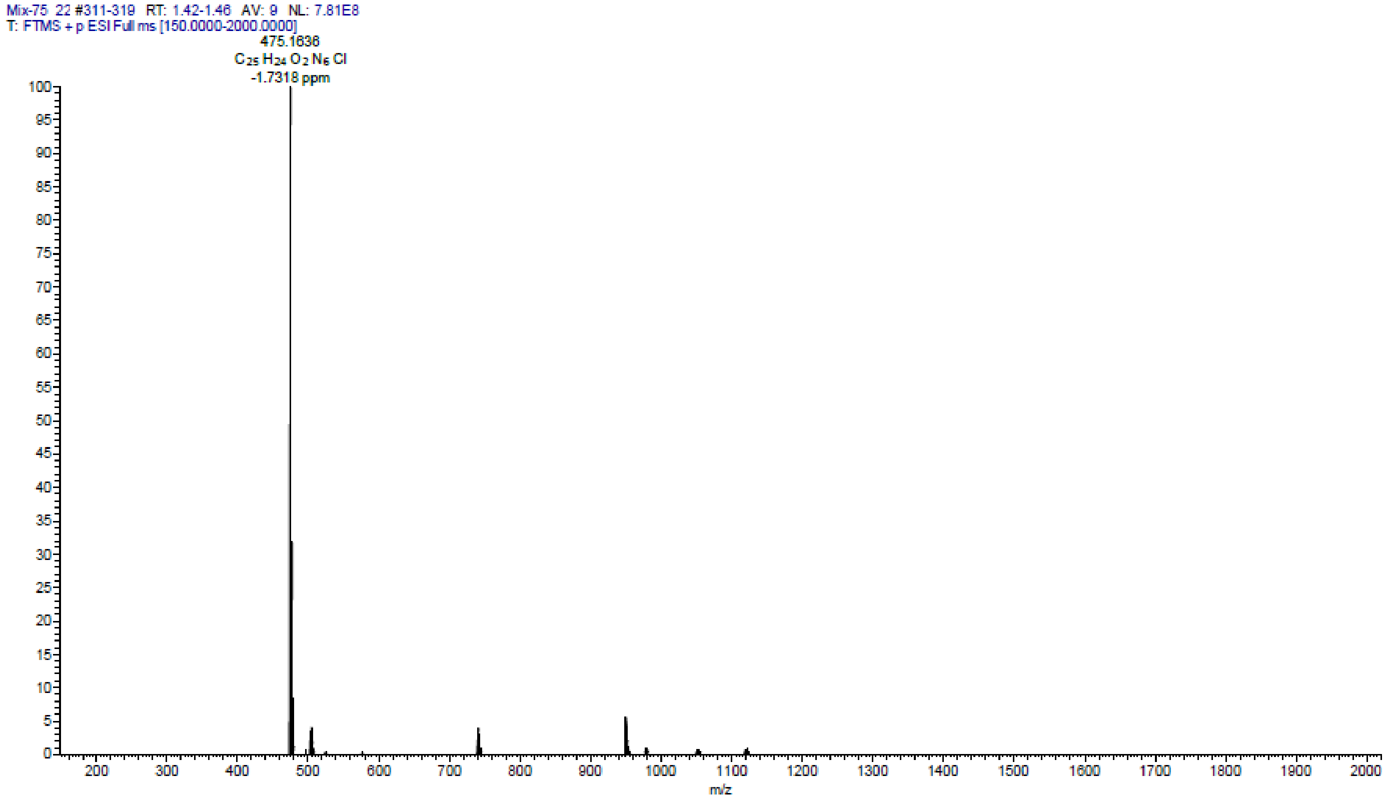

Compound 13

title	**sjae 54 184-3**
Method	AN_BASE.M
Date acquired	13-Nov-17, 17:25:02
FileName	Analysis\LCMS5_1113_064.D
Acq. method	AN_BASE valve: 2
Column	Waters XSelect CSH C18 (50x2.1mm, 3.5µ)
Flow	0.8 ml/min; Column temp: 25°C
Eluent A	95% acetonitrile + 5% 10 mM ammoniumbicarbonate in water
Eluent B	10mM ammoniumbicarbonate in water
Lin. Gradient	t=0 min 5% A, t=3.5 min 98% A, t=6 min 98% A
Posttime	2 min
Detection	DAD (210, 220 and 220-320 nm)
Detection	PDA (210-320 nm)
Detection	MSD (ESI pos/neg) mass range: 100-800

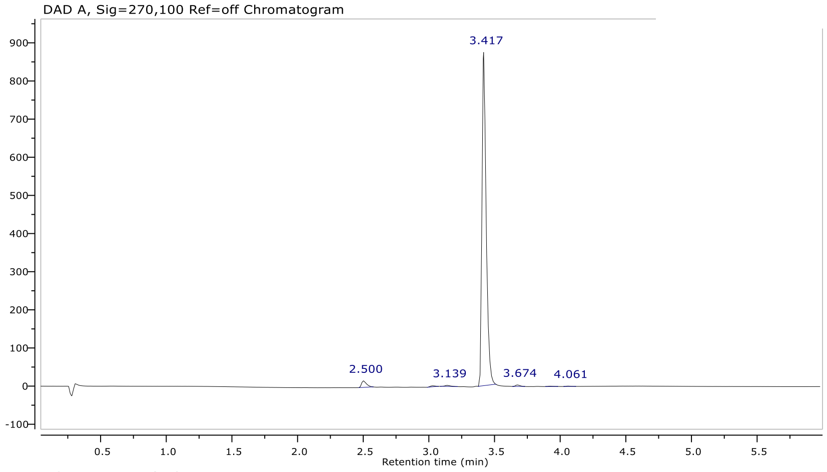

Integrals spectrum Chromatogram DAD A, Sig=270,100 Ref=off

rt (min)	height	area	area (%)
2.50	17.10	0.7428	2.22
3.03	2.693	0.1106	0.33
3.14	2.710	0.1349	0.40
3.42	873.8	32.30	96.37
3.67	3.915	0.1506	0.45
3.92	0.8755	0.03837	0.11
4.06	0.9464	0.03974	0.12

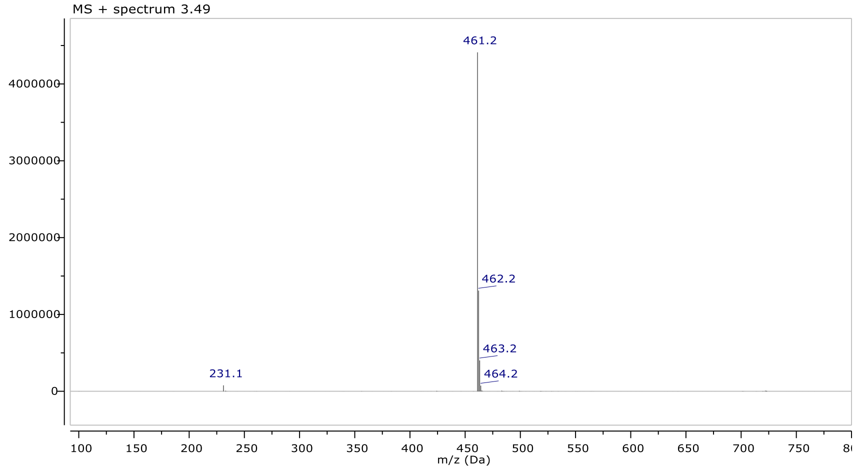

Title	**sjae 54 184-3**
Data File Name	1115_210
Origin	Bruker BioSpin GmbH
Method	
Pulse Sequence	zg30
Relaxation Delay	1 s
Solvent	CDCl3
Acquisition Date	2017-11-15T12:58:31
Temperature	~ 293.5 K
Number of Scans	64
Frequency	400.232471584084 MHz
Nucleus	1H

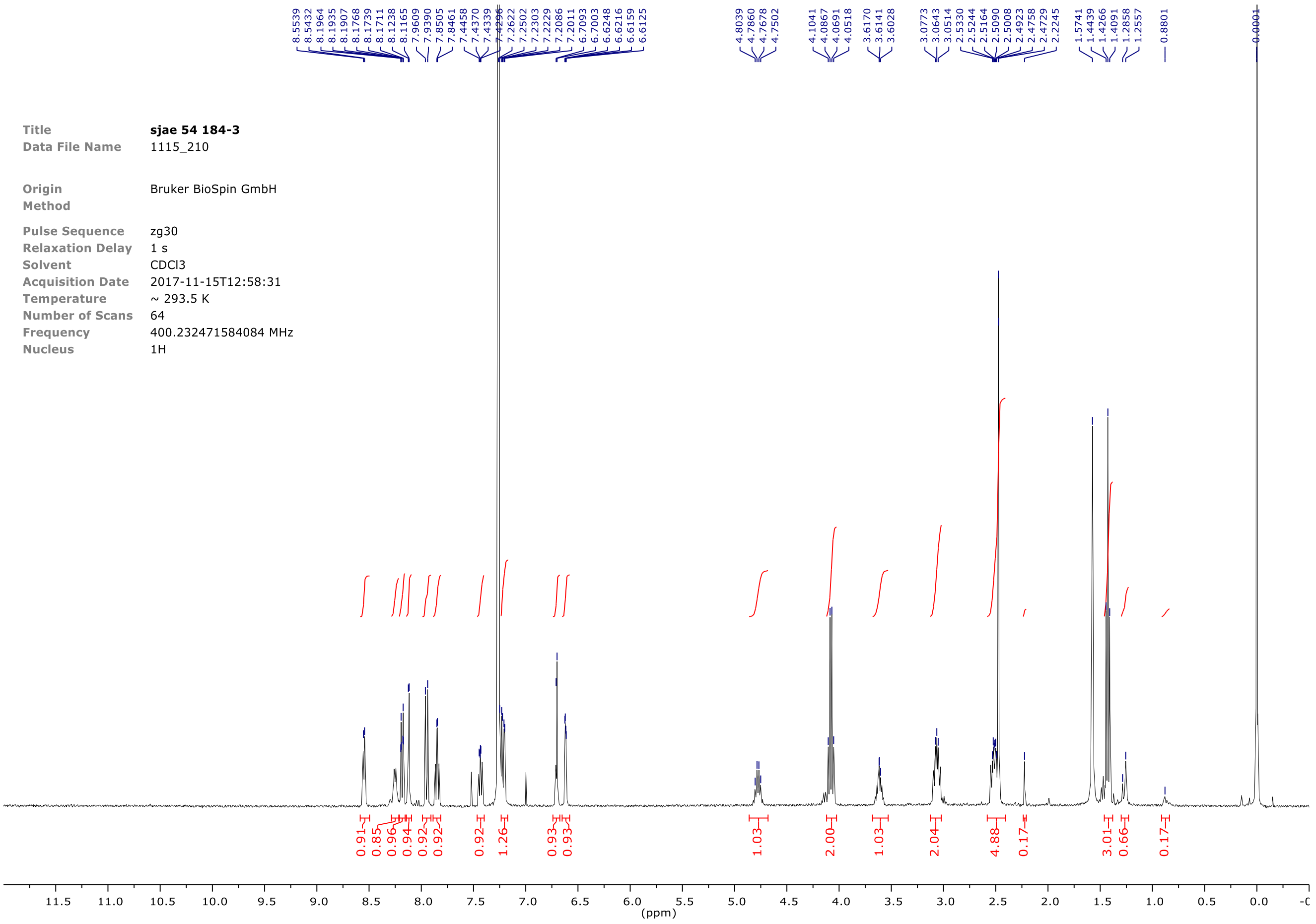

HRMS (m/z): C24H24N6O2S, [M+H]+ Calc: 461.17542; found: 461.1746, Δppm -1.85

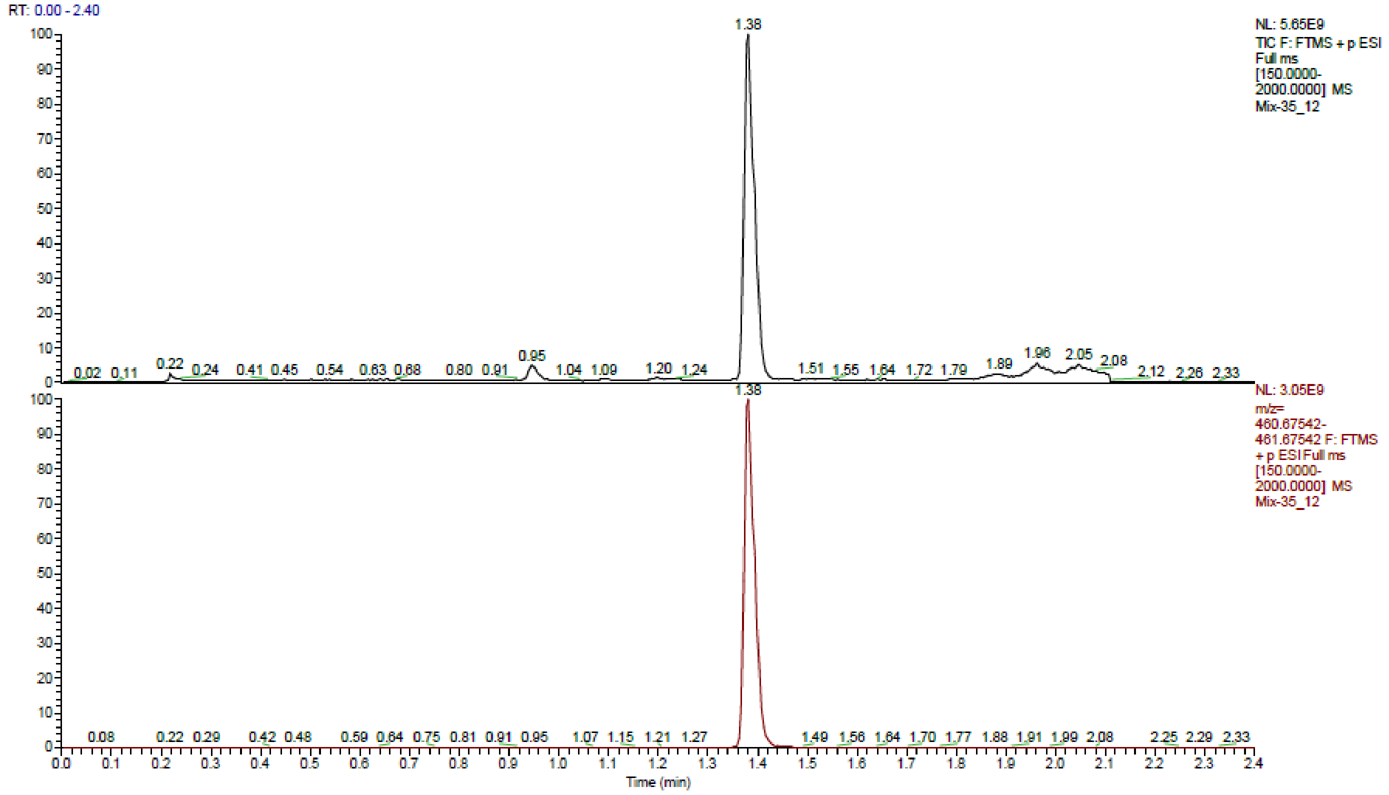

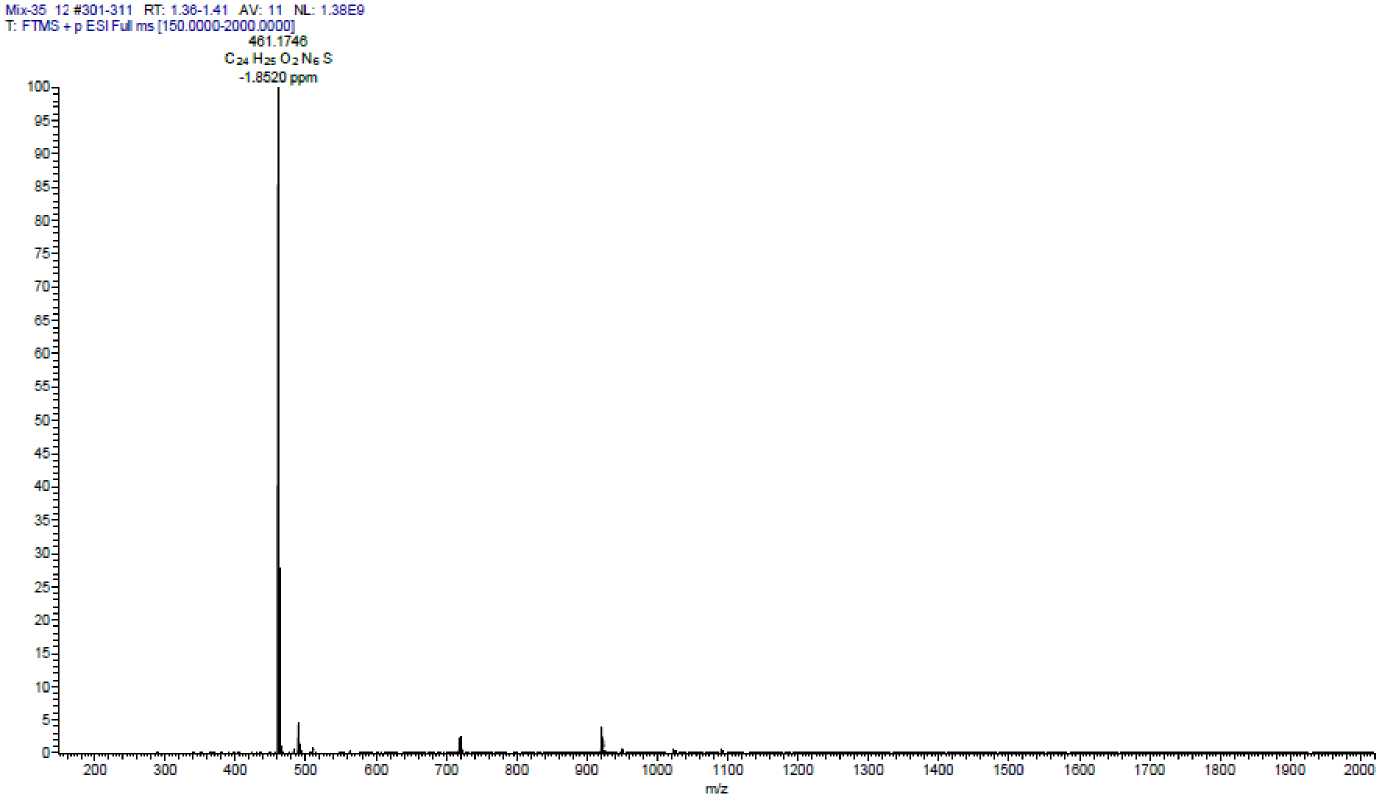

Compound 14

**title**	**sjae 213-2**
**Method**	AN_BASE.M
**Date acquired**	07-Dec-17, 19:01:03
**FileName**	Analysis\LCMS5_1207_108.D
**Acq. method**	AN_BASE valve: 2
**Column**	Waters XSelect CSH C18 (50x2.1mm, 3.5μ)
**Flow**	0.8 ml/min; Column temp: 25°C
**Eluent A**	95% acetonitrile + 5% 10 mM ammoniumbicarbonate in water
**Eluent B**	10mM ammoniumbicarbonate in water
**Lin. Gradient**	t=0 min 5% A, t=3.5 min 98% A, t=6 min 98% A
**Posttime**	2 min
**Detection**	DAD (210, 220 and 220-320 nm)
**Detection**	PDA (210-320 nm)
**Detection**	MSD (ESI pos/neg) mass range: 100-800

DAD A, Sig=270,100 Ref=off Chromatogram

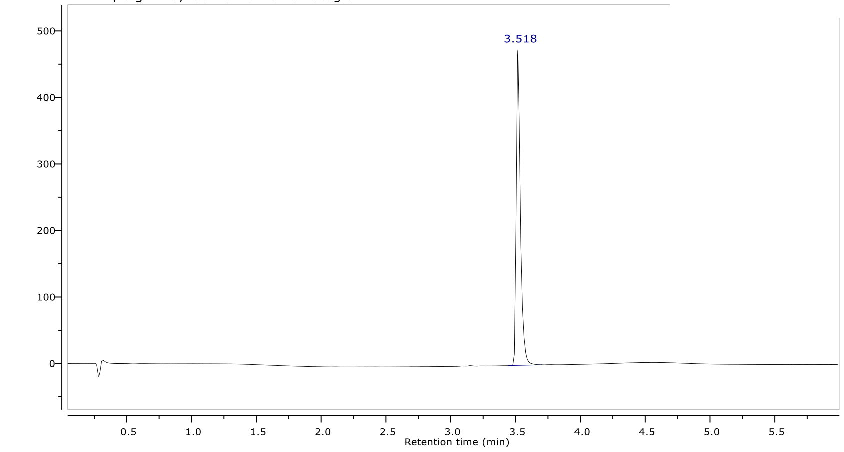

Integrals spectrum **Chromatogram DAD A, Sig=270,100 Ref=off**

rt (min)	height	area	area (%)
3.52	473.5	17.72	100.00

MS + spectrum 3.49..3.63

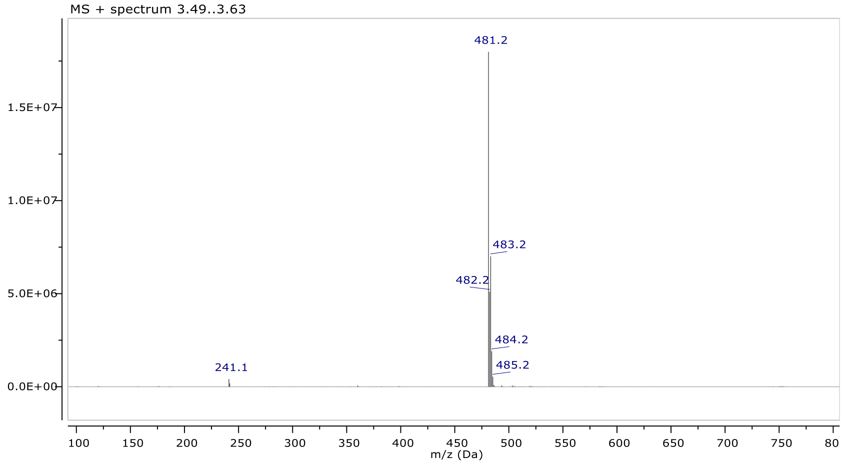

**Title**	**sjae 54 213-2**
**Data File Name**	1208_1050
**Origin**	Bruker BioSpin GmbH
**Method**	
**Pulse Sequence**	zg30
**Relaxation Delay**	1 s
**Solvent**	CDCl3
**Acquisition Date**	2017-12-10T09:36:42
**Temperature**	~ 298 K
**Number of Scans**	64
**Frequency**	400.232471584084 MHz
**Nucleus**	1H

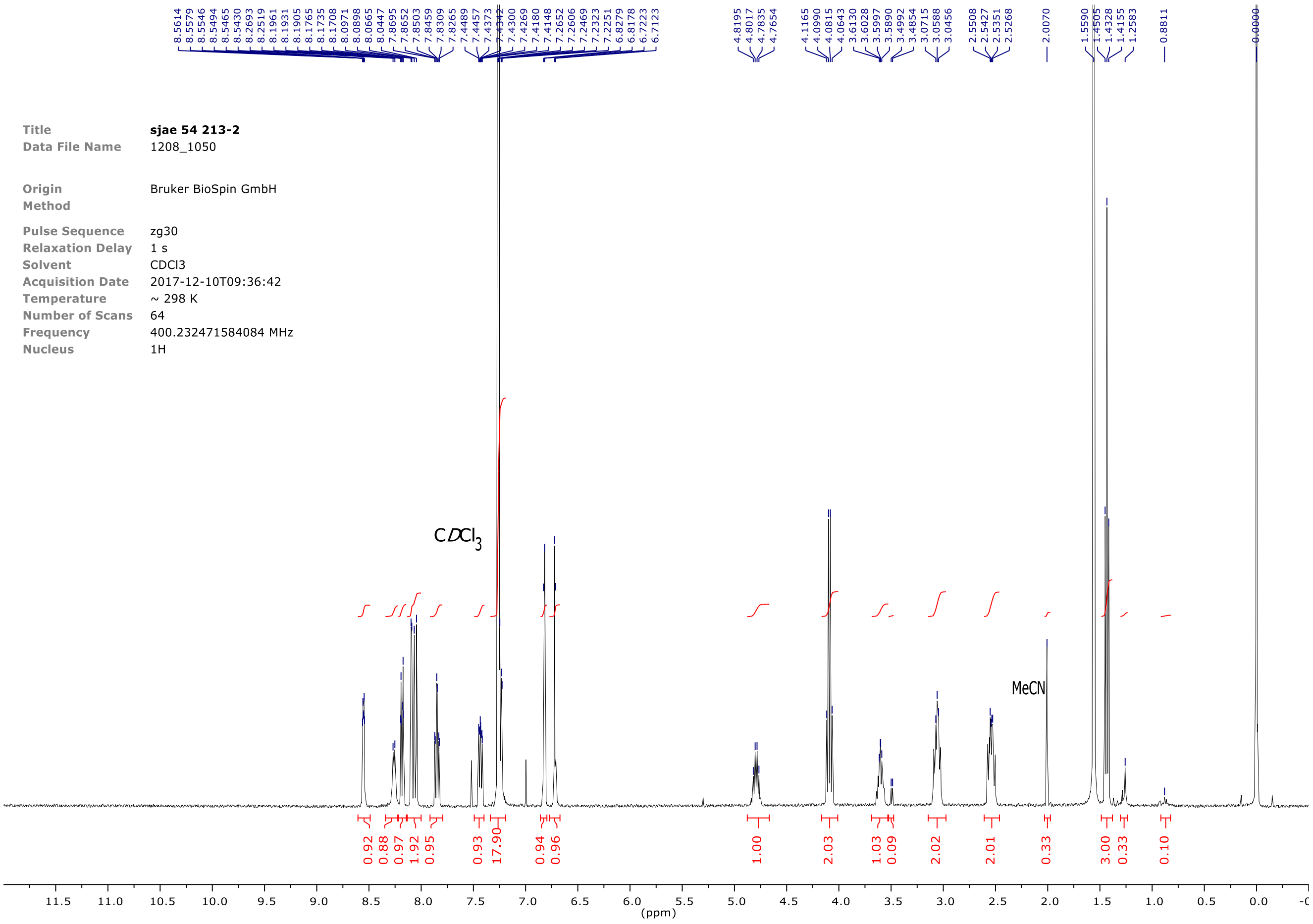

HRMS (m/z): C23H21N6O2ClS, [M+H]+ Calc: 481.12080; found: 481.1201, Δppm -1.41

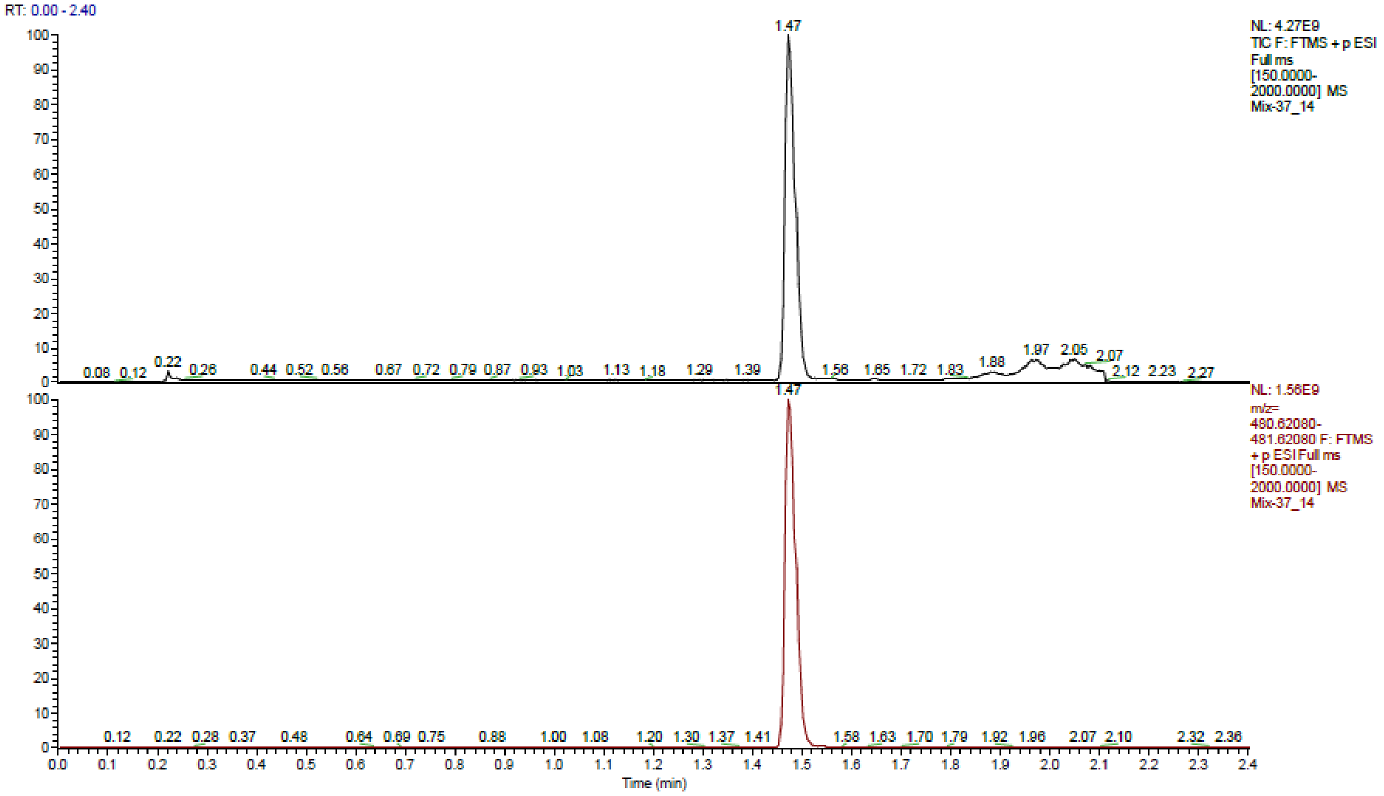

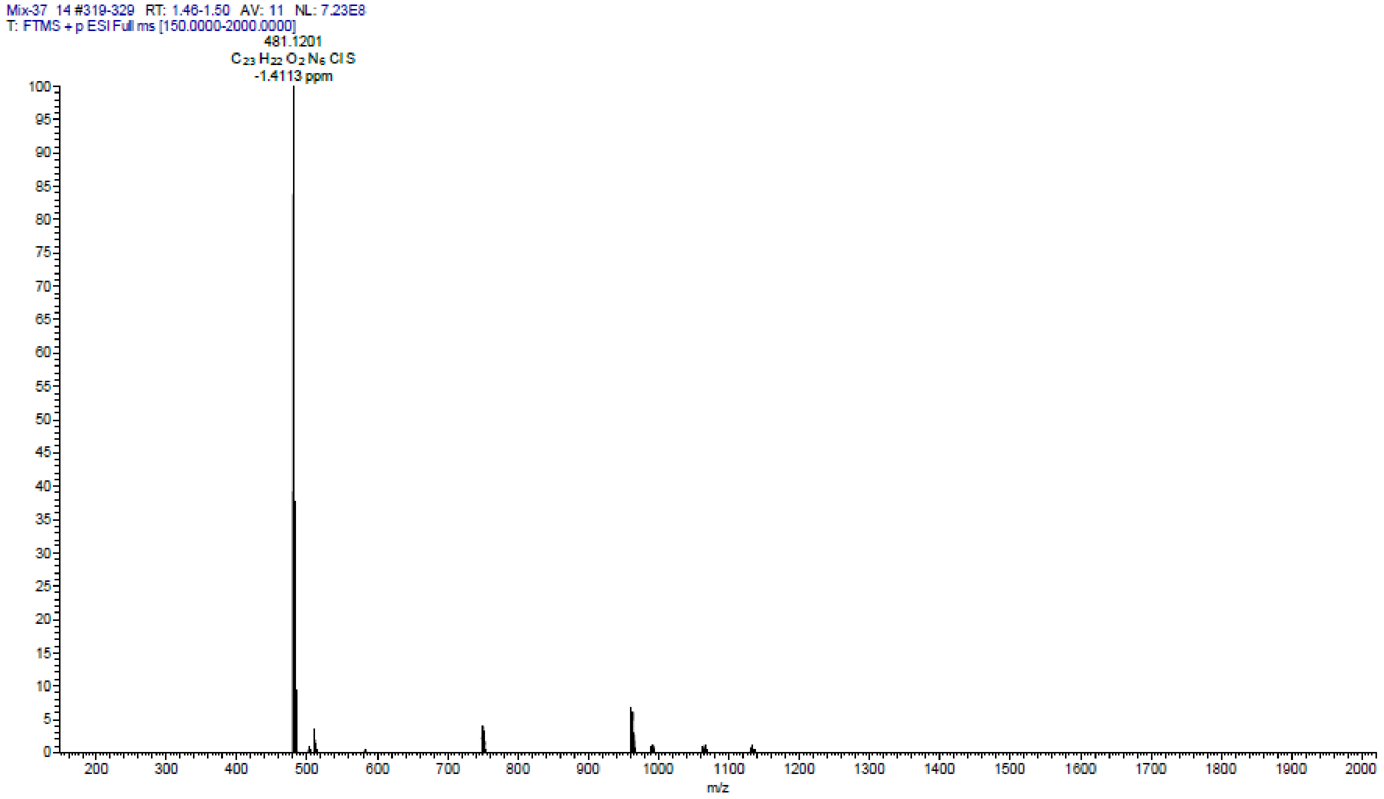

Compound 16a

title	**PINI22-34-1**
Method	AN_ACID.M
Date acquired	30-Apr-18, 07:56:13
FileName	Analysis\LCMS6_0430_007.D
Column	XSelect CSH C18 (50x2.1mm, 3.5μ) valve:3
Flow	0.8 ml/min; Column temp: 35°C
Eluent A	0.1% formic acid in acetonitrile
Eluent B	0.1% formic acid in water
Gradient	t=0 min 5% A, t=3.5 min 98% A, t=6 min 98%A
Posttime	2 min
Detection	DAD(210, 220 and 220-320nm)
Detection	PDA(210-320nm)
Detection	MSD (ESI pos/neg) mass range: 100 - 1000
Detection	ELSD gas temp: 40°C, flow 1.5 ml/min, gain 1

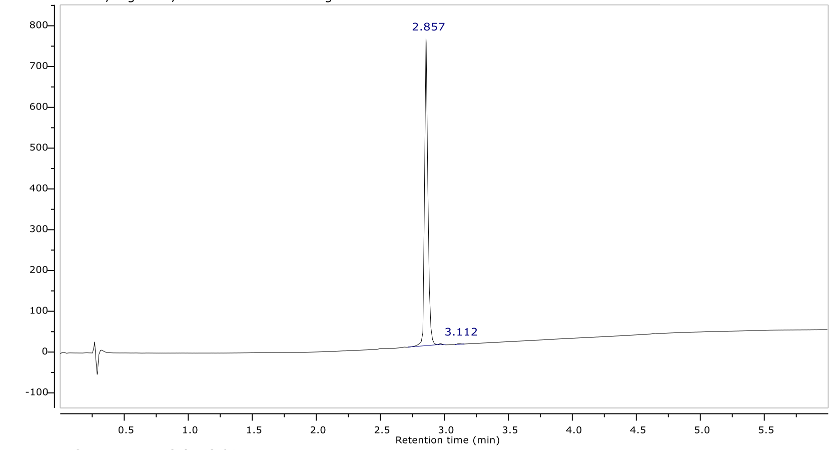

Integrals spectrum Chromatogram DAD A, Sig=270,100 Ref=off

rt (min)	height	area	area (%)
2.86	753.2	23.02	99.45
2.97	2.857	0.07367	0.32
3.11	1.839	0.05285	0.23

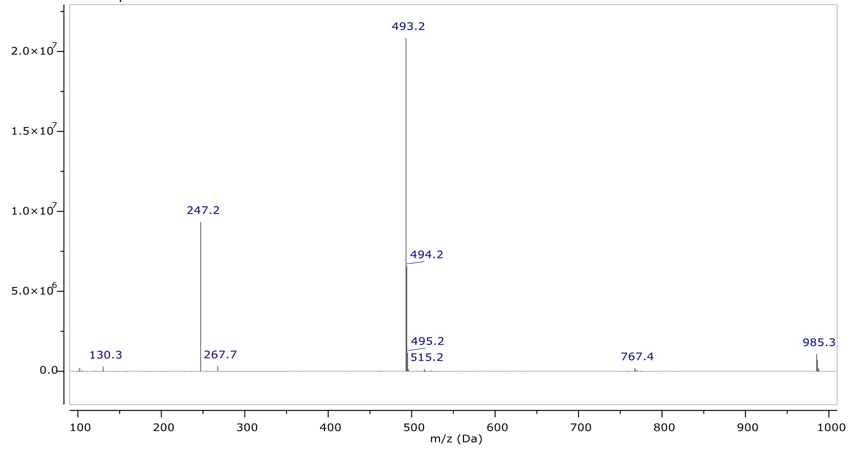

Title	**PINI22-34-1**
Data File Name	0426_270
Origin	Bruker BioSpin GmbH
Method	
Pulse Sequence	zg30
Relaxation Delay	1 s
Solvent	CDCl3
Acquisition Date	2018-04-26T15:16:59
Temperature	~ 293.1 K
Number of Scans	32
Frequency	400.232471584084 MHz
Nucleus	1H

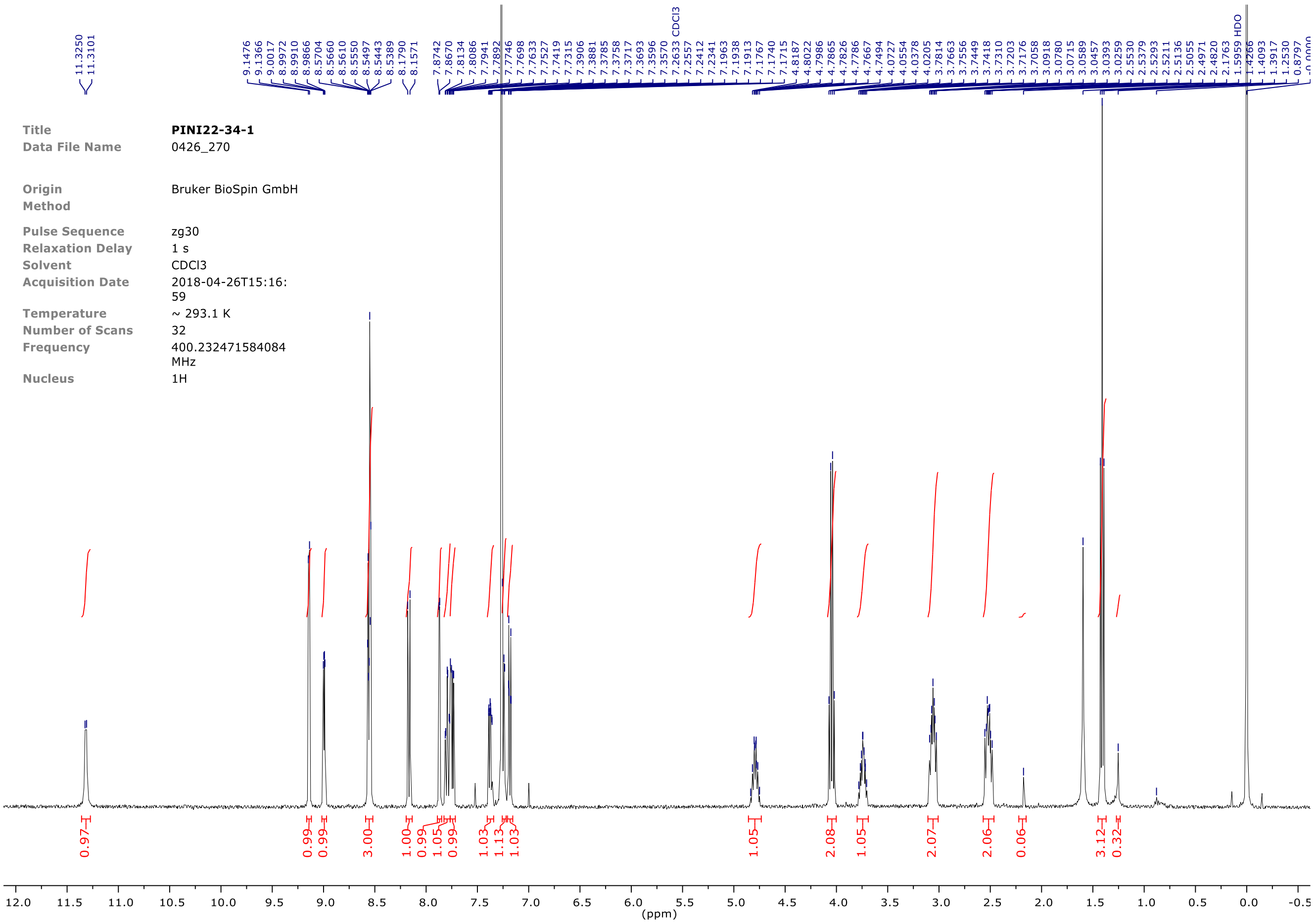

HRMS (m/z): C27H24N8O2, [M+H]+ Calc: 493.20950; found: 493.2085, Δppm -1.99

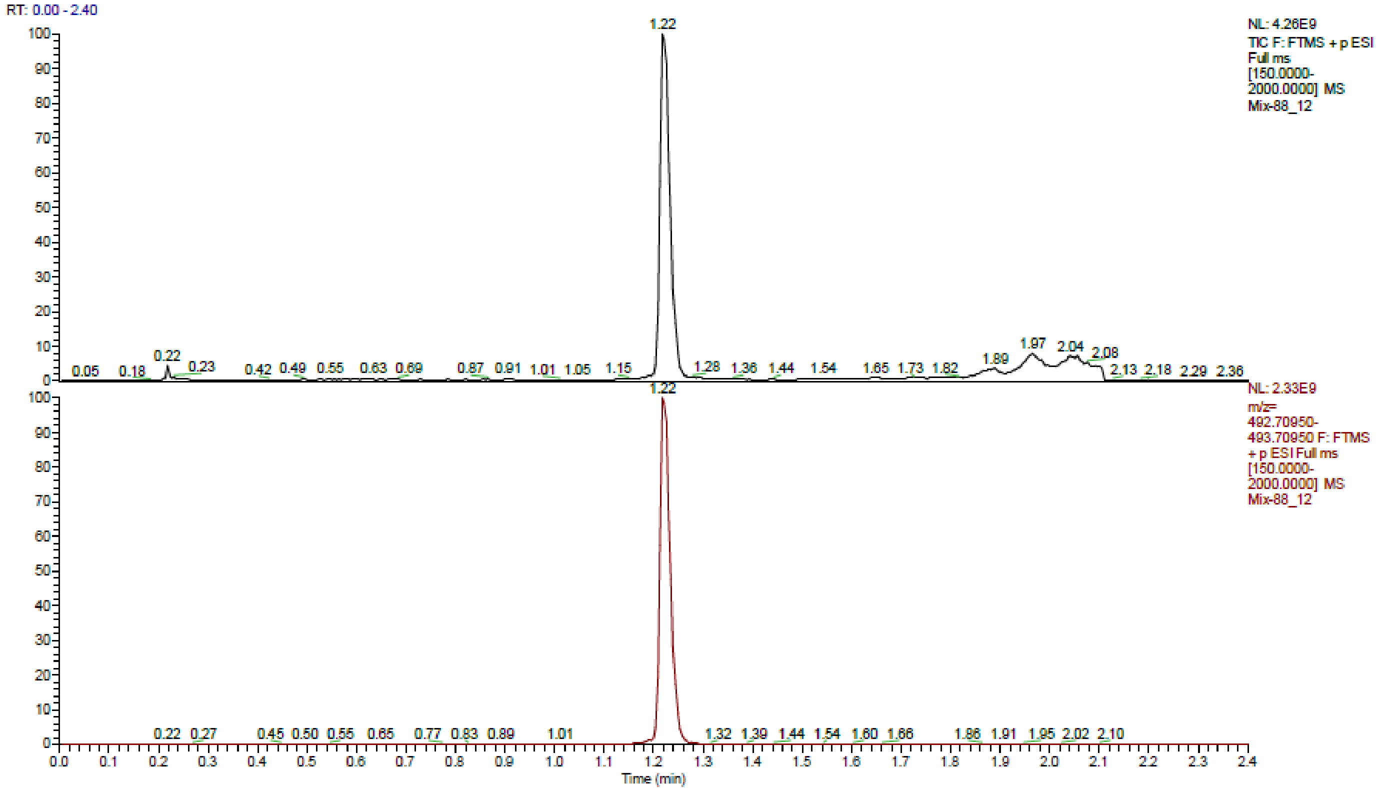

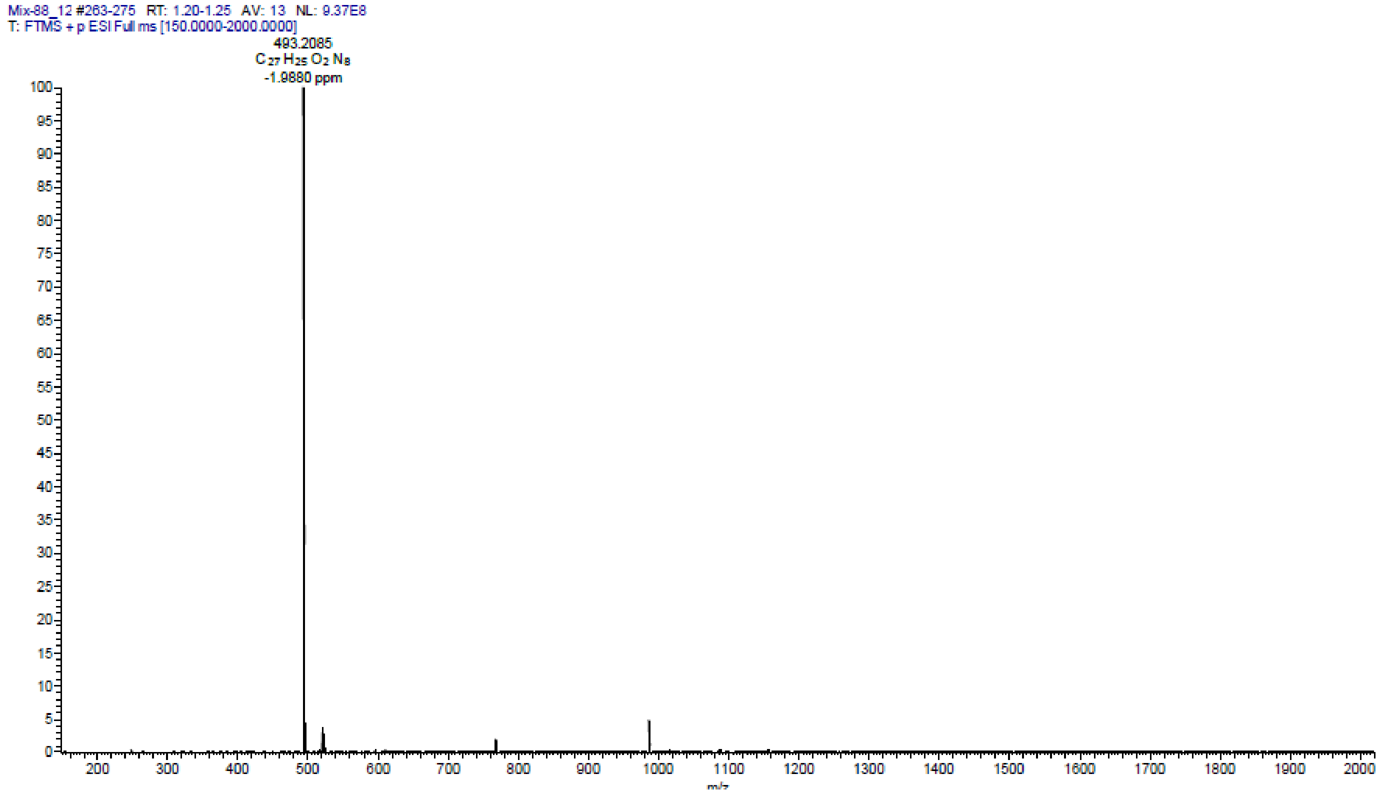

Compound 16b

title	**PINI23-45-1**
Method	AN_ACID.M
Date acquired	04-Oct-18, 16:32:14
FileName	Analysis\LCMS6_1004_072.D
Column	XSelect CSH C18 (50x2.1mm, 3.5μ) valve:3
Flow	0.8 ml/min; Column temp: 35°C
Eluent A	0.1% formic acid in acetonitrile
Eluent B	0.1% formic acid in water
Gradient	t=0 min 5% A, t=3.5 min 98% A, t=6 min 98%A
Posttime	2 min
Detection	DAD(210, 220 and 220-320nm)
Detection	PDA(210-320nm)
Detection	MSD (ESI pos/neg) mass range: 100 - 1000
Detection	ELSD gas temp: 40°C, flow 1.5 ml/min, gain 1

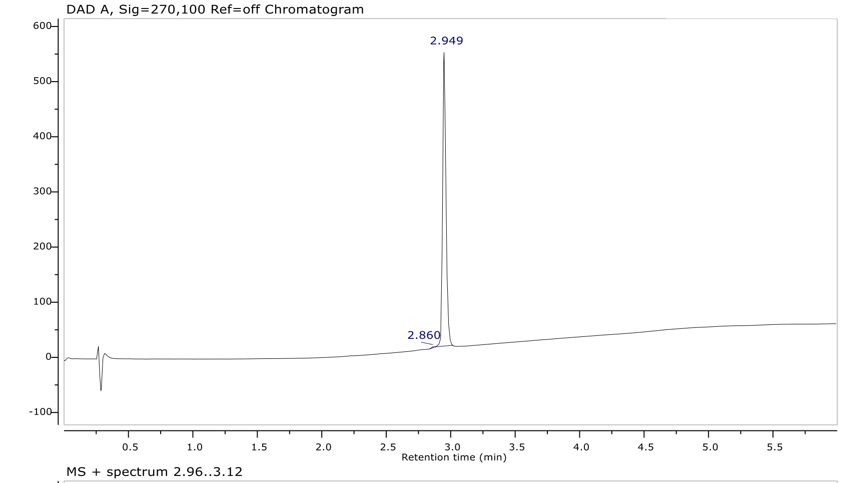

Integrals spectrum — Chromatogram DAD A, Sig=270,100 Ref=off

rt (min)	height	area	area (%)
2.86	1.630	0.03502	0.22
2.95	532.4	16.05	99.78

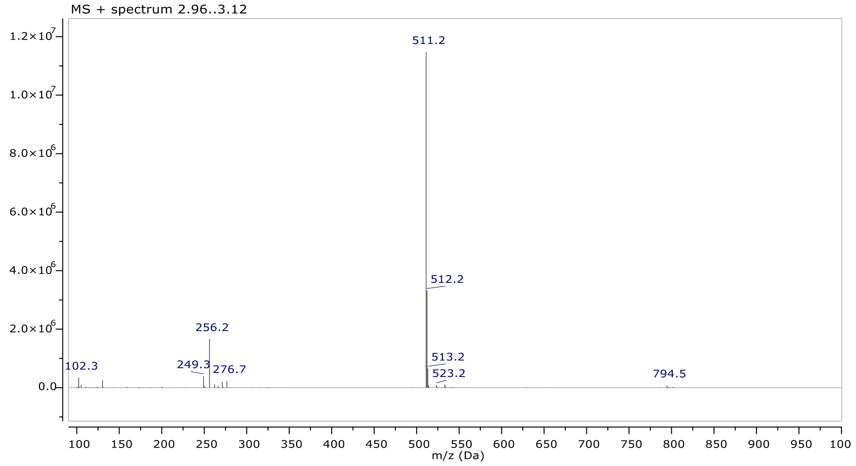

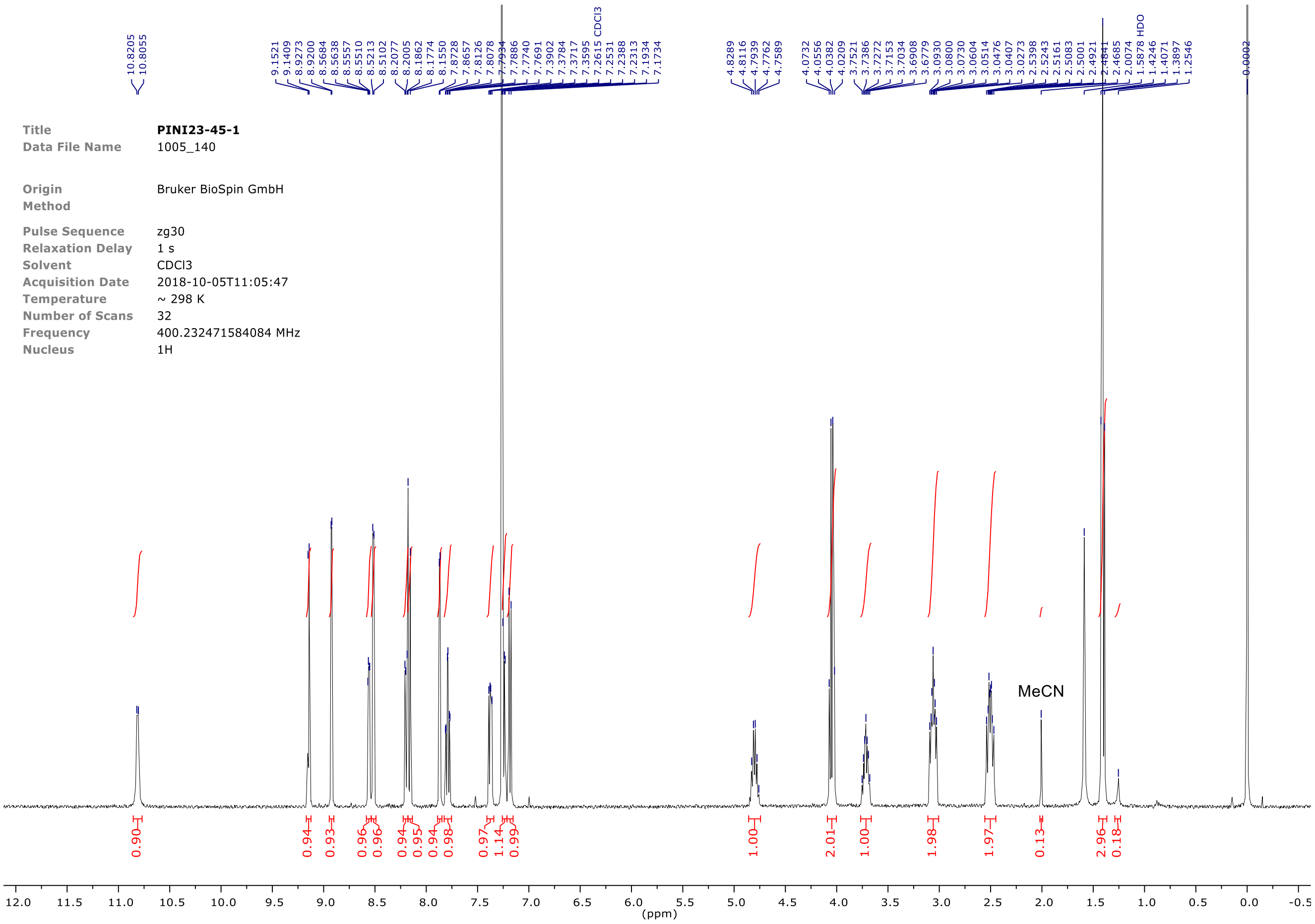

Title	**PINI23-45-1**
Data File Name	1005_140
Origin	Bruker BioSpin GmbH
Method	
Pulse Sequence	zg30
Relaxation Delay	1 s
Solvent	CDCl3
Acquisition Date	2018-10-05T11:05:47
Temperature	~ 298 K
Number of Scans	32
Frequency	400.232471584084 MHz
Nucleus	1H

HRMS (m/z): C27H23N8O2F, [M+H]+ Calc: 511.20008; found: 511.1991, Δppm -1.86

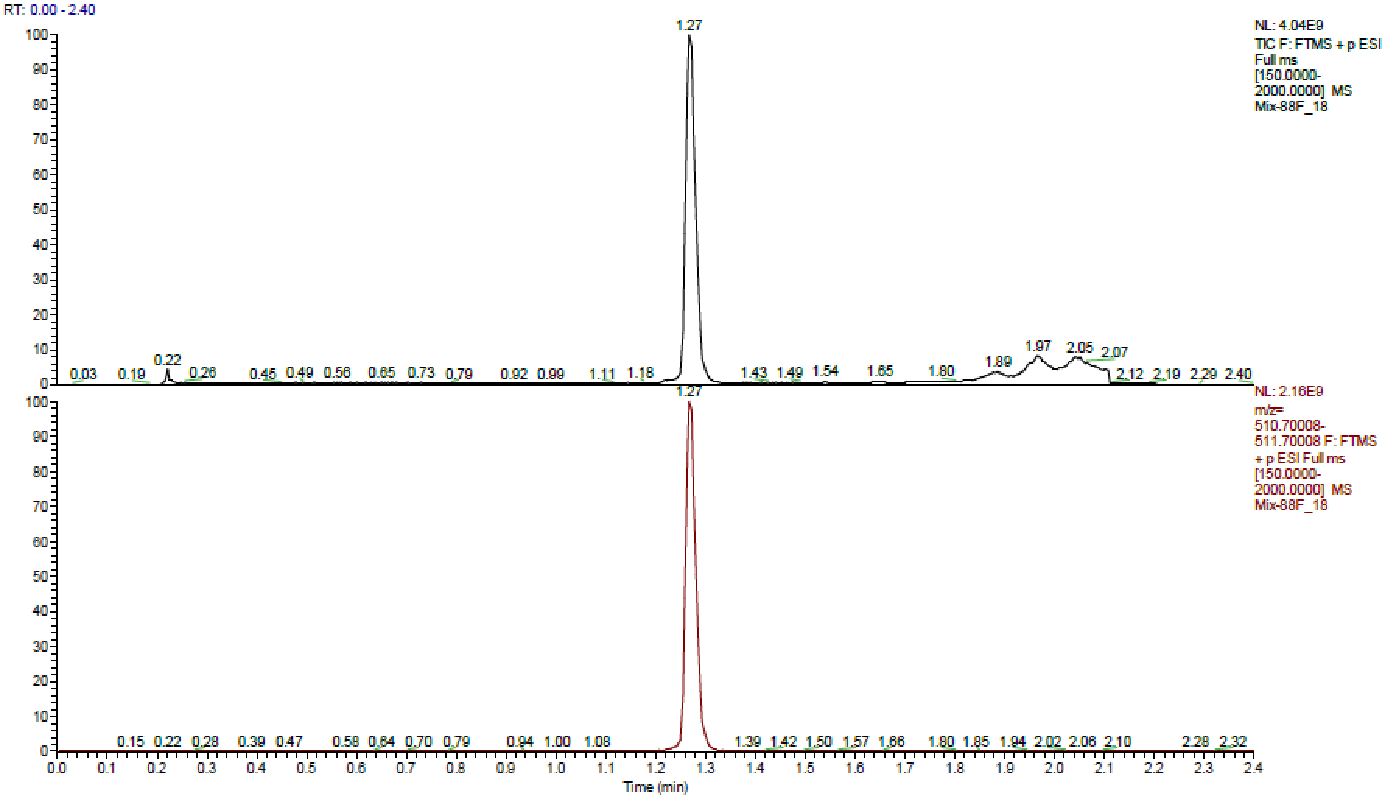

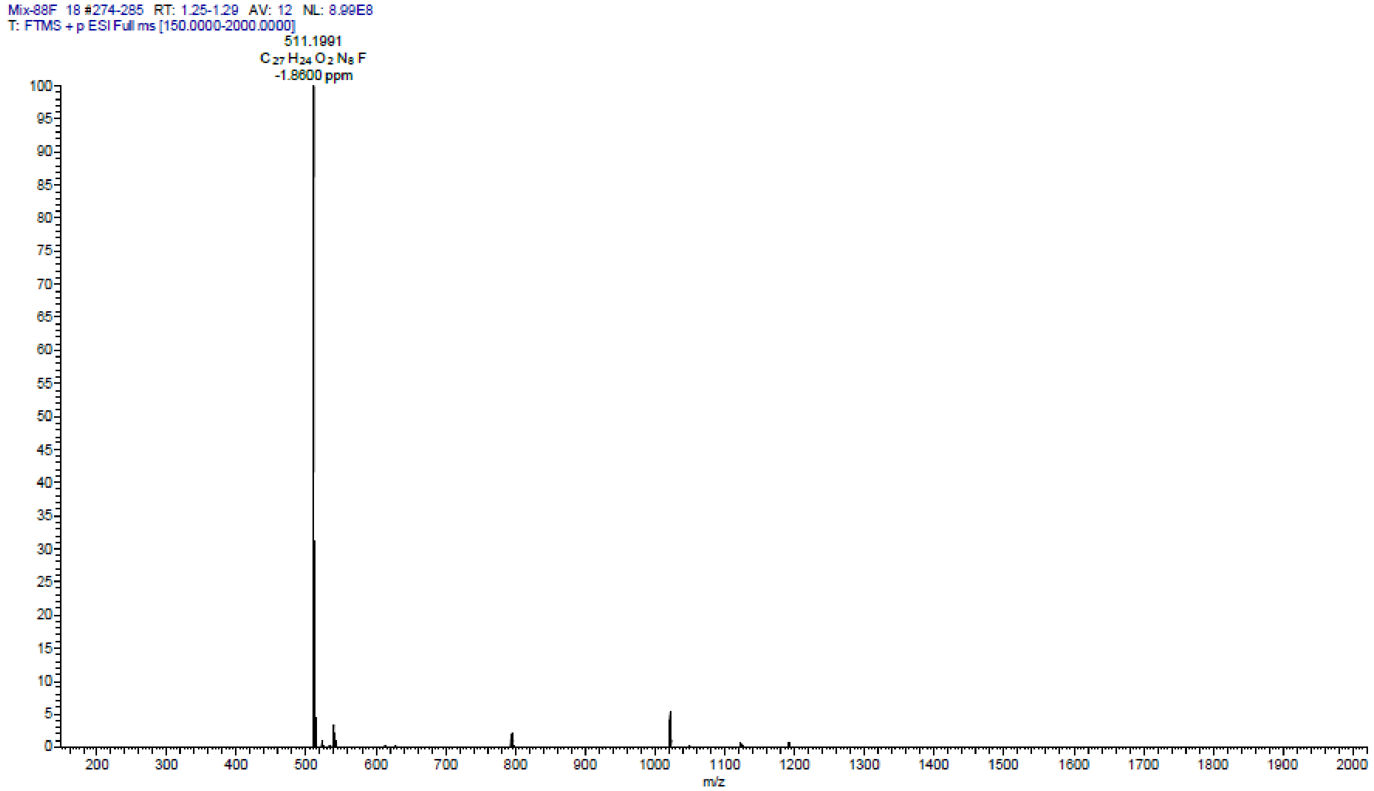

Compound 17a

**title**	**PINI22-87-1**
**Method**	AN_ACID.M
**Date acquired**	05-Jul-18, 21:33:18
**FileName**	Analysis\LCMS6_0705_091.D
**Column**	XSelect CSH C18 (50x2.1mm, 3.5μ) valve:3
**Flow**	0.8 ml/min; Column temp: 35°C
**Eluent A**	0.1% formic acid in acetonitrile
**Eluent B**	0.1% formic acid in water
**Gradient**	t=0 min 5% A, t=3.5 min 98% A, t=6 min 98%A
**Posttime**	2 min
**Detection**	DAD(210, 220 and 220-320nm)
**Detection**	PDA(210-320nm)
**Detection**	MSD (ESI pos/neg) mass range: 100 - 1000
**Detection**	ELSD gas temp: 40°C, flow 1.5 ml/min, gain 1

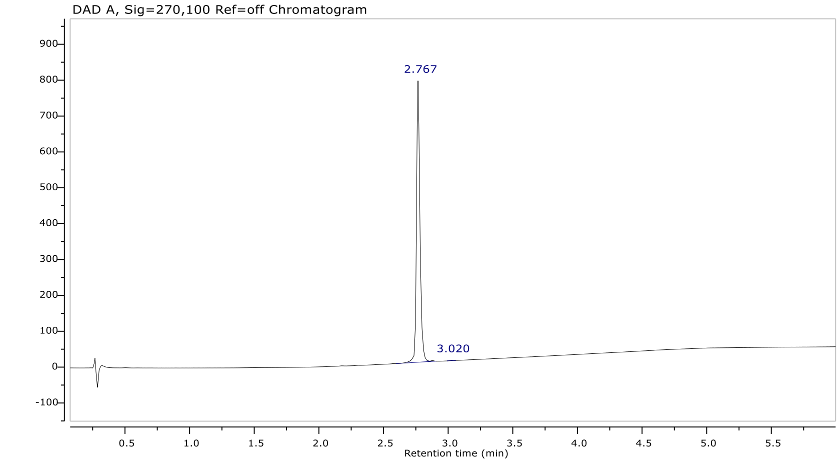

Integrals spectrum Chromatogram DAD A, Sig=270,100 Ref=off

rt (min)	height	area	area (%)
2.77	784.6	23.95	99.53
2.88	2.294	0.07873	0.33
3.02	1.337	0.03456	0.14

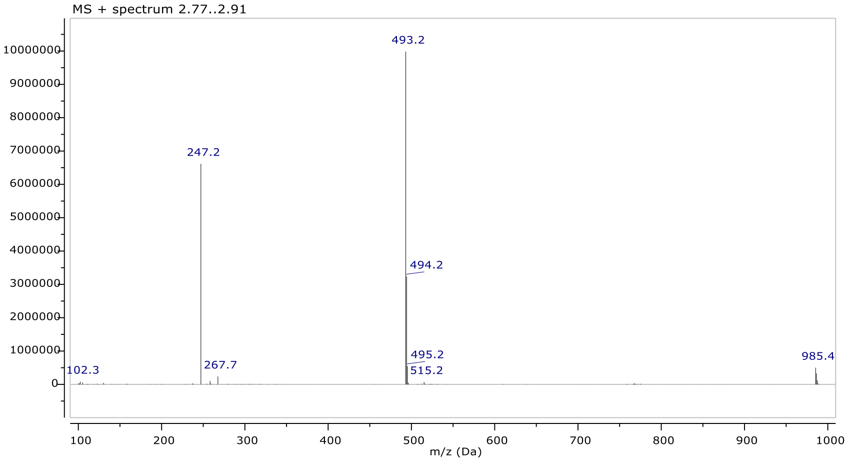

**Title**	**PINI22-87-1**
**Data File Name**	0706_150
**Origin**	Bruker BioSpin GmbH
**Method**	
**Pulse Sequence**	zg30
**Relaxation Delay**	1 s
**Solvent**	CDCl3
**Acquisition Date**	2018-07-06T10:59:12
**Temperature**	~ 298 K
**Number of Scans**	64
**Frequency**	400.232471584084 MHz
**Nucleus**	1H

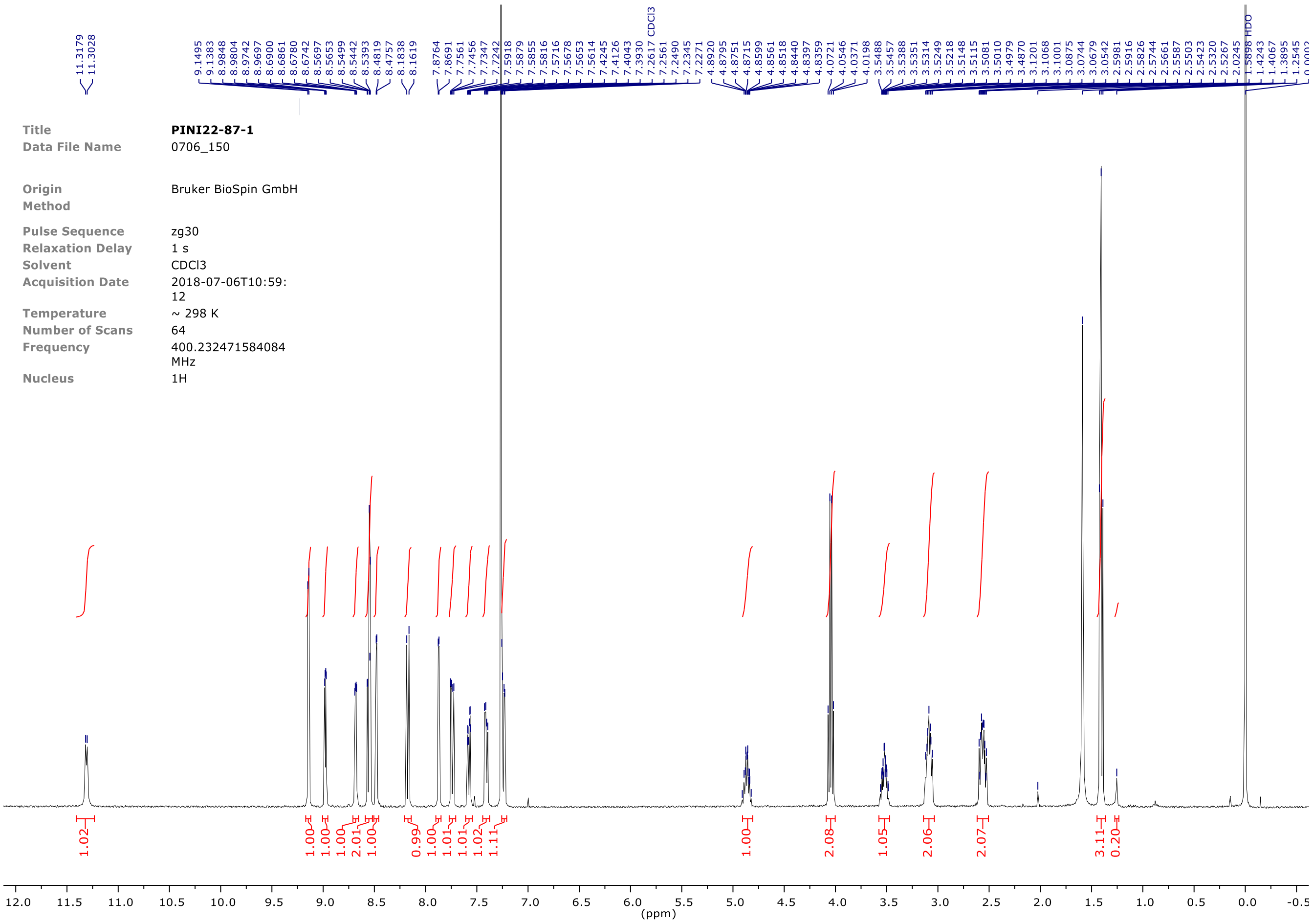

HRMS (m/z): C27H24N8O2, [M+H]+ Calc: 493.20950; found: 493.2084, Δppm -2.32

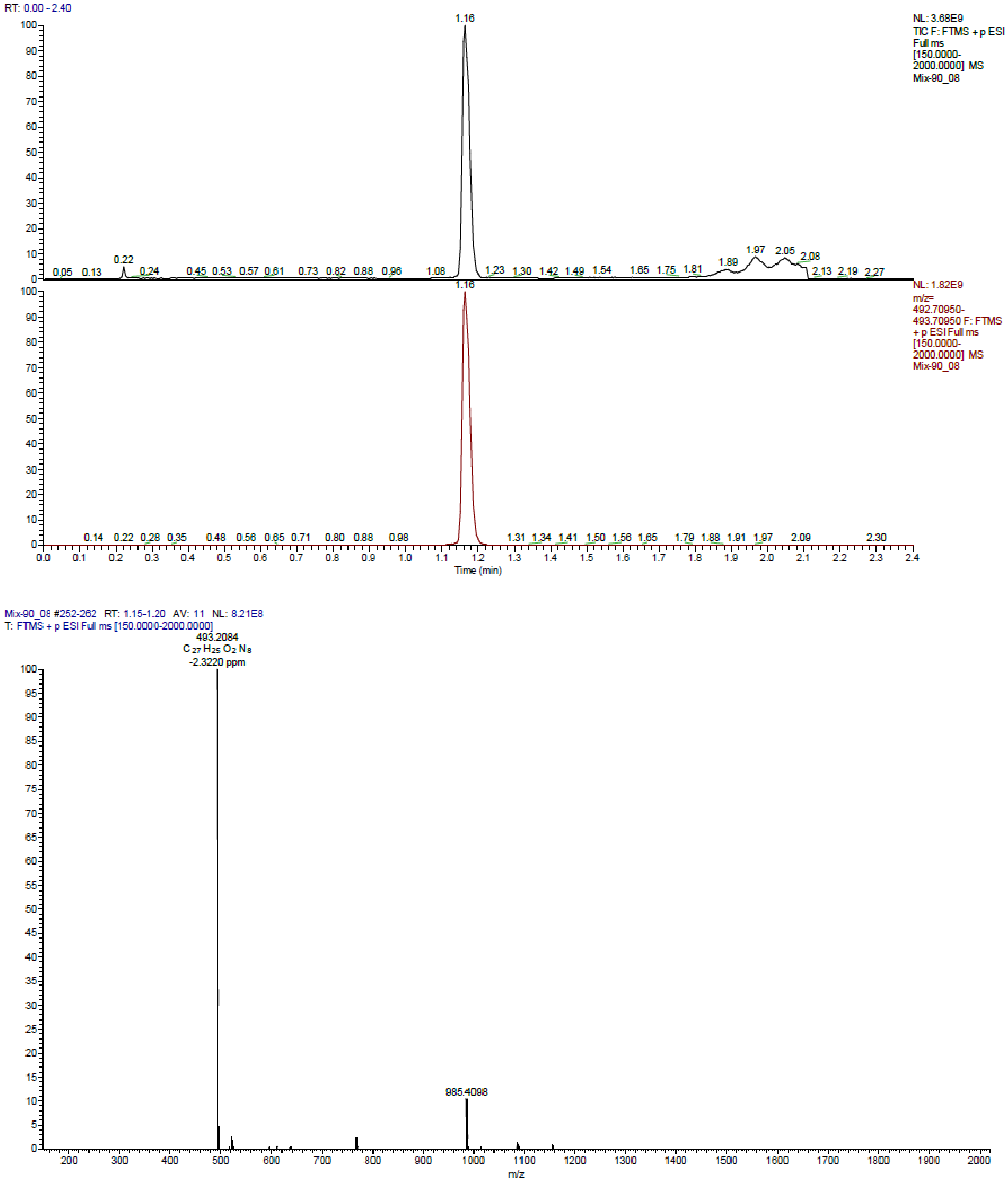

Compound 17b

title	**PINI23-46-1**
Method	AN_ACID.M
Date acquired	05-Oct-18, 18:11:59
FileName	Analysis\LCMS6_1005_093.D
Column	XSelect CSH C18 (50x2.1mm, 3.5µ) valve:3
Flow	0.8 ml/min; Column temp: 35°C
Eluent A	0.1% formic acid in acetonitrile
Eluent B	0.1% formic acid in water
Gradient	t=0 min 5% A, t=3.5 min 98% A, t=6 min 98%A
Posttime	2 min
Detection	DAD(210, 220 and 220-320nm)
Detection	PDA(210-320nm)
Detection	MSD (ESI pos/neg) mass range: 100 - 1000
Detection	ELSD gas temp: 40°C, flow 1.5 ml/min, gain 1

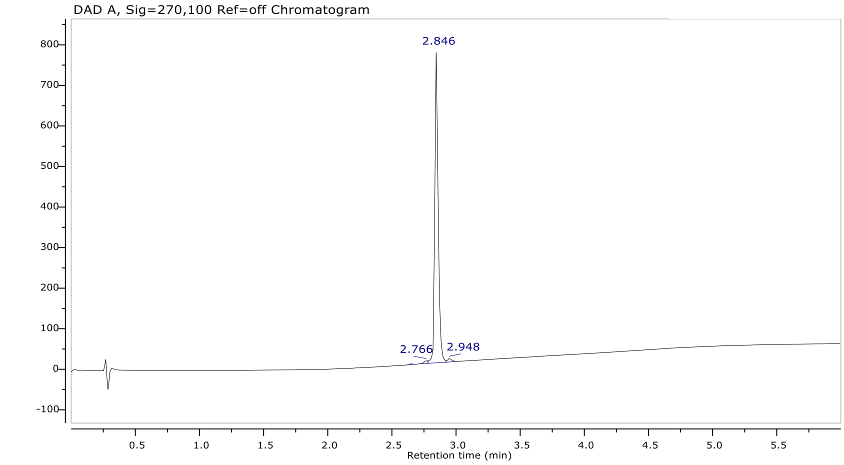

Integrals spectrum Chromatogram DAD A, Sig=270,100 Ref=off

rt (min)	height	area	area (%)
2.65	1.922	0.04536	0.19
2.77	6.284	0.1979	0.82
2.85	764.9	23.68	97.68
2.95	8.574	0.3195	1.32

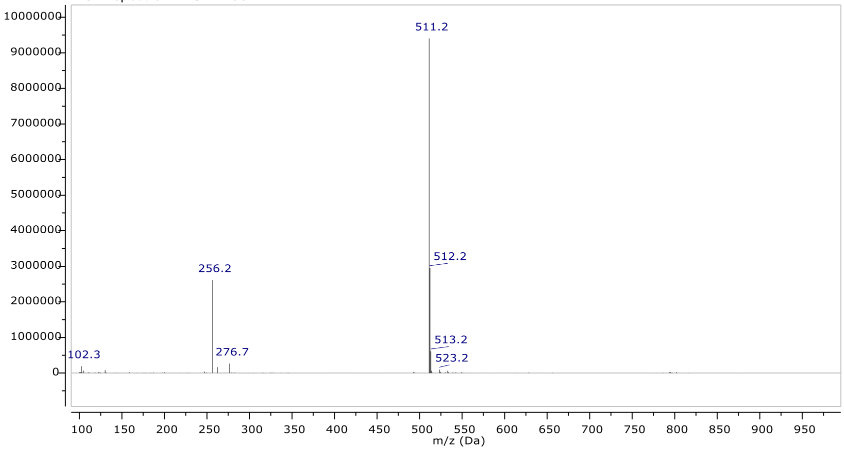

Title	**PINI23-46-1**
Data File Name	1005_150
Origin	Bruker BioSpin GmbH
Method	
Pulse Sequence	zg30
Relaxation Delay	1 s
Solvent	CDCl3
Acquisition Date	2018-10-05T11:16:33
Temperature	~ 298 K
Number of Scans	32
Frequency	400.232471584084 MHz
Nucleus	1H

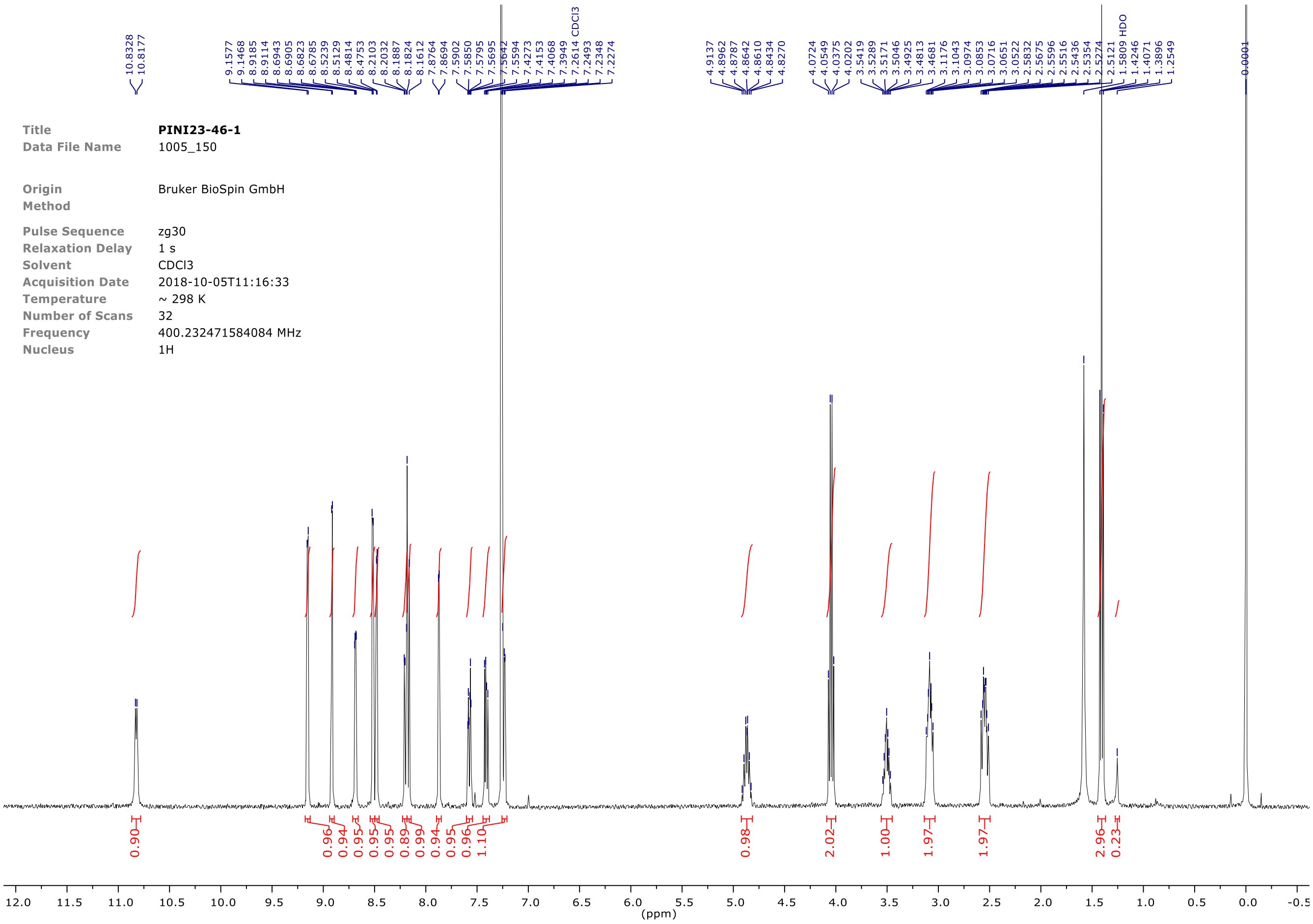

HRMS (m/z): C27H23N8O2F, [M+H]+ Calc 511.20008; found: 511.1991, Δppm -1.95

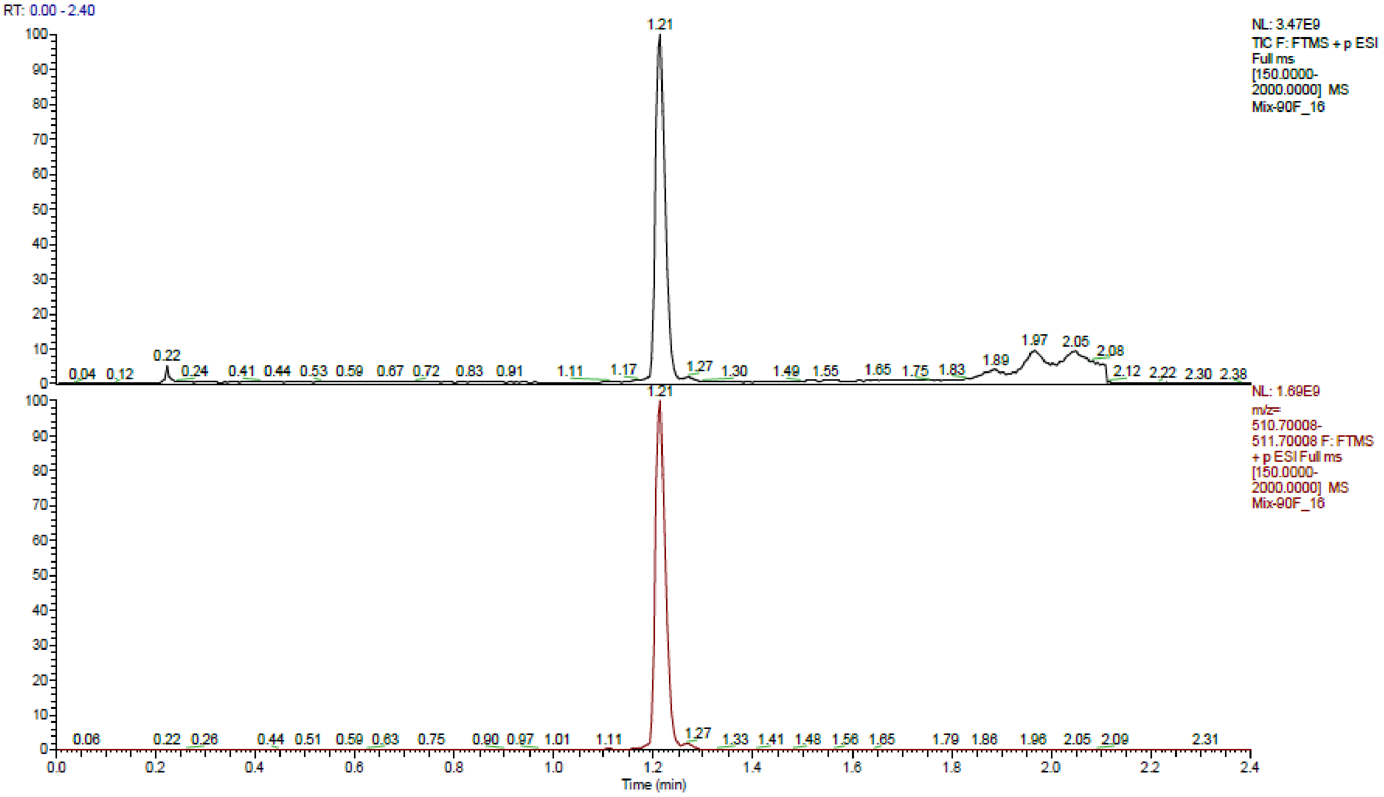

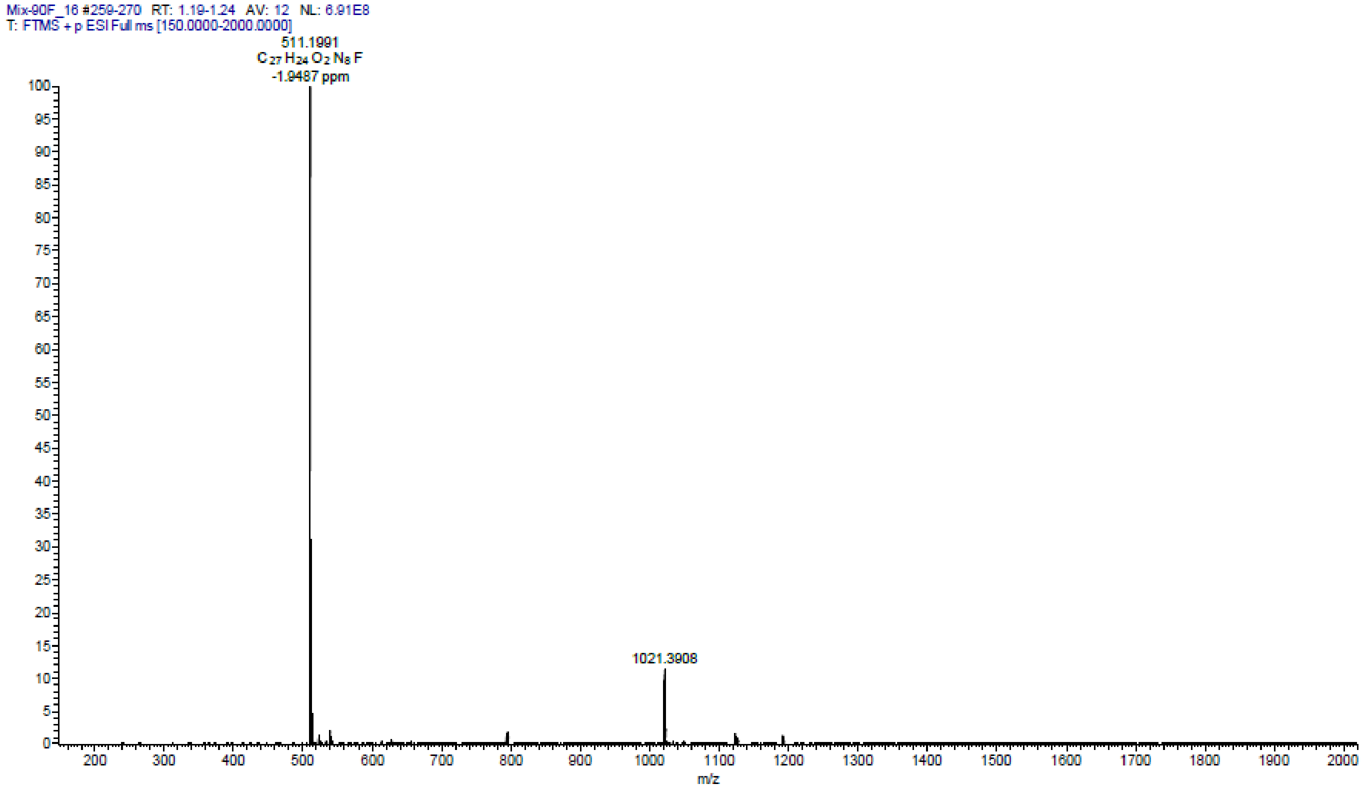

Compound 18a

**title**	**PINI22-80-1**
**Method**	AN_ACID.M
**Date acquired**	29-Jun-18, 18:00:30
**FileName**	Analysis\LCMS6_0629_074.D
**Column**	XSelect CSH C18 (50x2.1mm, 3.5µ) valve:3
**Flow**	0.8 ml/min; Column temp: 35°C
**Eluent A**	0.1% formic acid in acetonitrile
**Eluent B**	0.1% formic acid in water
**Gradient**	t=0 min 5% A, t=3.5 min 98% A, t=6 min 98%A
**Posttime**	2 min
**Detection**	DAD(210, 220 and 220-320nm)
**Detection**	PDA(210-320nm)
**Detection**	MSD (ESI pos/neg) mass range: 100 - 1000
**Detection**	ELSD gas temp: 40°C, flow 1.5 ml/min, gain 1

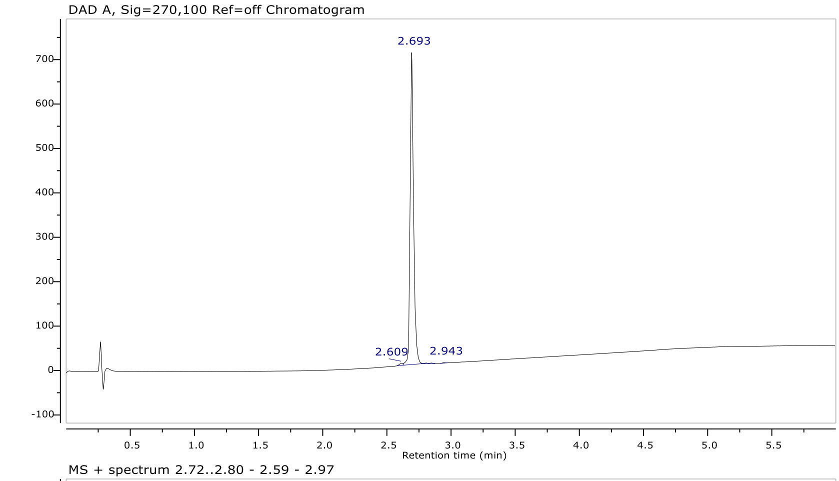

Integrals spectrum **Chromatogram DAD A, Sig=270,100 Ref=off**

rt (min)	height	area	area (%)
2.61	4.230	0.1266	0.58
2.69	702.3	21.52	99.02
2.80	1.392	0.02884	0.13
2.85	1.272	0.02770	0.13
2.94	1.254	0.03079	0.14

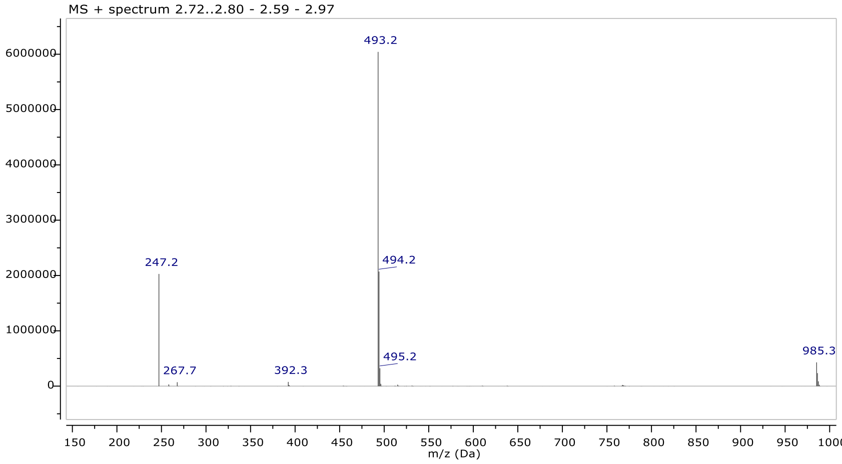

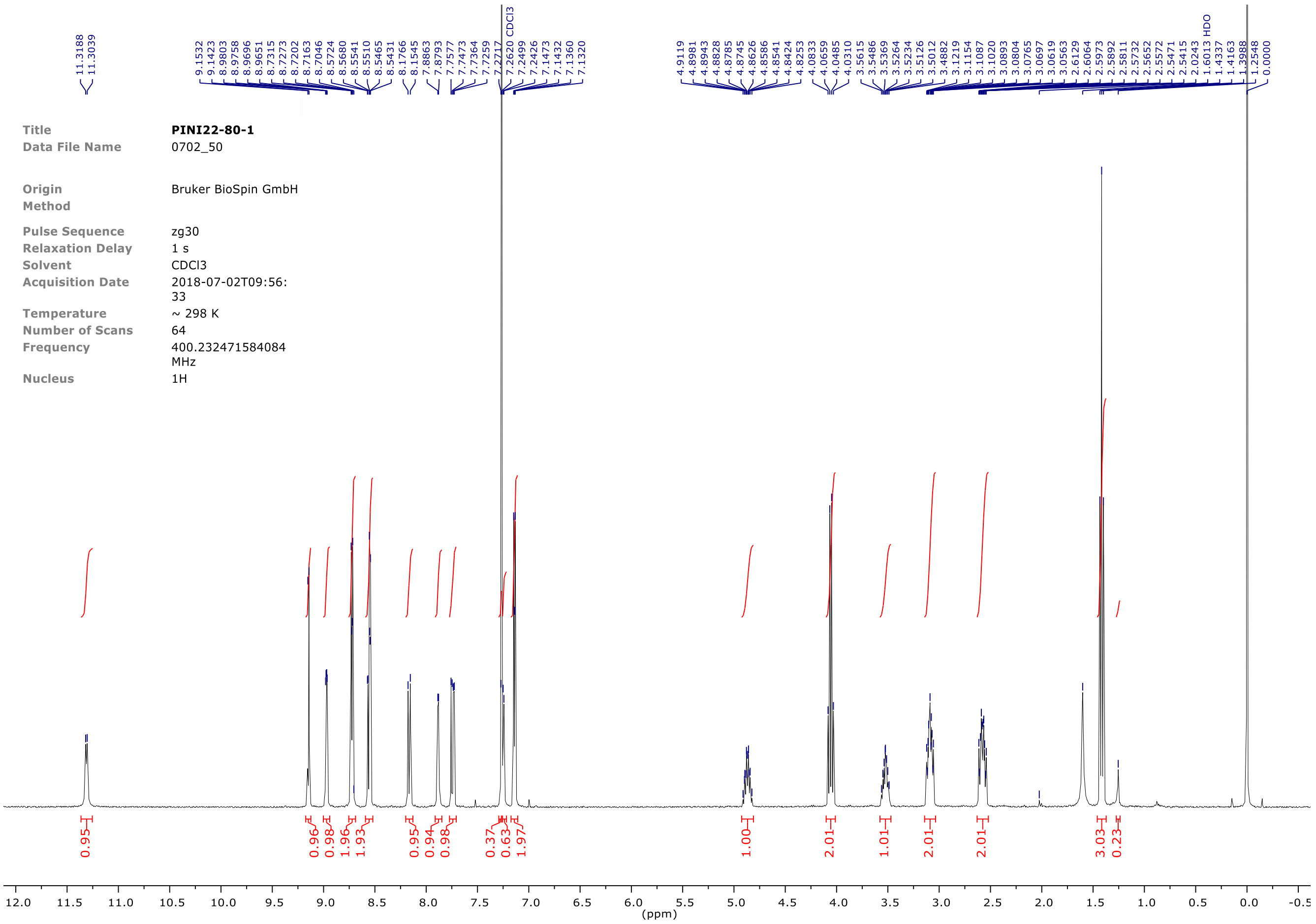

**Title**	**PINI22-80-1**
**Data File Name**	0702_50
**Origin**	Bruker BioSpin GmbH
**Method**	
**Pulse Sequence**	zg30
**Relaxation Delay**	1 s
**Solvent**	CDCl3
**Acquisition Date**	2018-07-02T09:56:33
**Temperature**	~ 298 K
**Number of Scans**	64
**Frequency**	400.232471584084 MHz
**Nucleus**	1H

HRMS (m/z): C27H24N8O2, [M+H]+ Calc: 493.20950; found: 493.2084, Δppm -2.16

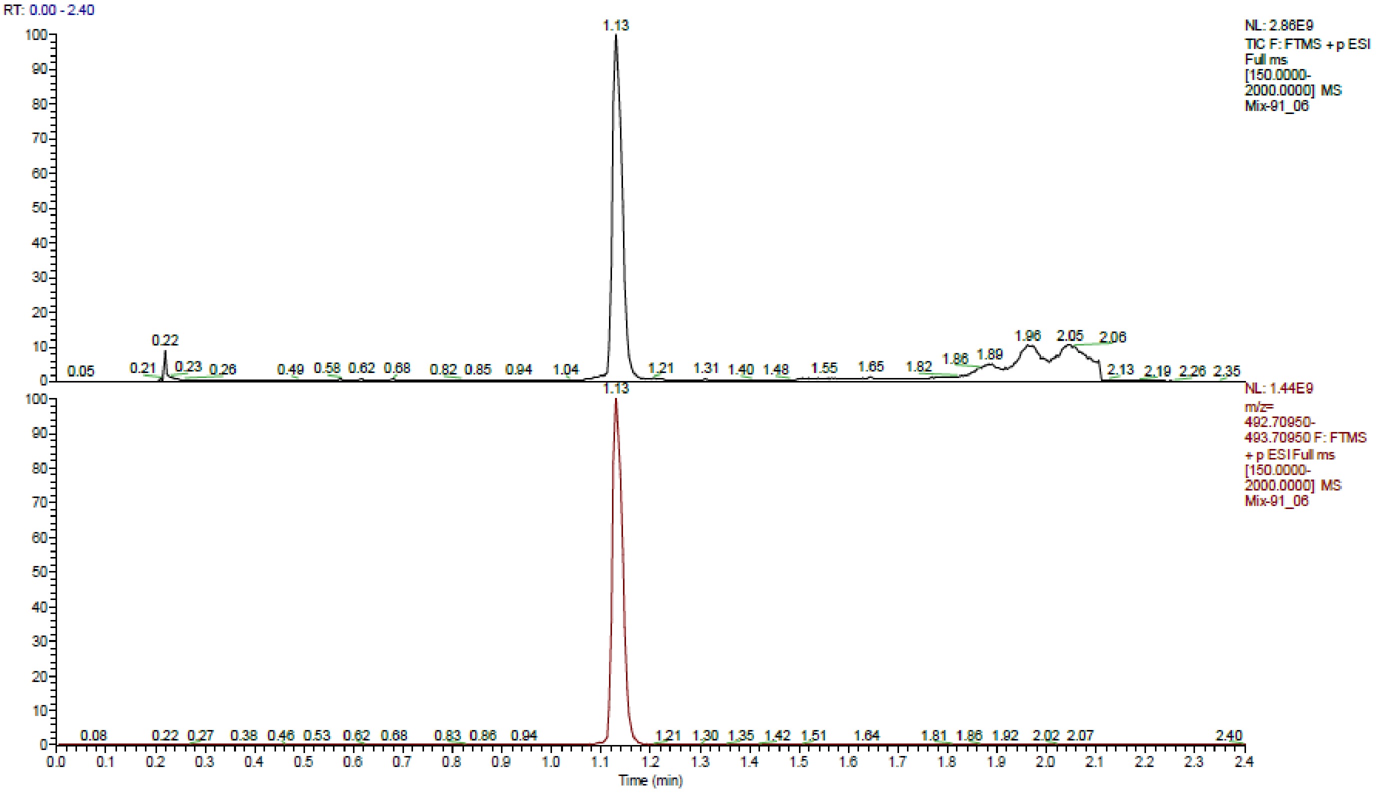

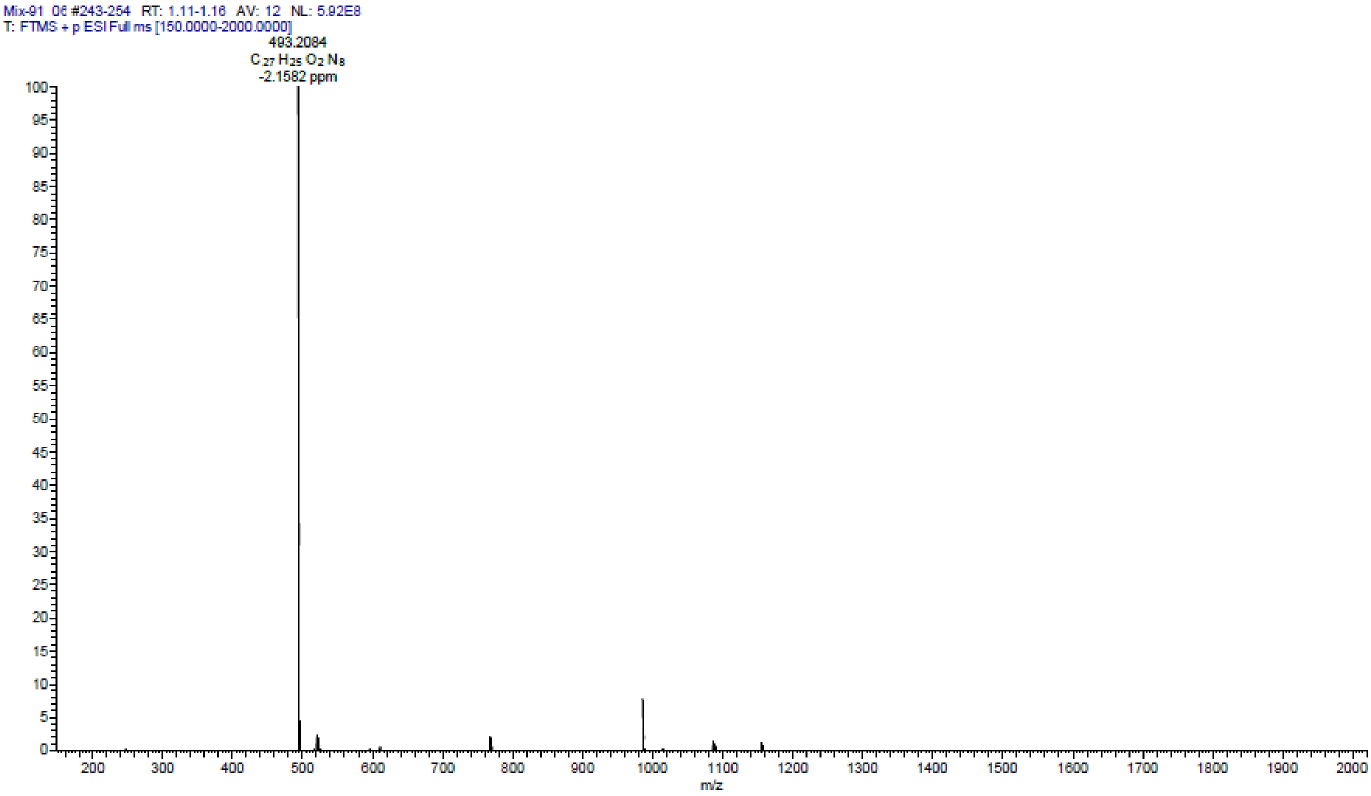

Compound 18b

**title**	**PINI23-47-1**
**Method**	AN_ACID.M
**Date acquired**	04-Oct-18, 16:49:04
**FileName**	Analysis\LCMS6_1004_074.D
**Column**	XSelect CSH C18 (50x2.1mm, 3.5μ) valve:3
**Flow**	0.8 ml/min; Column temp: 35°C
**Eluent A**	0.1% formic acid in acetonitrile
**Eluent B**	0.1% formic acid in water
**Gradient**	t=0 min 5% A, t=3.5 min 98% A, t=6 min 98%A
**Posttime**	2 min
**Detection**	DAD(210, 220 and 220-320nm)
**Detection**	PDA(210-320nm)
**Detection**	MSD (ESI pos/neg) mass range: 100 - 1000
**Detection**	ELSD gas temp: 40°C, flow 1.5 ml/min, gain 1

DAD A, Sig=270,100 Ref=off Chromatogram

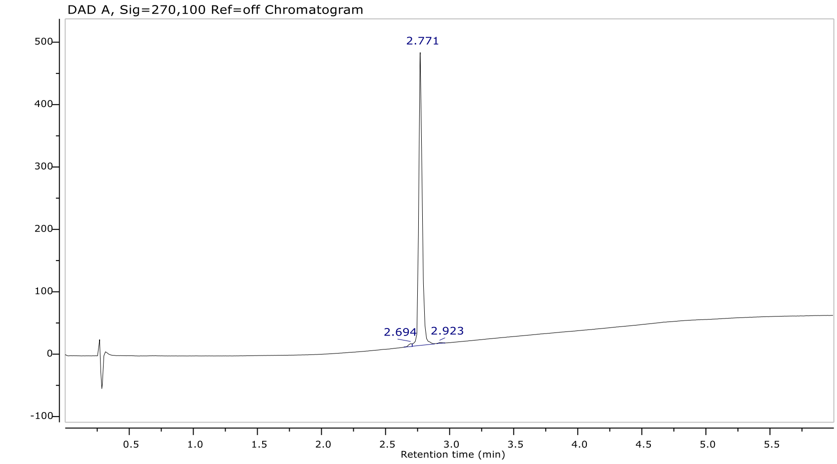

Integrals spectrum **Chromatogram DAD A, Sig=270,100 Ref=off**

rt (min)	height	area	area (%)
2.69	4.626	0.1446	0.98
2.77	469.5	14.57	98.77
2.92	1.248	0.03730	0.25

MS + spectrum 2.79..2.88

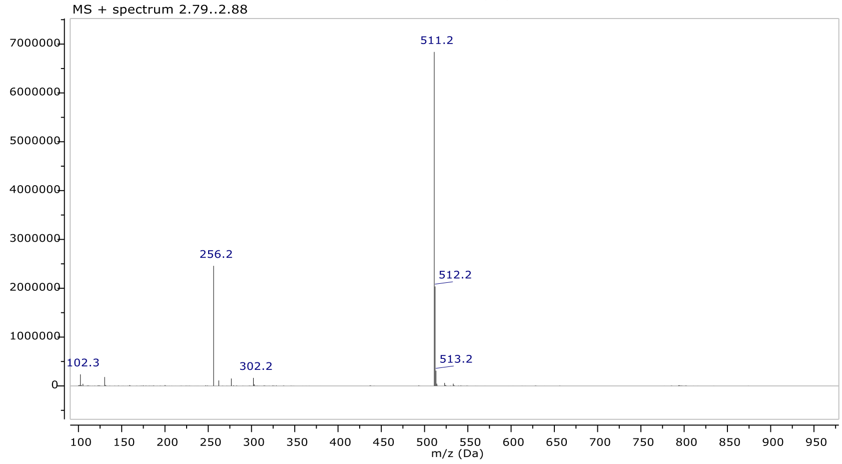

**Title**	**PINI23-47-1**
**Data File Name**	1005_160
**Origin**	Bruker BioSpin GmbH
**Method**	
**Pulse Sequence**	zg30
**Relaxation Delay**	1 s
**Solvent**	CDCl3
**Acquisition Date**	2018-10-05T11:28:28
**Temperature**	~ 298 K
**Number of Scans**	32
**Frequency**	400.232471584084 MHz
**Nucleus**	1H

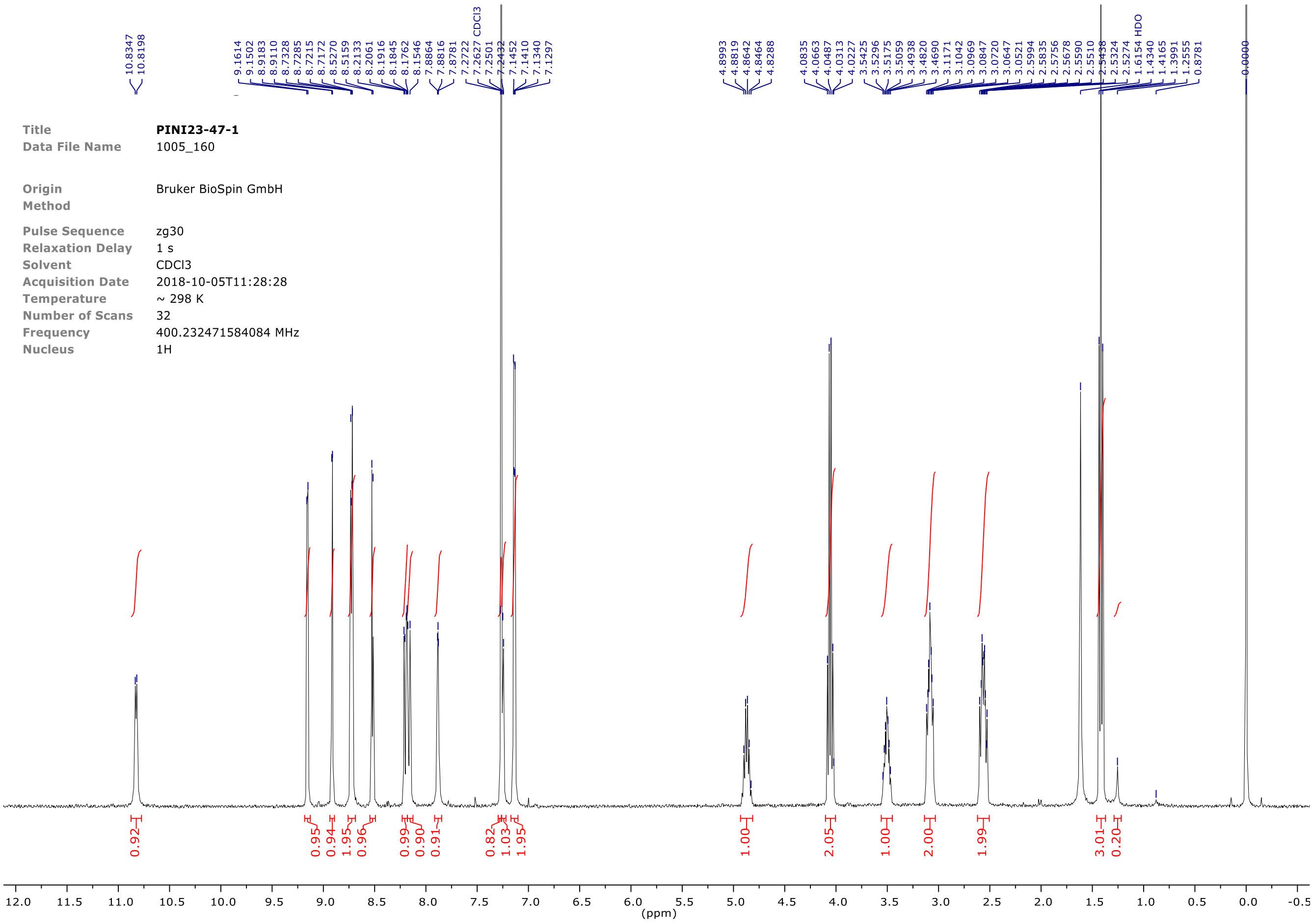

HRMS (m/z): C27H23N8O2F, [M+H]+ Calc: 511.20008; found: 511.1992, Δppm -1.69

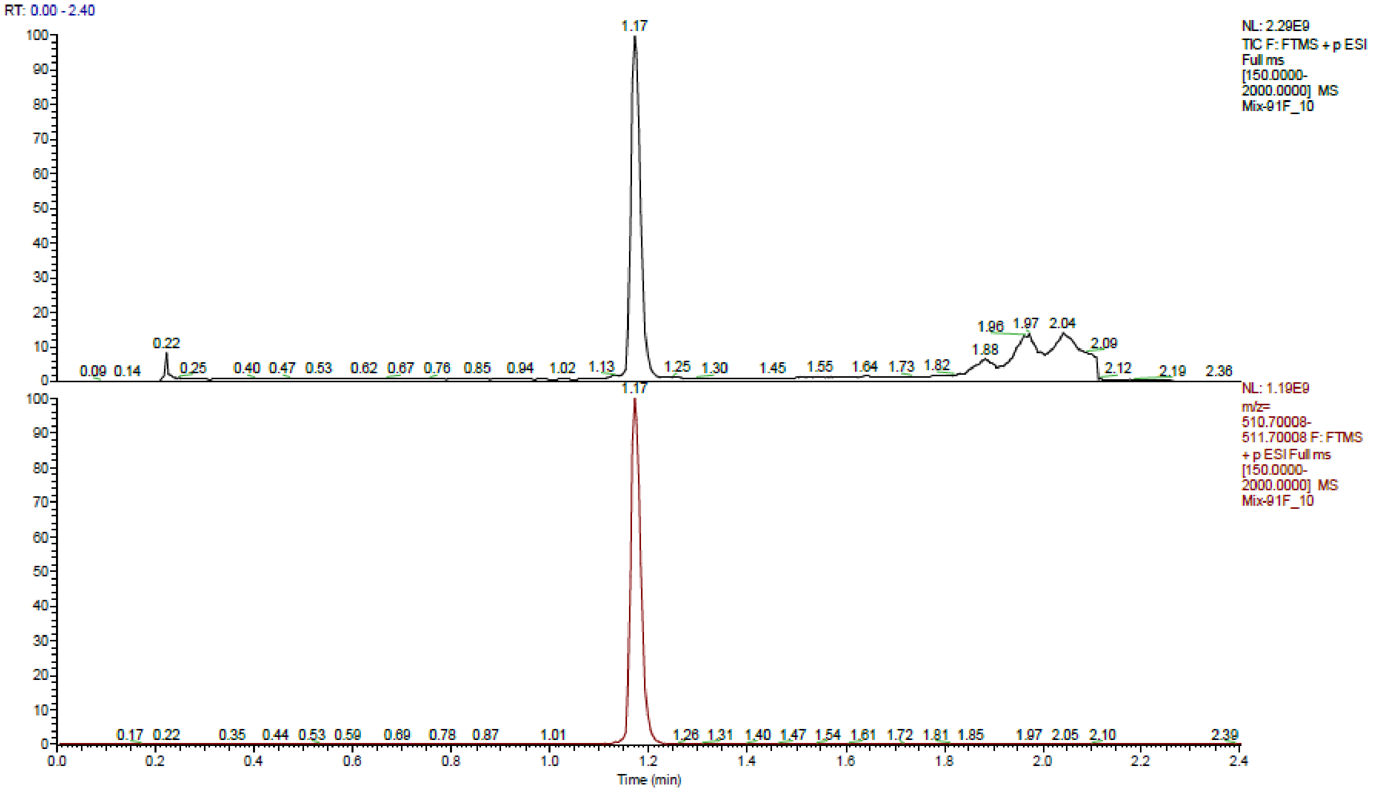

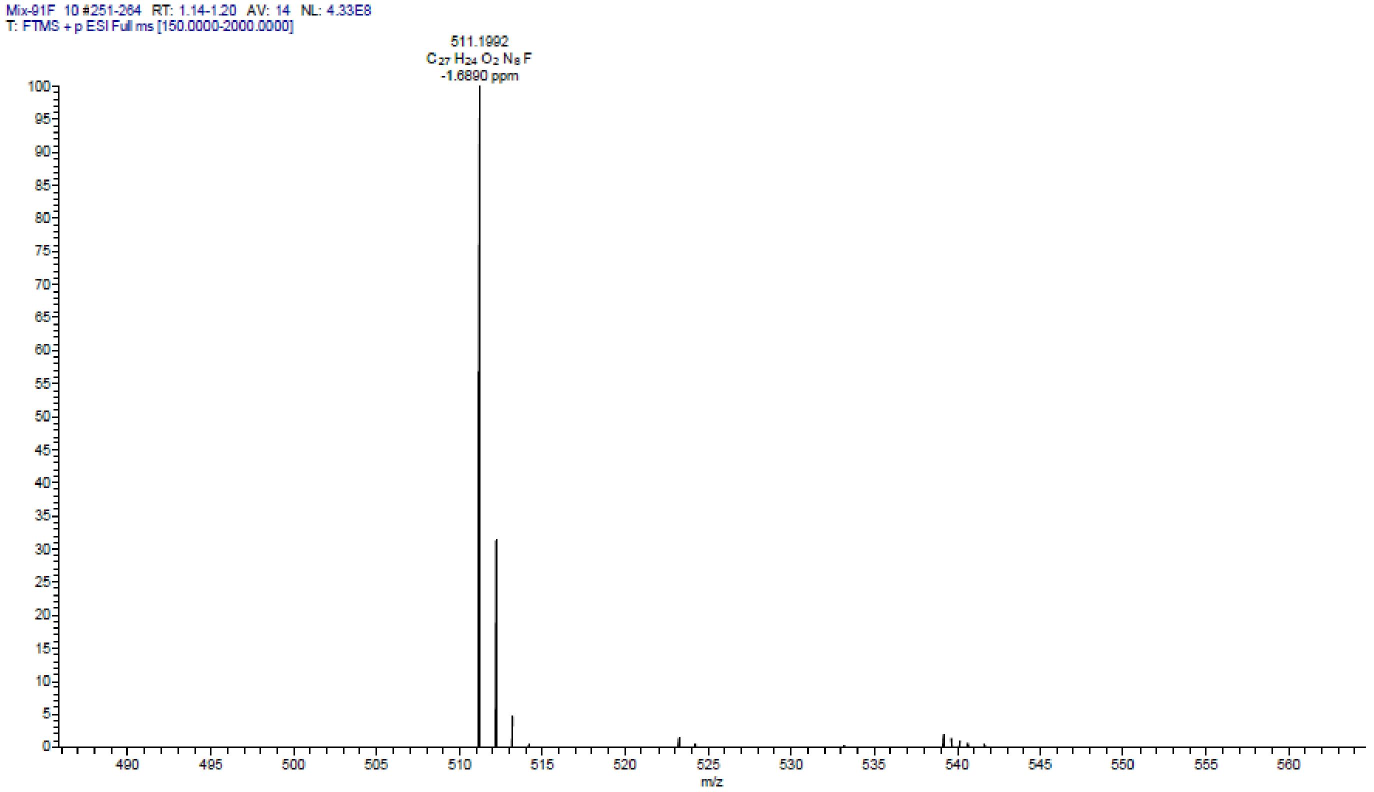

Compound 19

**title**	**PINI22-33-1**
**Method**	AN_ACID.M
**Date acquired**	30-Apr-18, 07:47:50
**FileName**	Analysis\LCMS6_0430_006.D
**Column**	XSelect CSH C18 (50x2.1mm, 3.5µ) valve:3
**Flow**	0.8 ml/min; Column temp: 35°C
**Eluent A**	0.1% formic acid in acetonitrile
**Eluent B**	0.1% formic acid in water
**Gradient**	t=0 min 5% A, t=3.5 min 98% A, t=6 min 98%A
**Posttime**	2 min
**Detection**	DAD(210, 220 and 220-320nm)
**Detection**	PDA(210-320nm)
**Detection**	MSD (ESI pos/neg) mass range: 100 - 1000
**Detection**	ELSD gas temp: 40°C, flow 1.5 ml/min, gain 1

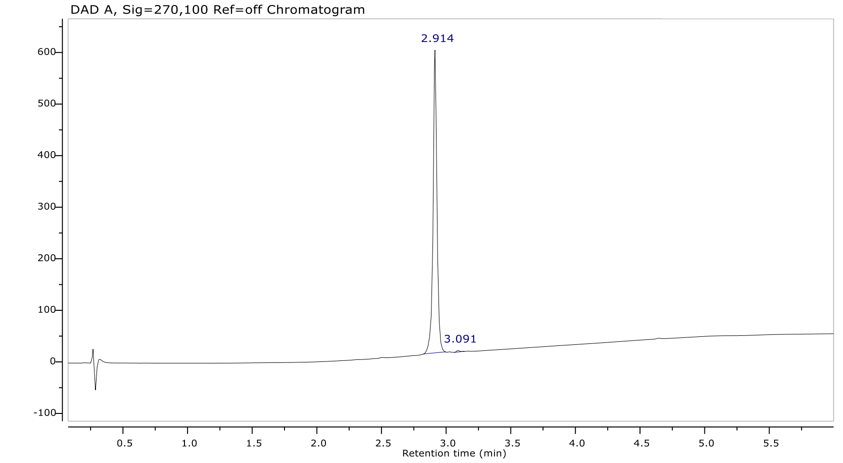

Integrals spectrum **Chromatogram DAD A, Sig=270,100 Ref=off**

rt (min)	height	area	area (%)
2.91	587.4	20.37	99.60
3.09	2.927	0.08126	0.40

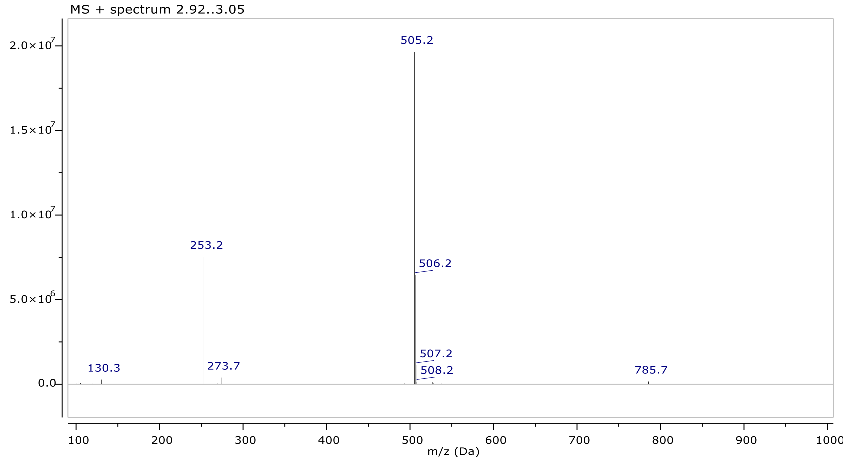

**Title**	**PINI22-33-1**
**Data File Name**	0426_260
**Origin**	Bruker BioSpin GmbH
**Method**	
**Pulse Sequence**	zg30
**Relaxation Delay**	1 s
**Solvent**	CDCl3
**Acquisition Date**	2018-04-26T15:07:51
**Temperature**	~ 293.2 K
**Number of Scans**	32
**Frequency**	400.232471584084 MHz
**Nucleus**	1H

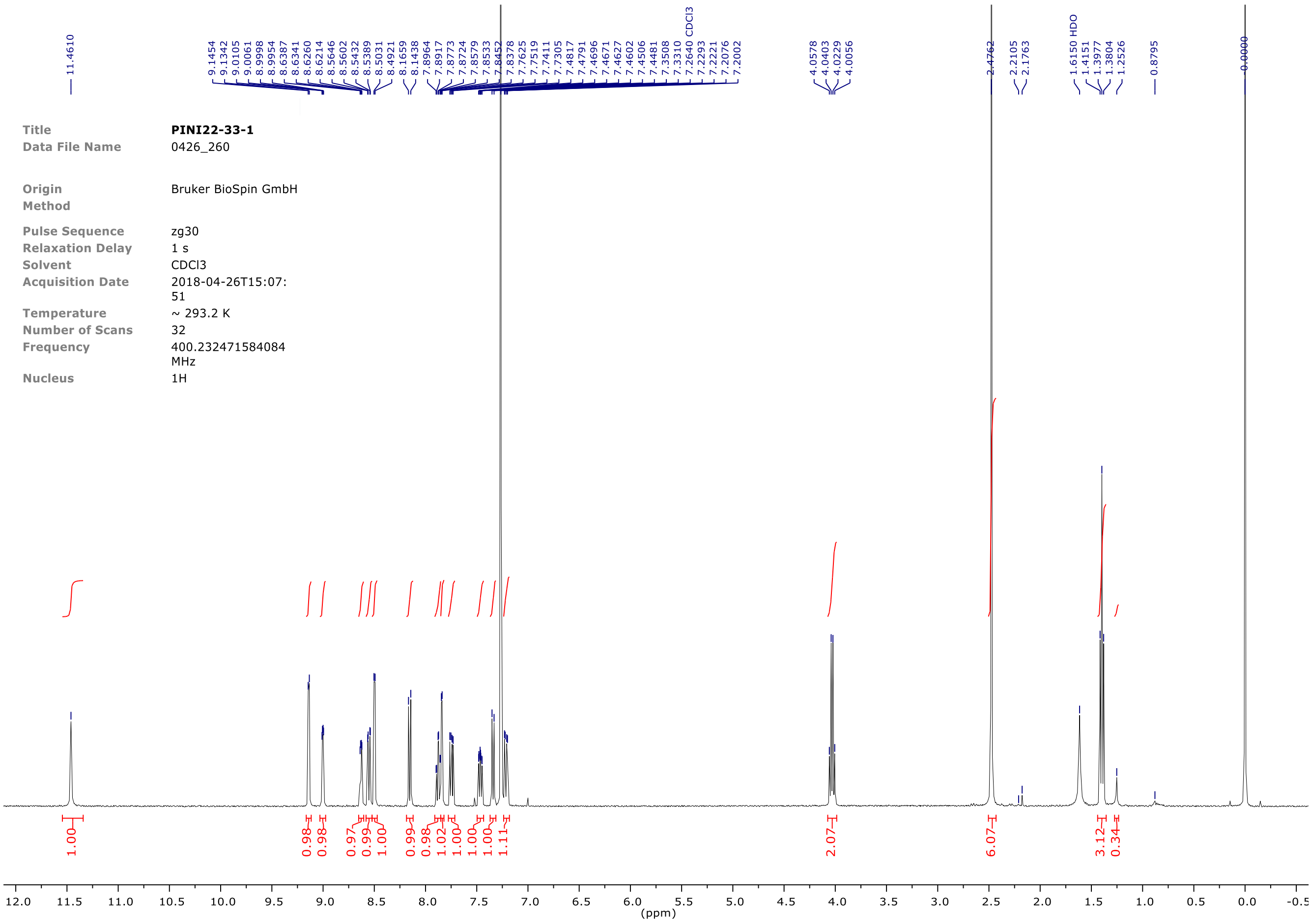

HRMS (m/z): C28H24N8O2, [M+H]+ Calc: 505.20950; found: 505.2086, Δppm -1.87

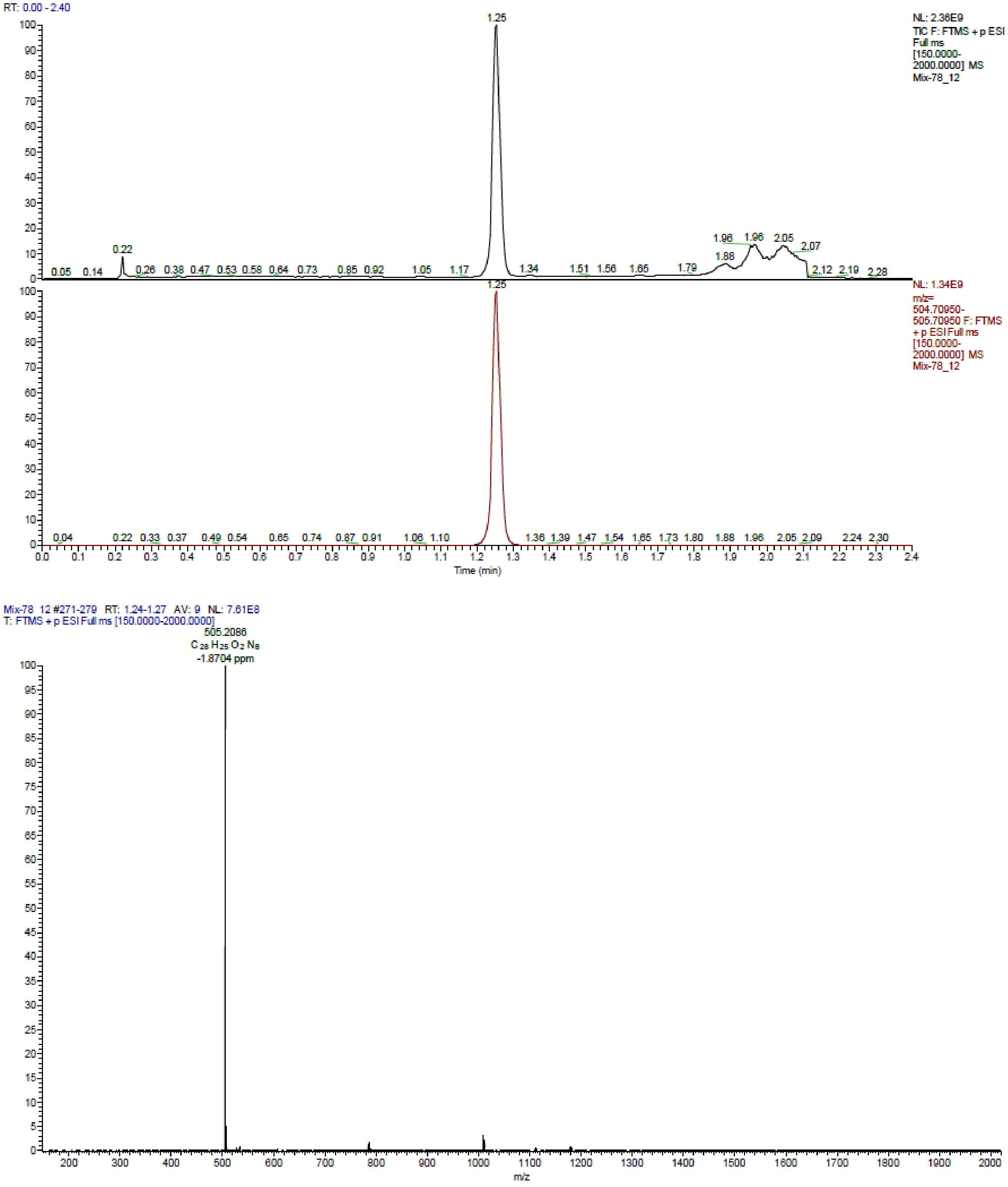

Compound 20

title	**PINI22-85-1**
Method	AN_ACID.M
Date acquired	05-Jul-18, 21:50:04
FileName	Analysis\LCMS6_0705_093.D
Column	XSelect CSH C18 (50x2.1mm, 3.5μ) valve:3
Flow	0.8 ml/min; Column temp: 35°C
Eluent A	0.1% formic acid in acetonitrile
Eluent B	0.1% formic acid in water
Gradient	t=0 min 5% A, t=3.5 min 98% A, t=6 min 98%A
Posttime	2 min
Detection	DAD(210, 220 and 220-320nm)
Detection	PDA(210-320nm)
Detection	MSD (ESI pos/neg) mass range: 100 - 1000
Detection	ELSD gas temp: 40°C, flow 1.5 ml/min, gain 1

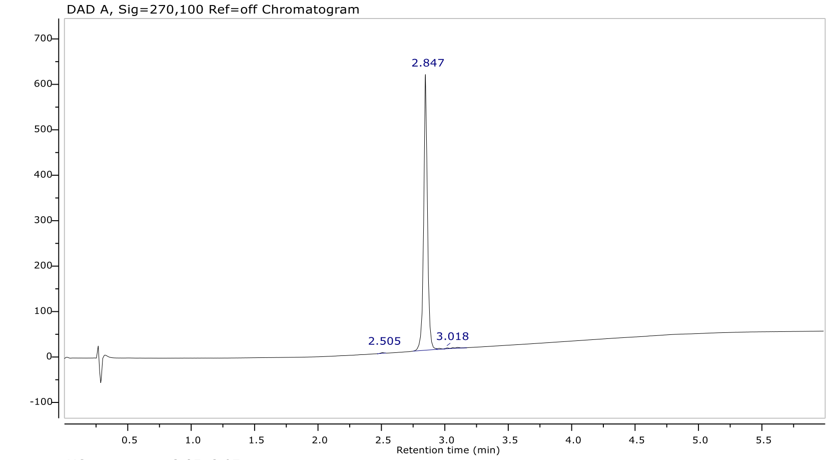

Integrals spectrum Chromatogram DAD A, Sig=270,100 Ref=off

rt (min)	height	area	area (%)
2.50	1.644	0.04149	0.20
2.85	607.0	20.92	98.75
2.96	2.220	0.07104	0.34
3.02	1.936	0.1515	0.72

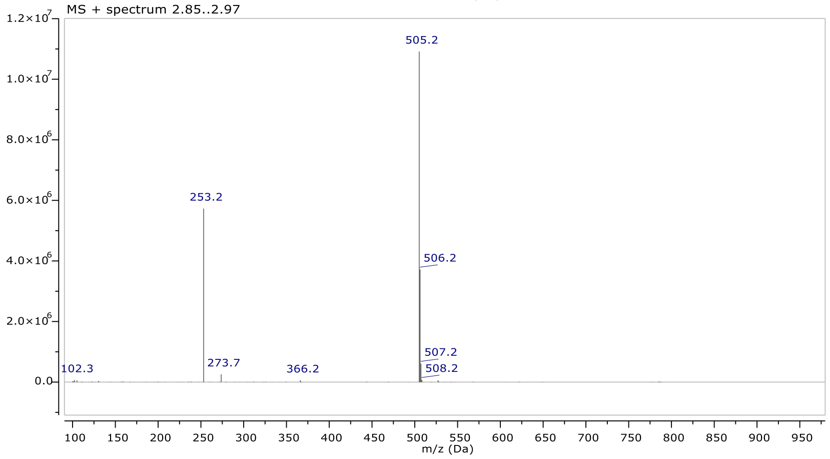

Title	**PINI22-85-1**
Data File Name	0706_100
Origin	Bruker BioSpin GmbH
Method	
Pulse Sequence	zg30
Relaxation Delay	1 s
Solvent	CDCl3
Acquisition Date	2018-07-06T10:08:46
Temperature	~ 298 K
Number of Scans	16
Frequency	400.232471584084 MHz
Nucleus	1H

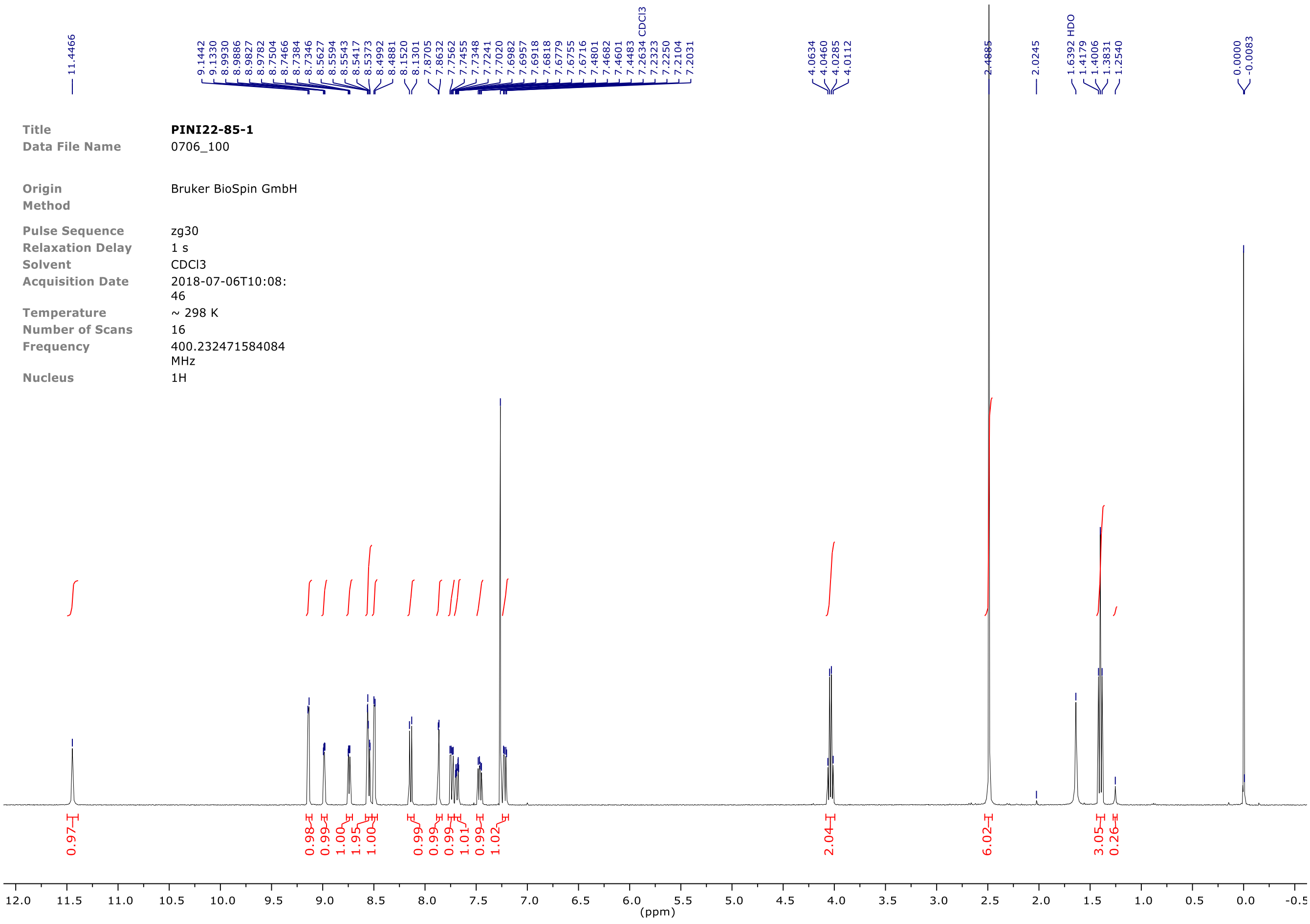

HRMS (m/z): C28H24N8O2, [M+H]+ Calc: 505.20950; found: 505.2085, Δppm -2.01

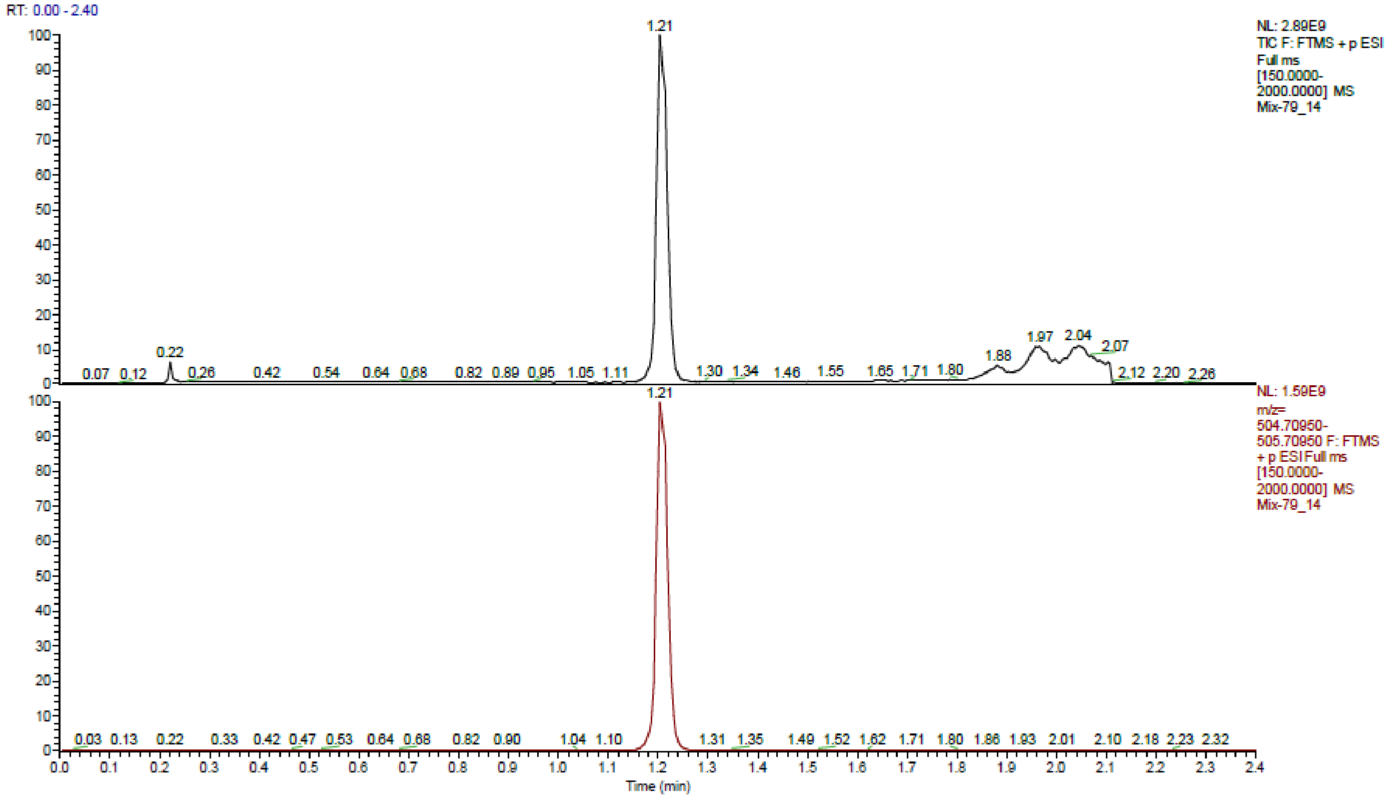

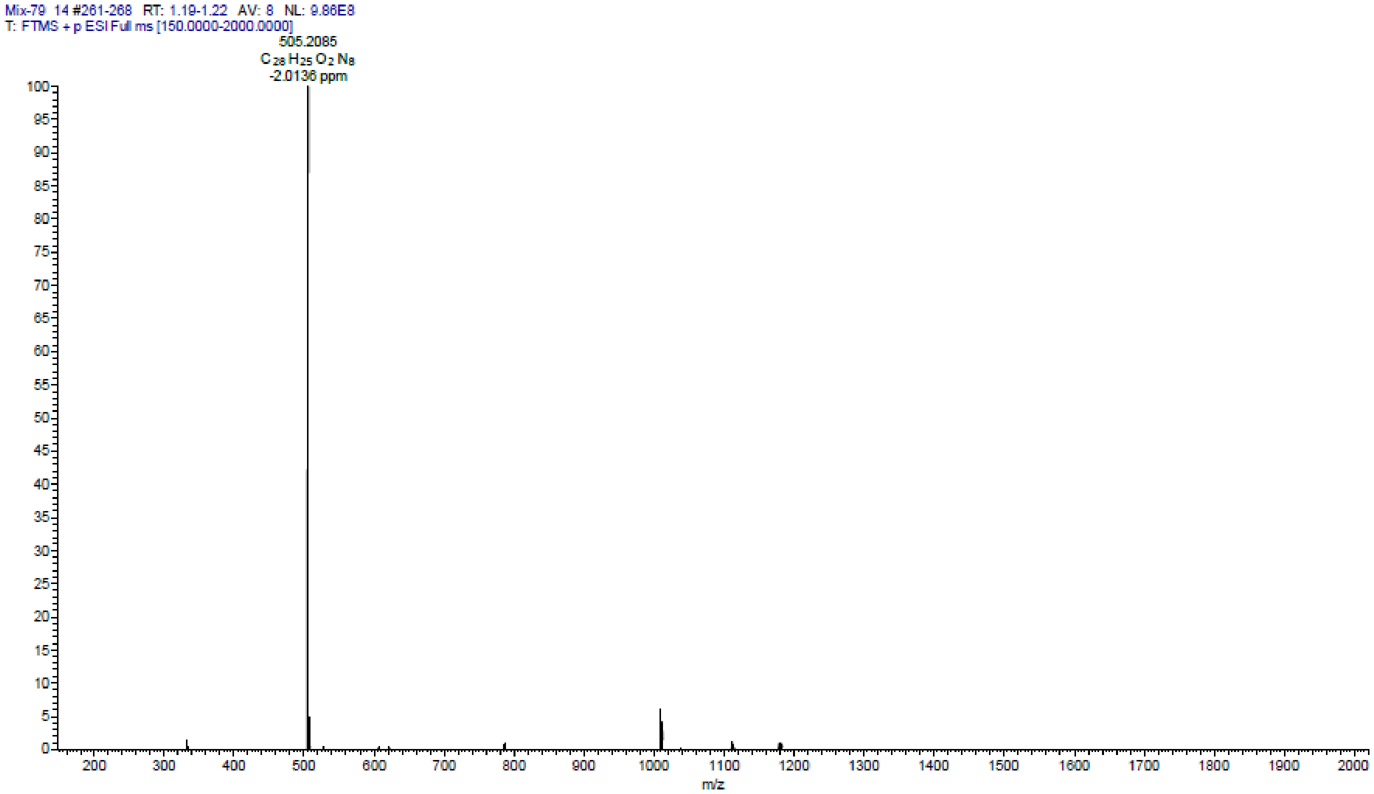

Compound 21

**title**	**PINI22-86-1**
**Method**	AN_ACID.M
**Date acquired**	05-Jul-18, 21:58:27
**FileName**	Analysis\LCMS6_0705_094.D
**Column**	XSelect CSH C18 (50x2.1mm, 3.5μ) valve:3
**Flow**	0.8 ml/min; Column temp: 35°C
**Eluent A**	0.1% formic acid in acetonitrile
**Eluent B**	0.1% formic acid in water
**Gradient**	t=0 min 5% A, t=3.5 min 98% A, t=6 min 98%A
**Posttime**	2 min
**Detection**	DAD(210, 220 and 220-320nm)
**Detection**	PDA(210-320nm)
**Detection**	MSD (ESI pos/neg) mass range: 100 - 1000
**Detection**	ELSD gas temp: 40°C, flow 1.5 ml/min, gain 1

DAD A, Sig=270,100 Ref=off Chromatogram

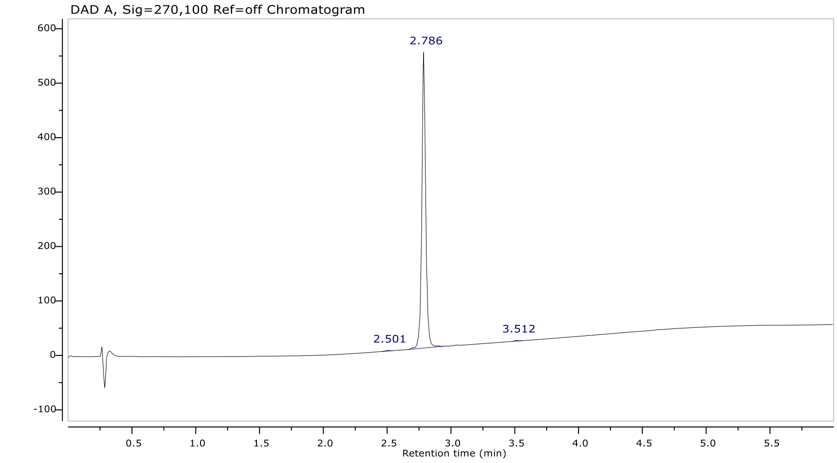

Integrals spectrum — **Chromatogram DAD A, Sig=270,100 Ref=off**

rt (min)	height	area	area (%)
2.50	1.141	0.04251	0.22
2.79	543.3	19.08	99.61
3.51	1.156	0.03178	0.17

MS + spectrum 2.79..2.95

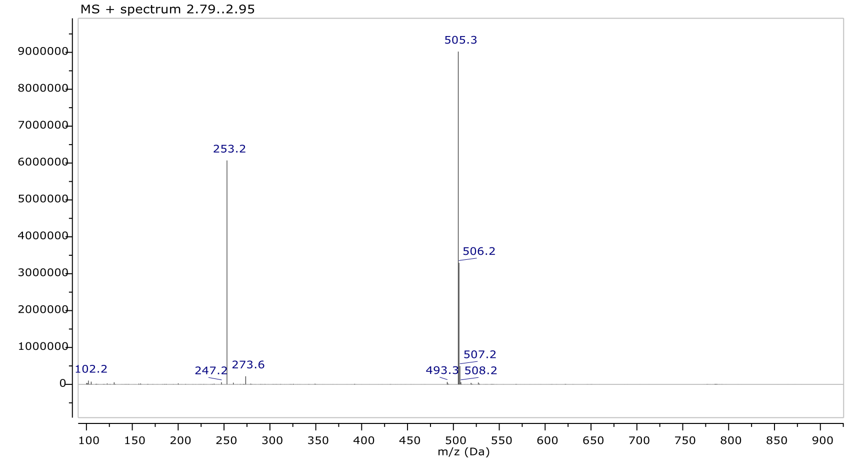

**Title**	**PINI22-86-1**
**Data File Name**	0706_110
**Origin**	Bruker BioSpin GmbH
**Method**	
**Pulse Sequence**	zg30
**Relaxation Delay**	1 s
**Solvent**	CDCl3
**Acquisition Date**	2018-07-06T10:21:18
**Temperature**	~ 298 K
**Number of Scans**	64
**Frequency**	400.232471584084 MHz
**Nucleus**	1H

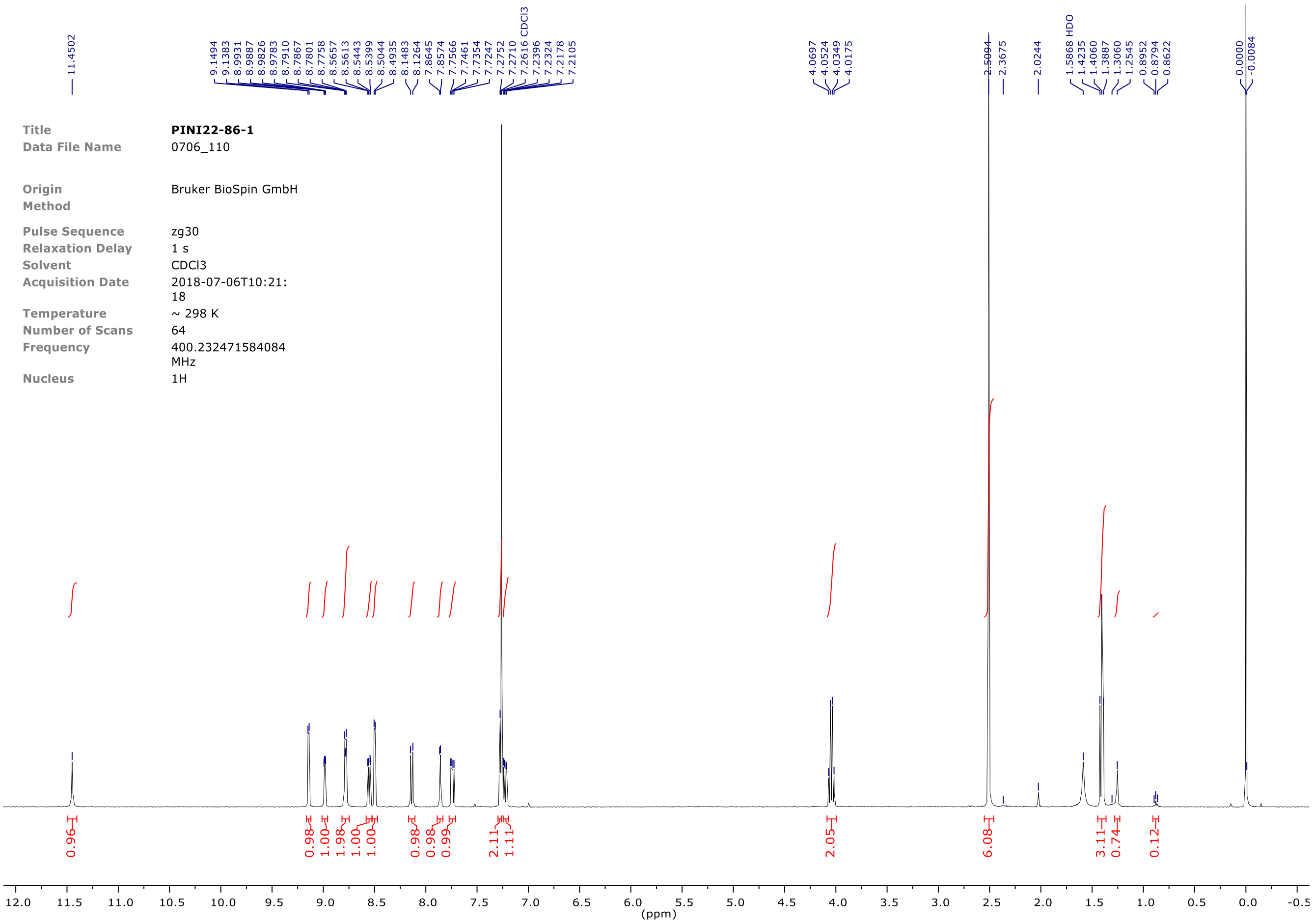

HRMS (m/z): C28H24N8O2, [M+H]+ Calc: 505.20950; found: 505.2085, Δppm -2.02

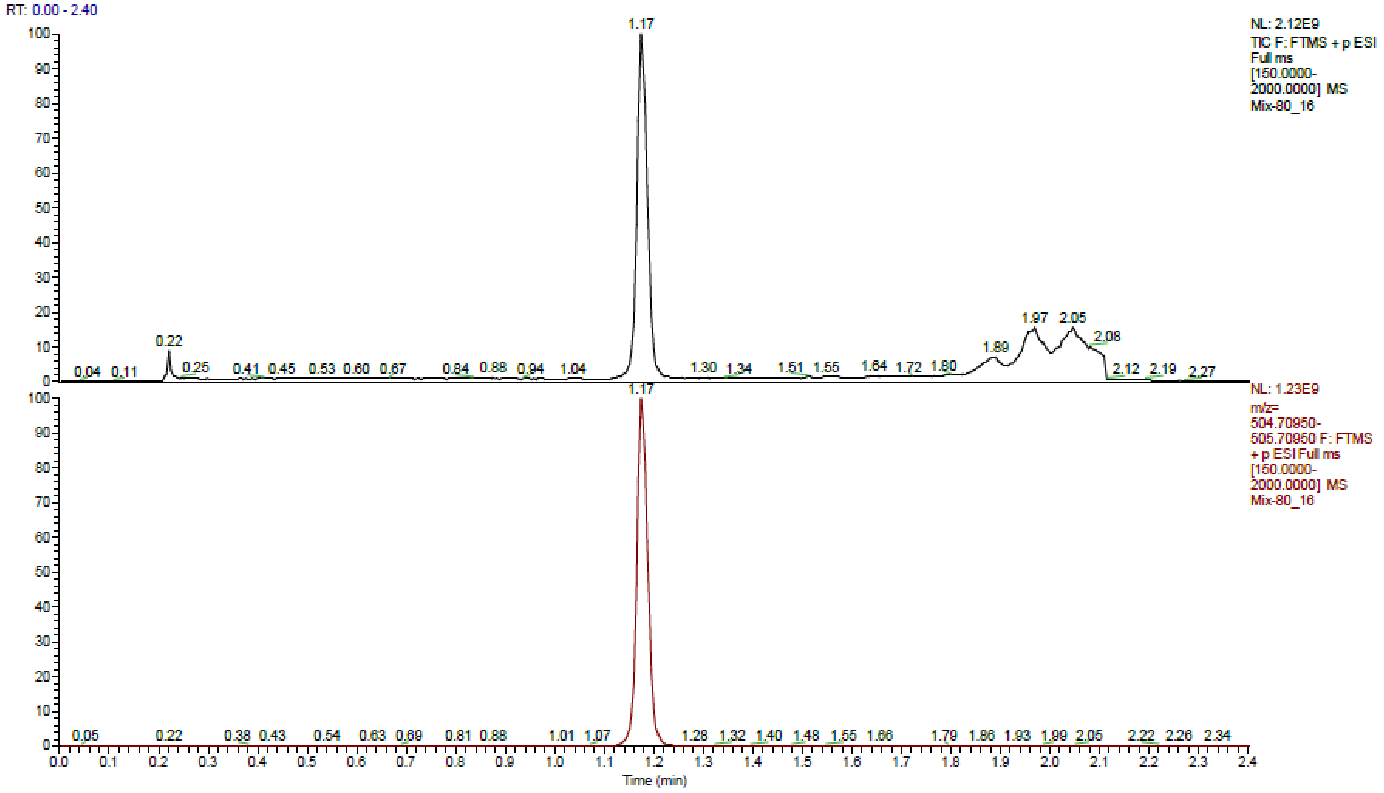

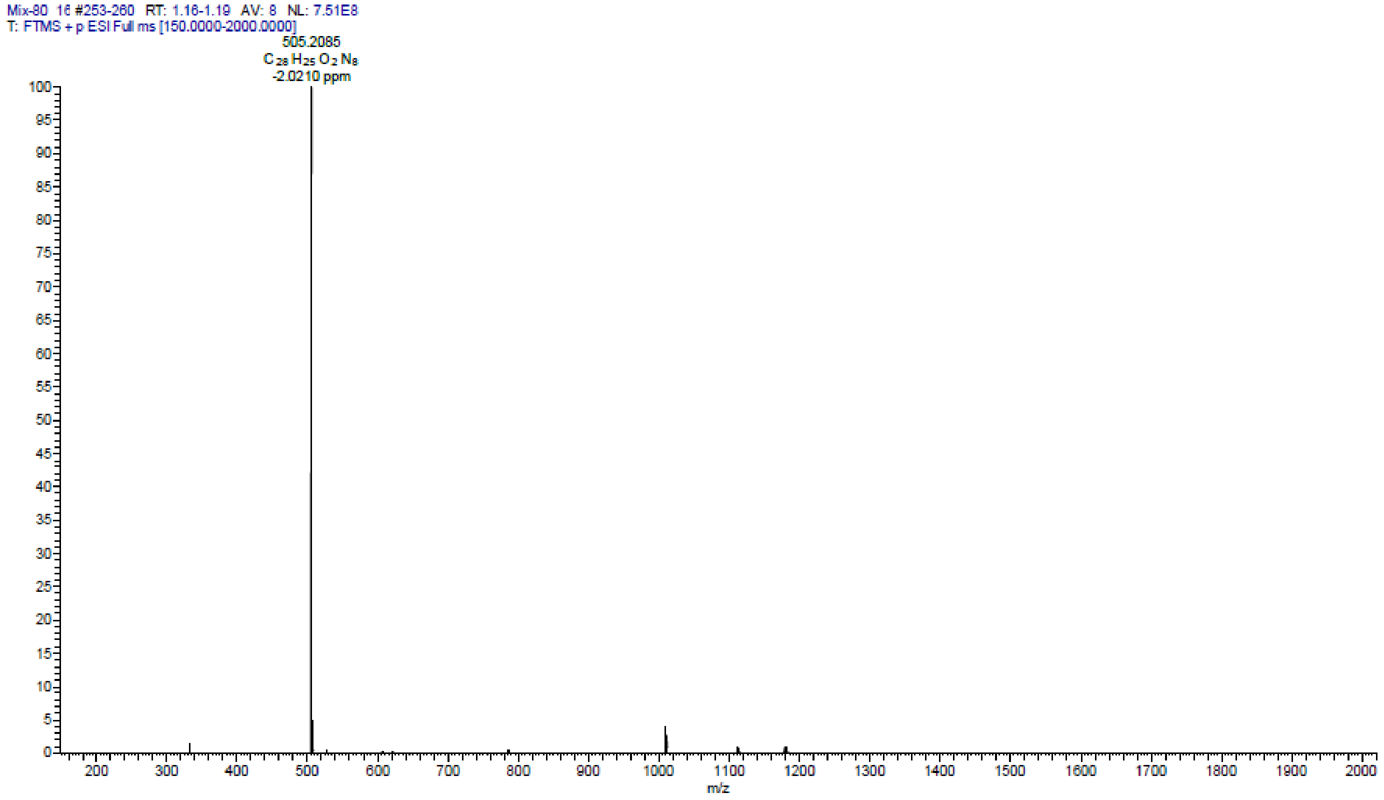

Compound 22a

**title**	**PINI23-82-1**
**Method**	AN_ACID.M
**Date acquired**	06-Dec-18, 17:29:41
**FileName**	Analysis\LCMS6_1206_116.D
**Column**	XSelect CSH C18 (50x2.1mm, 3.5µ) valve:3
**Flow**	0.8 ml/min; Column temp: 35°C
**Eluent A**	0.1% formic acid in acetonitrile
**Eluent B**	0.1% formic acid in water
**Gradient**	t=0 min 5% A, t=3.5 min 98% A, t=6 min 98%A
**Posttime**	2 min
**Detection**	DAD(210, 220 and 220-320nm)
**Detection**	PDA(210-320nm)
**Detection**	MSD (ESI pos/neg) mass range: 100 - 1000
**Detection**	ELSD gas temp: 40°C, flow 1.5 ml/min, gain 1

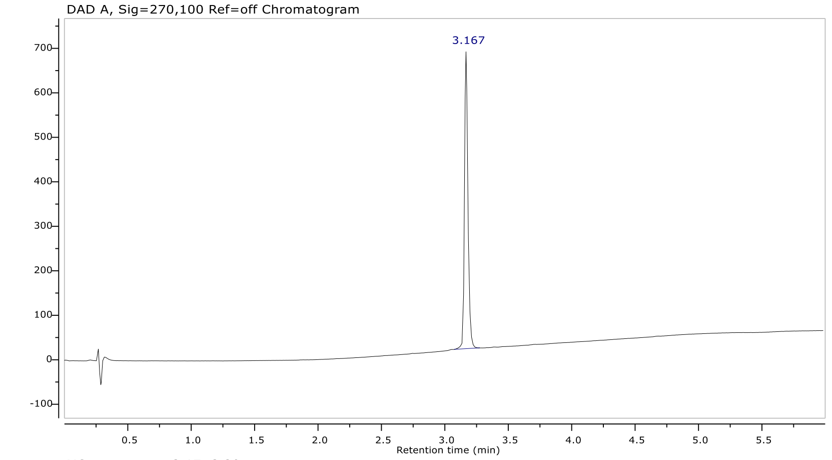

Integrals spectrum Chromatogram DAD A, Sig=270,100 Ref=off

rt (min)	height	area	area (%)
3.17	667.3	20.99	100.00

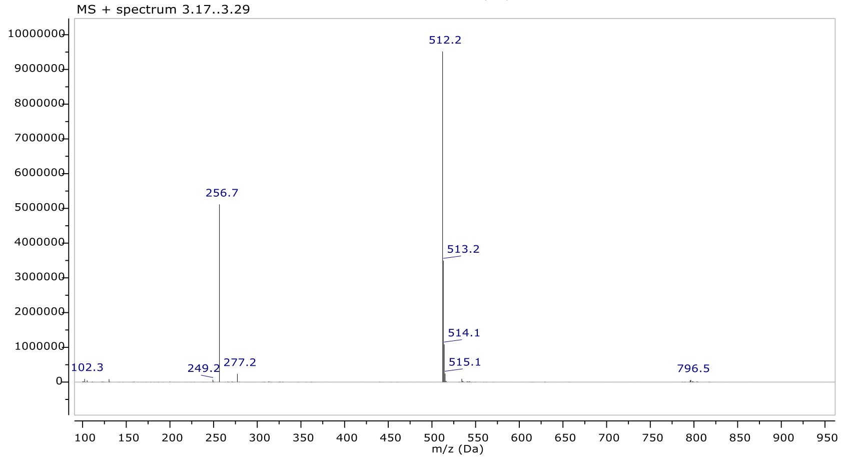

**Title**	**PINI23-82-1**
**Data File Name**	1207_20
**Origin**	Bruker BioSpin GmbH
**Method**	
**Pulse Sequence**	zg30
**Relaxation Delay**	1 s
**Solvent**	CDCl3
**Acquisition Date**	2018-12-07T08:50:41
**Temperature**	~ 294.06 K
**Number of Scans**	32
**Frequency**	400.232471584084 MHz
**Nucleus**	1H

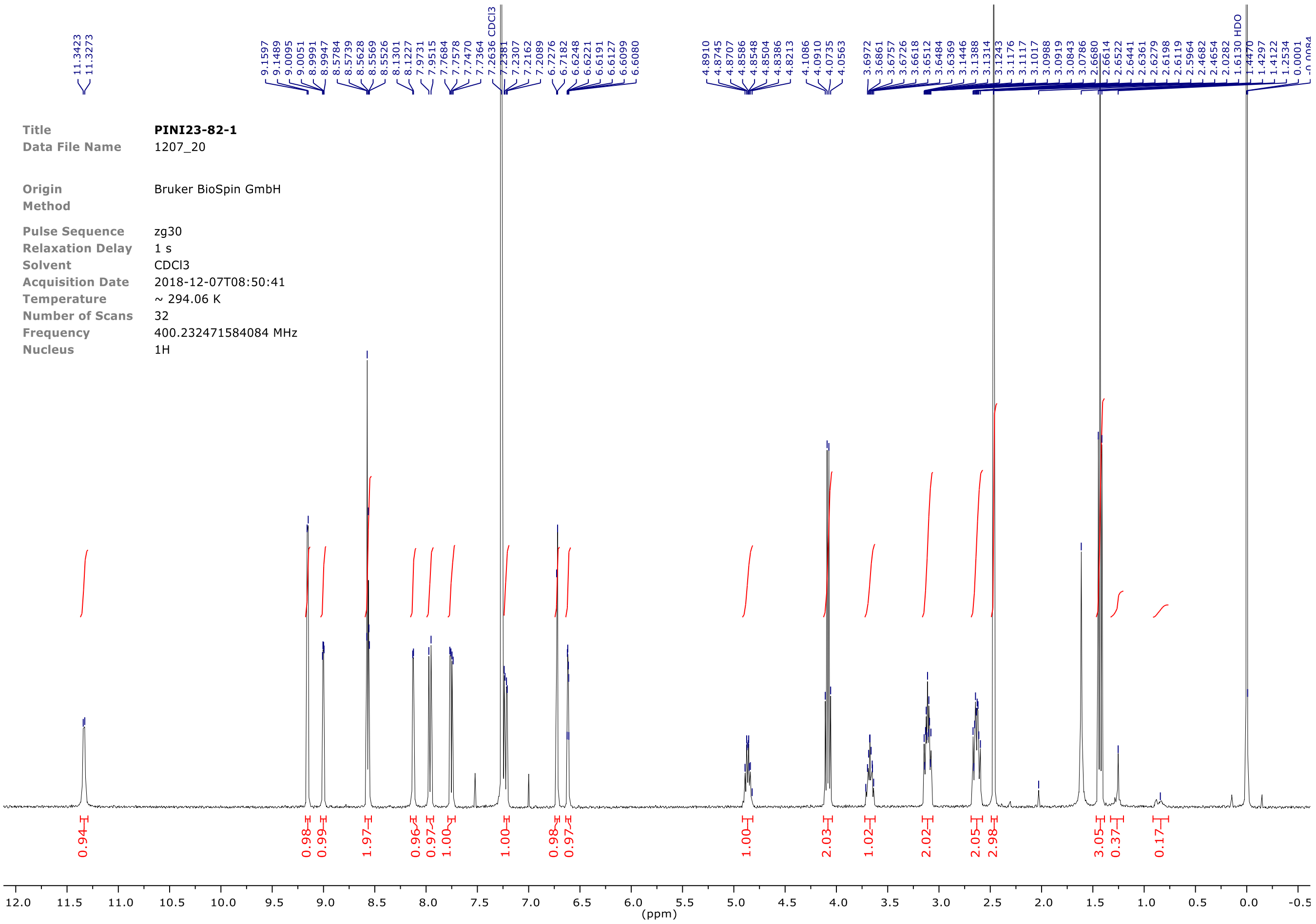

HRMS (m/z): C27H25N7O2S, [M+H]+ Calc: 512.18632; found: 512.1854, Δppm -1.73

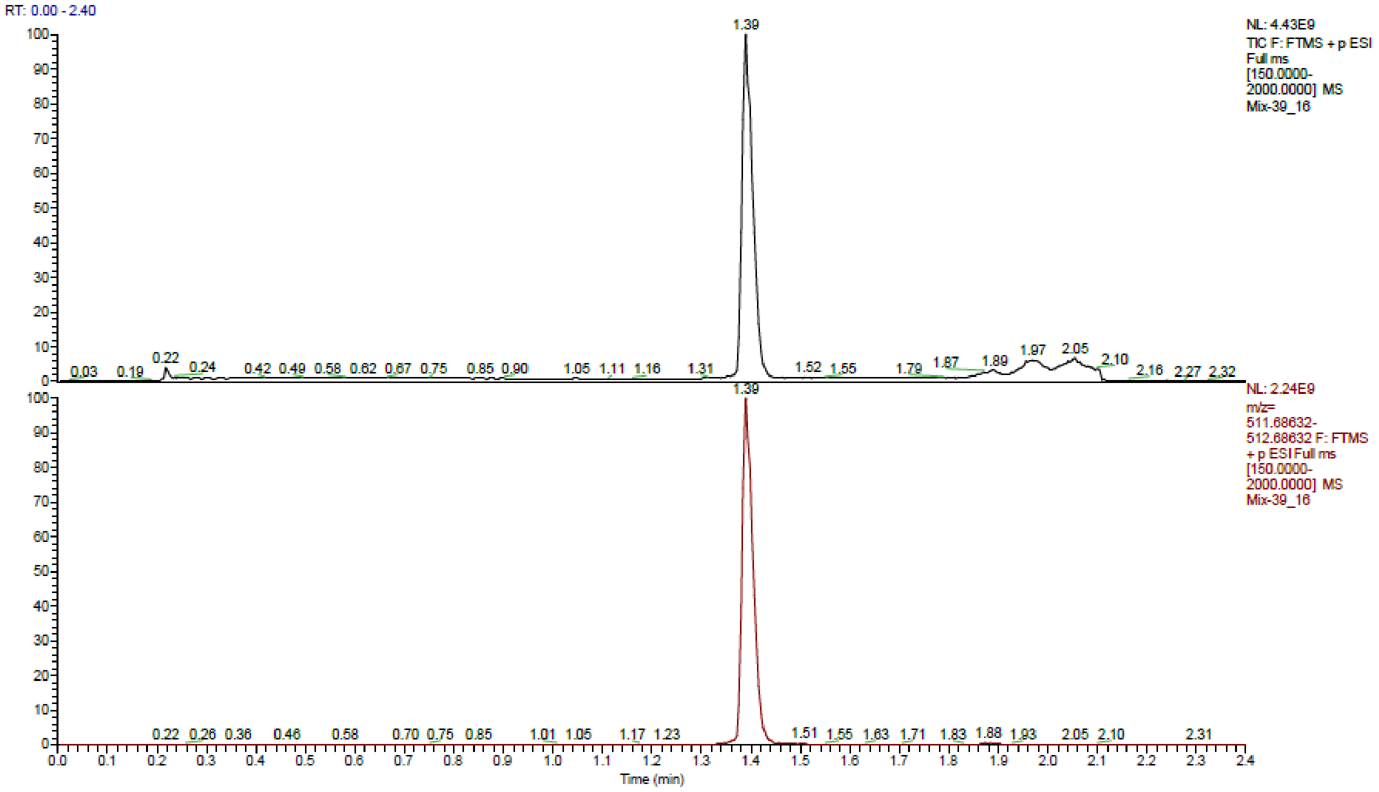

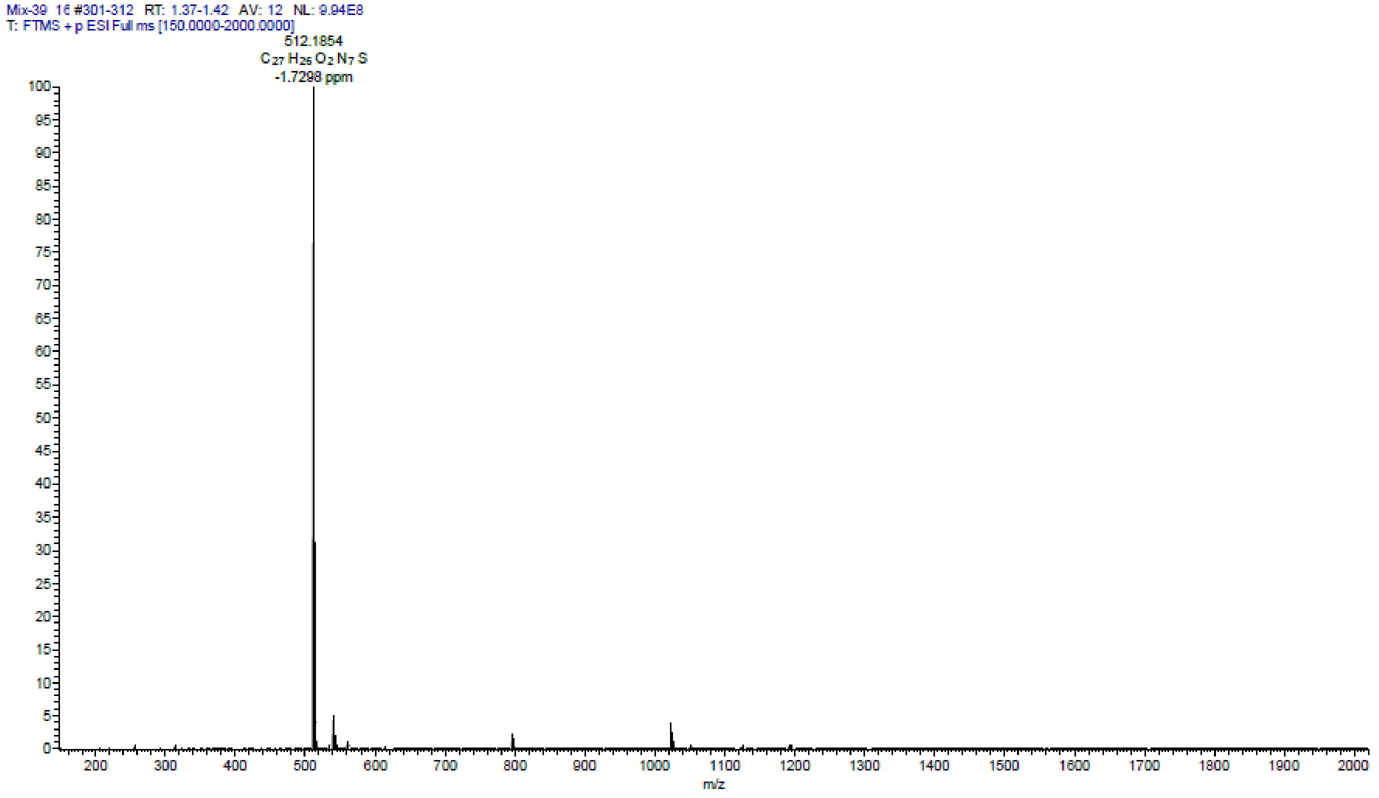

Compound 22b

**title**	**sjae 54 230-2**
**Method**	AN_BASE.M
**Date acquired**	11-Jan-18, 17:46:50
**FileName**	Analysis\LCMS5_0111_097.D
**Acq. method**	AN_BASE valve: 2
**Column**	Waters XSelect CSH C18 (50x2.1mm, 3.5µ)
**Flow**	0.8 ml/min; Column temp: 25°C
**Eluent A**	95% acetonitrile + 5% 10 mM ammoniumbicarbonate in water
**Eluent B**	10mM ammoniumbicarbonate in water
**Lin. Gradient**	t=0 min 5% A, t=3.5 min 98% A, t=6 min 98% A
**Posttime**	2 min
**Detection**	DAD (210, 220 and 220-320 nm)
**Detection**	PDA (210-320 nm)
**Detection**	MSD (ESI pos/neg) mass range: 100-800

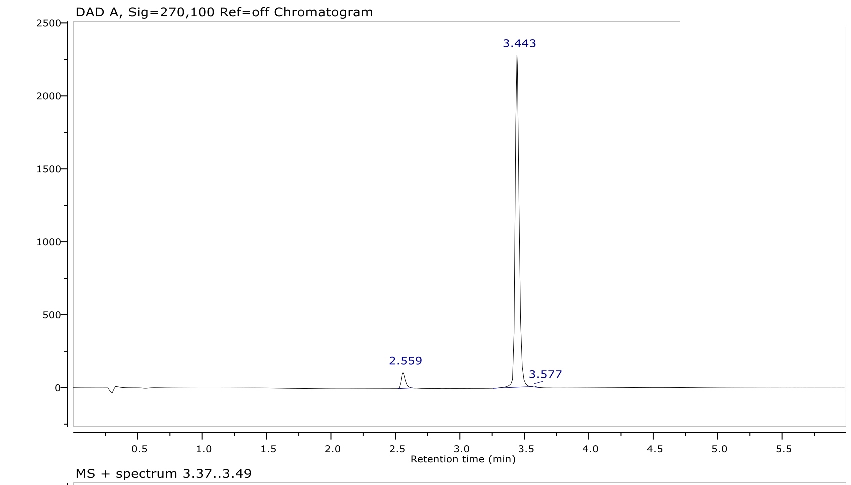

Integrals spectrum **Chromatogram DAD A, Sig=270,100 Ref=off**

rt (min)	height	area	area (%)
2.56	109.2	3.999	4.68
3.44	2276	81.19	95.10
3.58	6.724	0.1836	0.22

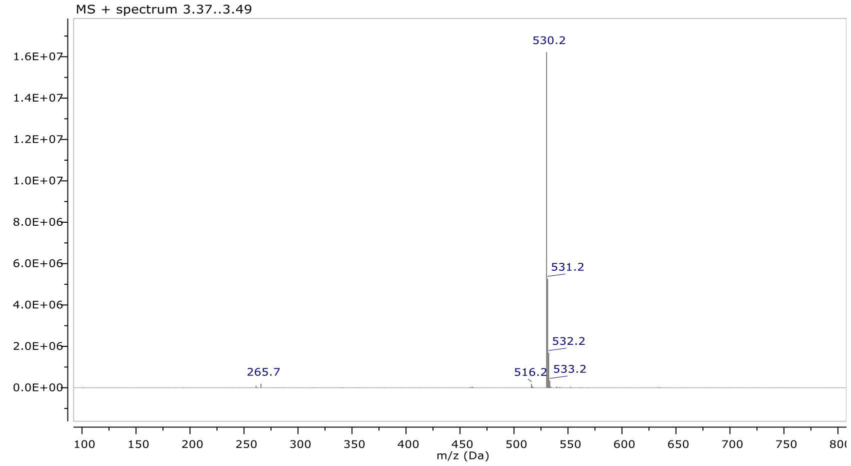

**Title**	**sjae 54 230-2**
**Data File Name**	0112_20
**Origin**	Bruker BioSpin GmbH
**Method**	
**Pulse Sequence**	zg30
**Relaxation Delay**	1 s
**Solvent**	CDCl3
**Acquisition Date**	2018-01-12T08:15:05
**Temperature**	~ 298 K
**Number of Scans**	32
**Frequency**	400.232471584084 MHz
**Nucleus**	1H

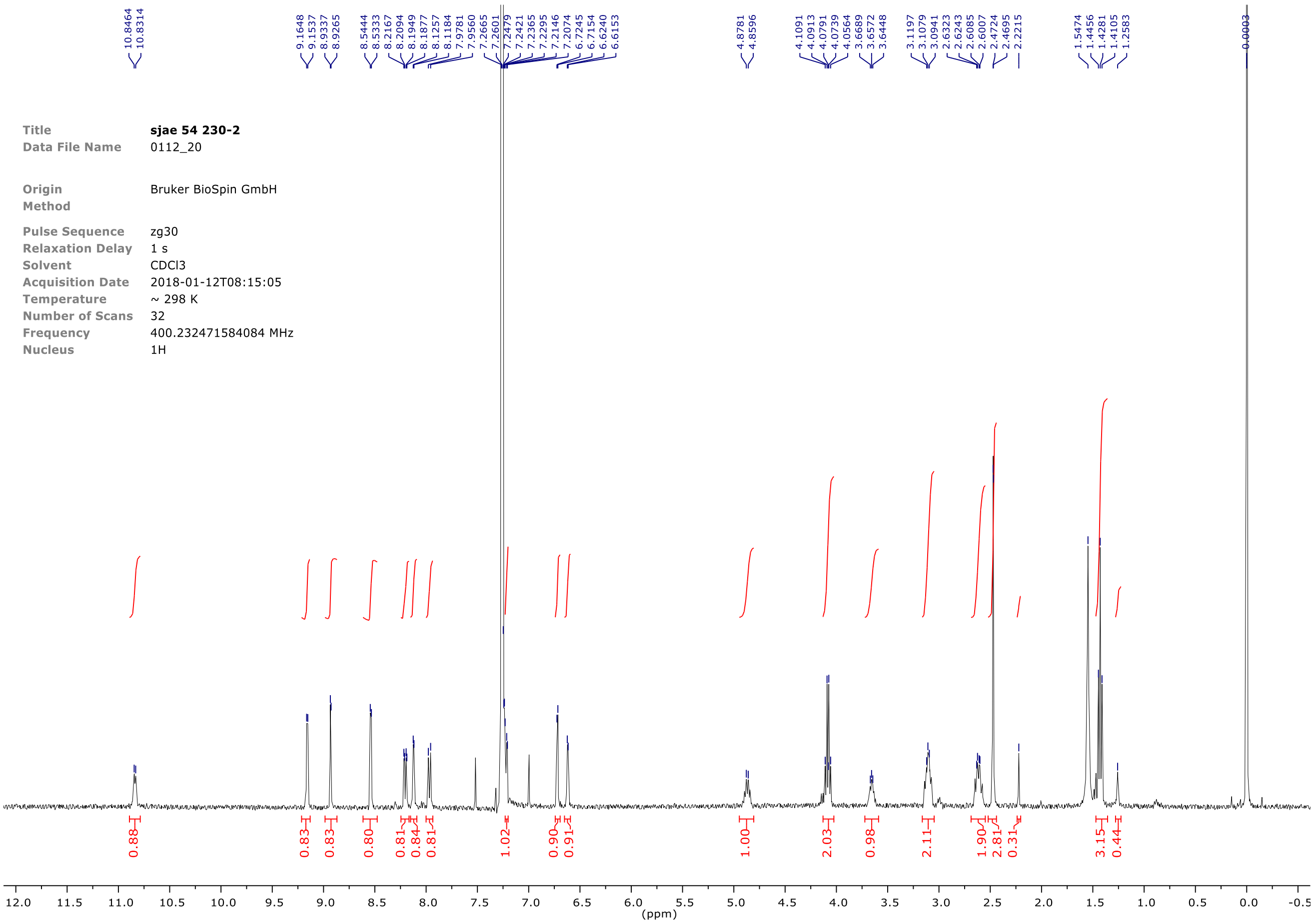

HRMS (m/z): C27H24N7O2FS, [M+H]+ Calc: 530.17690; found: 530.1761, Δppm -1.59

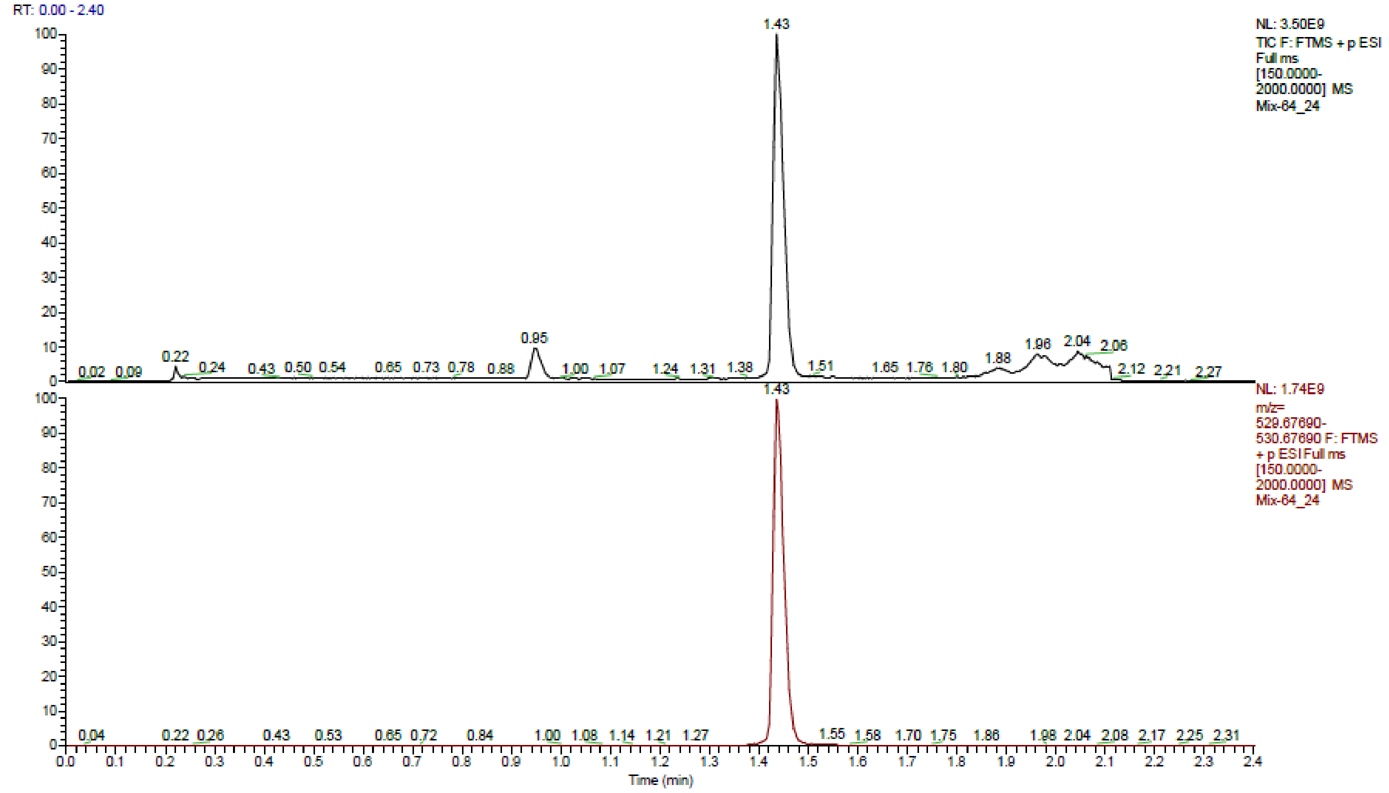

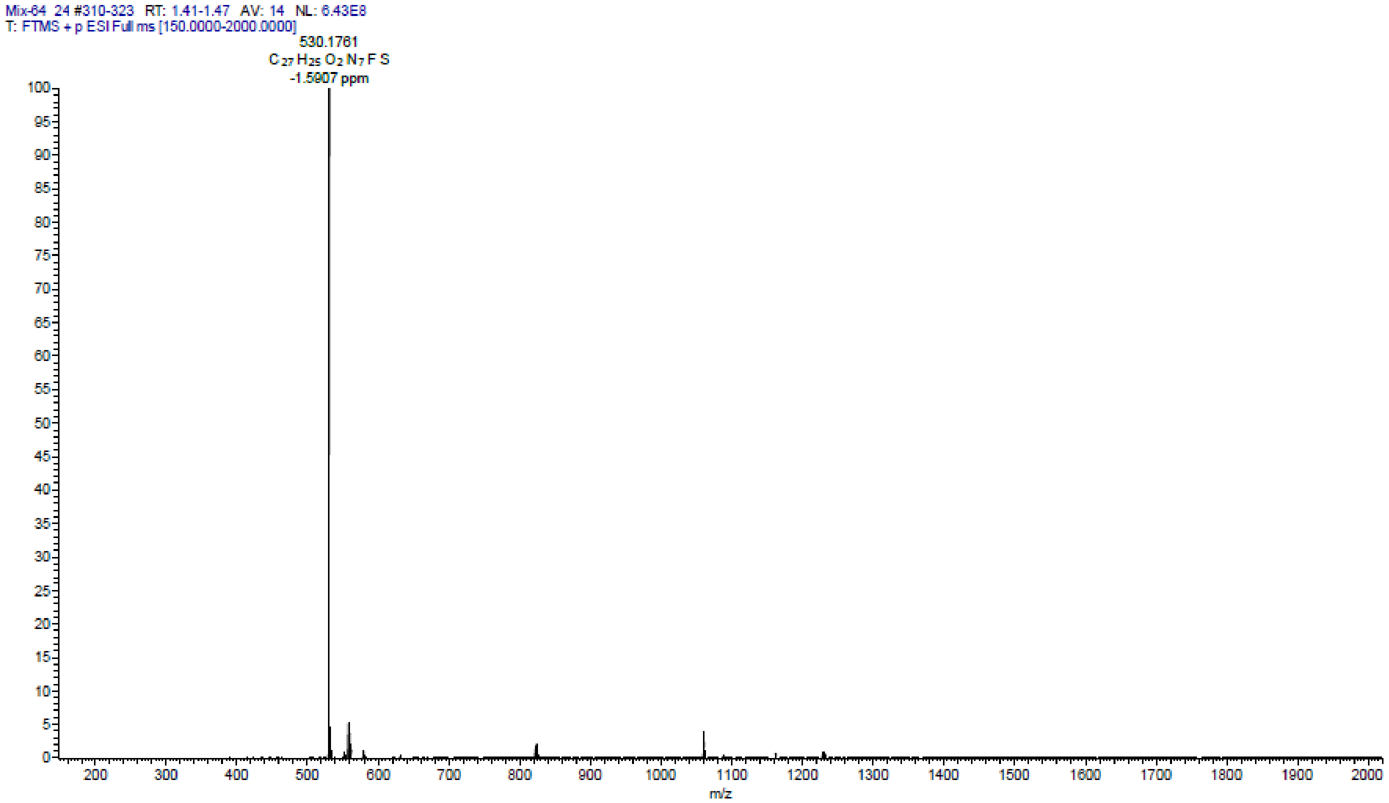

Compound 23

title	**sjae 54 232-2**
Method	AN_BASE.M
Date acquired	11-Jan-18, 18:03:54
FileName	Analysis\LCMS5_0111_099.D
Acq. method	AN_BASE valve: 2
Column	Waters XSelect CSH C18 (50x2.1mm, 3.5µ)
Flow	0.8 ml/min; Column temp: 25°C
Eluent A	95% acetonitrile + 5% 10 mM ammoniumbicarbonate in water
Eluent B	10mM ammoniumbicarbonate in water
Lin. Gradient	t=0 min 5% A, t=3.5 min 98% A, t=6 min 98% A
Posttime	2 min
Detection	DAD (210, 220 and 220-320 nm)
Detection	PDA (210-320 nm)
Detection	MSD (ESI pos/neg) mass range: 100-800

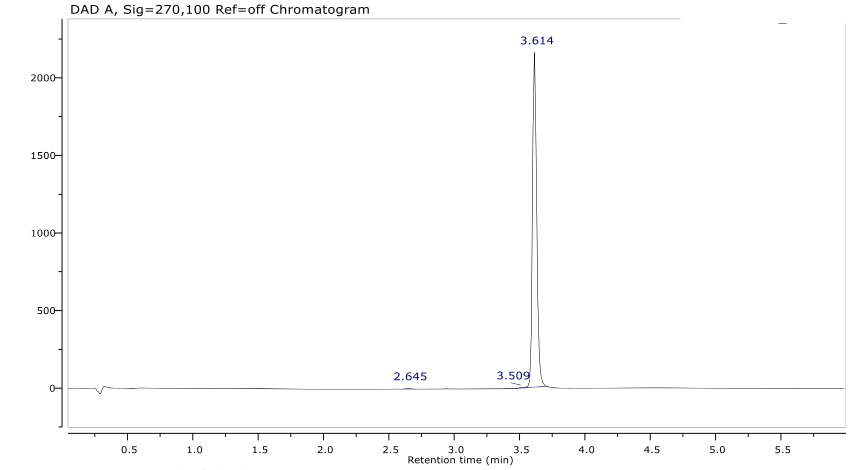

Integrals spectrum Chromatogram DAD A, Sig=270,100 Ref=off

rt (min)	height	area	area (%)
2.64	4.779	0.1530	0.19
3.51	4.086	0.1050	0.13
3.61	2157	80.90	99.68

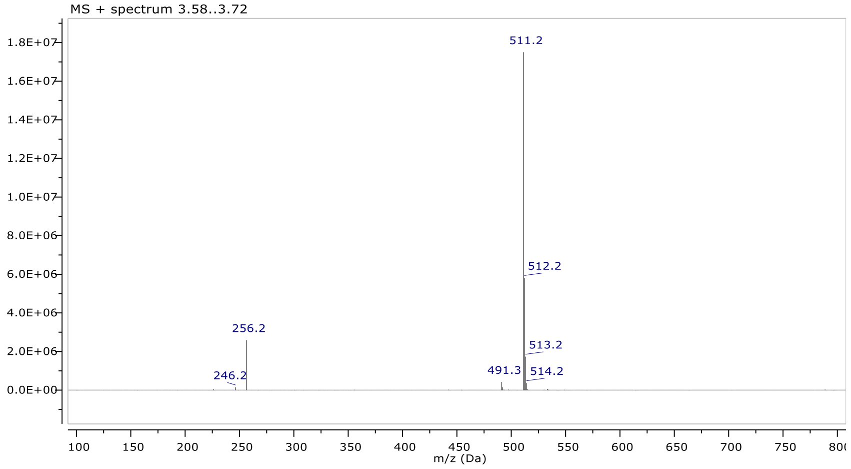

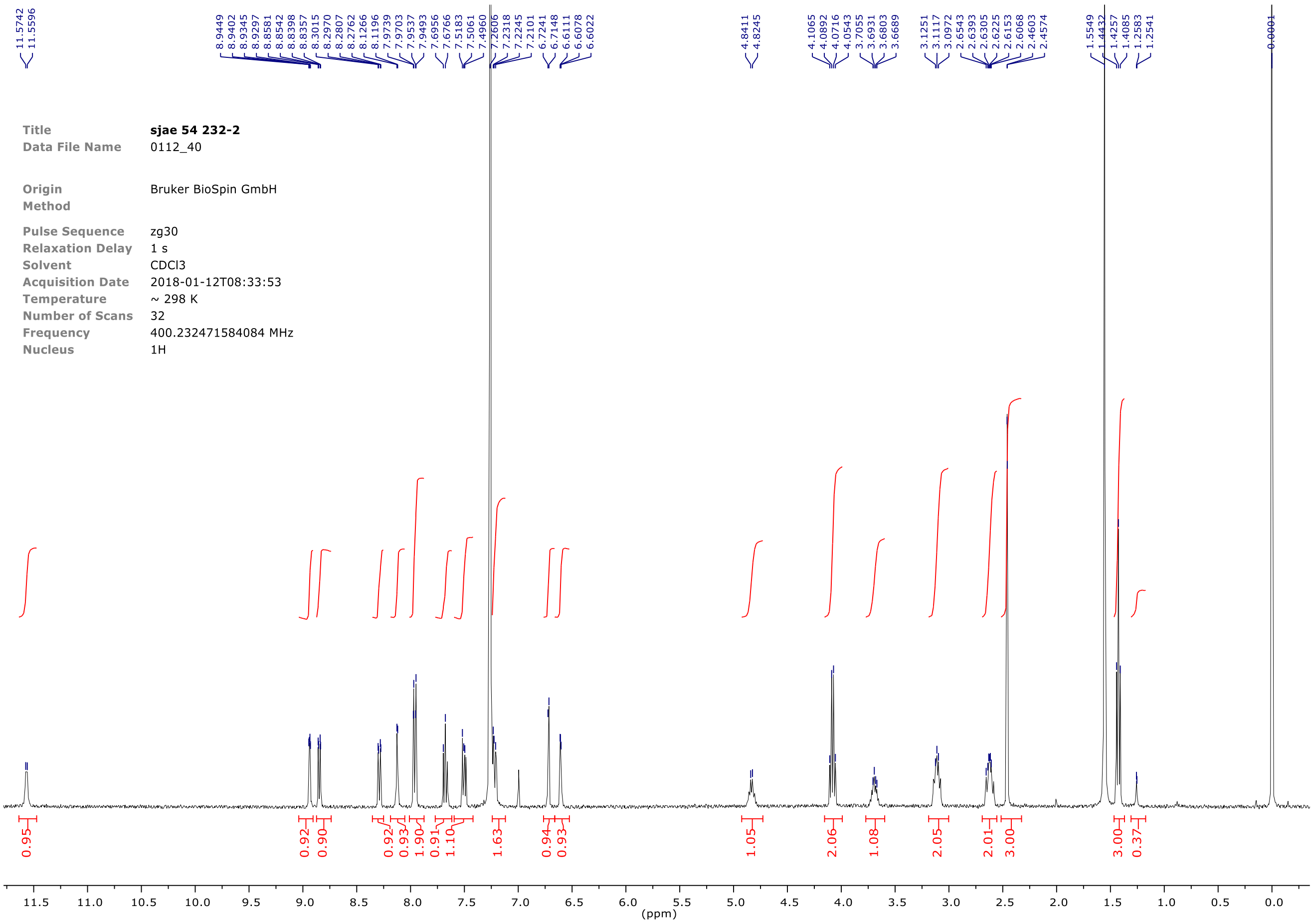

Title	**sjae 54 232-2**
Data File Name	0112_40
Origin	Bruker BioSpin GmbH
Method	
Pulse Sequence	zg30
Relaxation Delay	1 s
Solvent	CDCl3
Acquisition Date	2018-01-12T08:33:53
Temperature	~ 298 K
Number of Scans	32
Frequency	400.232471584084 MHz
Nucleus	1H

HRMS (m/z): C28H26N6O2S, [M+H]+ Calc: 511.19107; found: 511.1902, Δppm -1.63

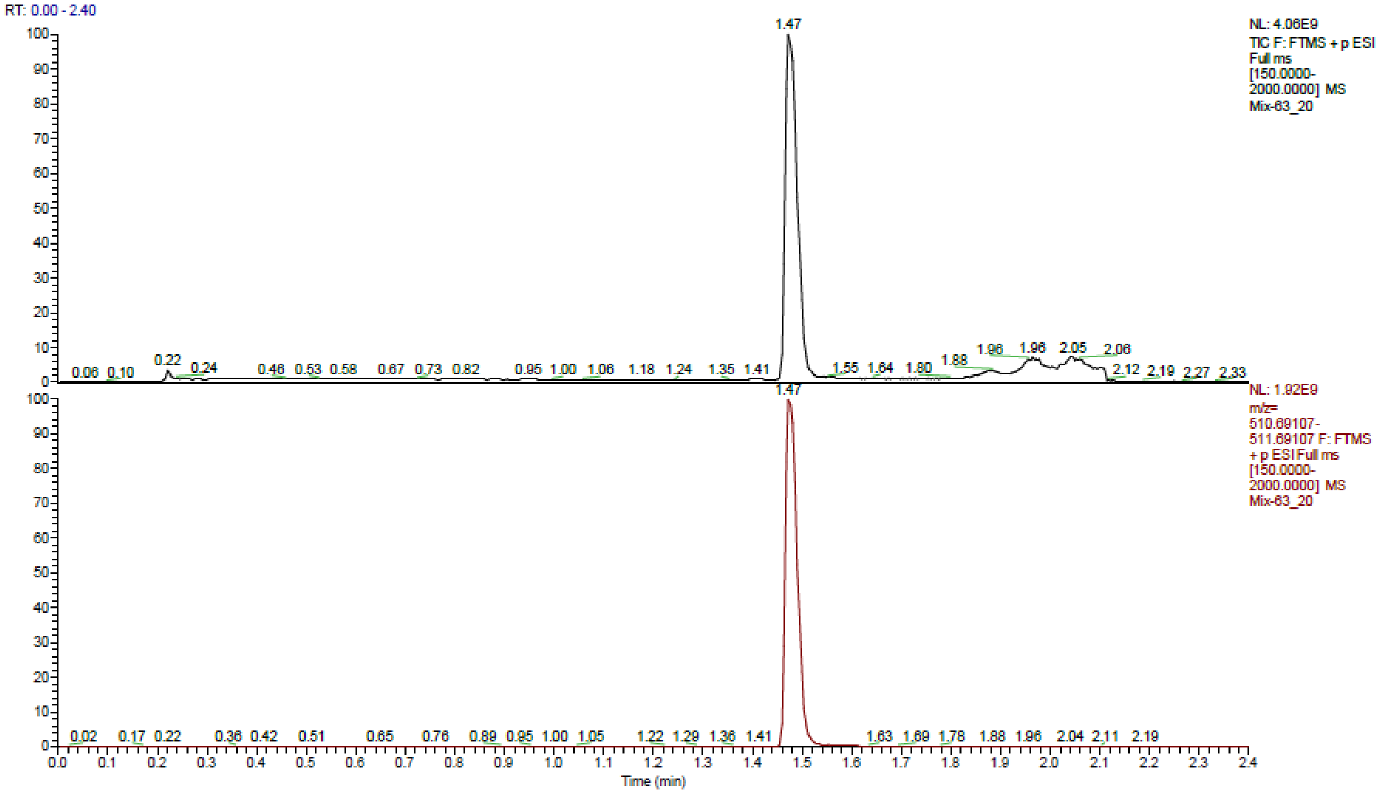

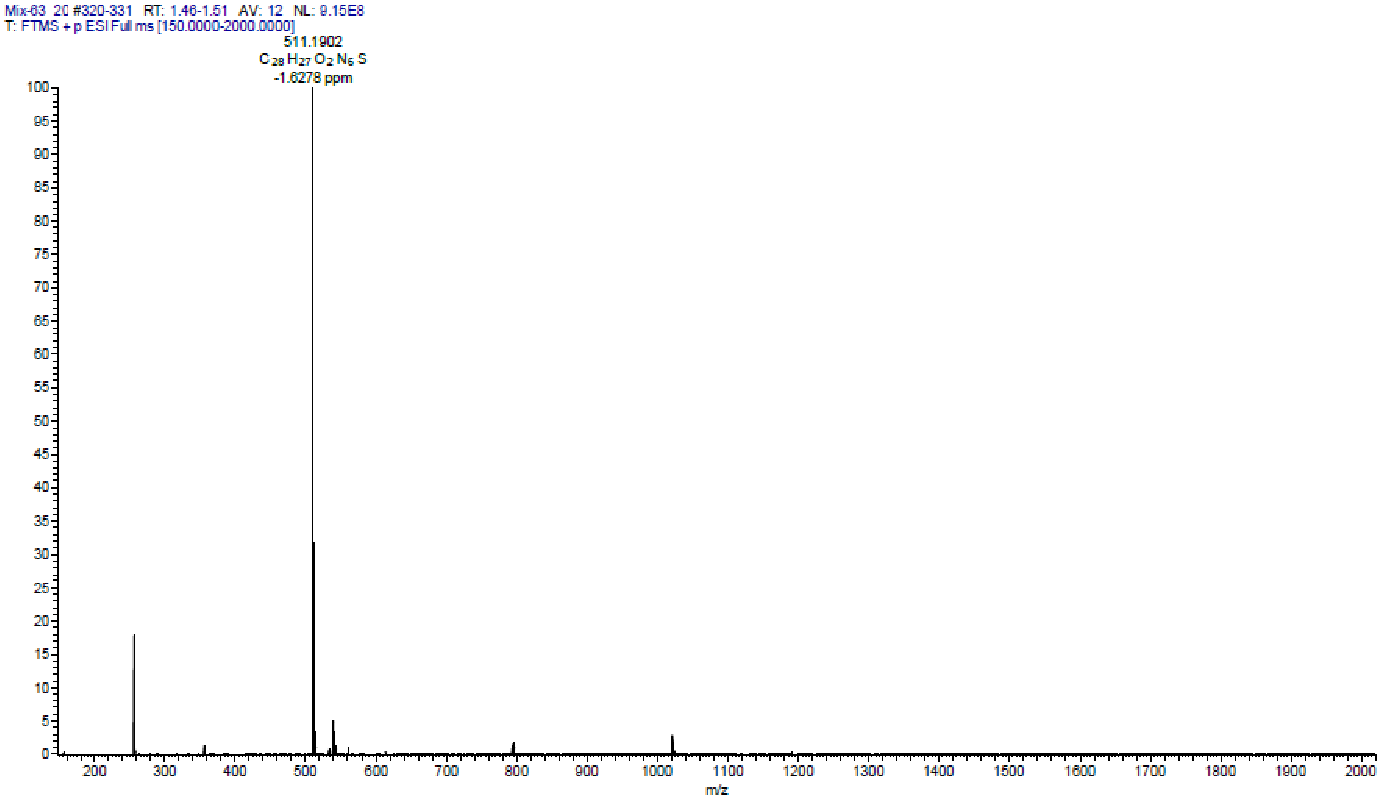

Compound 24 (OM-153)

title	**PINI23-83-4**
Method	AN_BASE.M
Date acquired	25-Jan-19, 16:55:31
FileName	Analysis\LCMS5_0125_080.D
Acq. method	AN_BASE valve: 2
Column	Waters XSelect CSH C18 (50x2.1mm, 3.5μ)
Flow	0.8 ml/min; Column temp: 25°C
Eluent A	100% acetonitrile
Eluent B	10mM ammoniumbicarbonate in water
Lin. Gradient	t=0 min 5% A, t=3.5 min 98% A, t=6 min 98% A
Posttime	2 min
Detection	DAD (210, 220 and 220-320 nm)
Detection	PDA (210-320 nm)
Detection	MSD (ESI pos/neg) mass range: 100-1000

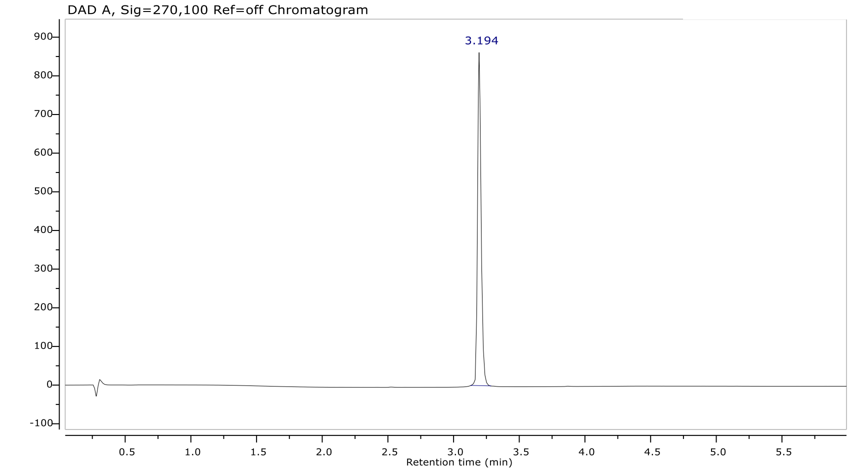

Integrals spectrum — Chromatogram DAD A, Sig=270,100 Ref=off

rt (min)	height	area	area (%)
3.19	861.8	27.05	100.00

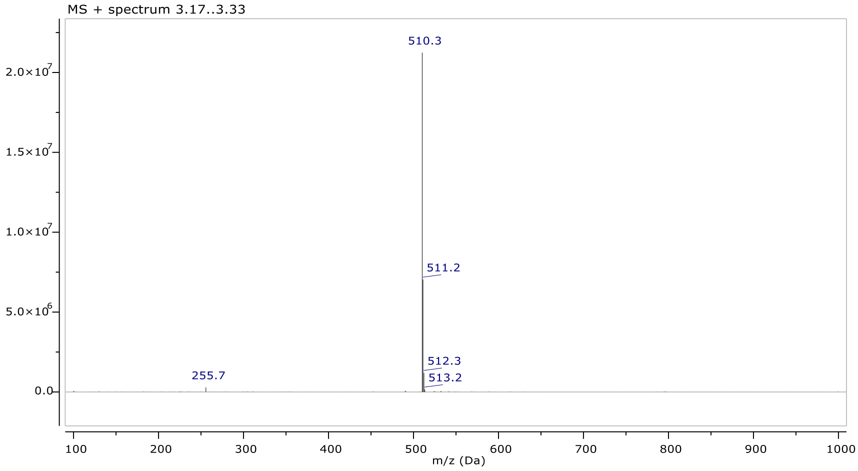

Title	**OM-153**
Data File Name	NMR_May03-2021_80
Origin	Bruker BioSpin GmbH
Method	1D
Pulse Sequence	zg30
Relaxation Delay	1 s
Solvent	DMSO
Acquisition Date	03 May 2021 (09:35)
Temperature	~ 25 °C
Number of Scans	16
Frequency	400 MHz
Nucleus	1H

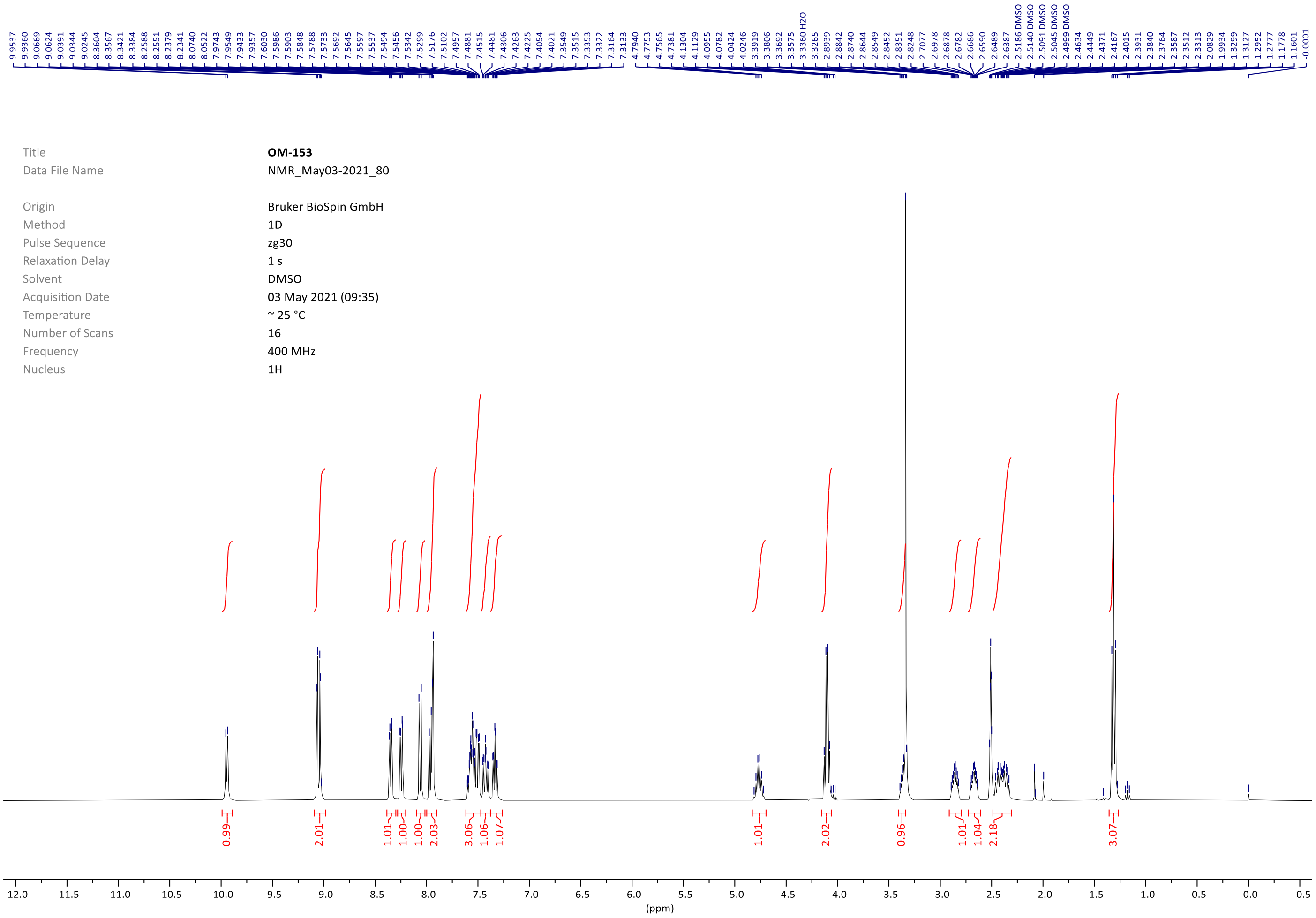

**title**	**PINI23-40-1**
**Date acquired**	25-Sep-2018, 19:01:04
**FileName**	Analysis\SFC1_180925_ch_PINI_17.raw
**UPLC**	Waters UPC2
**Acq. Method**	3_Cel1_05-50_f25_P170_Amm_10min
**Column**	Phenomenex Cellulose-1 (100x4.6mm 5μm)
**Flow**	2.5 ml/min; Column temp: 35°C; BPR: 170 bar
**Eluent A**	CO2
**Eluent B**	Methanol + 20mM ammonia
**Gradient**	t=0 min 5% B, t=7 min 50% B, t=10 min 50% B
**Posttime**	0.5 min
**Detection PDA**	210-320nm
**MS**	QDa
**Detection MS**	ESI (scan)
**Mass range**	100-650 (pos), 1Hz
**Time**	10 min
**Cone**	15 V

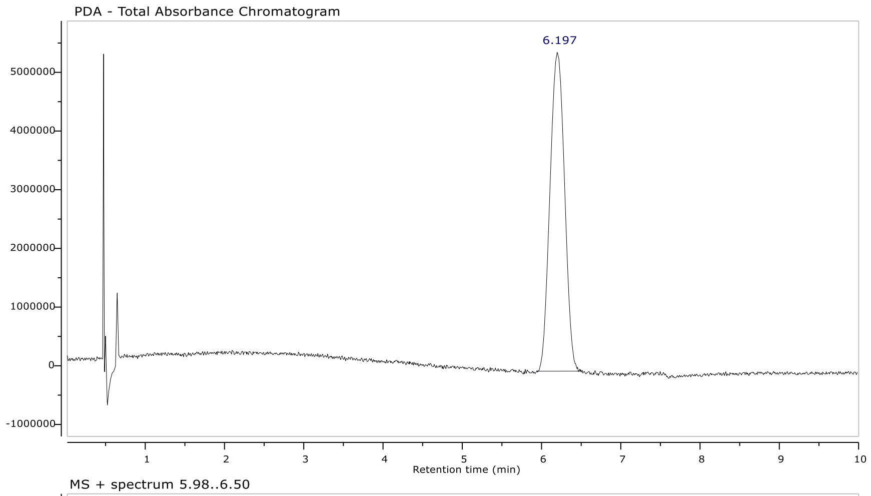

Integrals spectrum **PDA - Total Absorbance Chromatogram**

rt (min)	height	area	area (%)
6.20	5434219	1159261	100.00

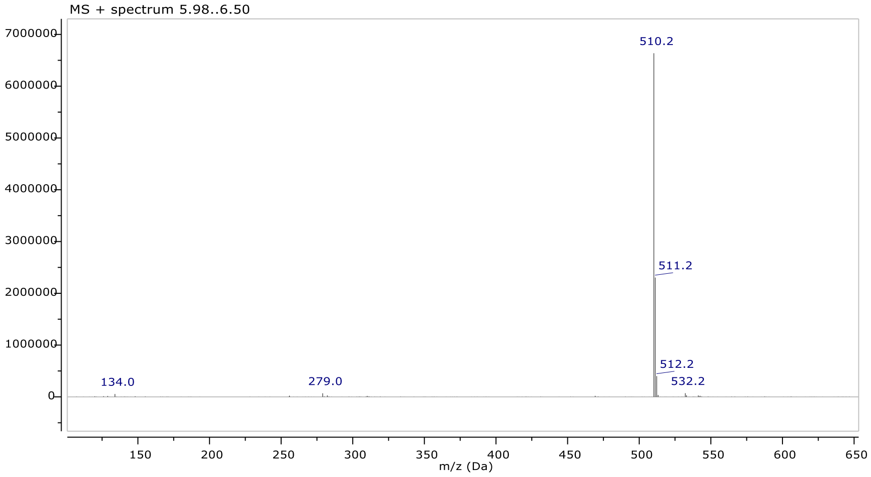

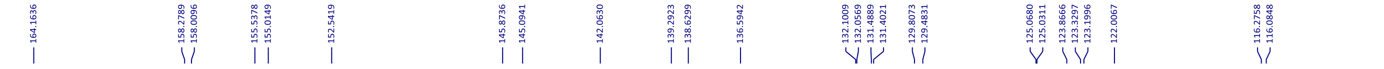

Title	**OM-153**
Data File Name	NMR_Apr30-2021_630
Origin	Bruker BioSpin GmbH
Method	JMOD
Pulse Sequence	jmod
Relaxation Delay	2 s
Solvent	DMSO
Acquisition Date	01 May 2021 (19:39)
Temperature	~ 25 °C
Number of Scans	4096
Frequency	101 MHz
Nucleus	13C

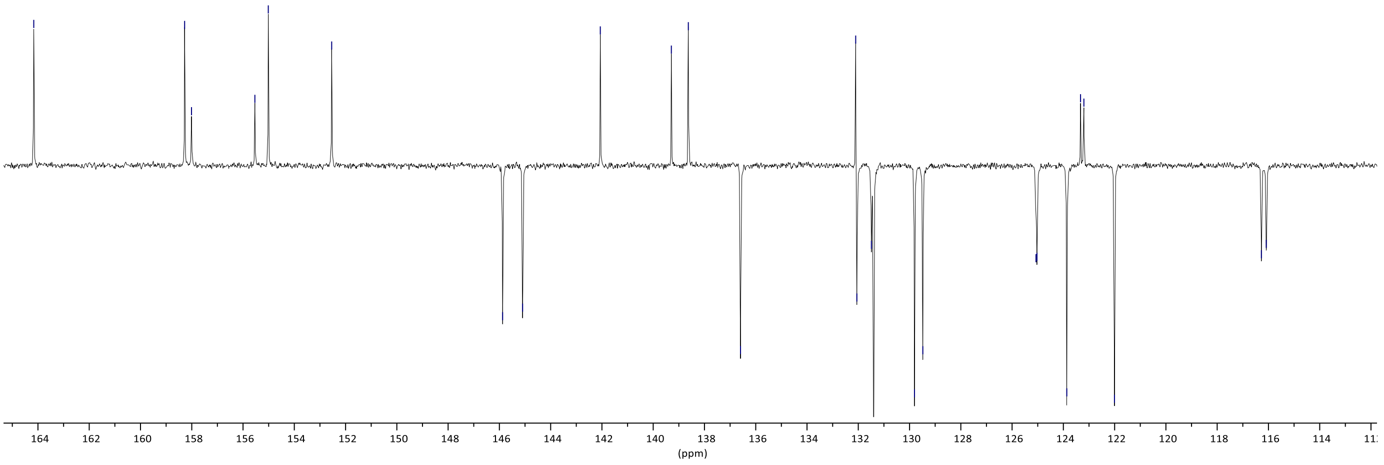

HRMS (m/z): $C_{28}H_{24}N_{7}O_{2}F$, $[M+H]^+$ Calc: 510.20483; found: 510.2040, Δppm -1.66

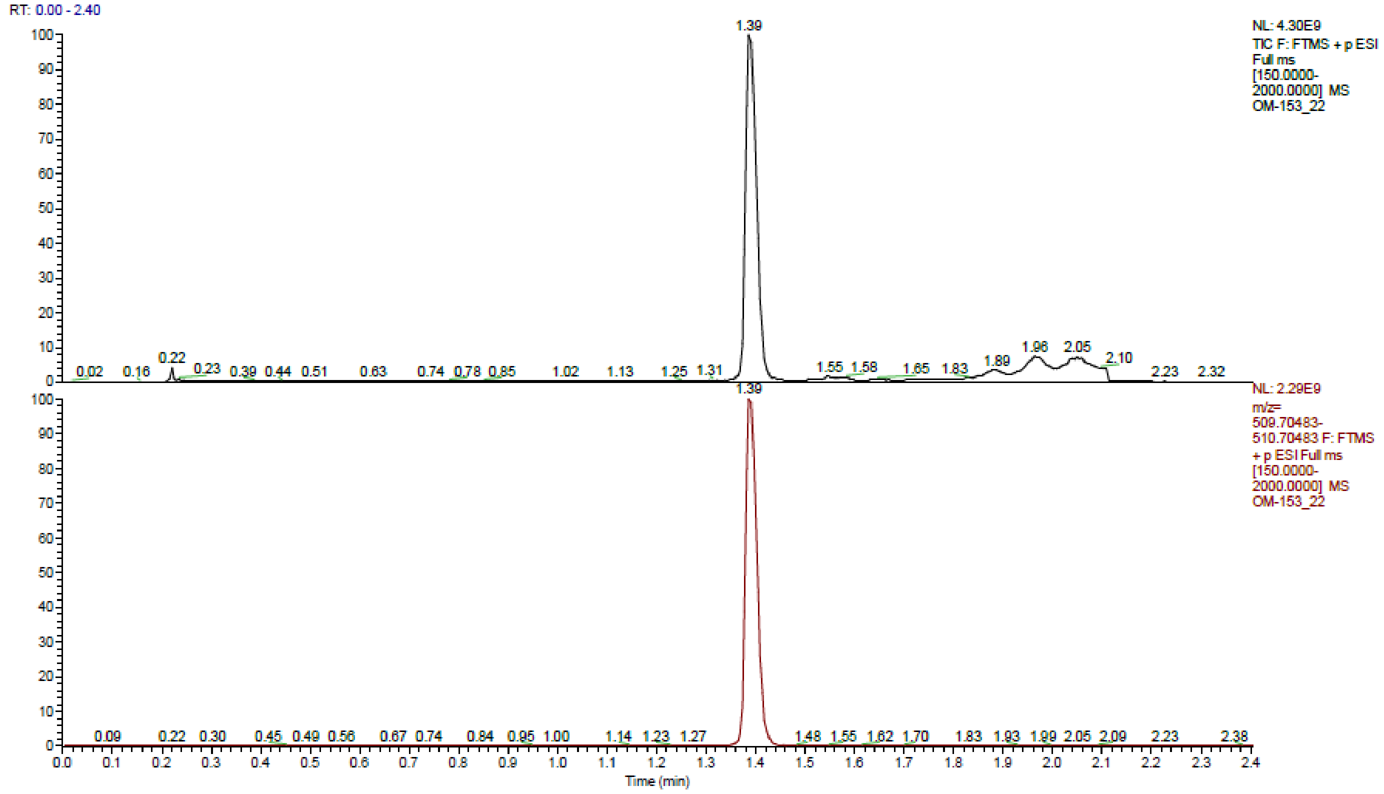

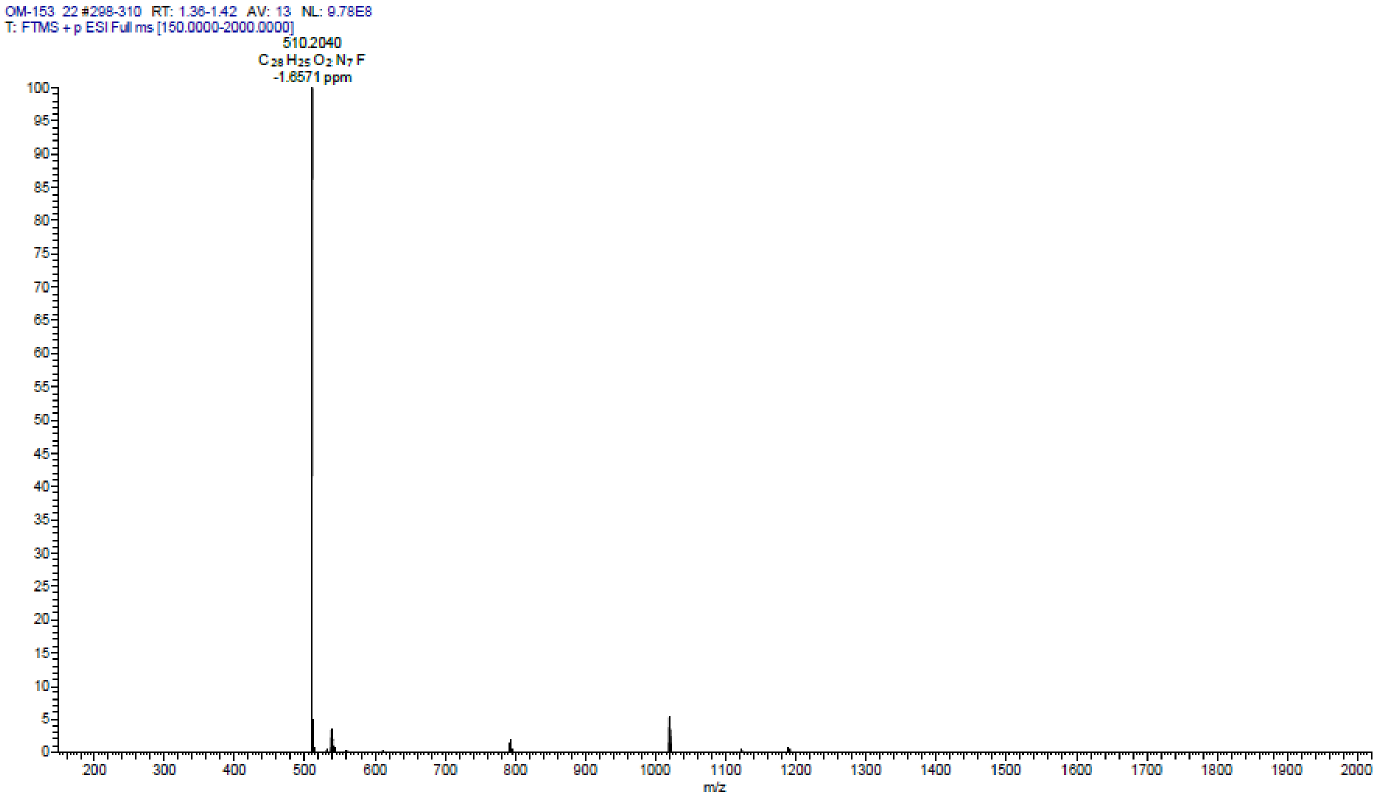

Compound 25

**title**	**PINI23-70-1**
**Method**	AN_ACID.M
**Date acquired**	08-Nov-18, 18:07:46
**FileName**	Analysis\LCMS6_1108_105.D
**Column**	XSelect CSH C18 (50x2.1mm, 3.5μ) valve:3
**Flow**	0.8 ml/min; Column temp: 35°C
**Eluent A**	0.1% formic acid in acetonitrile
**Eluent B**	0.1% formic acid in water
**Gradient**	t=0 min 5% A, t=3.5 min 98% A, t=6 min 98%A
**Posttime**	2 min
**Detection**	DAD(210, 220 and 220-320nm)
**Detection**	PDA(210-320nm)
**Detection**	MSD (ESI pos/neg) mass range: 100 - 1000
**Detection**	ELSD gas temp: 40°C, flow 1.5 ml/min, gain 1

DAD A, Sig=270,100 Ref=off Chromatogram

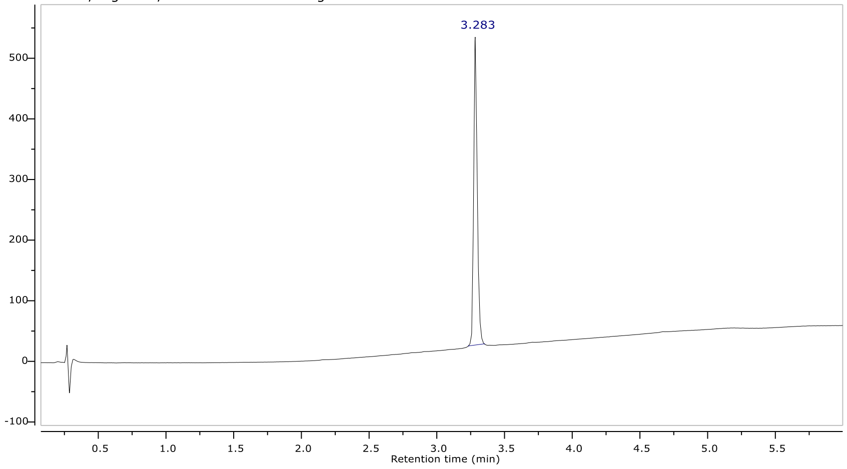

Integrals spectrum **Chromatogram DAD A, Sig=270,100 Ref=off**

rt (min)	height	area	area (%)
3.28	508.1	15.54	100.00

MS + spectrum 3.31..3.40

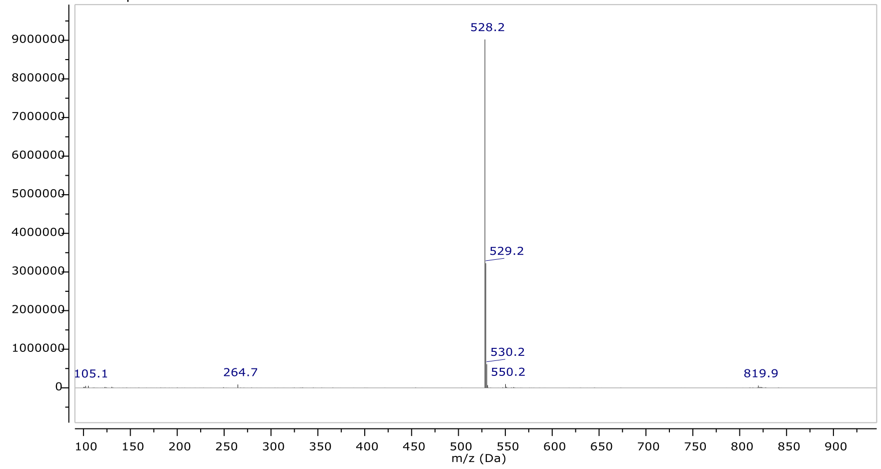

**Title**	**PINI23-70-1**
**Data File Name**	1109_250
**Origin**	Bruker BioSpin GmbH
**Method**	
**Pulse Sequence**	zg30
**Relaxation Delay**	1 s
**Solvent**	CDCl3
**Acquisition Date**	2018-11-09T13:15:47
**Temperature**	~ 298.06 K
**Number of Scans**	64
**Frequency**	400.232471584084 MHz
**Nucleus**	1H

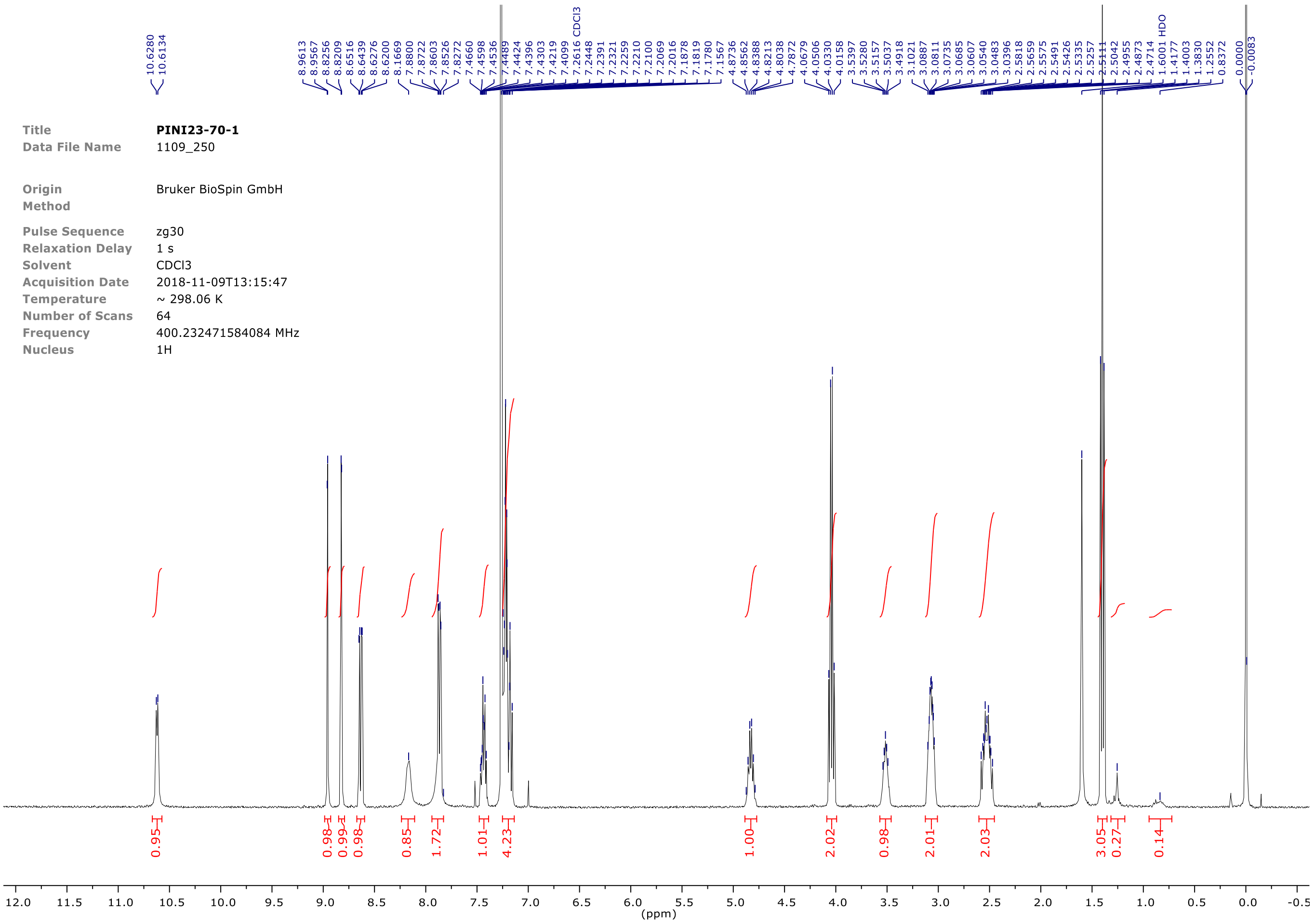

HRMS (m/z): C28H23N7O2F2, [M+H]+ Calc: 528.19541; found: 528.1946, Δppm -1.59

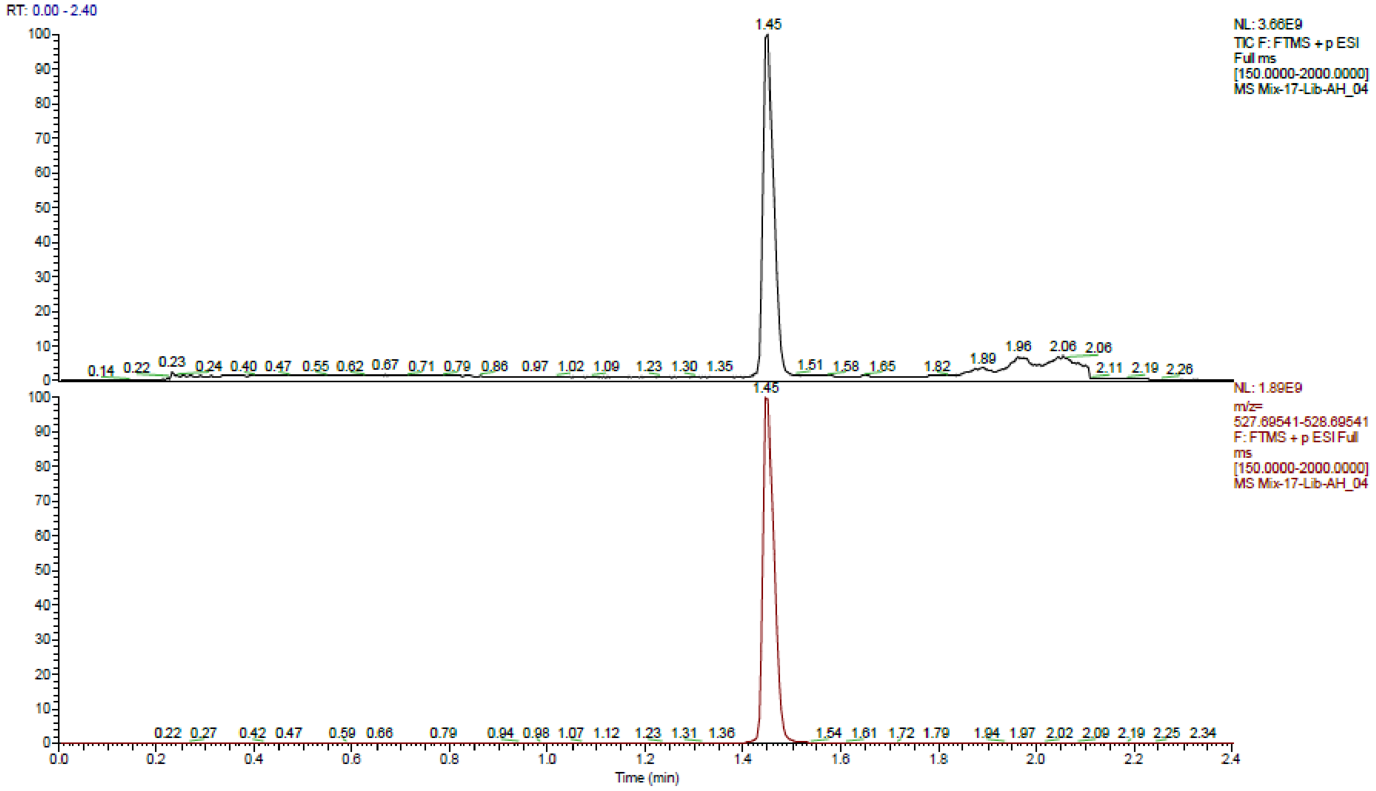

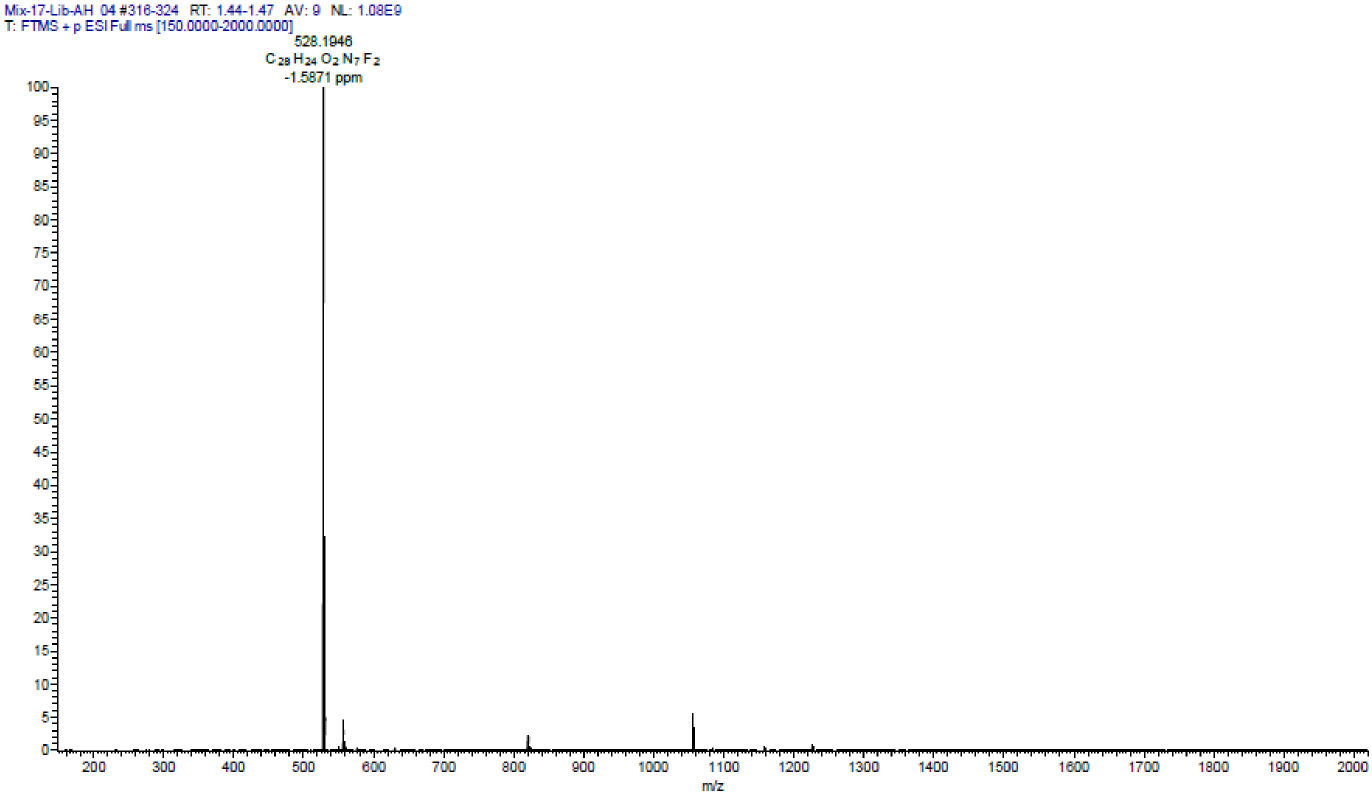

Compound 26

title	**YASM09-025-1**
Method	AN_ACID.M
Date acquired	26-Apr-21, 10:21:30
FileName	Analysis\LCMS22_20210426_RULE-25-1_18387.D
Acq.method	AN_ACID
Column	XSelect CSH C18 (50x2.1mm 3.5μ)
Flow	0.8 ml/min, Column temp.: 40°C
Eluent A	0.1% Formic acid in Water
Eluent B	0.1% Formic acid in Acetonitrile
Lin. gradient	t=0 min 5%B, t=4.5 min 98%B, t=6 min 98% B
Postrun	2 min
Detection	DAD (210, 220 and 220-320 nm)
Detection	PDA (210-320 nm)
Detection	MSD (ESI pos/neg) mass range 100-1000
Detection	ELSD (Neb temp. 50°C, gassflow 1.3 ml/min)

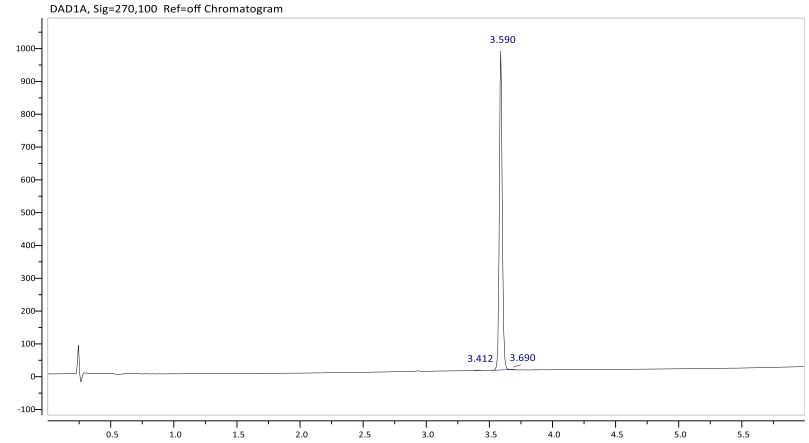

Integrals spectrum Chromatogram DAD1A, Sig=270,100 Ref=off

rt (min)	height	area	area (%)
3.41	1.012	30.59	0.10
3.59	971.8	30925	99.81
3.69	1.174	29.29	0.09

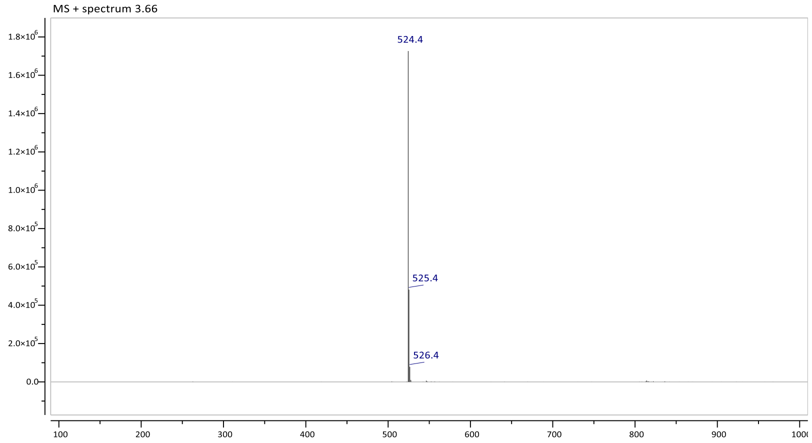

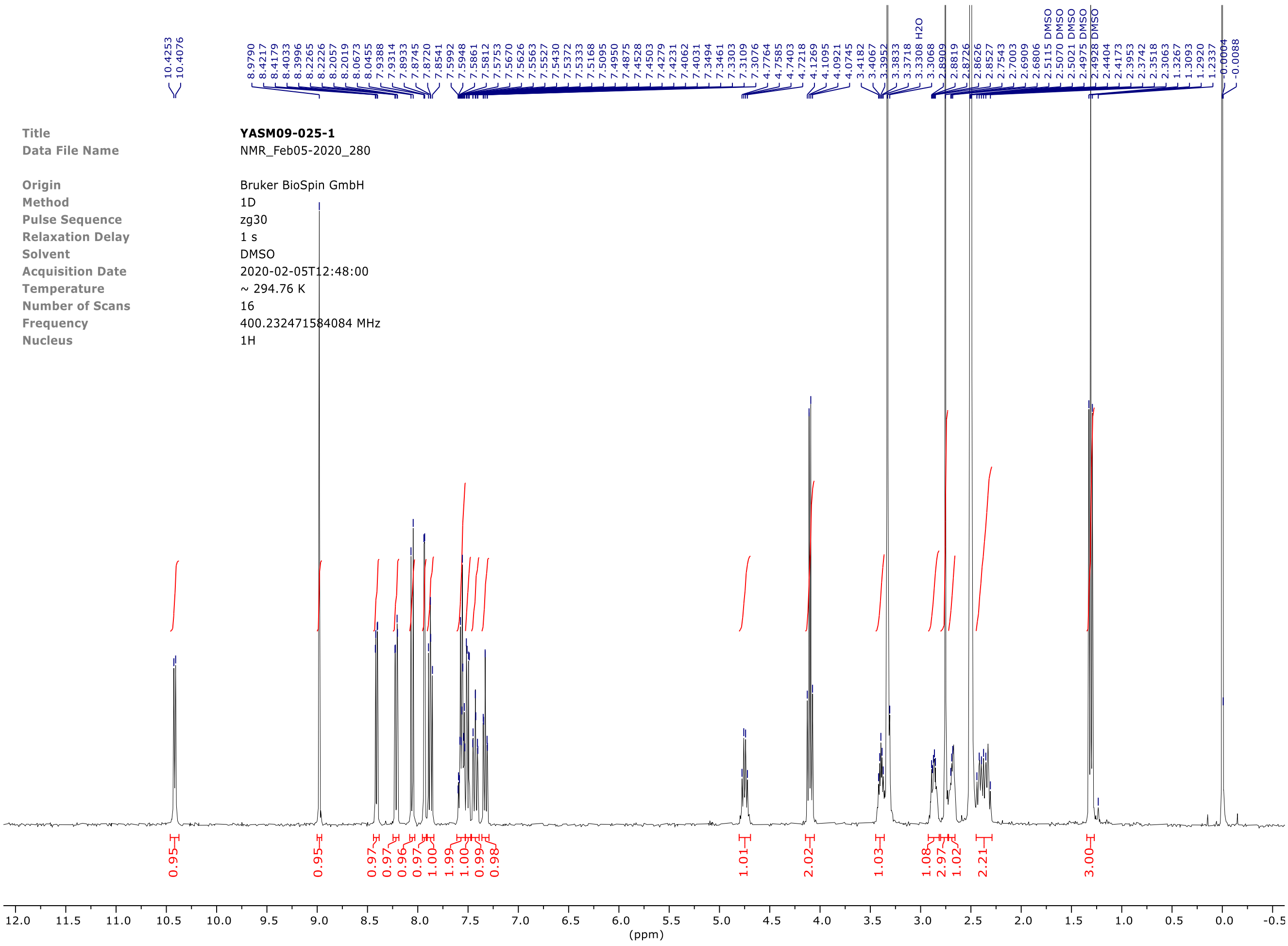

HRMS (m/z): C29H26N7O2F, [M+H]+ Calc: 524.22048; found: 524.2197, Δppm -1.56

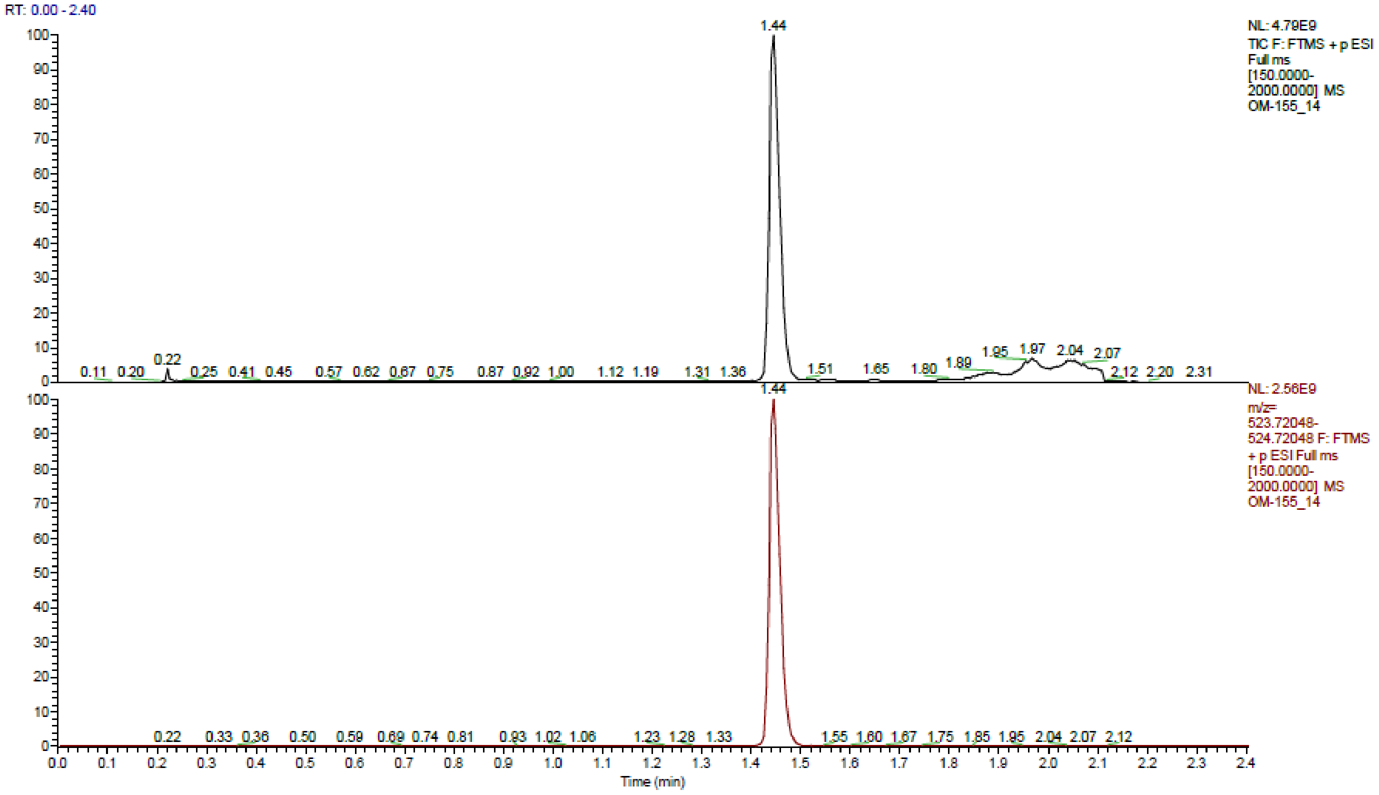

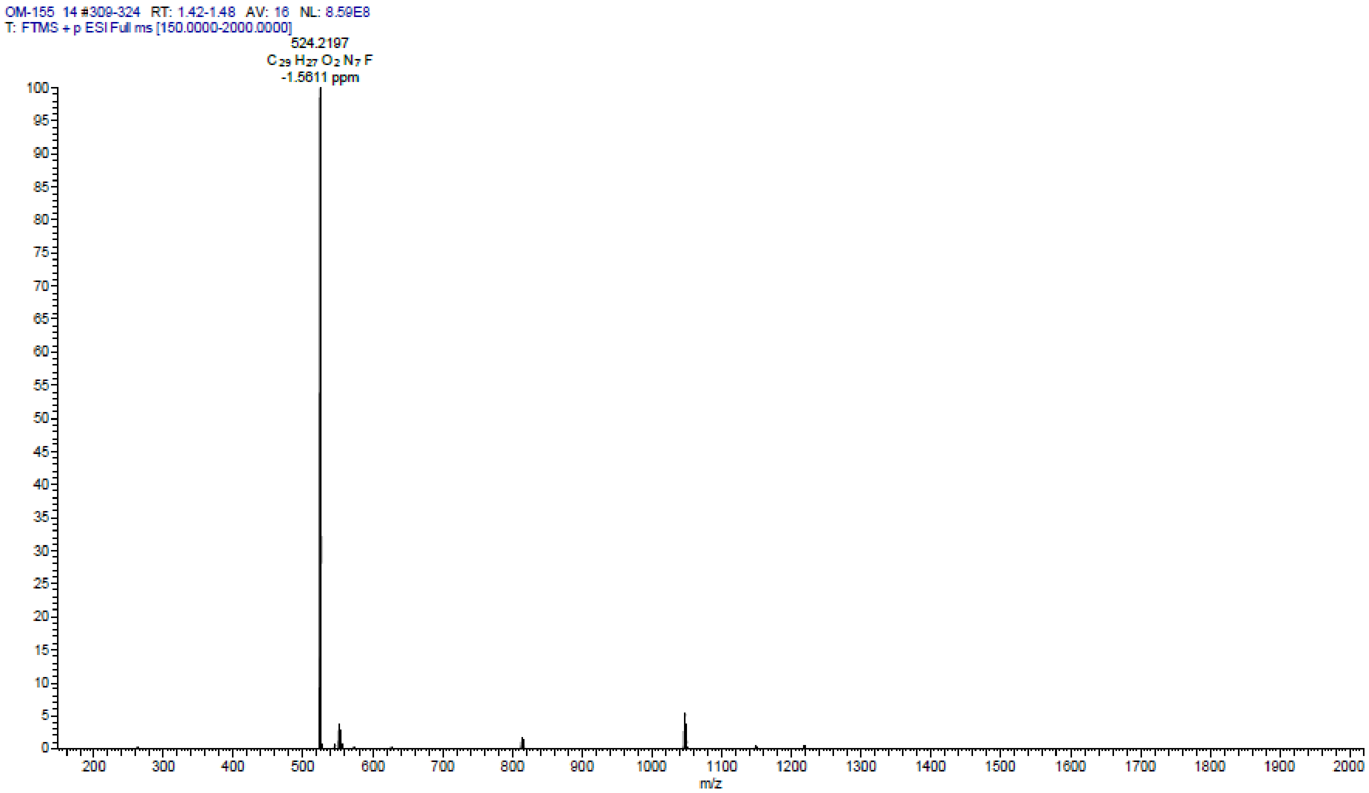

Compound 27

title	**YASM09-026-1**
Method	AN_ACID.M
Date acquired	26-Apr-21, 10:39:42
FileName	Analysis\LCMS22_20210426_RULE-26-1_18388.D
Acq.method	AN_ACID
Column	XSelect CSH C18 (50x2.1mm 3.5µ)
Flow	0.8 ml/min, Column temp.: 40°C
Eluent A	0.1% Formic acid in Water
Eluent B	0.1% Formic acid in Acetonitrile
Lin. gradient	t=0 min 5%B, t=4.5 min 98%B, t=6 min 98% B
Postrun	2 min
Detection	DAD (210, 220 and 220-320 nm)
Detection	PDA (210-320 nm)
Detection	MSD (ESI pos/neg) mass range 100-1000
Detection	ELSD (Neb temp. 50°C, gassflow 1.3 ml/min)

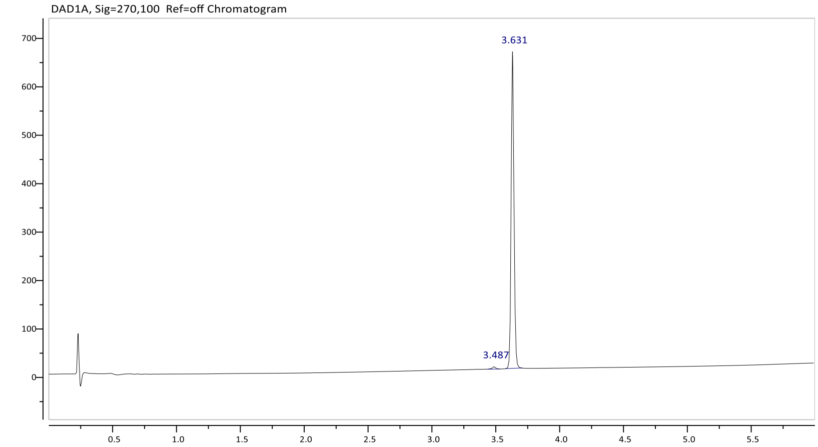

Integrals spectrum Chromatogram DAD1A, Sig=270,100 Ref=off

rt (min)	height	area	area (%)
3.49	4.877	185.4	0.91
3.63	653.7	20143	99.09

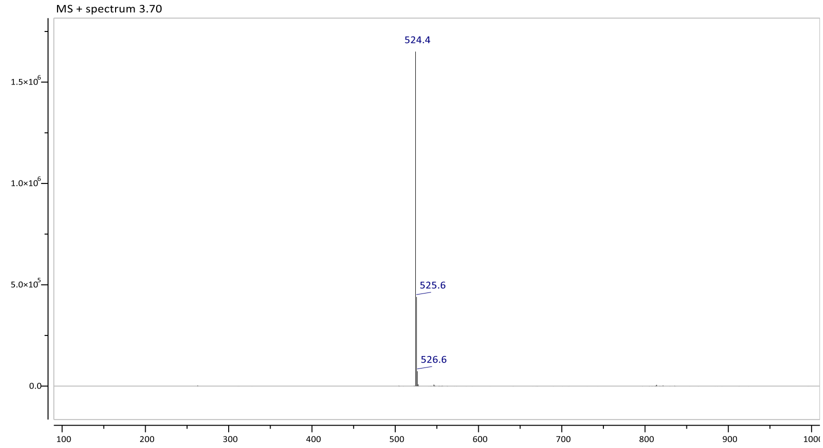

**Title**	**YASM09-026-1**
**Data File Name**	NMR_Feb10-2020_270
**Origin**	Bruker BioSpin GmbH
**Method**	1D
**Pulse Sequence**	zg30
**Relaxation Delay**	1 s
**Solvent**	DMSO
**Acquisition Date**	2020-02-10T12:54:00
**Temperature**	~ 294.76 K
**Number of Scans**	16
**Frequency**	400.232471584084 MHz
**Nucleus**	1H

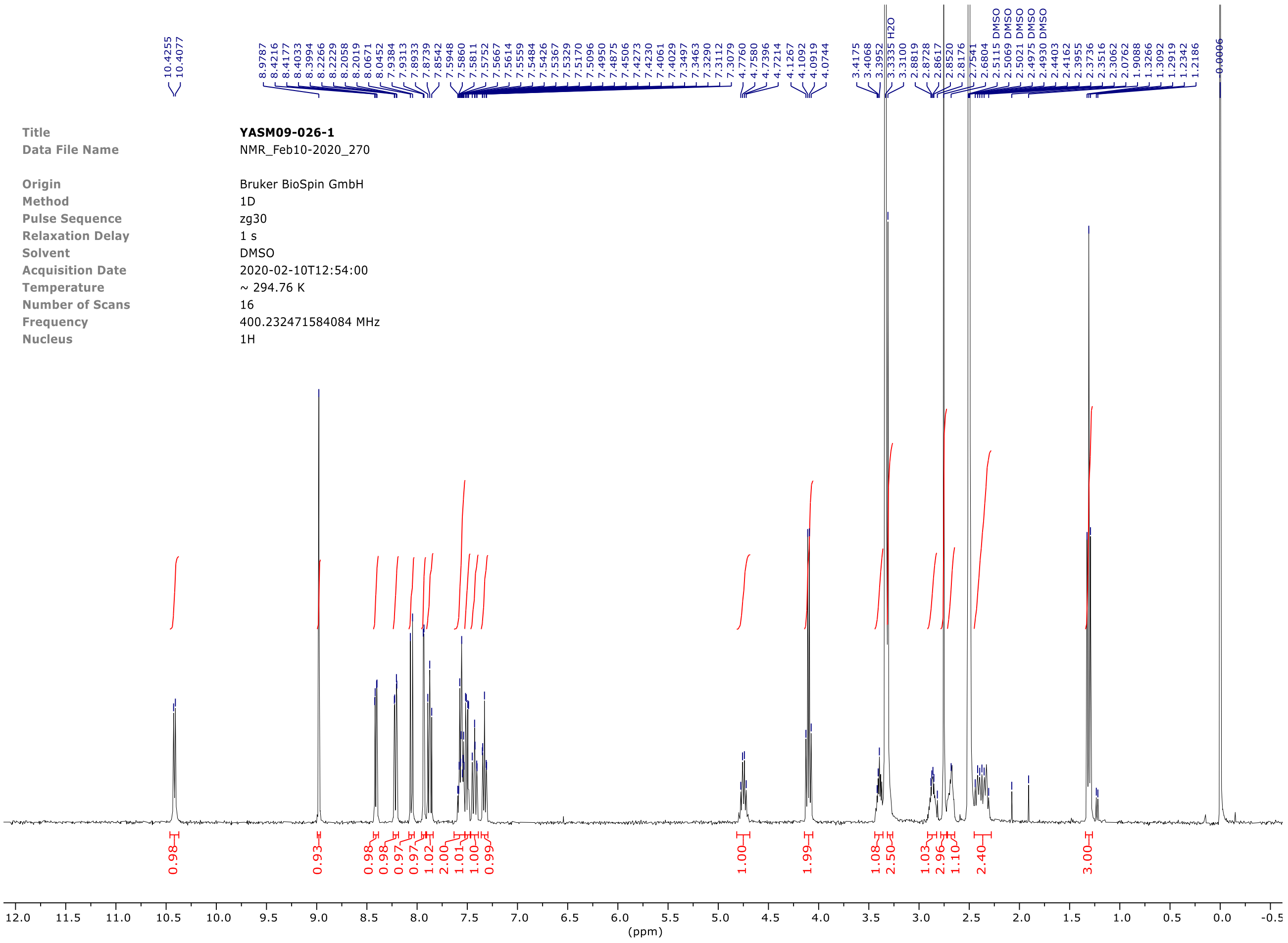

HRMS (m/z): C29H26N7O2F, [M+H]+ Calc: 524.22048; found: 524.2198, Δppm -1.36

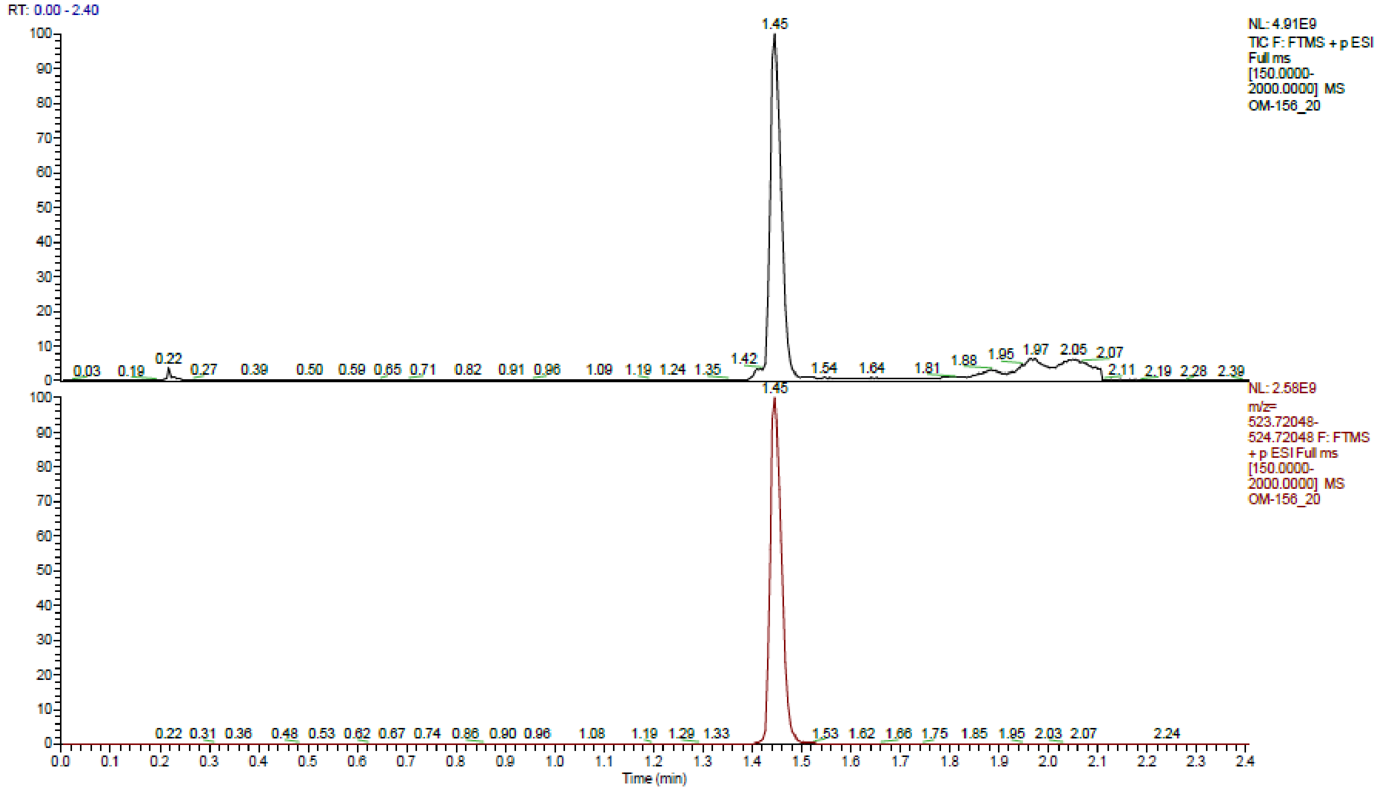

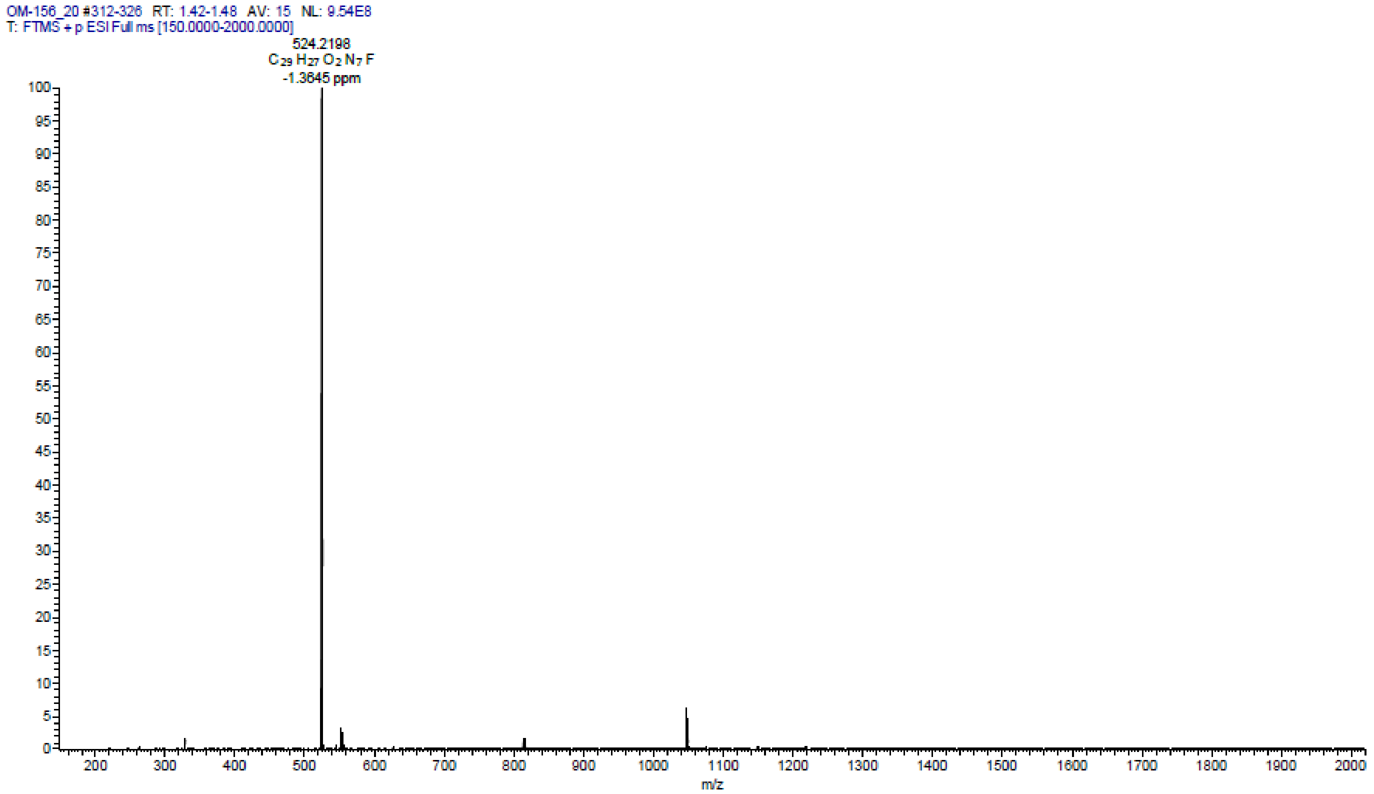

Compound 28

title	**RYASM09-002-3**
Method	AN_ACID.M
Date acquired	26-Apr-21, 10:07:50
FileName	Analysis\LCMS22_20210426_RULE-2-3_18386.D
Acq.method	AN_ACID
Column	XSelect CSH C18 (50x2.1mm 3.5µ)
Flow	0.8 ml/min, Column temp.: 40°C
Eluent A	0.1% Formic acid in Water
Eluent B	0.1% Formic acid in Acetonitrile
Lin. gradient	t=0 min 5%B, t=4.5 min 98%B, t=6 min 98% B
Postrun	2 min
Detection	DAD (210, 220 and 220-320 nm)
Detection	PDA (210-320 nm)
Detection	MSD (ESI pos/neg) mass range 100-1000
Detection	ELSD (Neb temp. 50°C, gassflow 1.3 ml/min)

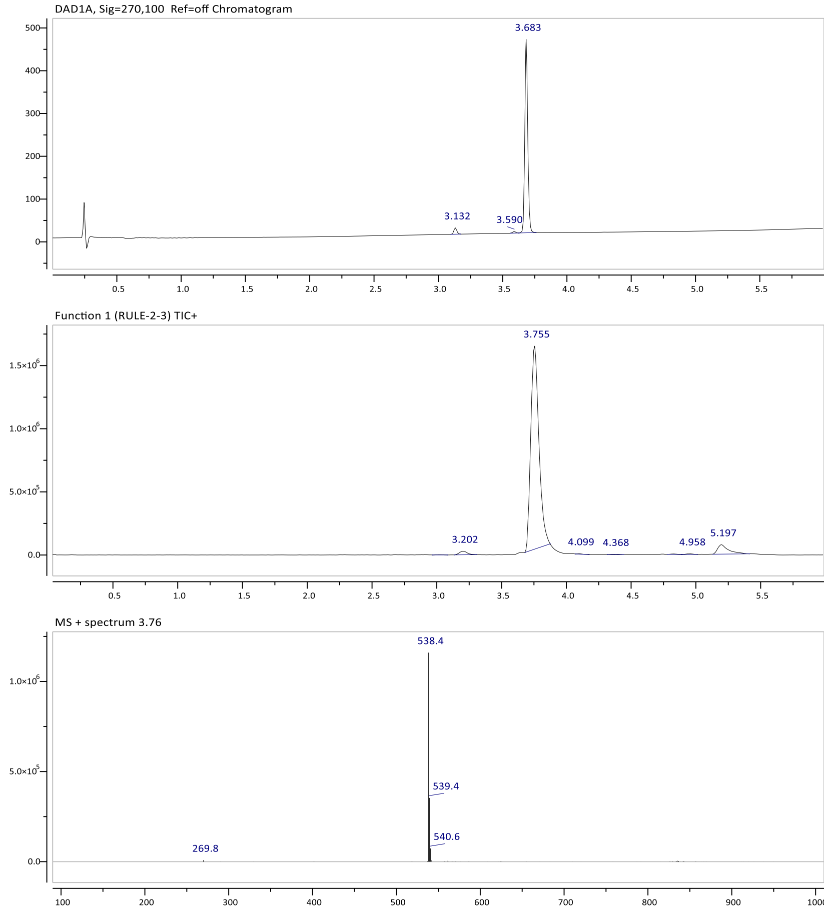

Integrals spectrum Chromatogram DAD1A, Sig=270,100 Ref=off

rt (min)	height	area	area (%)
3.13	14.86	479.2	3.22
3.59	3.818	129.8	0.87
3.68	452.3	14258	95.90

**Title**	**YASM09-002-3**
**Data File Name**	NMR_Feb05-2020_200
**Origin**	Bruker BioSpin GmbH
**Method**	1D
**Pulse Sequence**	zg30
**Relaxation Delay**	1 s
**Solvent**	DMSO
**Acquisition Date**	2020-02-05T11:18:00
**Temperature**	~ 294.86 K
**Number of Scans**	16
**Frequency**	400.232471584084 MHz
**Nucleus**	1H

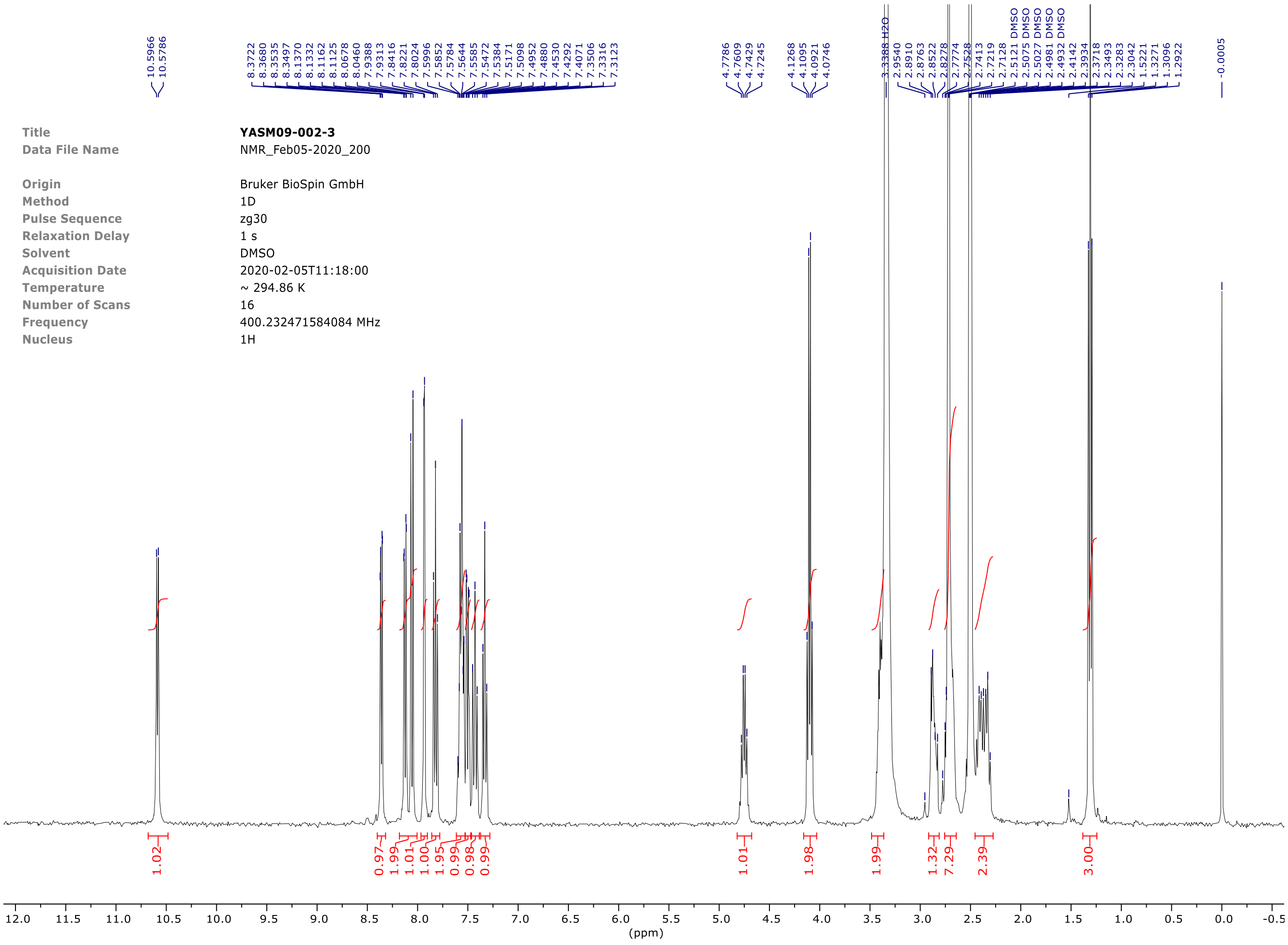

HRMS (m/z): C30H28N7O2F, [M+H]+ Calc: 538.23613; found: 538.2349, Δppm -2.27

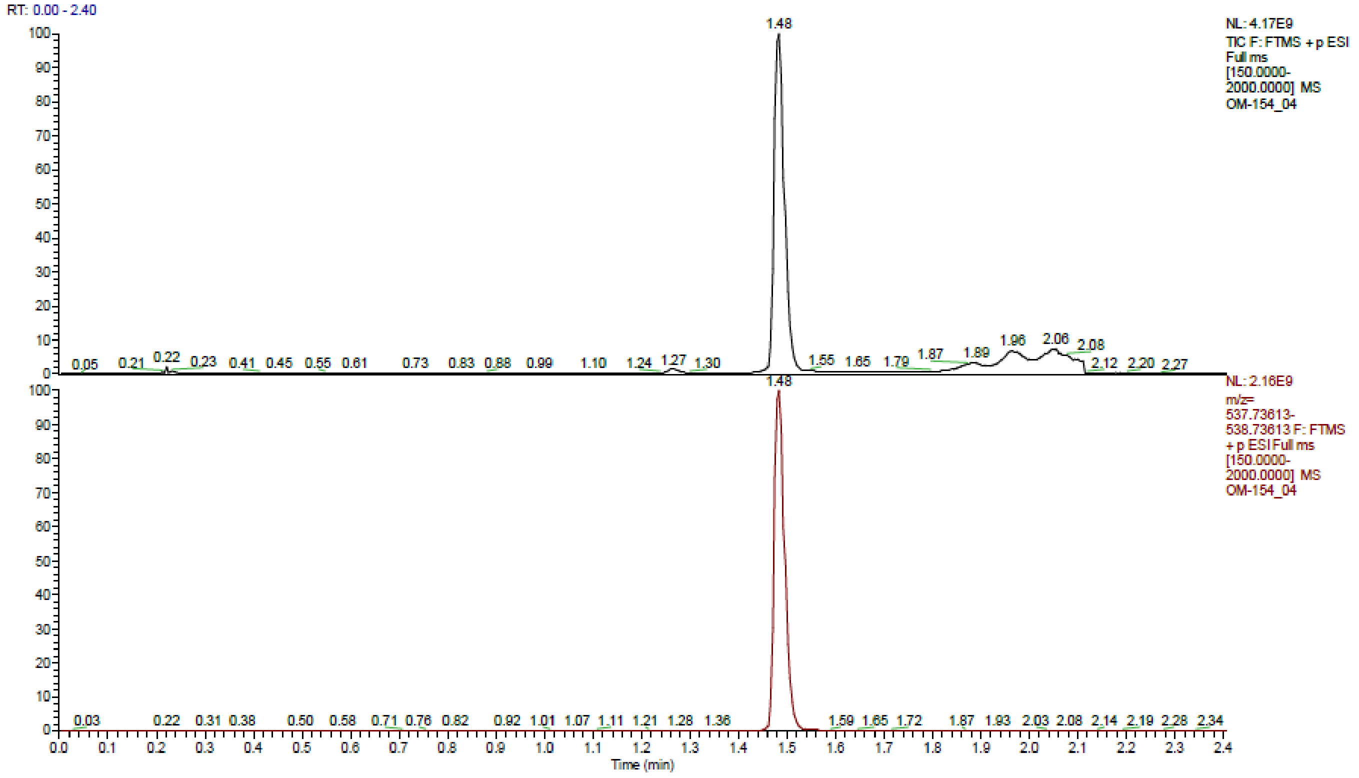

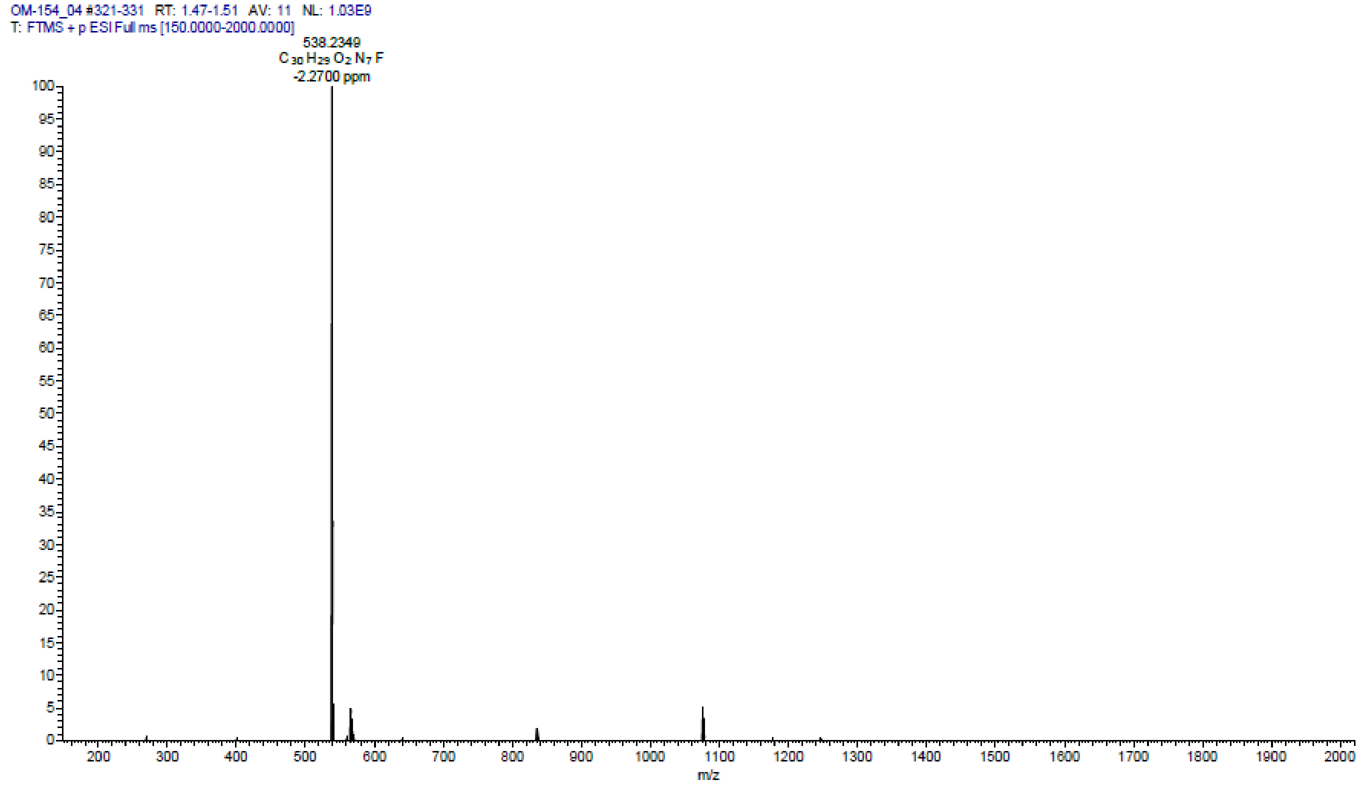

Compound 29a

**title**	**PINI23-66-1**
**Method**	AN_ACID.M
**Date acquired**	30-Oct-18, 16:45:50
**FileName**	Analysis\LCMS6_1030_092.D
**Column**	XSelect CSH C18 (50x2.1mm, 3.5µ) valve:3
**Flow**	0.8 ml/min; Column temp: 35°C
**Eluent A**	0.1% formic acid in acetonitrile
**Eluent B**	0.1% formic acid in water
**Gradient**	t=0 min 5% A, t=3.5 min 98% A, t=6 min 98%A
**Posttime**	2 min
**Detection**	DAD(210, 220 and 220-320nm)
**Detection**	PDA(210-320nm)
**Detection**	MSD (ESI pos/neg) mass range: 100 - 1000
**Detection**	ELSD gas temp: 40°C, flow 1.5 ml/min, gain 1

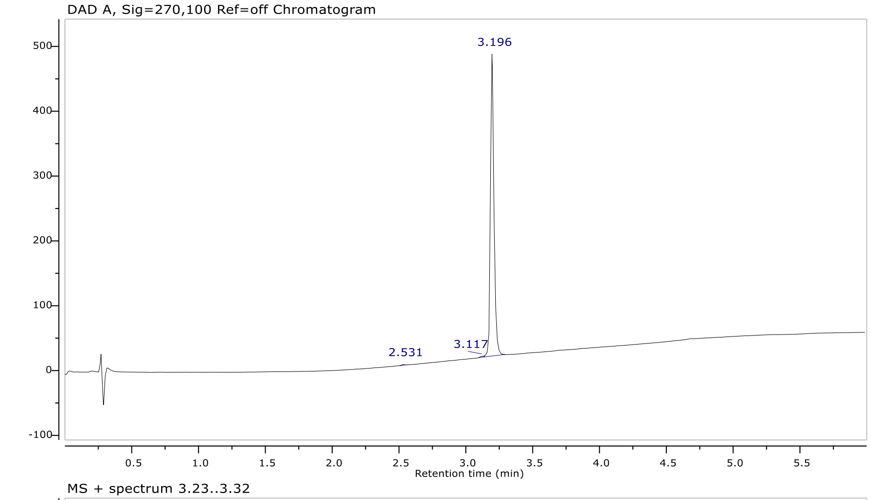

Integrals spectrum **Chromatogram DAD A, Sig=270,100 Ref=off**

rt (min)	height	area	area (%)
2.53	1.176	0.02484	0.17
3.12	1.485	0.04052	0.28
3.20	465.6	14.27	99.54

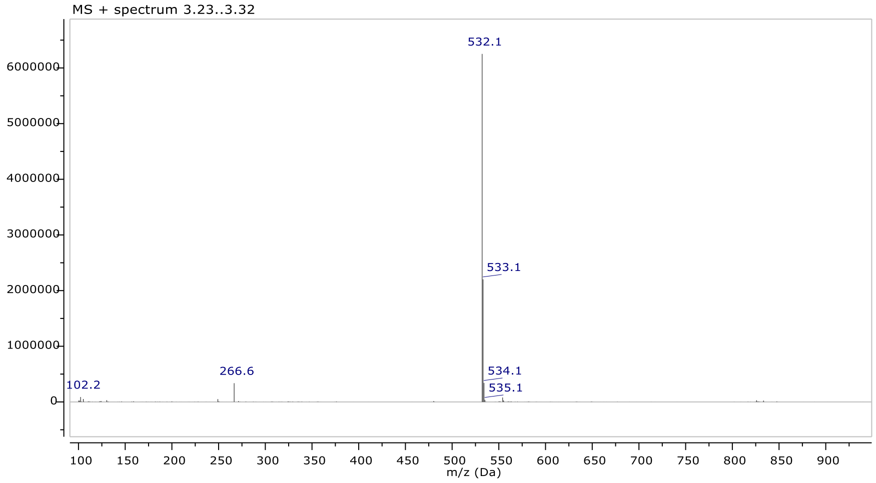

**Title**	**PINI23-66-1**
**Data File Name**	1031_120
**Origin**	Bruker BioSpin GmbH
**Method**	
**Pulse Sequence**	zg30
**Relaxation Delay**	1 s
**Solvent**	CDCl3
**Acquisition Date**	2018-10-31T09:56:02
**Temperature**	~ 298.06 K
**Number of Scans**	32
**Frequency**	400.232471584084 MHz
**Nucleus**	1H

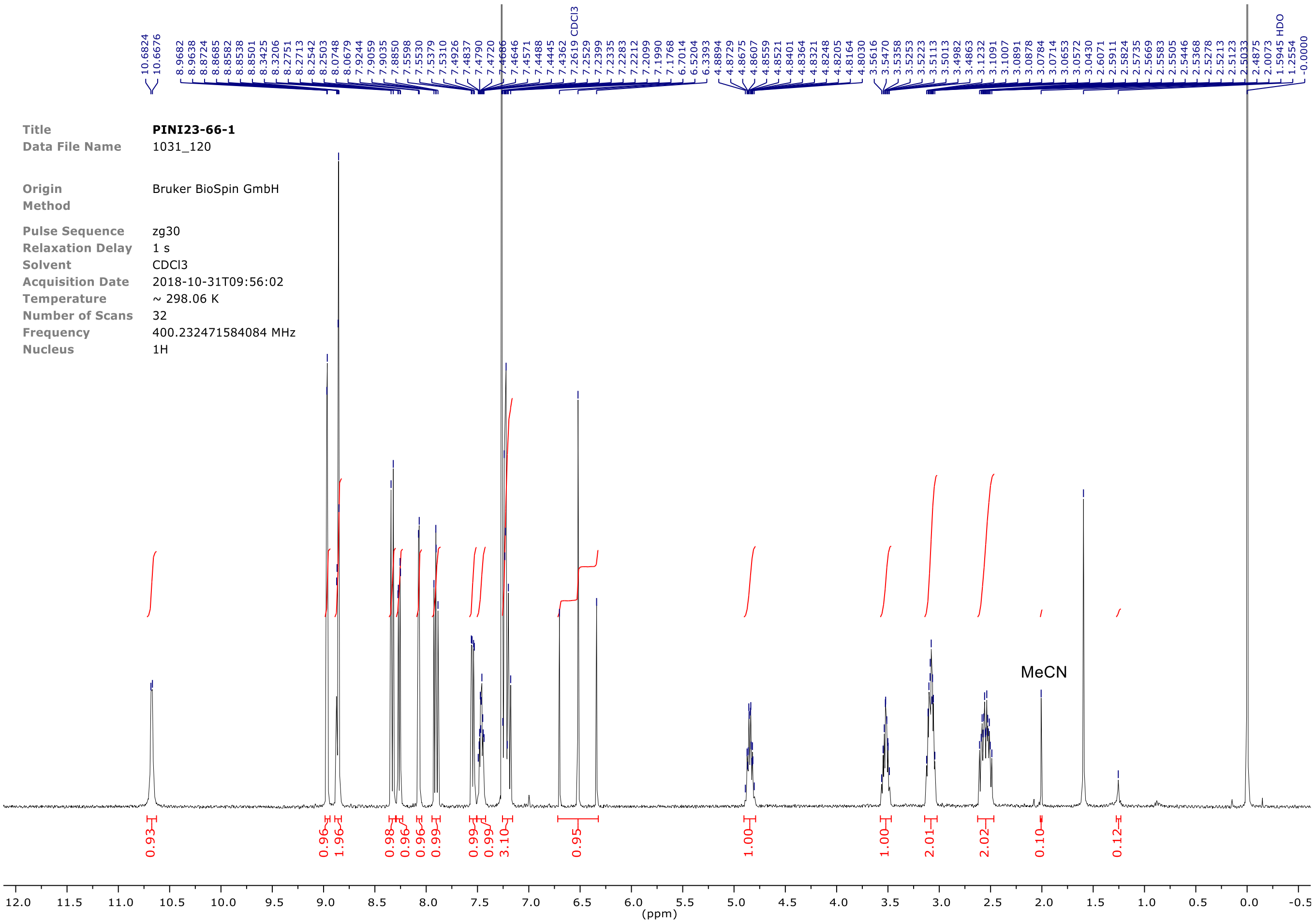

HRMS (m/z): C27H20N7O2F3, [M+H]+ Calc: 532.17033; found 532.1692, Δppm -2.12

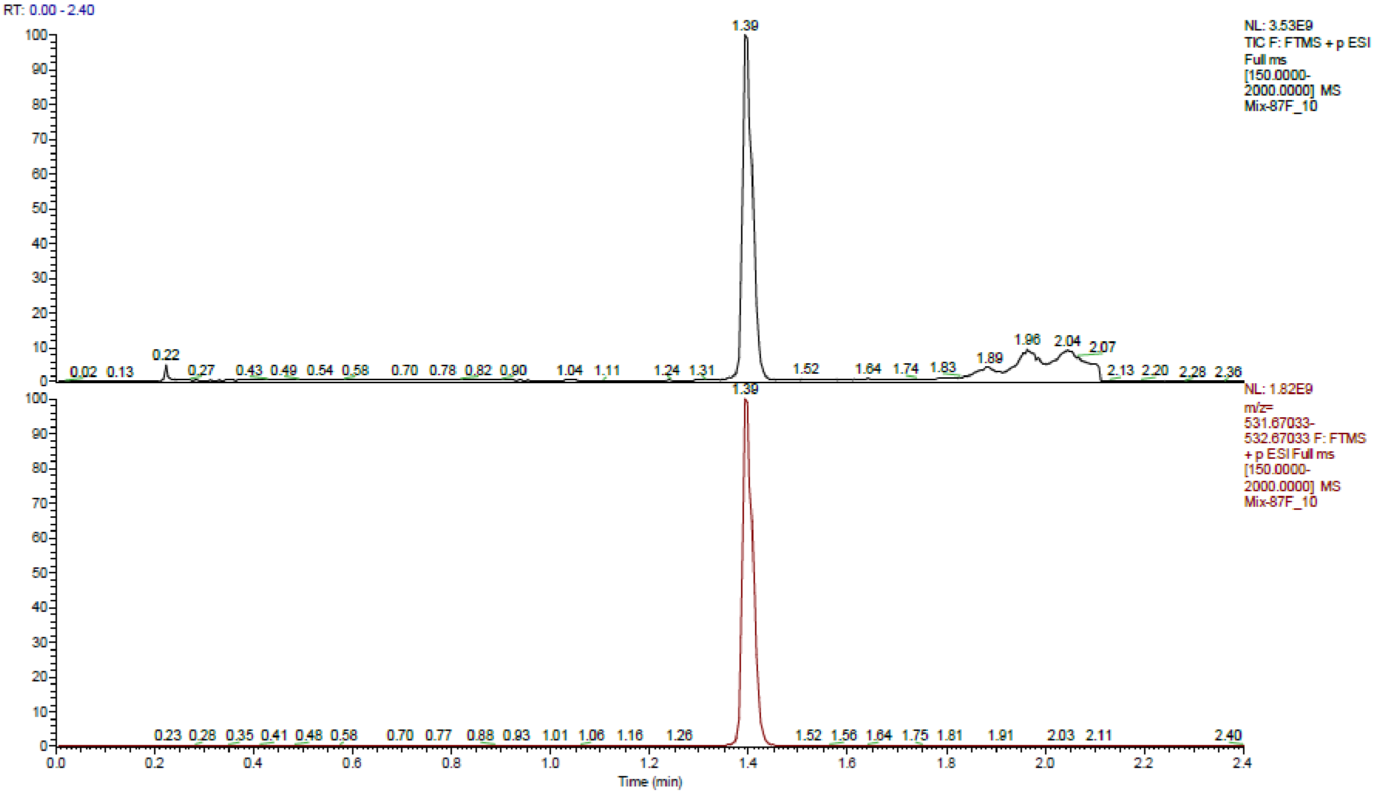

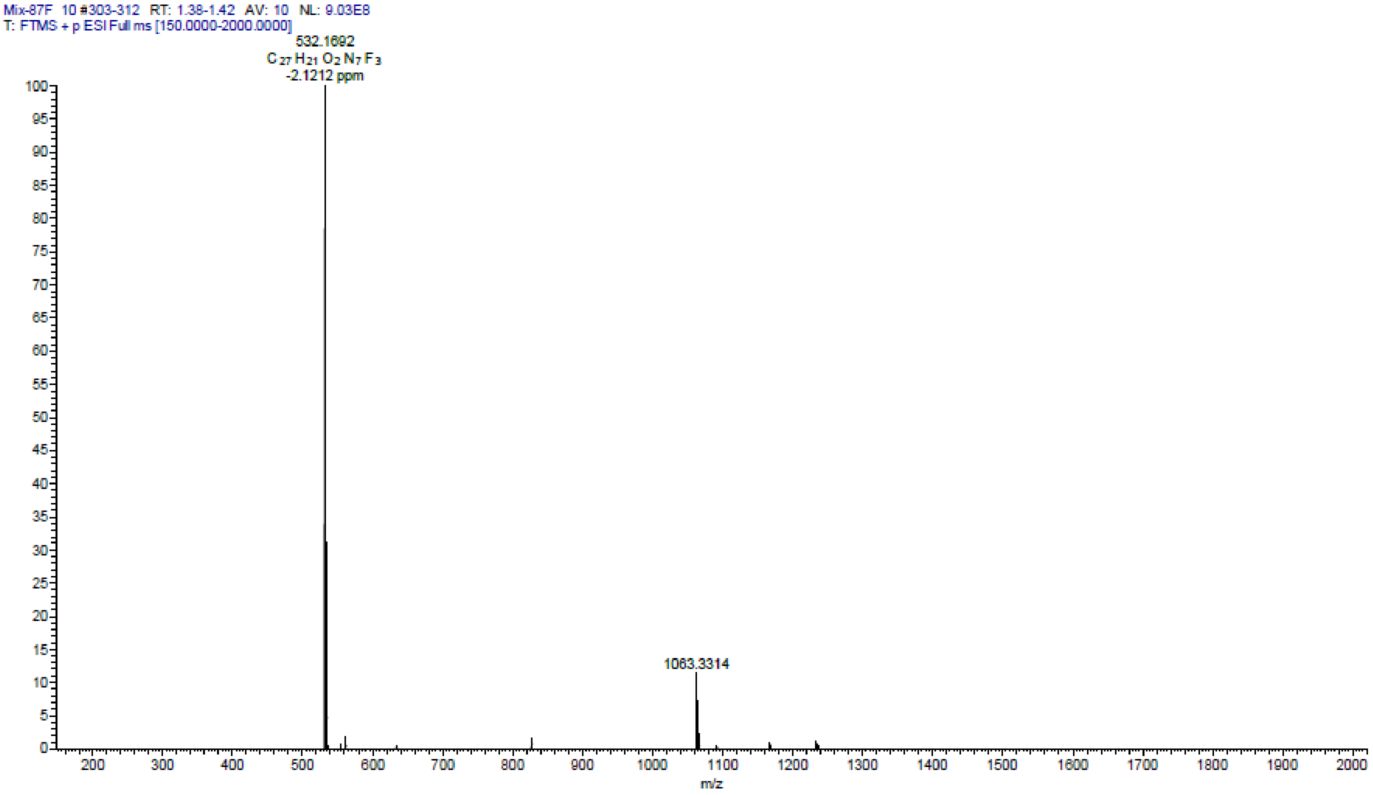

Compound 29b

title	**PINI23-69-1**
Method	AN_ACID.M
Date acquired	01-Nov-18, 17:02:08
FileName	Analysis\LCMS6_1101_131.D
Column	XSelect CSH C18 (50x2.1mm, 3.5μ) valve:3
Flow	0.8 ml/min; Column temp: 35°C
Eluent A	0.1% formic acid in acetonitrile
Eluent B	0.1% formic acid in water
Gradient	t=0 min 5% A, t=3.5 min 98% A, t=6 min 98%A
Posttime	2 min
Detection	DAD(210, 220 and 220-320nm)
Detection	PDA(210-320nm)
Detection	MSD (ESI pos/neg) mass range: 100 - 1000
Detection	ELSD gas temp: 40°C, flow 1.5 ml/min, gain 1

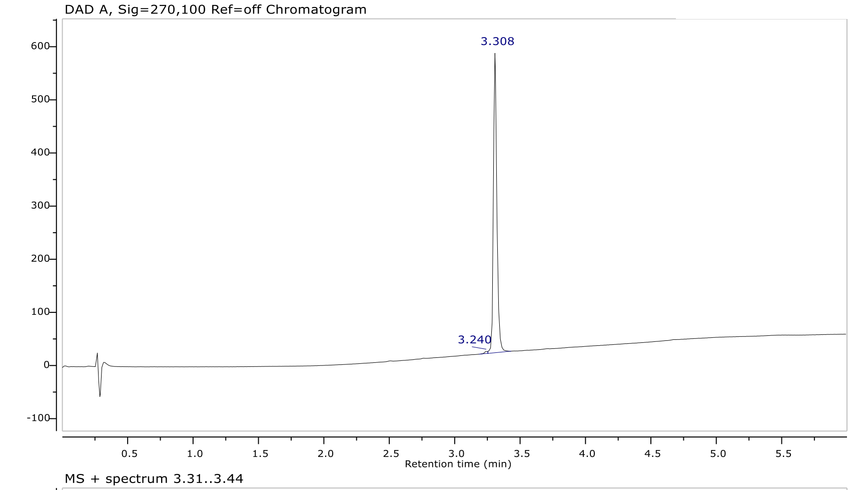

Integrals spectrum — Chromatogram DAD A, Sig=270,100 Ref=off

rt (min)	height	area	area (%)
3.24	3.732	0.1076	0.62
3.31	563.8	17.13	99.38

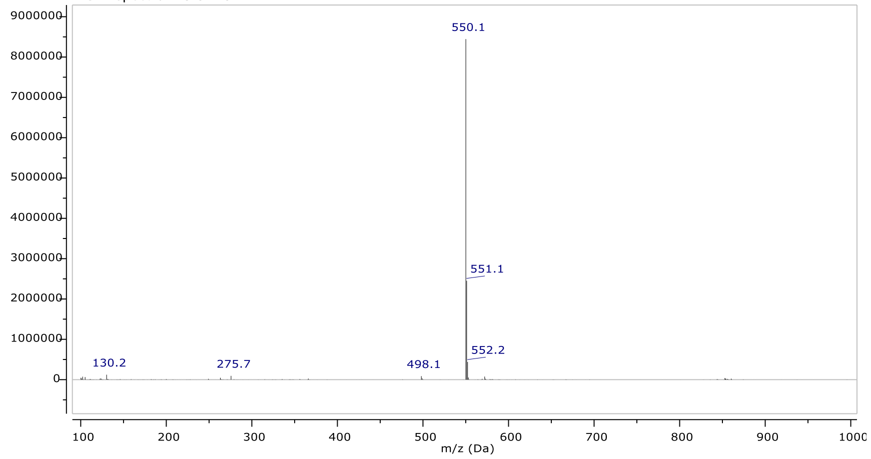

Title	**PINI23-69-1**
Data File Name	1102_200
Origin	Bruker BioSpin GmbH
Method	
Pulse Sequence	zg30
Relaxation Delay	1 s
Solvent	CDCl3
Acquisition Date	2018-11-02T11:06:20
Temperature	~ 297.86 K
Number of Scans	32
Frequency	400.232471584084 MHz
Nucleus	1H

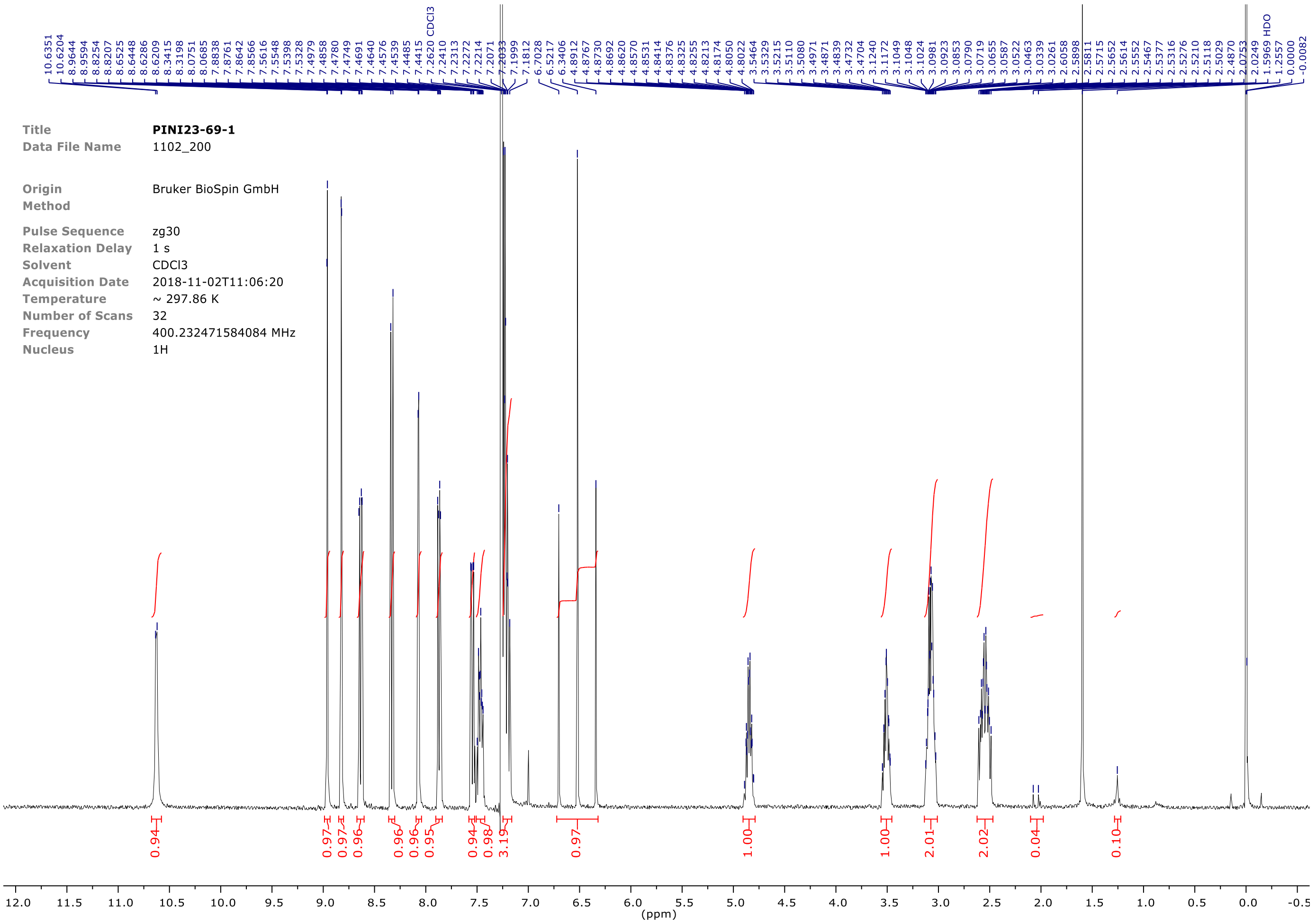

HRMS (m/z): C27H19N7O2F4, [M+H]+ Calc: 550.16091; found: 550.1597, Δppm -2.25

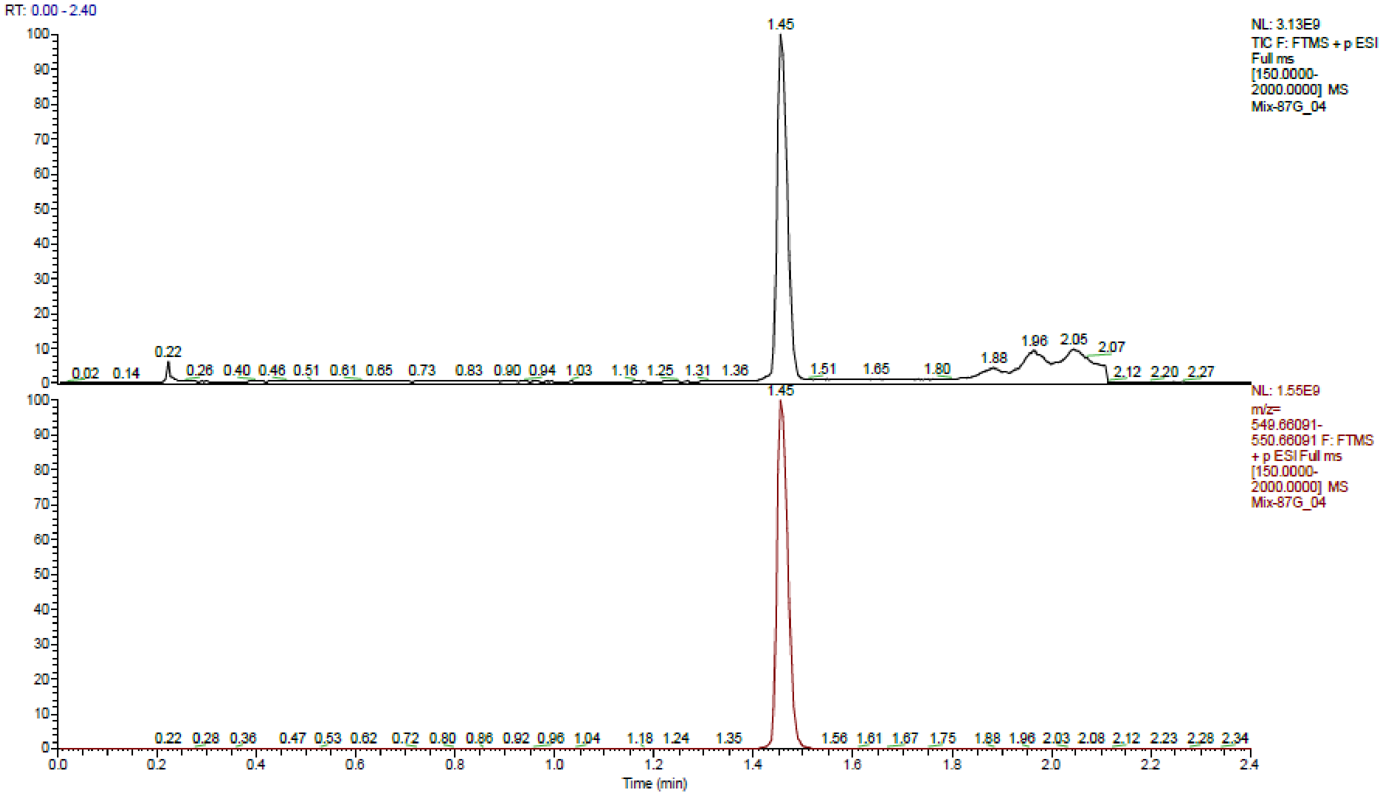

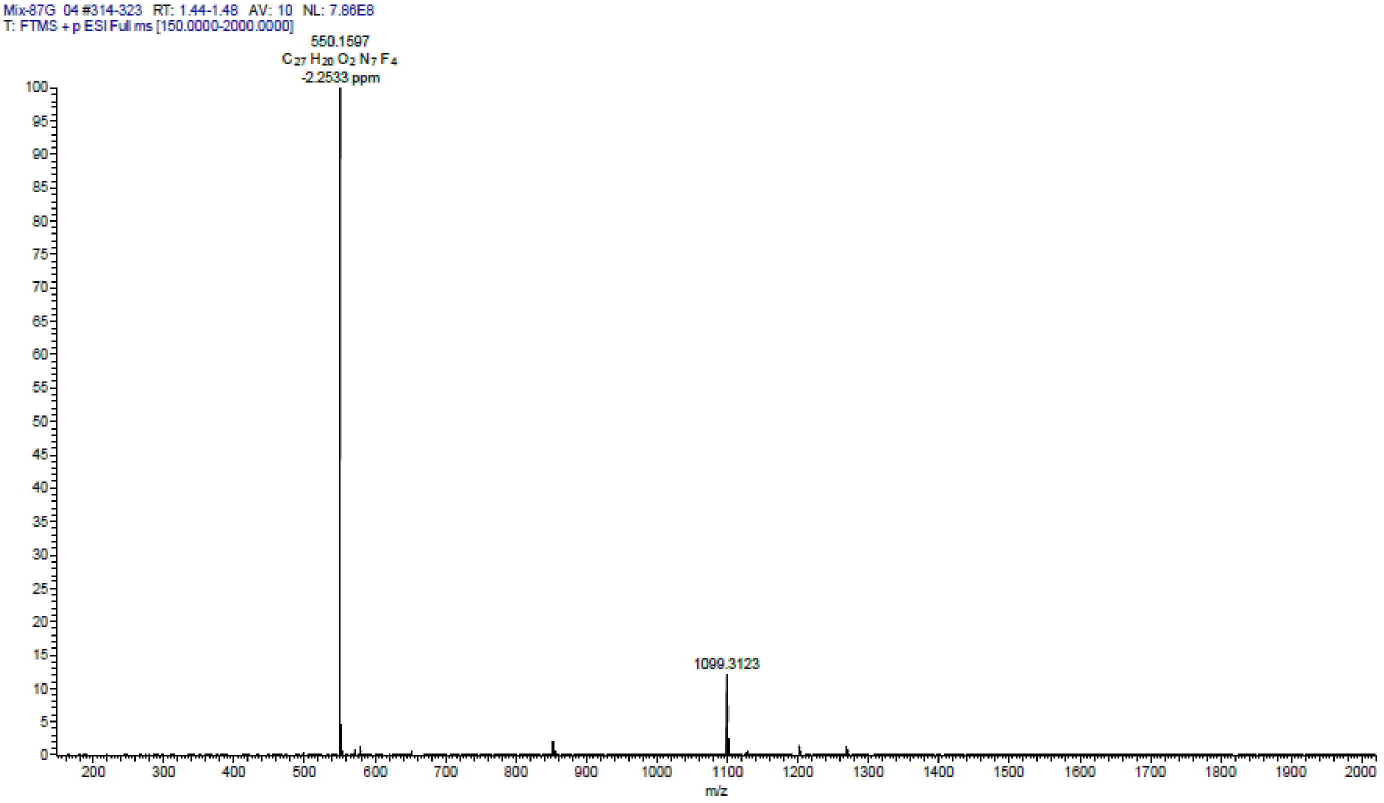

Compound 30a

**title**	**PINI23-64-1**
**Method**	AN_ACID.M
**Date acquired**	30-Oct-18, 16:29:06
**FileName**	Analysis\LCMS6_1030_090.D
**Column**	XSelect CSH C18 (50x2.1mm, 3.5μ) valve:3
**Flow**	0.8 ml/min; Column temp: 35°C
**Eluent A**	0.1% formic acid in acetonitrile
**Eluent B**	0.1% formic acid in water
**Gradient**	t=0 min 5% A, t=3.5 min 98% A, t=6 min 98%A
**Posttime**	2 min
**Detection**	DAD(210, 220 and 220-320nm)
**Detection**	PDA(210-320nm)
**Detection**	MSD (ESI pos/neg) mass range: 100 - 1000
**Detection**	ELSD gas temp: 40°C, flow 1.5 ml/min, gain 1

DAD A, Sig=270,100 Ref=off Chromatogram

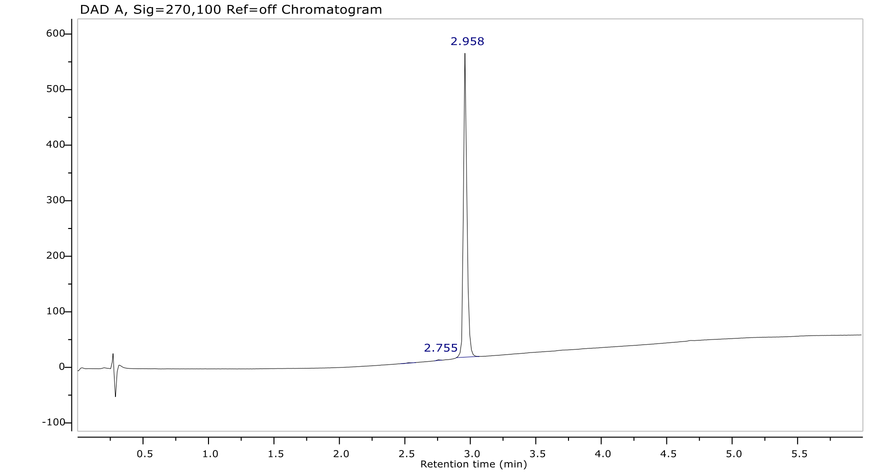

Integrals spectrum — **Chromatogram DAD A, Sig=270,100 Ref=off**

rt (min)	height	area	area (%)
2.53	0.8157	0.02801	0.16
2.76	1.220	0.02818	0.16
2.96	547.1	17.07	99.67

MS + spectrum 2.99..3.09

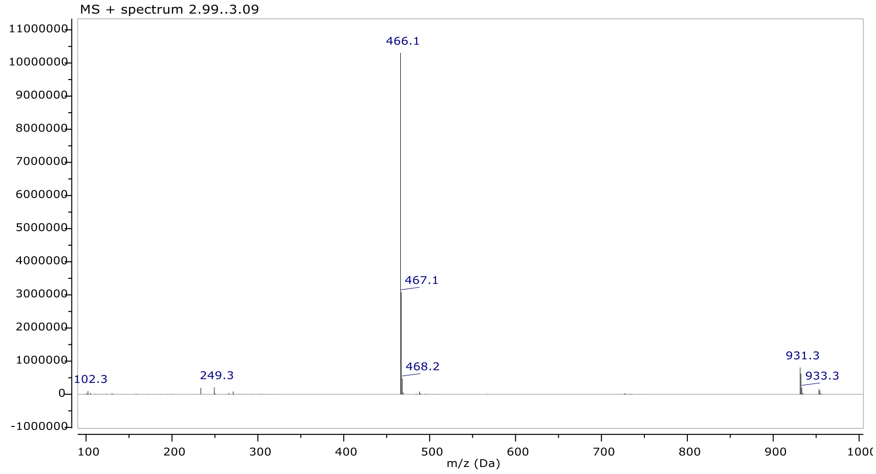

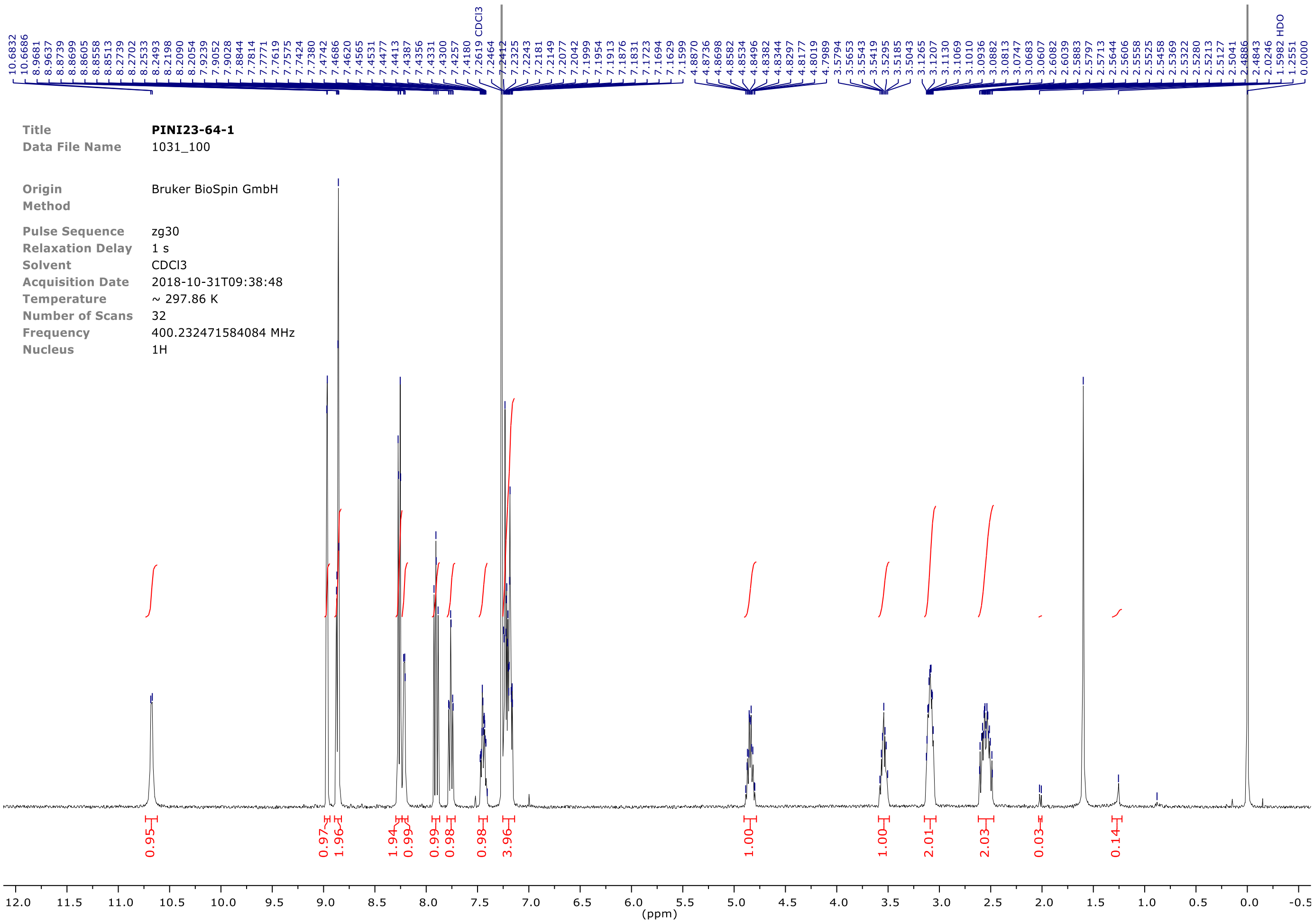

**Title**	**PINI23-64-1**
**Data File Name**	1031_100
**Origin**	Bruker BioSpin GmbH
**Method**	
**Pulse Sequence**	zg30
**Relaxation Delay**	1 s
**Solvent**	CDCl3
**Acquisition Date**	2018-10-31T09:38:48
**Temperature**	~ 297.86 K
**Number of Scans**	32
**Frequency**	400.232471584084 MHz
**Nucleus**	1H

HRMS (m/z): C26H20N7OF, [M+H]+ Calc: 466.17861; found: 466.1775, Δppm -2.34

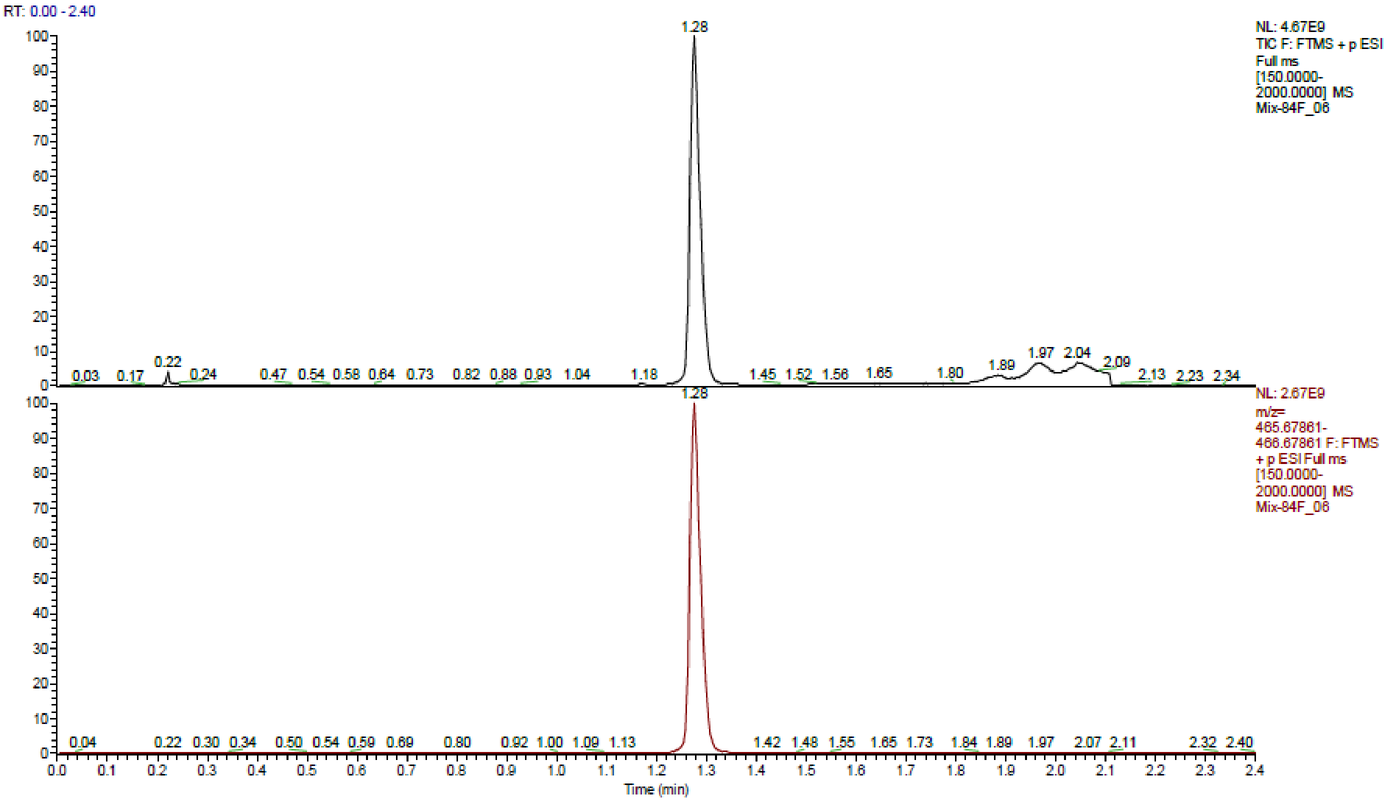

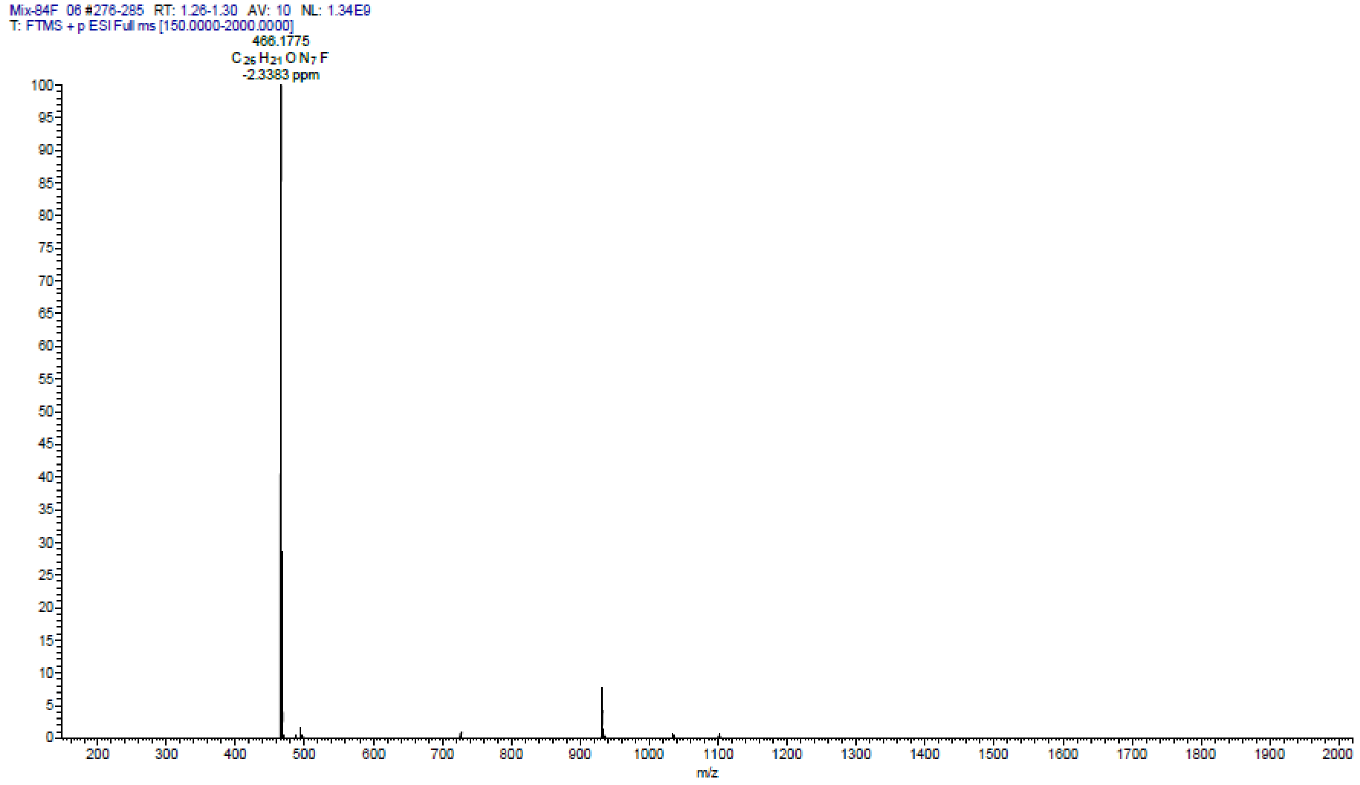

Compound 30b

title	**PINI23-67-1**
Method	AN_ACID.M
Date acquired	01-Nov-18, 16:45:23
FileName	Analysis\LCMS6_1101_129.D
Column	XSelect CSH C18 (50x2.1mm, 3.5μ) valve:3
Flow	0.8 ml/min; Column temp: 35°C
Eluent A	0.1% formic acid in acetonitrile
Eluent B	0.1% formic acid in water
Gradient	t=0 min 5% A, t=3.5 min 98% A, t=6 min 98%A
Posttime	2 min
Detection	DAD(210, 220 and 220-320nm)
Detection	PDA(210-320nm)
Detection	MSD (ESI pos/neg) mass range: 100 - 1000
Detection	ELSD gas temp: 40°C, flow 1.5 ml/min, gain 1

Integrals spectrum — Chromatogram DAD A, Sig=270,100 Ref=off

rt (min)	height	area	area (%)
3.08	691.8	21.53	100.00

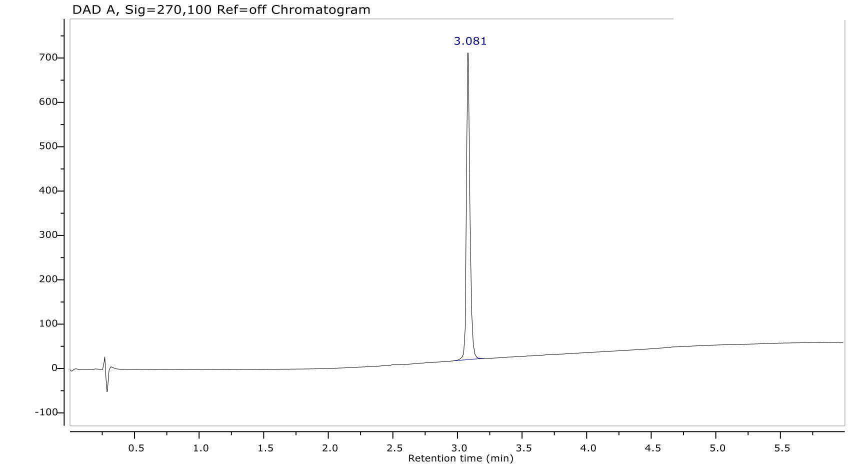

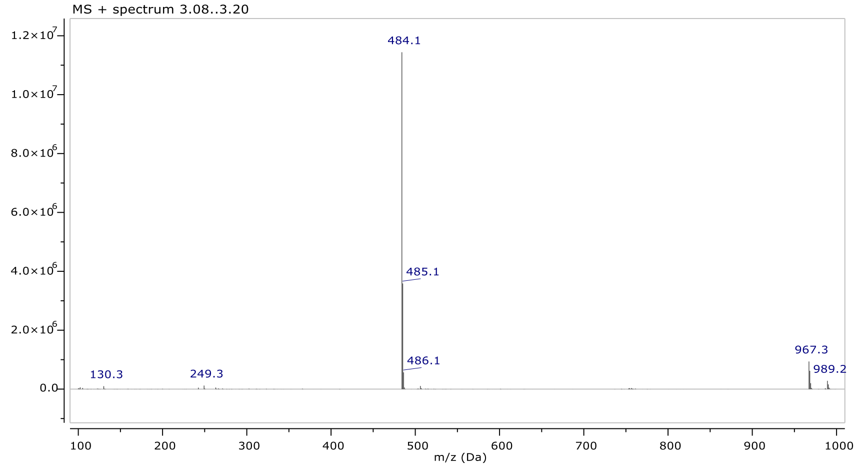

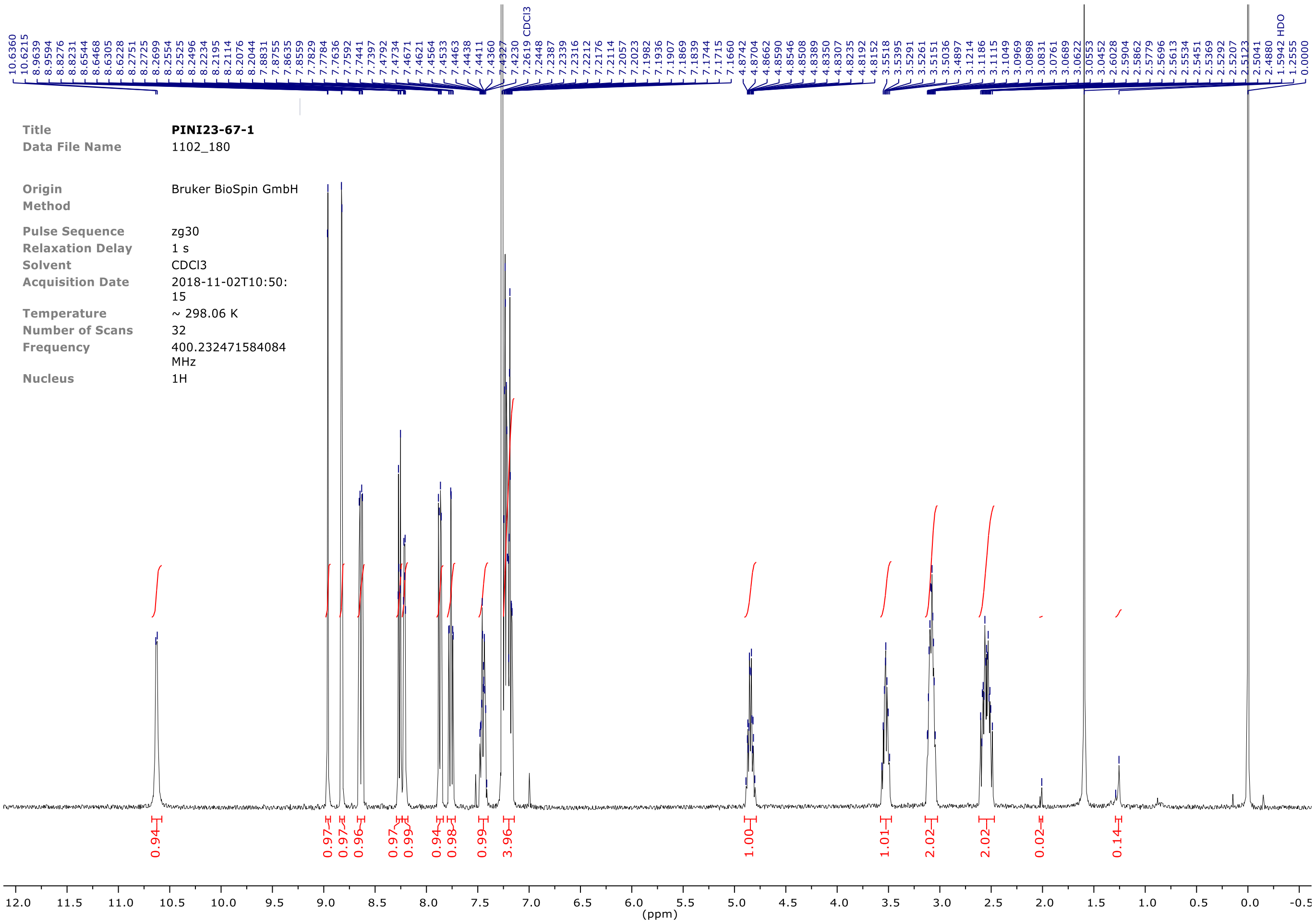

Title	**PINI23-67-1**
Data File Name	1102_180
Origin	Bruker BioSpin GmbH
Method	
Pulse Sequence	zg30
Relaxation Delay	1 s
Solvent	CDCl3
Acquisition Date	2018-11-02T10:50:15
Temperature	~ 298.06 K
Number of Scans	32
Frequency	400.232471584084 MHz
Nucleus	1H

HRMS (m/z): C26H19N7OF2, [M+H]+ Calc: 484.16919; found: 484.1684, Δppm -1.65

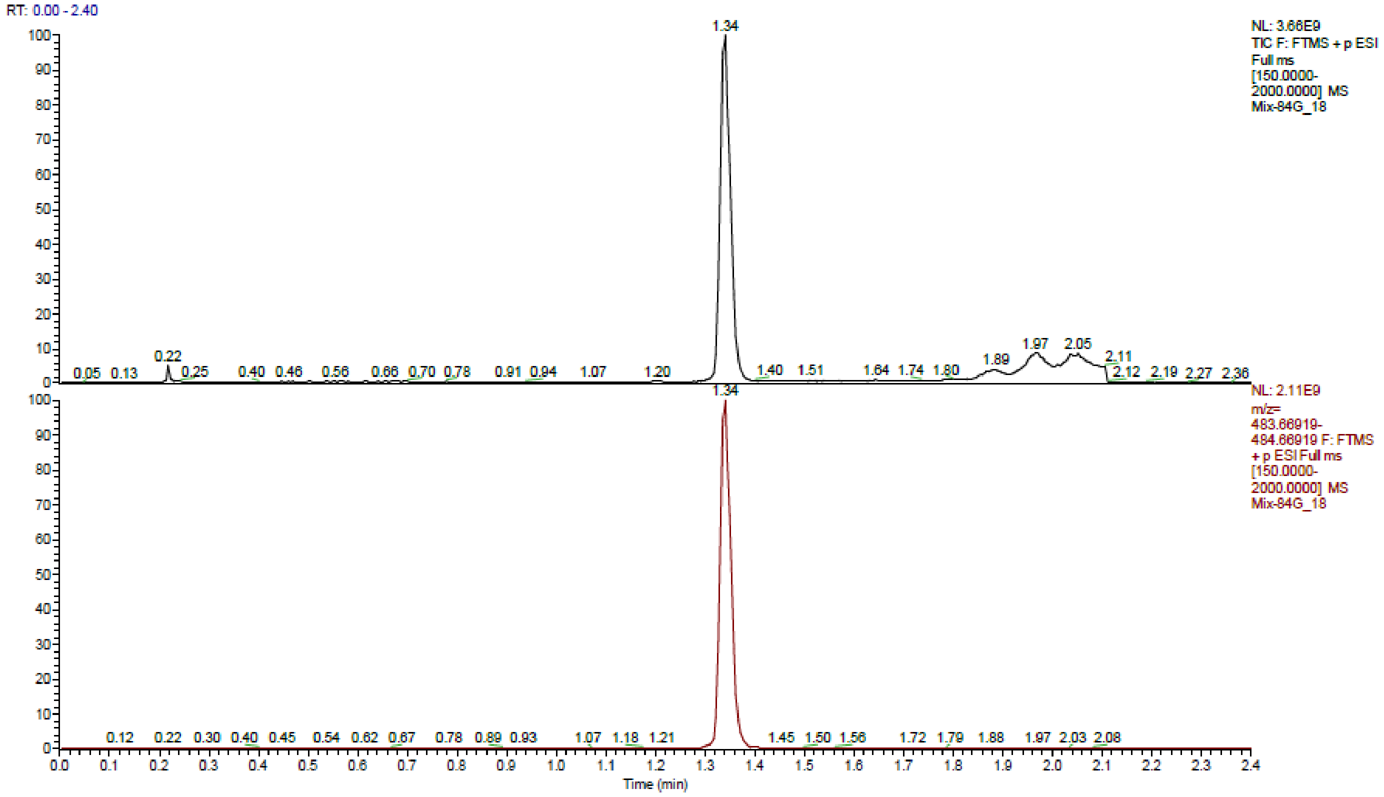

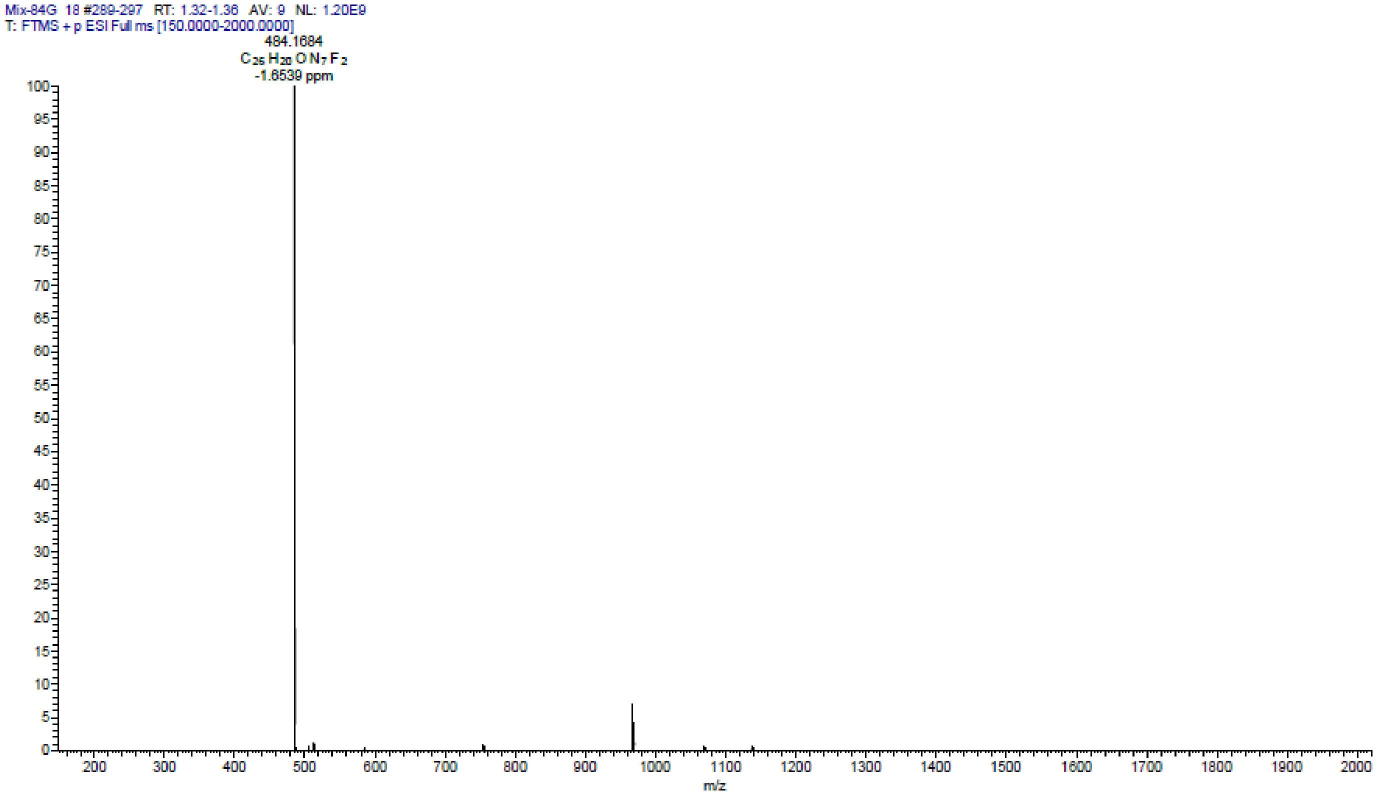

Compound 31a

**title**	**PINI23-65-1**
**Method**	AN_ACID.M
**Date acquired**	30-Oct-18, 16:37:28
**FileName**	Analysis\LCMS6_1030_091.D
**Column**	XSelect CSH C18 (50x2.1mm, 3.5μ) valve:3
**Flow**	0.8 ml/min; Column temp: 35°C
**Eluent A**	0.1% formic acid in acetonitrile
**Eluent B**	0.1% formic acid in water
**Gradient**	t=0 min 5% A, t=3.5 min 98% A, t=6 min 98%A
**Posttime**	2 min
**Detection**	DAD(210, 220 and 220-320nm)
**Detection**	PDA(210-320nm)
**Detection**	MSD (ESI pos/neg) mass range: 100 - 1000
**Detection**	ELSD gas temp: 40°C, flow 1.5 ml/min, gain 1

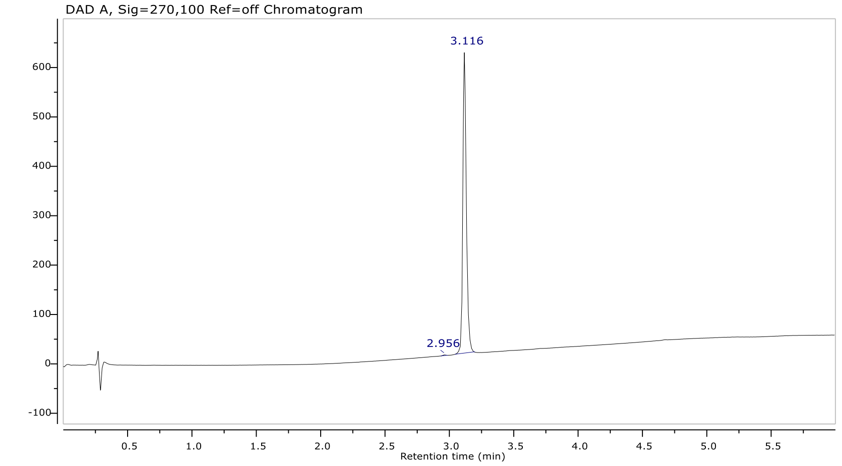

Integrals spectrum — **Chromatogram DAD A, Sig=270,100 Ref=off**

rt (min)	height	area	area (%)
2.96	1.353	0.03264	0.17
3.12	608.6	19.02	99.83

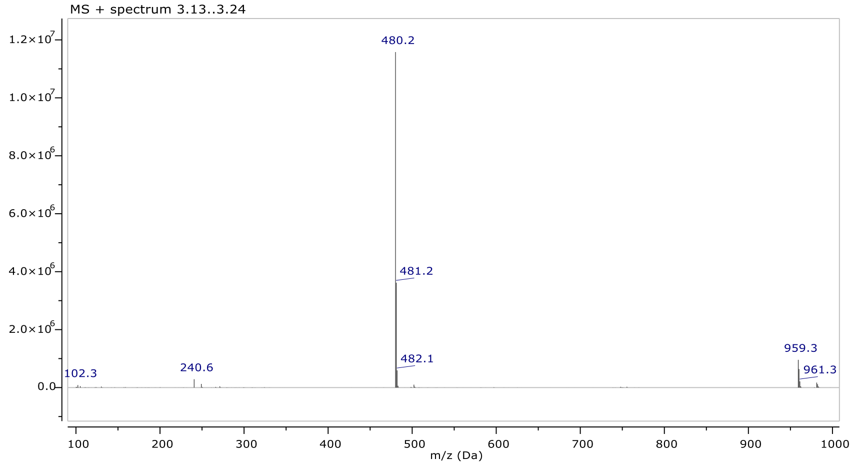

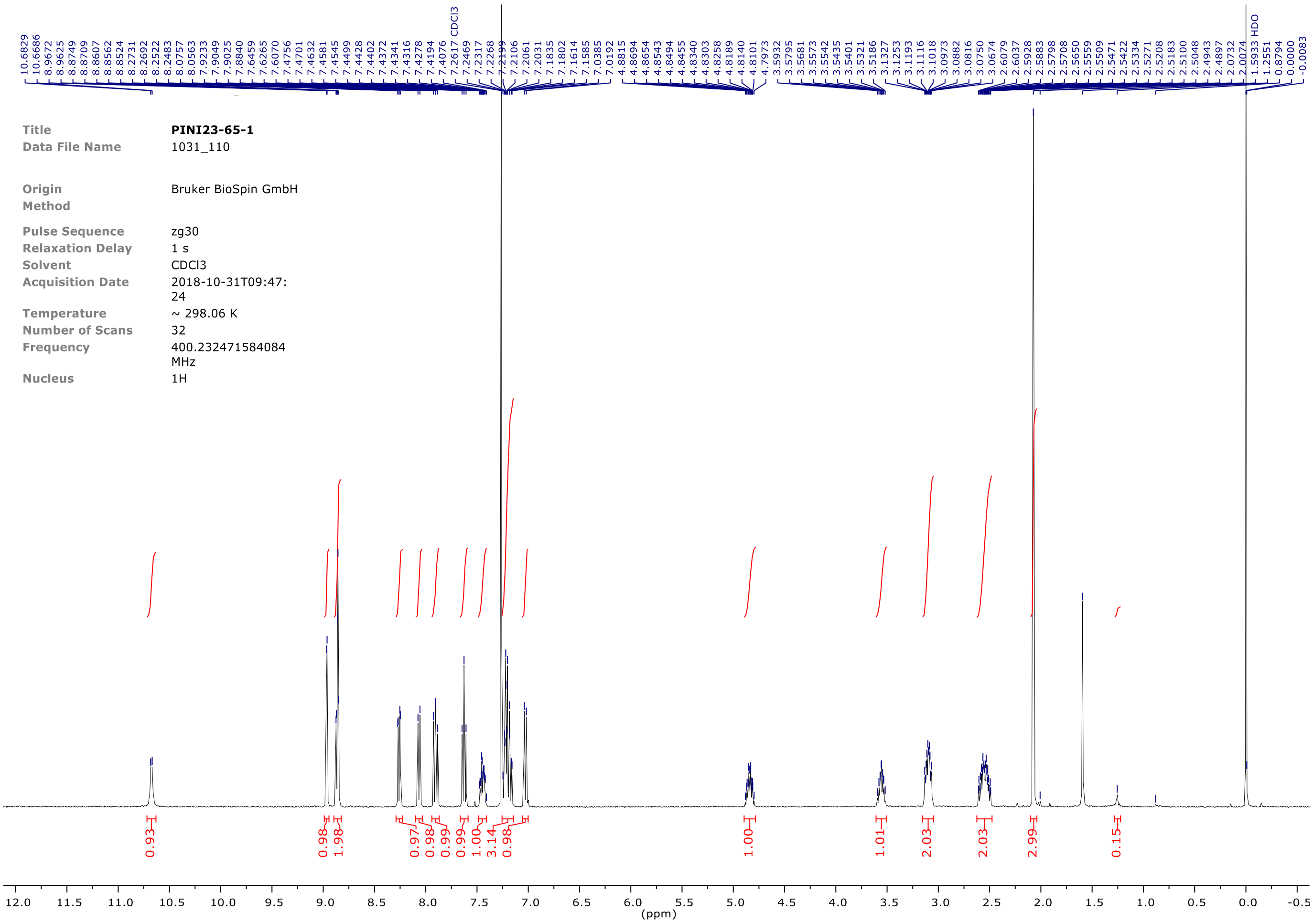

**Title**	**PINI23-65-1**
**Data File Name**	1031_110
**Origin**	Bruker BioSpin GmbH
**Method**	
**Pulse Sequence**	zg30
**Relaxation Delay**	1 s
**Solvent**	CDCl3
**Acquisition Date**	2018-10-31T09:47:24
**Temperature**	~ 298.06 K
**Number of Scans**	32
**Frequency**	400.232471584084 MHz
**Nucleus**	1H

HRMS (m/z): C27H22N7OF, [M+H]+ Calc: 480.19426; found: 480.1934, Δppm -1.75

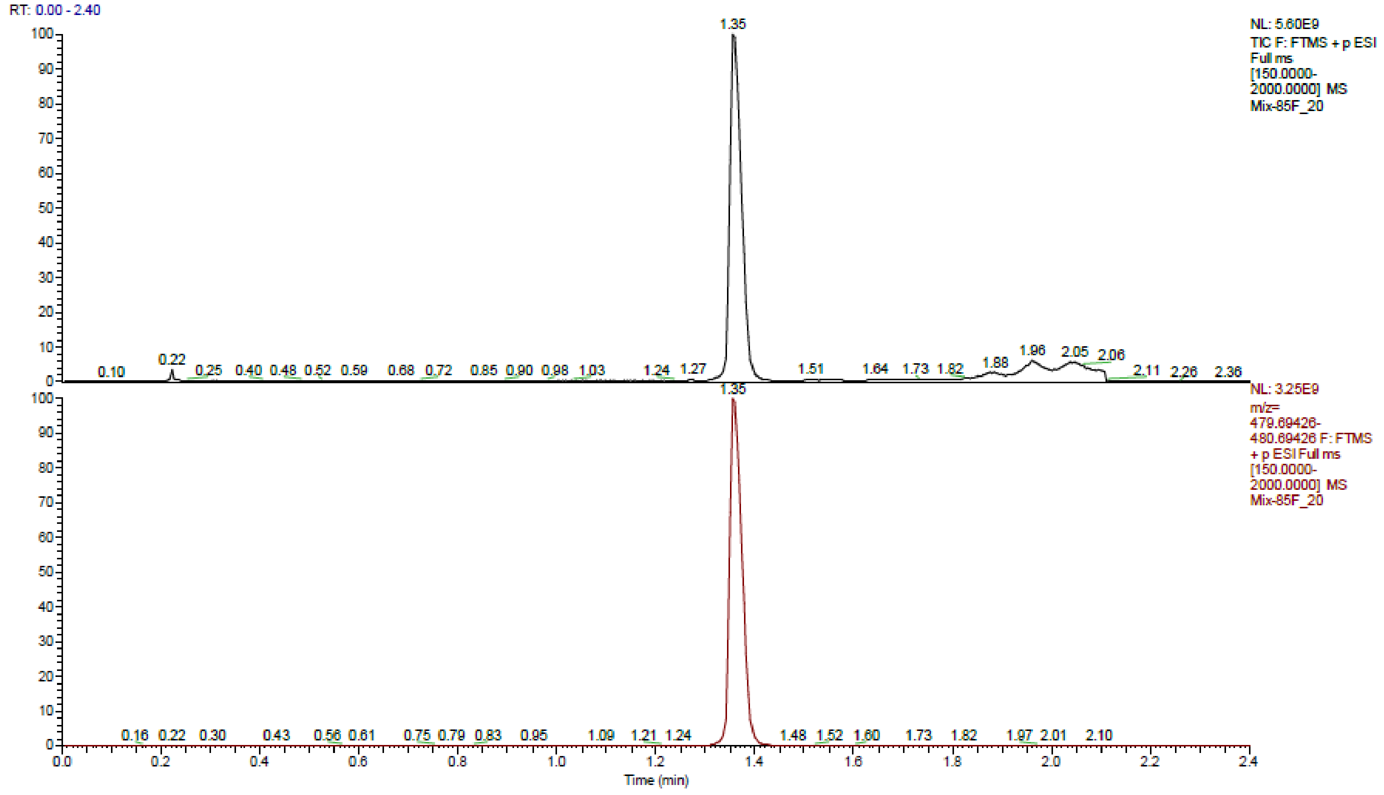

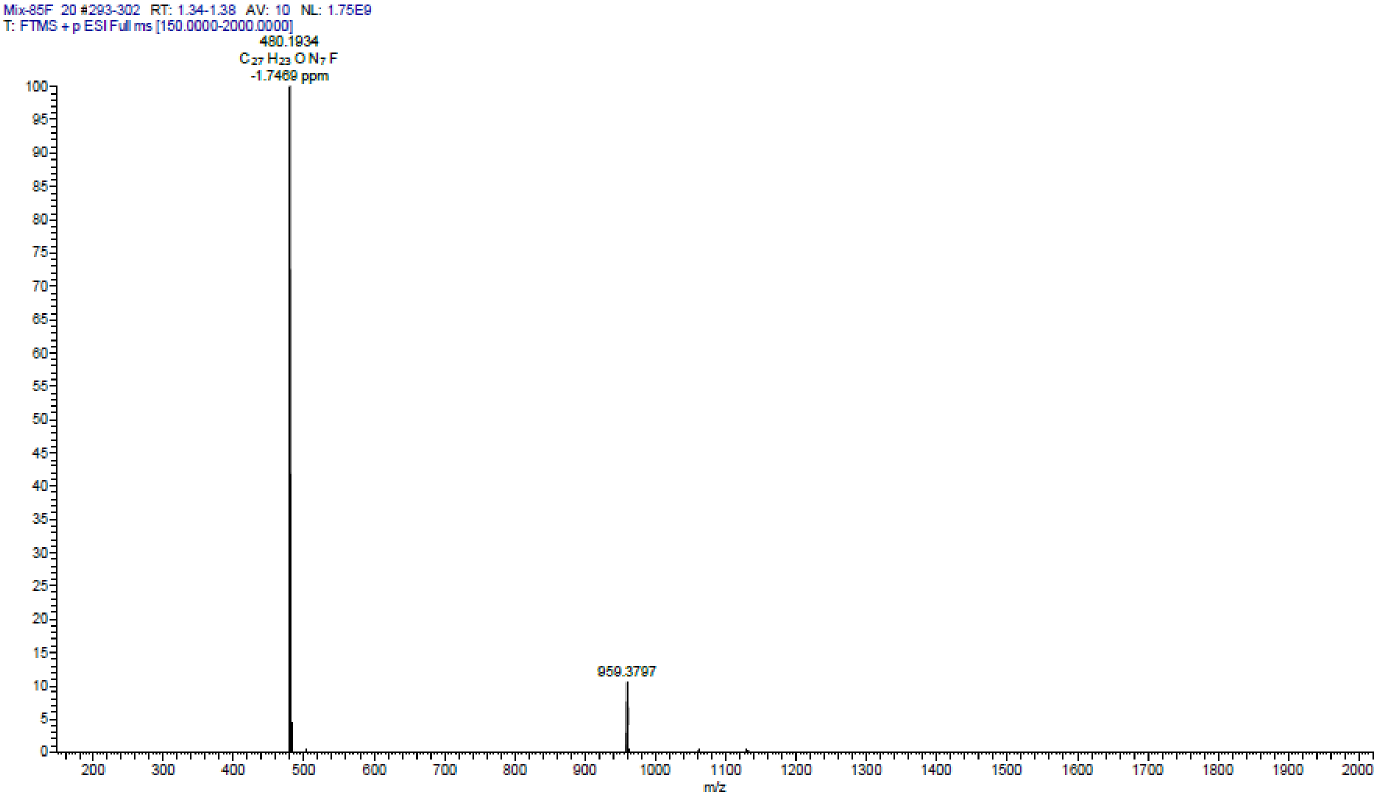

Compound 31b

title	**PINI23-68-1**
Method	AN_ACID.M
Date acquired	01-Nov-18, 16:53:45
FileName	Analysis\LCMS6_1101_130.D
Column	XSelect CSH C18 (50x2.1mm, 3.5µ) valve:3
Flow	0.8 ml/min; Column temp: 35°C
Eluent A	0.1% formic acid in acetonitrile
Eluent B	0.1% formic acid in water
Gradient	t=0 min 5% A, t=3.5 min 98% A, t=6 min 98%A
Posttime	2 min
Detection	DAD(210, 220 and 220-320nm)
Detection	PDA(210-320nm)
Detection	MSD (ESI pos/neg) mass range: 100 - 1000
Detection	ELSD gas temp: 40°C, flow 1.5 ml/min, gain 1

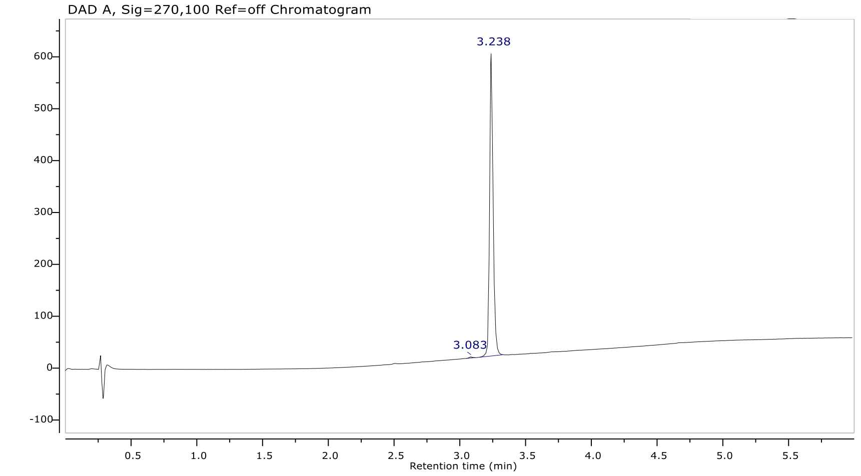

Integrals spectrum — Chromatogram DAD A, Sig=270,100 Ref=off

rt (min)	height	area	area (%)
3.08	2.177	0.05783	0.32
3.24	583.1	18.02	99.68

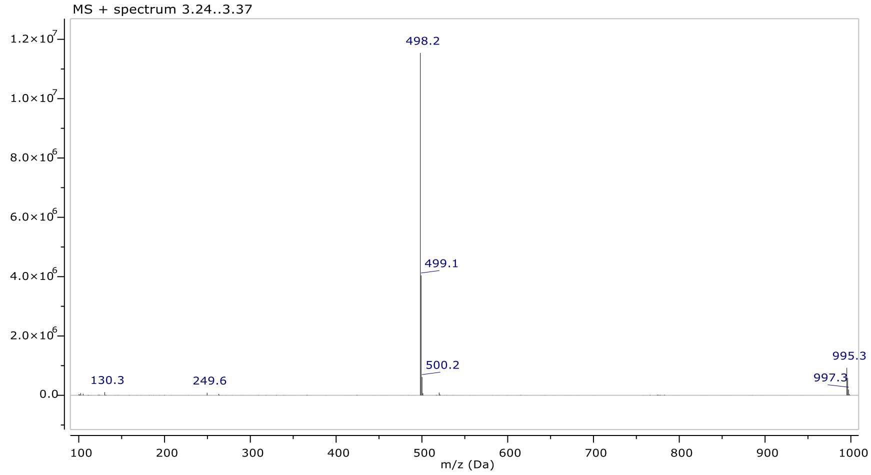

Title	**PINI23-68-1**
Data File Name	1102_190
Origin	Bruker BioSpin GmbH
Method	
Pulse Sequence	zg30
Relaxation Delay	1 s
Solvent	CDCl3
Acquisition Date	2018-11-02T10:58:19
Temperature	~ 297.76 K
Number of Scans	32
Frequency	400.232471584084 MHz
Nucleus	1H

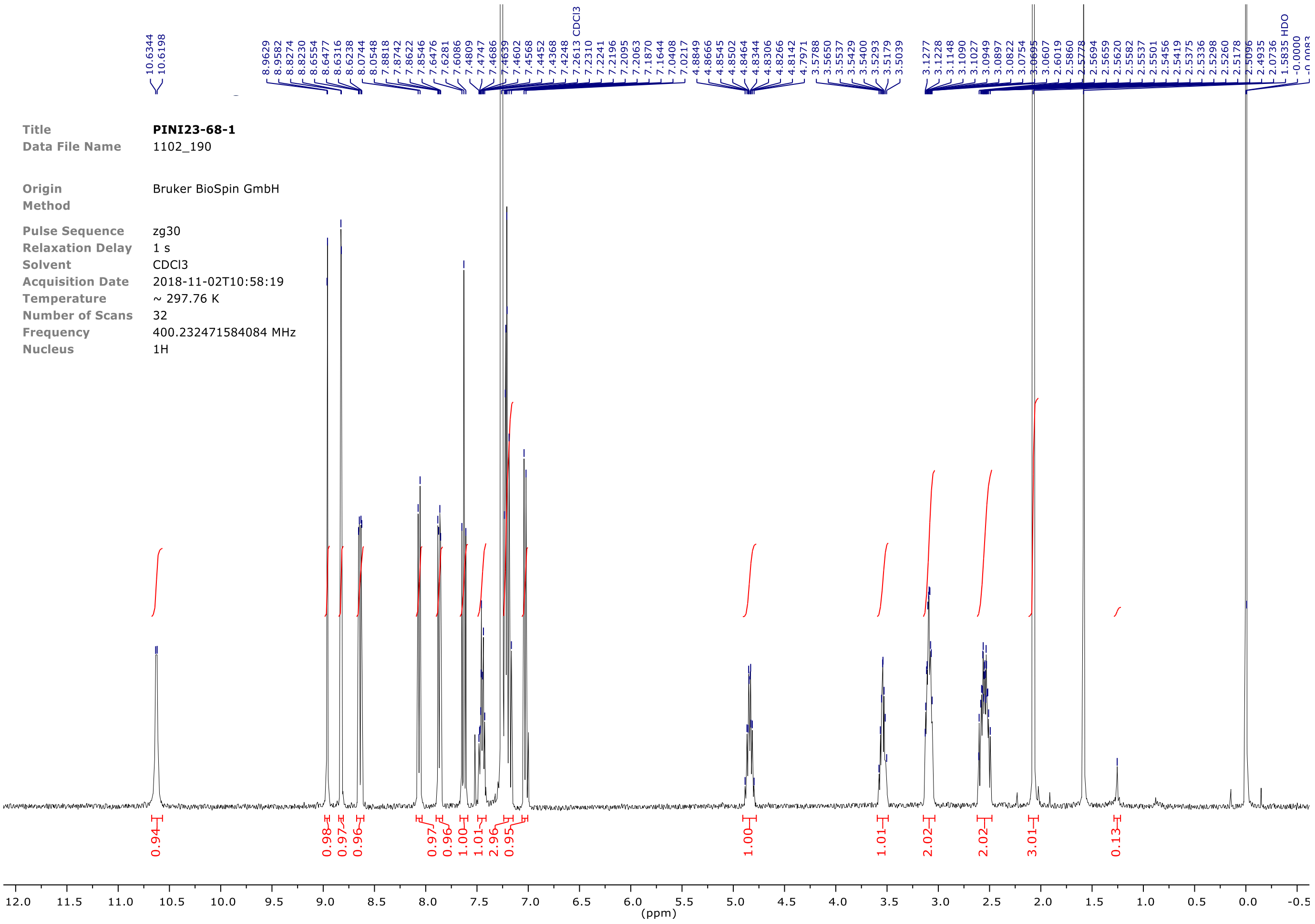

HRMS (m/z): C27H21N7OF2, [M+H]+ Calc: 498.18484; found: 498.1838, Δppm -2.11

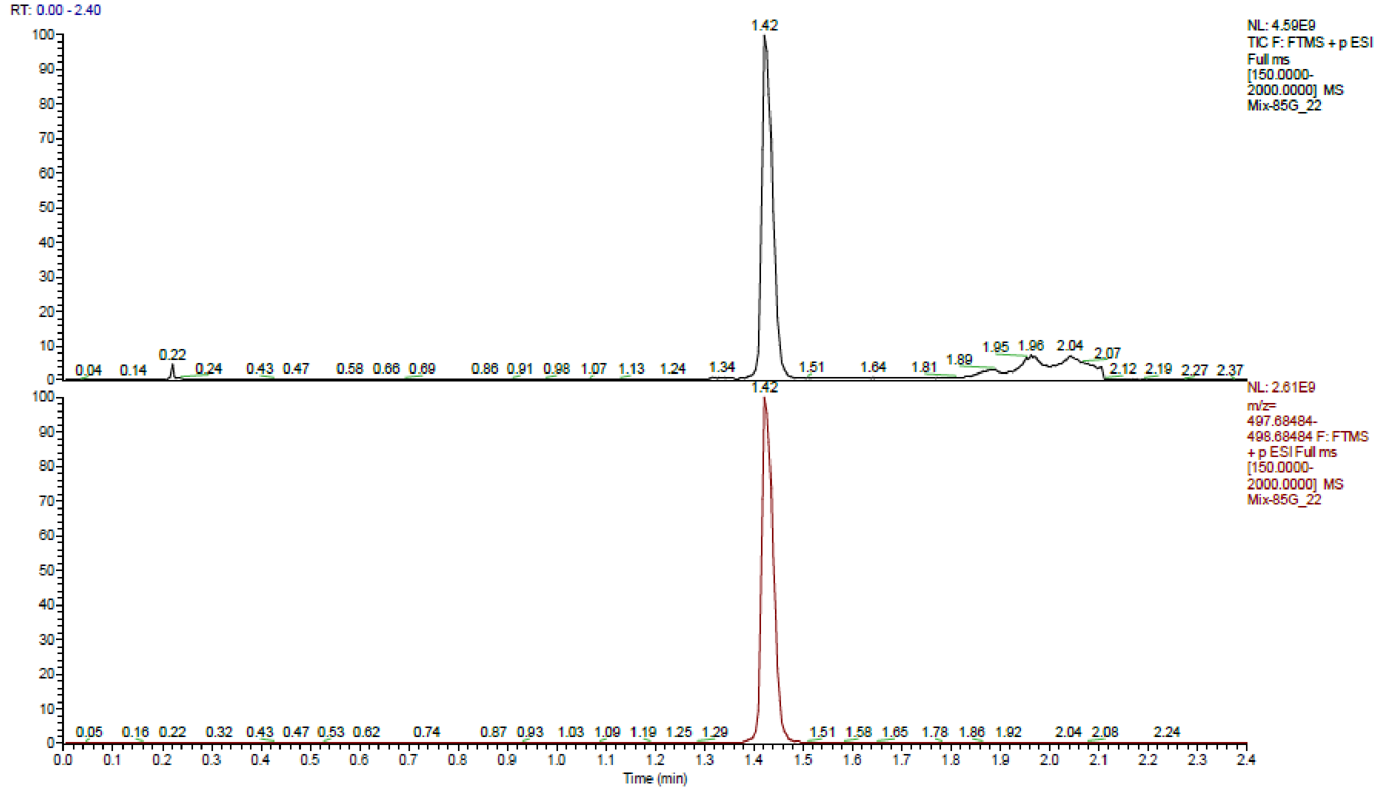

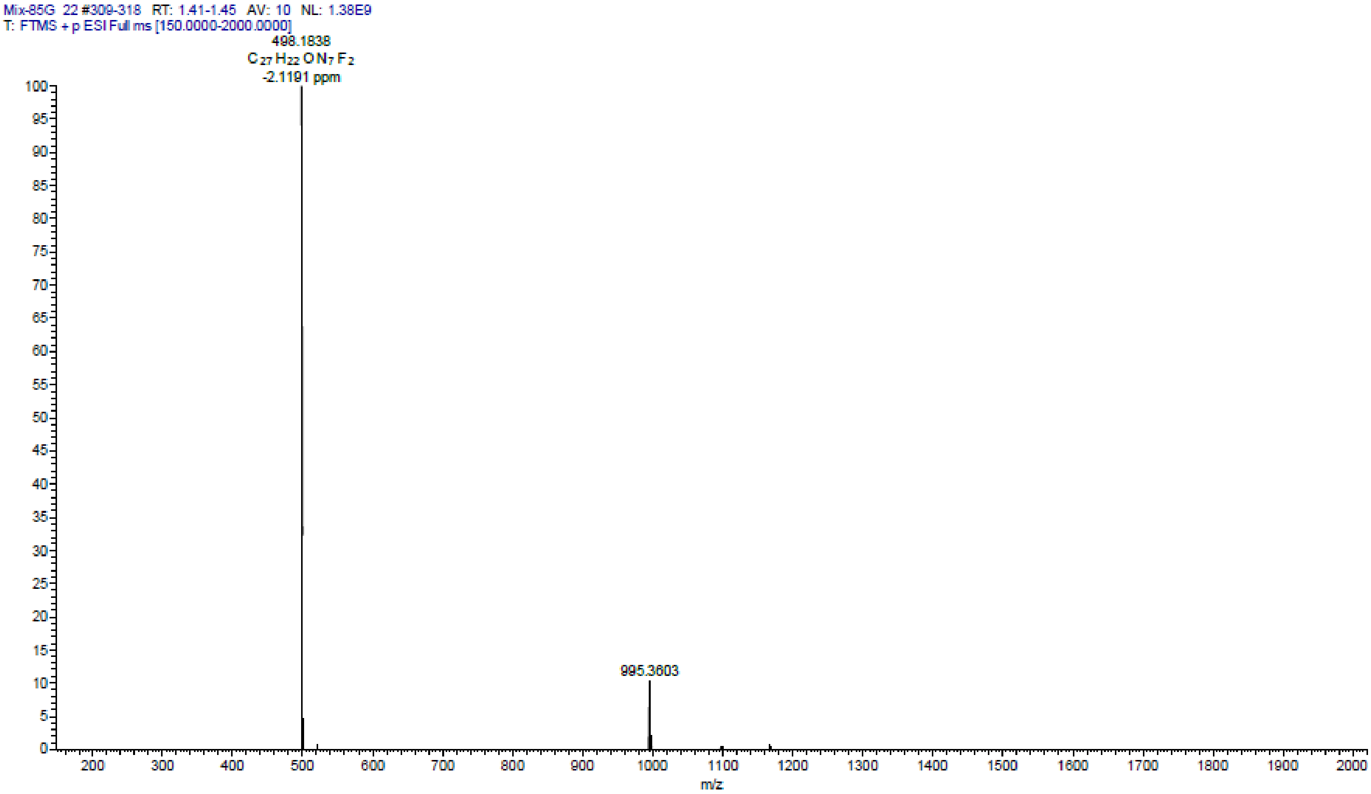

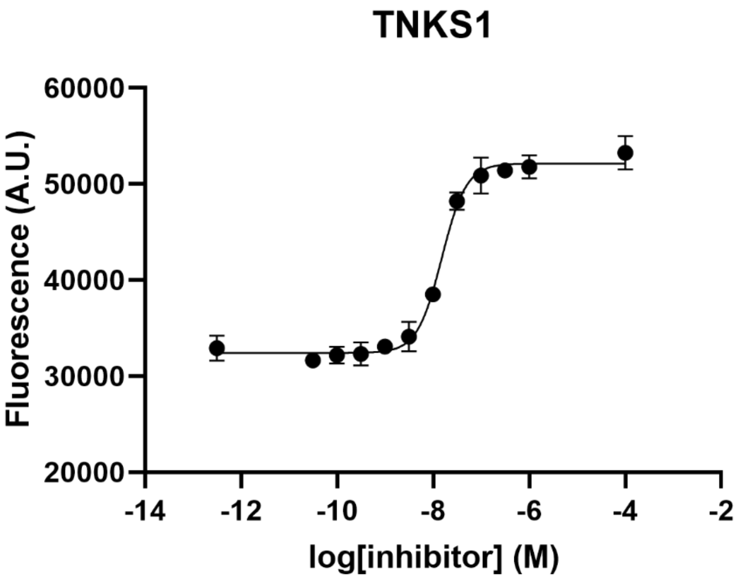

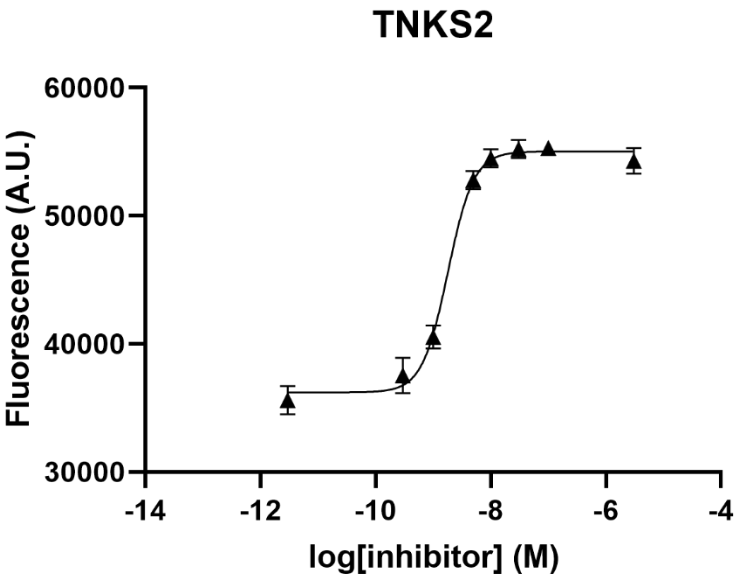

Figure S-1. Dose response curves for compound **24** (OM-153) in the biochemical assay of TNKS1/2 respectively with error bars showing the SD (n=3).

PARP	**1** (OM-1700)	**24** (OM-153)
PARP1	29 µM	> 100 µM
PARP2	26 µM	> 100 µM
PARP3	75 µM	> 100 µM
PARP4	> 100 µM	> 100 µM
PARP5a (TNKS1) [b]	125 nM (6.90 ± 0.05)	12.2 nM (7.914 ± 0.03)
PARP5b (TNKS2) [b]	14 nM (7.85 ± 0.04)	1.6 nM (8.79 ± 0.085)
PARP10	>> 10 µM	>> 10 µM
PARP12 [a]	>> 10 µM	>> 10 µM
PARP14	> 100 µM	> 100 µM
PARP15 [a]	>> 10 µM	>> 10 µM

[a] concentration limited by DMSO tolerance
[b] between brackets the pIC_{50} ± SEM is given
> under 50% inhibition
>> no inhibition detected

Table S-1. IC_{50} data of PARPs

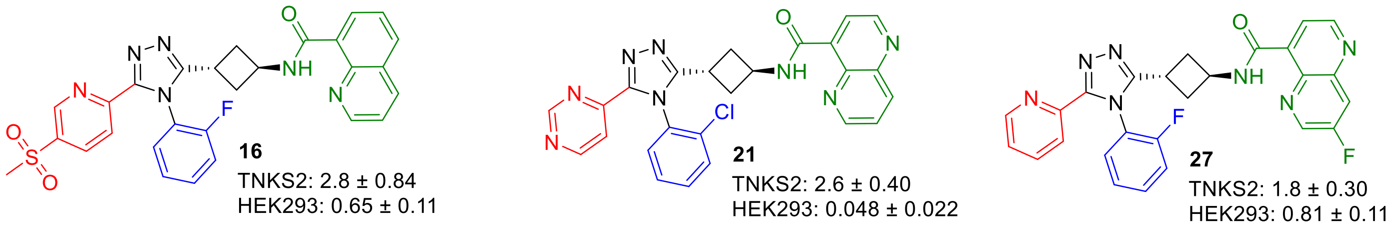

Chart S-1. Selected compounds from our previous paper[1]

**Compound**	**24** (OM-153)
PDB code	7O6X
Beam line	DLS I04
Wavelength (Å)	0.97949
Space group	$P2_12_12_1$
Cell dimensions a, b, c (Å)	41.39,<br>76.43,<br>147.73
Resolution (Å)	41.4 - 2.2 (2.279 - 2.2)
R_{merge}	0.2496 (1.482)
I / σI	8.19 (1.33)
Completeness (%)	99.74 (99.50)
Redundancy	13.4 (13.6)
**Refinement**	
R_{work} / R_{free}	0.2157 / 0.2543
**B-factors**	
Protein	41.5
Inhibitor	37.6
**R.m.s.d.**	
Bond lengths (Å)	0.013
Bond angles (°)	1.63
**Ramachandran plot (%)**	
Favored regions	99.48
Additionally allowed regions	0.52

Table S-2. Data collection and refinement statistics for the cocrystal structure of TNKS2 in complex with compound **24** (OM-153).

References

(1) Waaler, J.; Leenders, R. G. G.; Sowa, S. T.; Alam Brinch, S.; Lycke, M.; Nieczypor, P.; Aertssen, S.; Murthy, S.; Galera-Prat, A.; Damen, E.; Wegert, A.; Nazaré, M.; Lehtiö, L.; Krauss, S. Preclinical Lead Optimization of a 1,2,4-Triazole Based Tankyrase Inhibitor. *J. Med. Chem.* **2020**, *63* (13), 6834–6846.
